# APAV: An advanced pangenome analysis and visualization toolkit

**DOI:** 10.1371/journal.pcbi.1013288

**Published:** 2025-07-07

**Authors:** Xiaorui Dong, Du Jiao, Hongzhang Xue, Shiyu Fan, Chaochun Wei

**Affiliations:** Department of Bioinformatics and Biostatistics, School of Life Sciences and Biotechnology, Shanghai Jiao Tong University, Shanghai, China; Robert Koch Institute: Robert Koch Institut, GERMANY

## Abstract

Traditional pangenome analysis focuses on gene presence/absence variations (gene PAVs). However, the current methods for gene PAV analysis are insensitive to detect small but valuable mutations within gene regions, and they overlook variations in intergenic regions. Additionally, the visual inspection of PAVs is an important but time-consuming step for pangenome analysis and result interpretation. To address these issues, we present APAV, an advanced toolkit designed for comprehensive PAV analysis and visualization. It integrates gene element-level PAV analysis and provides PAV analysis for arbitrary given regions in a genome. The resulted PAV profile can be visualized and investigated interactively with reports in HTML format, enabling researchers to conveniently verify sequencing read depth, target region coverage, and intervals of absence for each PAV. Furthermore, APAV offers various subsequent analysis and visualization functions based on the PAV profile table, including basic statistics, sample clustering, genome size estimation, and phenotype association analysis. We demonstrated the capability of APAV with pangenome analysis of tumor genomes and rice genomes. Performing PAV analysis at the element level not only provides more accurate information about the variations but also uncovers a larger number of variations for the phenotype-genotype association studies. In the rice genome analysis, we identified over twenty thousand distributed genes and more than fifty thousand distributed genetic elements. In the tumor genome analysis, element-level analysis revealed approximately three times as many phenotype-related genes as gene-level analysis. This indicates that altering the PAV unit from genes to smaller segments or elements can lead to more biological insights.

## Author summary

A pangenome is the collection of all genetic information of a population. A pangenome can be much more complete than an individual genome. Using a pangenome as the baseline for genomics study is getting more and more prevalent. However, current methods using a pangenome as the reference focus on gene-level presence/absence variations, which will ignore variations in smaller but valuable genomic regions such as exons or gene fragments, which can be a tenth of a gene in size. The tool APAV we present here is designed to analyse and visualize presence/absence variations for genomic regions of any sizes, spanning from genes to any genomic regions of interest. This tool provides a pipeline suitable for analysing eukaryotic linear pangenomes, expands the target region from genes to any genomic regions of interest, and supports element-level analysis. Additionally, it offers a variety of subsequent analysis and visualization methods for PAVs, as well as an interactive report generation function.

## Introduction

The pangenome was first introduced to investigate the genetic diversity of a population or species of eukaryotes [1] and the concept has gradually been applied to plants and animals in recent years [2,3]. A key step in pangenome research is gene presence/absence variation (PAV) analysis, which provides a new dimension of genomic variations [4]. Due to the large sizes of the genomes and the huge number of individuals, the map-to-pan strategy was extensively employed to identify gene PAVs in eukaryotic pangenomes [5–8]. EUPAN [9] and HUPAN [8] determine gene PAVs by calculating the coverage of gene regions. PSVCP [10] identifies sequence PAVs by merging PAVs of 20 bp intervals. However, all of them offer minimal visualization functions. Panache [11] supports visualization of sequence PAVs on linear pangenomes, but it cannot perform PAV identification and requires a pre-calculated PAV matrix as input. PPanG [12] can display gene annotations together with a graph pangenome and its linear genomes simultaneously, but it does not support PAV identification or statistical analysis and requires pre-existing annotation data for all genomes. Gene PAV studies involve genes from the reference genome and non-reference genome sequences, and they will reveal the core and distributed genes. Nevertheless, current gene PAV analysis uses gene as the unit for presence/absence analysis, which is limited by its rough resolution and is not able to detect the impact of smaller presence/absence variations in exons, intragenic segments, gene upstream/downstream regions, and noncoding regions. PAVs in a finer resolution can lead to more accurate and richer insights for genomic studies. It is important to note that threshold selection and alignment artifacts can impact PAV determination. Therefore, visualization of PAVs plays an important role in reducing potential false positives. Although tools like Integrative Genomics Viewer (IGV) [13] and JBrowse2 [14] can display sequence alignments, the process can be cumbersome and slow when dealing with large numbers of PAVs.

Here we present APAV, an Advanced Presence/Absence Variation analysis and visualization toolkit, for the comprehensive PAV study. It contains a suite of tools for finding genomic PAVs at both the whole gene level and genic element level, together with various plotting functions and automatic report generating functions for PAV analysis. APAV can be applied to any given genomic region and any organism. We demonstrated the usage of APAV using the rice pangenome and gastric cancer pangenome as examples.

## Design and implementation

### Overview of APAV pipeline

**Fig 1:**
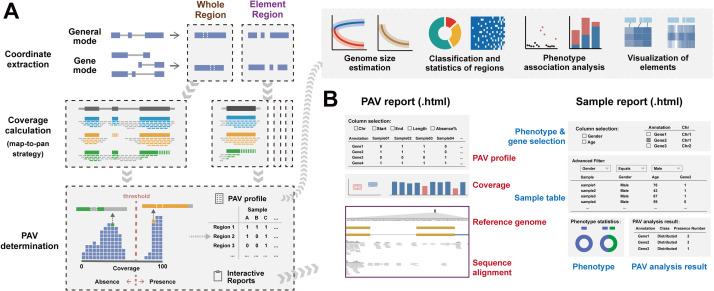
Overview of APAV pipeline. **(A) Workflow for PAV calling and consequent analysis.** The coordinates of the target region are first extracted from a GFF file or BED file. The coverage of both the whole region and the element region is calculated based on the BAM files. The PAVs are determined according to their coverage, and interactive reports are generated. Subsequent analyses can be performed using the PAV tables, including genome size estimation, classification and statistical analysis, phenotypic association analysis, and visualization of elements. **(B) Interactive analysis reports.** The PAV report presents PAV tables, coverage data, pangenome sequences, genome annotation, and sequence alignments. The sample report presents sample tables, phenotype information, and real-time PAV analysis results.

APAV operates in two modes: a gene mode for gene and genic element PAVs, and a general mode for any target region and its associated element PAVs (Figs 1 and S1). Users can run APAV using either a pre-built one-step pipeline or step-by-step operations. The main steps can be executed automatically using the “*geneBatch*” or “*generalBatch*” command. The “*geneBatch*” command is specifically designed for gene regions and requires a GFF file and BAM files as inputs. The “*generalBatch*” command applies to any target region and takes a BED file and BAM files as input. This process involves extracting coordinates, computing coverage, identifying PAVs, generating reports in HTML format, and producing preview figures. Users can use APAV’s visualization commands in a Linux environment or utilize the APAVplot package in an R environment for further visualization.

### Coordinate extraction and coverage calculation

The “*gff2bed*” command extracts the coordinates of genes and genetic elements (exons, CDSs, UTRs, upstream and downstream regions) from an input genome annotation file in GFF format, then outputs the results to a BED file (S2 Fig). For genes with multiple transcripts, overlapping elements with identical coordinates are consolidated to eliminate redundant information. For other annotated target regions on the genome, only a BED file is required.

The “*staCov*” command calculates the coverage of target regions and element regions based on BAM files. It calculates sequencing depth using SAMtools depth [15] and determines the coverage by counting the percentage of bases covered within the target region. For general target regions, discontinuous regions with the same annotation are treated as distinct elements. By adding the “*--asgene*” parameter, the target regions are treated as genes. This command provides six options for selecting representative transcripts: the transcript with the longest CDS region, the transcript with the longest exon region, the longest transcript, the transcript with the highest coverage, all transcripts, and the entire gene body (S3 Fig). Neighboring elements with the same coverage can be further merged by adding the corresponding parameter. The “*covPlotHeat*” visualization command presents an overview of coverage across samples in a detailed heatmap (S4 Fig).

### PAV determination

The coverage profile can be used as input to the “*callPAV*” command to determine the PAVs for both target regions and element regions (S4 Fig). This command offers two methods for classifying presence/absence categories: fixed threshold and adaptive threshold for coverage. The fixed threshold is provided by the user. Values exceeding the threshold are recognized as present, while those below are considered absent. The adaptive threshold method is based on clustering coverage across all samples in each region, categorizing groups with higher coverages as present and those with lower coverages as absent. The process eventually generates PAV tables and two interactive reports for visual view (Fig 1B). The reports are stored in a new folder, which includes the PAV report, the sample report, JS files, and data files. The PAV report allows researchers to query and filter the PAV results. For any region of interest, users can click on the corresponding table row to view the coverage distribution of presence and absence groups in a box plot. In addition, a bar chart is provided to examine the values for each sample. The sequence alignments for specific samples can be checked in a genome browser. This genome browser offers pangenome sequences, a track for displaying the target region, annotation, and alignment tracks. The browser was constructed based on the JBrowse2 framework [14].

Additionally, based on the gene PAV profile, researchers can obtain the gene family PAV profile using the “*gFamPAV*” command. A gene family is considered present if at least one of its member genes is present. Like the gene PAV profile, the gene family profile can be used as input data for subsequent analyses.

### Genome size estimation

The “*pavSize*” command simulates random combinations of genomes to estimate the size of the pangenome and core genome based on the PAV table. During each estimation, a random selection of genomes (ranging from one to all) will be made without replacement, and the number of pangenomes and core genomes in these samples will be counted. The estimation can also be performed in groups. The “*pavPlotSize*” visualization command takes the output of the “*pavSize*” command and draws the growth curve of genome size estimation. It offers three types of charts: line plot with error bars, jitter plot and ribbon chart.

### Classification and statistics of regions

Target regions can be divided into core regions (which are present in all individuals) and distributed regions (which are not present in all individuals). The distributed regions can be further classified into softcore regions, distributed regions and private regions. Various complex charts provide insight into the classifications and distribution of target regions. The “*pavPlotStat*” visualization command displays the number of target regions. The “*pavPlotHist*” visualization command visualizes the classifications and distribution of the target regions. The “*pavPlotHeat*” visualization command presents an overview of the PAV profile in a complex heatmap. The “*pavPlotBar*” command shows the proportions of different categories. The “*pavPCA*” visualization command performs PCA analysis on the PAV table. The “*pavCluster*” visualization command clusters samples based on the PAV table.

### Phenotype association analysis

The “*pavStaPheno*” command enables users to conduct phenotype association analysis of distributed target regions, facilitating the exploration of the potential biological impact of PAVs. For phenotypes with discrete values, the command conducts Fisher’s exact test to assess the independence of phenotype groups and PAV categorization. For phenotypes with continuous values, it uses the Wilcoxon rank sum test to compare the coverage of samples with presence variations to those with absence variations. The findings can be viewed using various visualization commands. The “*pavPlotPhenoHeat*” command visualizes the outcomes of a phenotype association analysis as a heatmap. The “*pavPlotPhenoBlock*” command illustrates the proportion of individuals exhibiting particular regions across various groups. The “*pavPlotPhenoMan*” command generates a Manhattan plot that displays the outcomes of a selected phenotype. The “*pavPlotPhenoBar*” and “*pavPlotPhenoVio*” commands emphasize the relationship between a specific genetic region and a distinct phenotype.

### Visualization of elements

To visualize the presence/absence of elements within the target region effectively, APAV offers three commands for visualization at different levels. The “*elePlotCov*” command shows element coverage, the “*elePlotPAV*” command displays the PAV of elements, and the “*elePlotDepth*” visualization command illustrates the depth of elements. We created a composite graph type to effectively present various data for elements. The main body of this image is a heatmap that displays coverage, PAV, or depth for all elements. Additionally, annotations can be added alongside the heatmap to provide phenotypic information about the samples. The top of the heatmap indicates the coordinates of all elements and gene annotations.

### R package APAVplot

We developed APAVplot, an R package focused on visualization for pangenome analysis. All visualization commands in APAV call functions from APAVplot. This package runs in the R environment, which offers enhanced interactivity and user experience. Researchers can analyze and observe PAV data more easily and flexibly. The primary input consists of the coverage profile, the PAV profile, and phenotype information, which can be obtained through APAV or prepared by the user. APAVplot covers a variety of commonly used analysis and visualization methods in pangenome PAV analysis. It can display the distribution of gene coverage, conduct PAV common analysis, perform phenotype association analysis, draw growth curves for genome estimations, and illustrate element regions. These figures are mainly drawn using the R package ggplot2 [16] and ComplexHeatmap [17]. APAVplot provides richer parameter settings and graphical content, which can be used to show results from APAV or be used independently.

## Results

We developed APAV, which offers enhanced visualization capabilities and expands PAV analysis from gene regions to element regions. To demonstrate its capabilities, we applied it to two datasets for pangenome analysis.

**Fig 2:**
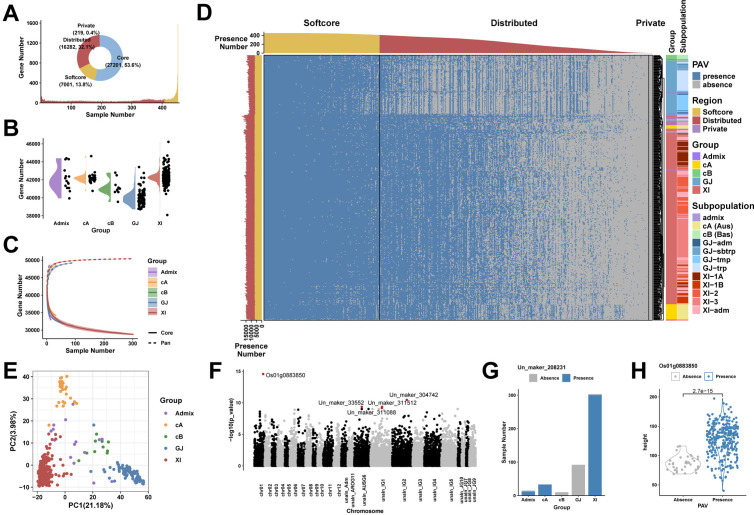
Visualization of pangenome analysis results in rice genomes. **(A) Proportion and distribution of core, softcore, distributed, and private genes.** The proportion is shown in the pie chart and the distribution of gene numbers is shown in the bar plot. **(B) Distribution of gene counts across accession groups.** Each point in the figure represents a sample. **(C) Pangenome size estimation.** The pangenome and core-genome sizes are drawn for different accession groups of rice samples. **(D) The heatmap of the PAV profile**. The annotations above and to the left indicate the total number of genes. Each row of the heatmap is for a sample and each column is for a gene. **(E) PCA results of the distributed gene PAV.** Each point in the figure represents a sample. **(F) Manhattan plot of phenotype association analyses for plant height.** The five points with the highest p-values are marked with red dots. **(G) An example illustrating the correlation between phenotypic groups and the gene PAV. (H) An example displaying phenotypic differences between presence and absence group samples.**

**Fig 3:**
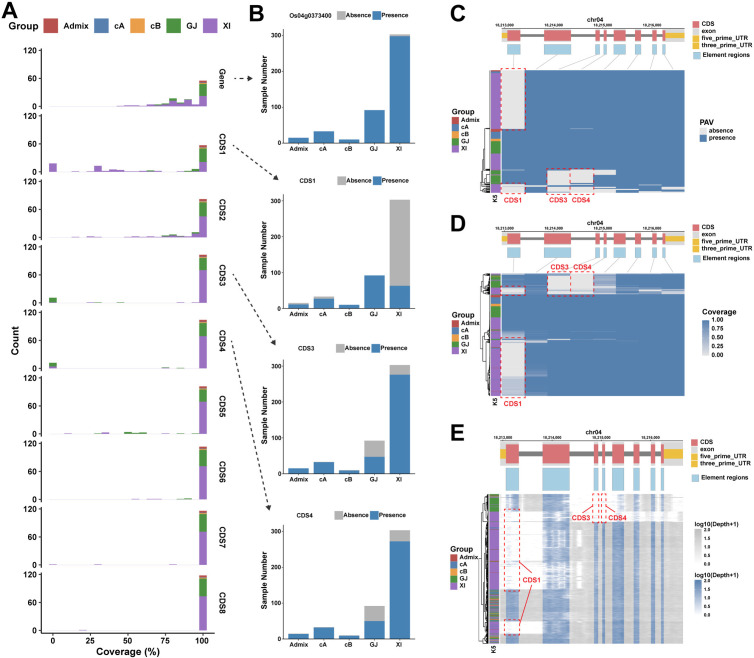
Element-level analysis for *Os04g0373400* gene in the rice genome. **(A) Coverage distribution of the gene and all CDSs**. **(B) The number of samples with gene/CDS absence and presence within each rice group.** Significant associations are observed between the rice groups and CDS1, CDS3, and CDS4 (all *p* < 0.001, Fisher’s test). **(C-E) The heatmap for element level PAVs in terms of PAV(C), coverage (D), and sequence alignment depth (E) of elements in the *Os04g0373400* gene.** The absent regions of corresponding samples are highlighted with red dashed boxes.

In the first dataset, we conducted the PAV analysis on 453 rice genomes with high sequencing depth. The accessions were categorized into five major groups: XI, GJ, cA, cB, and admixed. We calculated the coverage of genes and elements (including exons, CDSs, and UTRs) and determined PAV at a threshold of 50%. A total of 23,502 distributed genes (Fig 2A) and 50,492 distributed elements were identified. The half-violin plot indicated that the XI accessions had the highest average gene count, while the GJ accessions had the lowest (Fig 2B). Additionally, various groups exhibited distinct growth patterns in the genome size estimation (Fig 2C). The heatmap illustrates the PAV gene profile of major groups (Fig 2D). The PCA and clustering results showed that the major rice groups could be distinguished based on PAVs of distributed genes (Fig 2D-E). Phenotype association analyses were performed for both gene PAVs and element PAVs concerning plant height and accession groups. At the element level, we found more regions related to phenotypes. Compared to the 20 genes identified by gene level, the element level reported 37 elements related to 27 genes significantly associated with plant height (*p* < 1e-5), including 19 out of 20 genes identified by gene level and additional 8 genes (S5 Fig). A similar behavior exists for accession groups. A notable example is the *Os04g0373400* gene, which encodes a multidrug and toxic compound extrusion (MATE) protein involved in various physiological functions of plant growth [18,19]. Element level PAV analysis revealed its association with accession groups, which was overlooked in gene PAV analysis (Fig 3). The first CDS was primarily absent in the XI group (adjusted *p* = 3.95e-56), while the GJ group lost proportionately more of the third CDS (adjusted *p* = 2.01e-15) and the fourth CDS (adjusted *p* = 6.21e-12). Another example is the *sd1* gene, often referred to as the green revolution gene, which was highly correlated with plant height as reported previously. Element level PAV analysis further indicated that the absence of its first CDS region contributed to this association (adjusted *p* = 0.0052) (S6 Fig).

In the second dataset, we obtained genomic data from a previous study involving 127 male gastric cancer patients and aligned them to the gastric cancer pangenome [20]. Based on the BAM files, we used APAV to identify, analyze, and visualize PAVs (S7 Fig). Firstly, we extracted the coordinates of all genetic elements from the annotation file, including exons, CDSs, UTRs, and 100*10 bp upstream and downstream regions. We then selected the transcript with the longest CDS region as a representative transcript to calculate the coverage of genes and genetic elements. Regions with coverage exceeding 80% were considered to be present. Eventually, we identified 208 distributed genes. We estimated the genome size, illustrated the distribution of genes in the population, and performed phenotypic association analyses for all distributed genes and genetic elements. Additionally, we compared the results at both the gene level and the element (CDS and UTR) level (S8 Fig). Analyzing at the element level allowed us to identify shorter PAV variants, providing an alternative perspective and form of variation for studying genetic diversity (S8A Fig). This approach also made the absence of elements more apparent (S8D Fig). Furthermore, we discovered more potential associations with phenotypes in regions analyzed at smaller resolutions (S8C Fig). For example, the *PIM3* gene, a member of the proto-oncogene PIM family with serine/threonine kinase activity [21], was related to gastric adenoma-adenocarcinoma sequence and progression in gastric cancer [22]. Our results indicated that the absence of the *PIM3* gene mainly occurs in the CDS region of the sixth exon (S9 Fig). Notably, all of the absent samples had an *Epstein-Barr virus* (EBV) infection diagnosis. The expression level of *PIM3* can be validated by RNA-seq, and it is found that tumor samples with exon 6 deletion revealed a lower expression level. Additionally, we also found that the 501–600 bp region upstream of the gene correlated with *Helicobacter pylori* (HP) infection, and the downstream 401–500 bp was associated with Bormann classification.

The above examples demonstrate the powerful visualization capabilities of APAV, which can be applied for both data exploration and final result plotting. APAV also reveals the potential of element level PAV analysis. By expanding PAV analysis from the gene level to the element level, we can uncover a large number of additional variations associated with phenotypes, which can be critical in applications like disease genomics.

## Availability and future directions

The source code for APAV can be accessed freely at https://github.com/SJTU-CGM/APAV, while the source code for the R package APAVplot can be accessed freely at https://github.com/SJTU-CGM/APAVplot. Demo datasets are available for download from https://github.com/SJTU-CGM/APAV/tree/main/demo. Additionally, software documentation, as well as code and data for reproducing case studies, can be accessed at https://cgm.sjtu.edu.cn/APAV/.

APAV is primarily used for constructing linear pangenomes by combining reference genomes with non-reference sequences derived from next-generation sequencing data. In the future, we plan to incorporate new methods to support variant analysis of pangenomes created through whole-genome alignment strategies, which are typically employed in third-generation sequencing data. These improvements will enable the detection of a wider variety of variant types.

## Supporting information

S1 Text Usage Guide of APAV. (DOCX)

S2 Text Usage Guide of APAVplot. (DOCX)

S3 Text The code for the case study analysis on rice and cancer datasets. (DOCX)

S1 Fig The workflow of APAV. (DOCX)

S2 Fig Schematic diagram of the “gff2bed” command. (DOCX)

S3 Fig Description of the main parameters of the “staCov” command. (DOCX)

S4 Fig Description of the parameters of the “callPAV” command. (DOCX)

S5 Fig Comparison of results at the gene level and element level in the rice genomes. (DOCX)

S6 Fig Element-level analyses for *sd1*(*Os01g0883800*) gene. (DOCX)

S7 Fig Visualization of pangenome analysis results in tumor genomes. (DOCX)

S8 Fig Comparison of results at the gene level and element level in the tumor genomes. (DOCX)

S9 Fig Element-level analyses for the *PIM3* gene. (DOCX)

## References

[1] Tettelin H, Masignani V, Cieslewicz MJ, Donati C, Medini D, Ward NL, et al. Genome analysis of multiple pathogenic isolates of Streptococcus agalactiae: implications for the microbial “pan-genome”. Proc Natl Acad Sci U S A. 2005;102(39):13950–5. doi: 10.1073/pnas.0506758102 16172379 PMC1216834

[2] Sherman RM, Salzberg SL. Pan-genomics in the human genome era. Nat Rev Genet. 2020;21(4):243–54. doi: 10.1038/s41576-020-0210-7 32034321 PMC7752153

[3] Shi J, Tian Z, Lai J, Huang X. Plant pan-genomics and its applications. Mol Plant. 2023;16(1):168–86. doi: 10.1016/j.molp.2022.12.009 36523157

[4] Vernikos G, Medini D, Riley DR, Tettelin H. Ten years of pan-genome analyses. Curr Opin Microbiol. 2015;23:148–54. doi: 10.1016/j.mib.2014.11.016 25483351

[5] Hu Z, Wei C, Li Z. Computational Strategies for Eukaryotic Pangenome Analyses. In: Tettelin H, Medini D, editors. The Pangenome: Diversity, Dynamics and Evolution of Genomes. Cham (CH): Springer. Copyright 2020, The Author(s). 2020. p. 293–307.

[6] Zhang F, Xue H, Dong X, Li M, Zheng X, Li Z, et al. Long-read sequencing of 111 rice genomes reveals significantly larger pan-genomes. Genome Res. 2022;32(5):853–63. doi: 10.1101/gr.276015.121 35396275 PMC9104699

[7] Li Z, Liu X, Wang C, Li Z, Jiang B, Zhang R, et al. The pig pangenome provides insights into the roles of coding structural variations in genetic diversity and adaptation. Genome Res. 2023;33(10):1833–47. doi: 10.1101/gr.277638.122 37914227 PMC10691484

[8] Duan Z, Qiao Y, Lu J, Lu H, Zhang W, Yan F, et al. HUPAN: a pan-genome analysis pipeline for human genomes. Genome Biol. 2019;20(1):149. doi: 10.1186/s13059-019-1751-y 31366358 PMC6670167

[9] Hu Z, Sun C, Lu K-C, Chu X, Zhao Y, Lu J, et al. EUPAN enables pan-genome studies of a large number of eukaryotic genomes. Bioinformatics. 2017;33(15):2408–9. doi: 10.1093/bioinformatics/btx170 28369371

[10] Wang J, Yang W, Zhang S, Hu H, Yuan Y, Dong J, et al. A pangenome analysis pipeline provides insights into functional gene identification in rice. Genome Biol. 2023;24(1):19. doi: 10.1186/s13059-023-02861-9 36703158 PMC9878884

[11] Durant É, Sabot F, Conte M, Rouard M. Panache: a web browser-based viewer for linearized pangenomes. Bioinformatics. 2021;37(23):4556–8. doi: 10.1093/bioinformatics/btab688 34601567 PMC8652104

[12] Liu M, Zhang F, Lu H, Xue H, Dong X, Li Z, et al. PPanG: a precision pangenome browser enabling nucleotide-level analysis of genomic variations in individual genomes and their graph-based pangenome. BMC Genomics. 2024;25(1):405. doi: 10.1186/s12864-024-10302-5 38658835 PMC11044437

[13] Thorvaldsdóttir H, Robinson JT, Mesirov JP. Integrative Genomics Viewer (IGV): high-performance genomics data visualization and exploration. Brief Bioinform. 2013;14(2):178–92. doi: 10.1093/bib/bbs017 22517427 PMC3603213

[14] Diesh C, Stevens GJ, Xie P, De Jesus Martinez T, Hershberg EA, Leung A, et al. JBrowse 2: a modular genome browser with views of synteny and structural variation. Genome Biol. 2023;24(1):74. doi: 10.1186/s13059-023-02914-z 37069644 PMC10108523

[15] Danecek P, Bonfield JK, Liddle J, Marshall J, Ohan V, Pollard MO, et al. Twelve years of SAMtools and BCFtools. Gigascience. 2021;10(2):giab008. doi: 10.1093/gigascience/giab008 33590861 PMC7931819

[16] Wickham H. ggplot2: Elegant Graphics for Data Analysis. New York: Springer-Verlag. 2016.

[17] Gu Z, Eils R, Schlesner M. Complex heatmaps reveal patterns and correlations in multidimensional genomic data. Bioinformatics. 2016;32(18):2847–9. doi: 10.1093/bioinformatics/btw313 27207943

[18] Upadhyay N, Kar D, Deepak Mahajan B, Nanda S, Rahiman R, Panchakshari N, et al. The multitasking abilities of MATE transporters in plants. J Exp Bot. 2019;70(18):4643–56. doi: 10.1093/jxb/erz246 31106838

[19] Du Z, Su Q, Wu Z, Huang Z, Bao J, Li J, et al. Genome-wide characterization of MATE gene family and expression profiles in response to abiotic stresses in rice (Oryza sativa). BMC Ecol Evol. 2021;21(1):141. doi: 10.1186/s12862-021-01873-y 34243710 PMC8268253

[20] Yu Y, Zhang Z, Dong X, Yang R, Duan Z, Xiang Z, et al. Pangenomic analysis of Chinese gastric cancer. Nat Commun. 2022;13(1):5412. doi: 10.1038/s41467-022-33073-7 36109518 PMC9477819

[21] Konietzko U, Kauselmann G, Scafidi J, Staubli U, Mikkers H, Berns A, et al. Pim kinase expression is induced by LTP stimulation and required for the consolidation of enduring LTP. EMBO J. 1999;18(12):3359–69. doi: 10.1093/emboj/18.12.3359 10369676 PMC1171416

[22] Zheng H-C, Tsuneyama K, Takahashi H, Miwa S, Sugiyama T, Popivanova BK, et al. Aberrant Pim-3 expression is involved in gastric adenoma-adenocarcinoma sequence and cancer progression. J Cancer Res Clin Oncol. 2008;134(4):481–8. doi: 10.1007/s00432-007-0310-1 17876606 PMC12161629

