# Supplementary material for: APAV: An advanced pangenome analysis and visualization toolkit
Source: PLoS Comput Biol. 2025 Jul 7;21(7):e1013288. doi: 10.1371/journal.pcbi.1013288 (PMC12251200; doi:10.1371/journal.pcbi.1013288)
Supplement: S1 Text — (DOCX) [file pcbi.1013288.s001.docx]

**S1 Text: Supporting information for “APAV: An advanced pangenome analysis and visualization toolkit”**

**Usage Guide of APAV**

1. **Workflow**

APAV is implemented in Perl and R for Linux platforms (Fig S1). Complete details are available at <https://github.com/SJTU-CGM/APAV>. A listing of all commands can be obtained with “*apav --help*” or “*apav -h*”. The usage information for each command can be shown with the “*--help*” or “*-h*” option after each command name. A comprehensive list of parameters for all commands is also available at <https://cgm.sjtu.edu.cn/APAV/usage.html>.

Usage: apav <command> ...

Available commands:

| Pipeline | geneBatch | Automatically execute main commands for genes |
| --- | --- | --- |
|  | generalBatch | Automatically execute main commands for the general target regions |
| Extract positions | gff2bed | Extract the coordinates of target regions from a GFF format file |
| Calculate coverage | staCov | Calculate coverage of target regions |
|  | mergeElecov | Merge neighboring elements with the same coverage |
|  | covPlotHeat | Plot a heatmap to give an overview of the coverage profile across samples |
| Determine PAV | callPAV | Determine presence/absence variations based on coverage |
|  | gFamPAV | Determine gene family presence/absence based on the gene PAV table |
|  | mergeElePAV | Merge neighboring elements with the same PAV |
| Estimate genome size | pavSize | Estimate the size of pangenome and core genome from the PAV table |
|  | pavPlotSize | Draw estimated growth curves |
| PAV analysis | pavPlotStat | Plot a half-violin chart to show the number of regions in each group of samples |
|  | pavPlotHist | Plot a ring chart and a histogram to show the classification and distribution of target regions |
|  | pavPlotHeat | Plot a complex heat map to give an overview of the PAV profile |
|  | pavPlotBar | Plot a stacked bar chart to show the classifications of target regions in all samples |
|  | pavPCA | Perform PCA analysis for the PAV table and plot results |
|  | pavCluster | Cluster samples based on the PAV table and plot results |
| Phenotype association | pavStaPheno | Perform Fisher's exact test and Wilcoxon tests to determine phenotype association |
|  | pavPlotPhenoHeat | Show the main result of phenotype association analysis with a heat map |
|  | pavPlotPhenoBlock | Display the percentage of samples containing target regions in each group of a discrete phenotype |
|  | pavPlotPhenoMan | Draw a Manhattan plot to show the results of a given phenotype |
|  | pavPlotPhenoBar | Show the correlation between a specific genomic region and a specific phenotype in a bar plot |
|  | pavPlotPhenoVio | Show the correlation between a specific genomic region and a specific phenotype in a violin plot |
| Visualization of element regions | elePlotCov | Display the coverage of elements in a specific target region |
|  | elePlotPAV | Display the PAV of elements in a specific target region |
|  | elePlotDepth | Display the depth of elements in a specific target region |

1. **Integrated commands**

The process can be automated with the “*geneBatch*” or “*generalBatch*” command, which entails extracting coordinates, computing coverage, identifying PAVs, and generating web reports, along with default preview figures. Users can also use APAV's visualization commands in a Linux environment or the APAVplot R package in R for enhanced visualization.

After running the command, you will get the following results:

| A file with the suffix “.bed” | | Coordinates of genetic elements. (only for the “geneBatch” command) |
| --- | --- | --- |
| The files with the suffix “.cov” and “_ele.cov” | | Coverage profile of target region and element region. |
| A file with the suffix “_ele.mcov” | | Coverage profile of merged element region. (with the “--merge” option) |
| The files with the suffix “_all.pav” and “_ele_all.pav” | | PAV profile of target region and element region. |
| The files with the suffix “_dispensable.pav” and “_ele_dispensable.pav” | | PAV profile of dispensable region. |
| A file with the suffix “.fpav” | | PAV profile of gene family. (only for the “geneBatch” command with the “--fam” option) |
| A folder with the suffix “_report” | A file named “PAV” | A web report for PAV profile. |
|  | A file named “Sample” | A web report for samples. |
|  | A folder named “js” | JS files. |
|  | A folder named “css” | CSS files. |
|  | A folder named “browser” | Data for tracks in the genome browser. |
| A folder with the suffix “_ele_report” | A file named “PAV” | An interactive web report for PAV profile. |
|  | A file named “Sample” | An interactive web report for samples. |
|  | A folder named “js” | JS files. |
|  | A folder named “css” | CSS files. |
| A folder named “estimation” | A file with the suffix “.size” | The result of estimation. |
|  | A PDF file with the suffix “_size_curve” | The growth curve of genome estimation. |
| A folder named “coverage_visualization” | A PDF file with the suffix “_cov_heatmap” | A heatmap of coverage profile. |
| A folder named “common_analysis” | A PDF file with the suffix “_all_pav_sta” | A half-violin chart to show the number of regions. |
|  | A PDF file with the suffix “_all_pav_hist” | A ring chart and a histogram to show the classifications and distribution of target regions. |
|  | A PDF file with the suffix “_all_pav_stackbar” | A stacked bar chart to show the classifications of target regions in all samples. |
|  | A PDF file with the suffix “_dispensable_pav_cluster” | A circular clustering tree for the samples. |
|  | A PDF file with the suffix “_dispensable_pav_pca” | A scatter plot presenting the results of the PCA analysis |
| A folder named “phenotype_association” | A file with the suffix “.phenores” | The result of phenotype association. |
|  | The PDF files with the suffix “_manhattan” | Manhattan plots for phenotypes. |
|  | The PDF files with the suffix “_block” | The chart drawn by “pavPlotPhenoBlock” command for discrete phenotypes. |
|  | A folder named “example” | Some examples to show the effect of commands “pavPlotPhenoBar” and “pavPlotPhenoVio”. |
| A folder named “element_visualization” | | Some examples to show the effect of commands “elePlotCov”, “elePlotPAV”, and “elePlotDepth”. |

**Examples:**

| $ cd ${APAV_PATH}/demo/ |
| --- |

| *## demo1: Some genes on human chromosome 19*  $ apav geneBatch --gff demo1_gene.gff3 --bamdir bam --pheno demo_sample.pheno --fa demo.fa.gz --fam demo1_gene.fam --up_n 10 --down_n 10 --chrl demo1.chrl --out demo1 |
| --- |

| *## demo2: Some proteins on human chromosome 19*  $ apav generalBatch --bed demo2_general.bed --bamdir bam --pheno demo_sample.pheno --fa demo.fa.gz --out demo2 |
| --- |

| *## demo3: Some repeat sequences on human chromosome 19*  $ apav generalBatch --bed demo3_general.bed --bamdir bam --pheno demo_sample.pheno --fa demo.fa.gz --rmele --out demo3 |
| --- |

1. **Coordinate extraction**

When working with simple annotated target regions on the genome, you can use the BED file directly and skip this step. However, in most cases, the target region of interest is the gene, which is a complex annotated structure within the genome. The “*gff2bed*” command is used to extract the coordinates of genes and genetic elements from a GFF format file and then outputs them to a BED file for subsequent steps (Fig S2).

Genetic elements with the same coordinates will be consolidated to remove redundancy:

- If an exon contains only CDS or UTR, it is labeled as “[exonN(CDS)]”, “[exonN(3UTR)]” or “[exonN(5UTR)]”.
- If an exon contains multiple components, it is described as “[exonN:CDS]”, “[exonN:3UTR]” or “[exonN:5UTR]”.
- If an exon is present in more than one transcript, the information is combined into “[TN:exonN:CDS,TN:exonN:CDS]”.

Additionally, coordinate information for upstream and downstream gene fragments can be extracted, represented as “[UP:N-N]” and “[DOWN:N-N]”.

**Examples:**

| *## Extracting the exon, CDS and UTR*  $ apav gff2bed --gff demo1_gene.gff3  *## Output: demo1_gene.bed* |
| --- |

| *## Adding elements in upstream and downstream*  $ apav gff2bed --gff demo1_gene.gff3 --chrl demo1.chrl --up_n 10 --up_bin 100 --down_n 10 --down_bin 100  *## Output: demo1_gene.bed* |
| --- |

1. **Coverage calculation**

The “*staCov*” command is used to calculate the coverage of target regions and element regions by counting the percentage of covered bases (Fig S3). It calls “*samtools depth*” to compute the read depth at each position in the BAM files specified in the “*--bamdir*” parameter. A base is considered covered when the number of mapped reads exceeds the value specified in the “*--mincov*” parameter.

For genes, it offers six methods for selecting representative transcripts:

- “cdslen”: using the transcript with the longest CDS region as the target region, and calculating coverage for the corresponding elements;
- “exonlen”: using the transcript with the longest exon region as the target region, and calculating coverage of the corresponding elements;
- “len”: using the longest transcript as the target region;
- “highcov”: using the transcript with the highest coverage as the target region;
- “none”: considering the entire gene body as the target region;
- “all”: including all transcript regions, but calculating coverage only for the elements.

For general target regions, discontinuous regions annotated with the same annotation are treated as distinct elements. The “*staCov*” command calculates both the coverage of the entire region and the coverage of the elements.

**Examples:**

| *## Gene mode*  $ apav staCov --bed demo1_gene.bed --bamdir bam --asgene --rep cdslen  *## Output: demo1_gene.cov, demo1_gene_ele.cov* |
| --- |

| *## General mode*  $ apav staCov --bed demo2_general.bed --bamdir bam  *## Output: demo2_general.cov, demo2_general_ele.cov* |
| --- |

Additionally, the “*mergeEleCov*” command can merge neighboring regions with the same coverage (either all 0 or all 1). This sub-command is included within the “*staCov*” command and will be executed automatically when the “*--merge*” option is added.

For genes, the merged elements are symbolized as follows:

- When merging CDS and UTR on the same exon, they are represented as “exonN:(CDS&3UTR)” or “exonN:(CDS&5UTR)”;
- When merging neighboring exons on the same transcript, they are represented as “exonN(CDS)+exonM(CDS)”;
- When merging upstream or downstream elements, they are represented as “UP:N-N” or “DOWN:N-N”.

For general target regions, the merged elements are represented as “REGION:[N-N]”.

**Examples:**

| *## Adding `--merge` option to `staCov` command*  $ apav staCov --bed demo1_gene.bed --bamdir bam --asgene --merge --out demo1_m  *## Ouput: demo1_m.cov, demo1_m_ele.cov, demo1_m_ele.mcov* |
| --- |

| *## Using `mergeEleCov` command*  $ apav mergeEleCov --elecov demo1_gene_ele.cov --asgene  *## Output: demo1_gene_ele.mcov* |
| --- |

The “*covPlotHeat*” visualization command provides an overview of coverage across samples in a complex heatmap. It calls R scripts and generates a PDF file. Users can display phenotype data alongside the heatmap using the “*--pheno*” option. The usage information and a complete parameter list can be obtained with “*apav covPlotHeat --help*” or “apav covPlotHeat -h”.

**Examples:**

| $ apav covPlotHeat --cov demo1_gene.cov |
| --- |


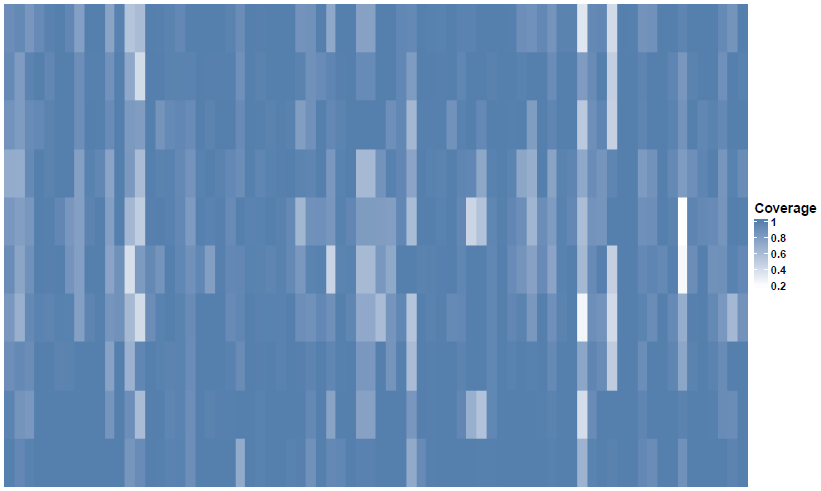


| *## Default palette*  $ apav covPlotHeat --cov demo1_gene.cov \  --pheno demo_sample.pheno \  --cluster_rows --cluster_columns \  --show_row_names --row_names_side right  *## Custom colors*  $ apav covPlotHeat --cov demo1_gene.cov \  --pheno demo_sample.pheno \  --cluster_rows --cluster_columns \  --show_row_names --row_names_side right \  --cov_colors 'white,#D98D8D' \  --pheno_info_color_list Location=#EDD12F,#C783C7 \  --pheno_info_color_list Age=#C6E2FF,#6CA6CD |
| --- |


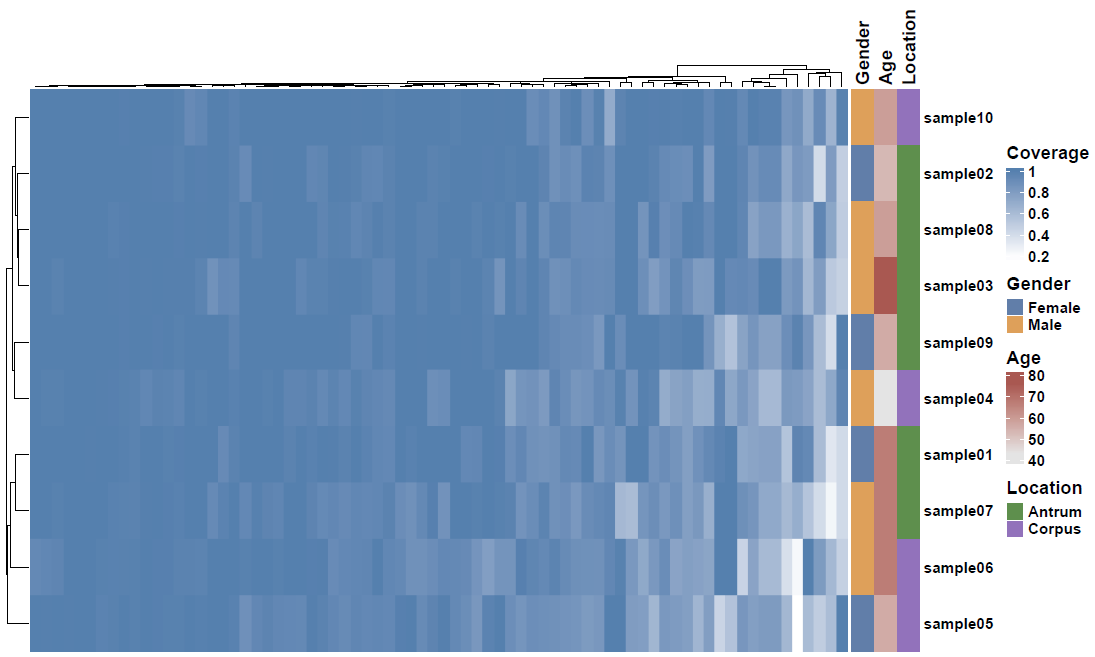


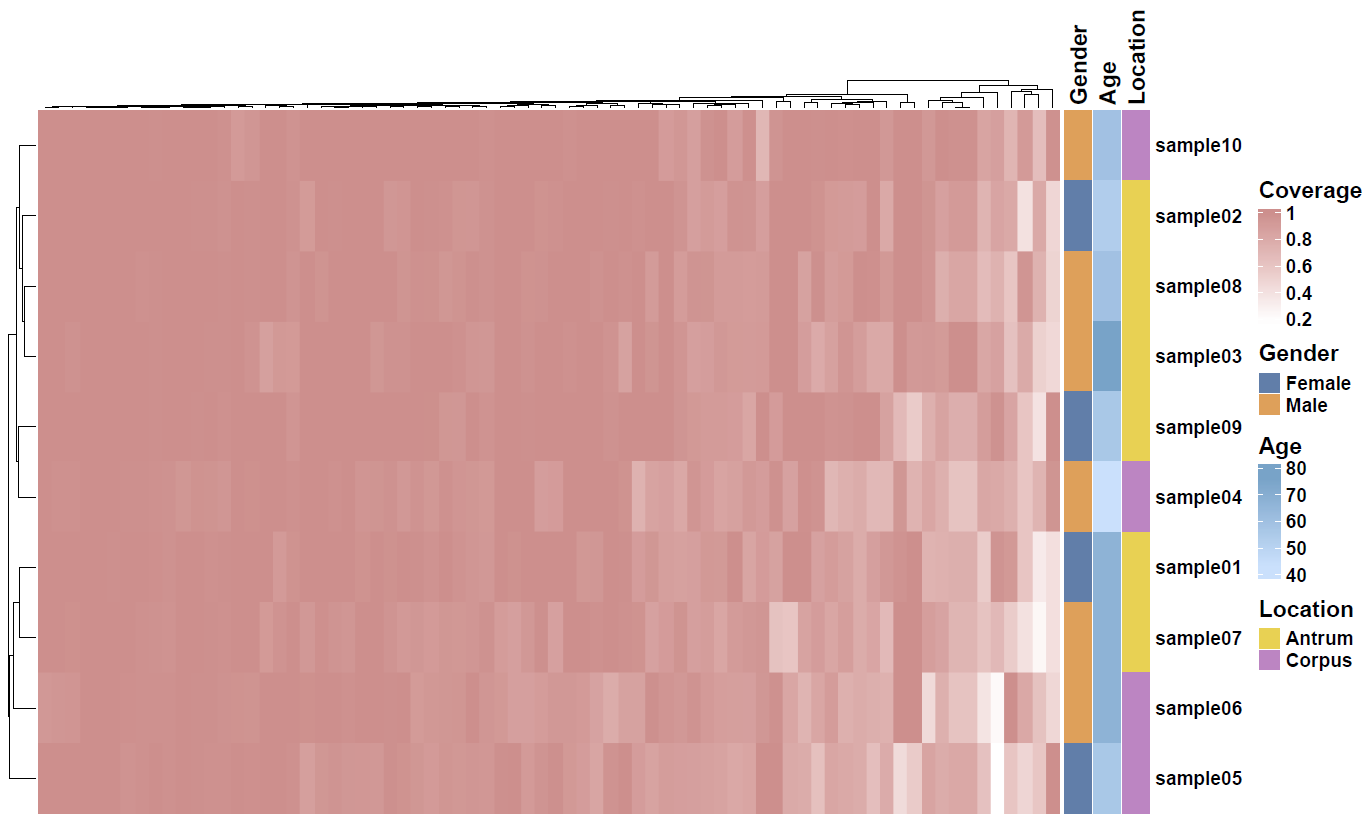


1. **PAV determination**

The “*callPAV*” command is used to determine presence or absence based on coverage (Fig S4). It offers two methods for PAV determination:

- Using a fixed threshold: Values greater than the threshold set by the “*-t*” parameter are classified as present, while values below the threshold are classified as absent.
- Using an adaptive threshold for each unit. If the sample with the highest uncovered percentage is smaller than the “*-a*” parameter, all samples will be considered present. Otherwise, a clustering process is employed to identify the threshold. The clustering process involves the following steps:

    1. Select the maximum and minimum values as the two centers of subgroups, then divide the other values into two groups based on their distance from the centers.

    2. Recalculate the median of each group to establish new centers.

    3. Regroup the values according to their distances from the new centers.

4. Repeat the above steps until the grouping is stable (or the iteration count reaches the maximum threshold specified by the “*-n*” parameter). Samples with smaller values are determined to be absent, while samples with larger values are determined to be present.

The parameters “*--fa*”, “*--gff*”, and “*--bamdir*” are optional and used to configure tracks in the genome browser. If these parameters are not added, the genome browser panel will not be included in the report. To include the genome browser panel, you must provide at least the “*--fa*” parameter, and the other two parameters can be provided as needed.

The final output includes PAV profiles and interactive web reports (Fig S5). PAV profiles contain all target regions and dispensable regions. In the PAV profile, “1” indicates presence, and “0” indicates absence. The reports are stored in a new folder that contains the PAV report, sample report, JS files, CSS files, and data files.

For the reports, please note the following points:

- You need to move the entire report folder (e.g., “demo1_gene_report/”) to the web server, maintaining the original file structure. Afterward, you can view the report by accessing “PAV.html” and “sample.html”.
- Since BAM files are typically large, we provide two strategies to reduce storage space consumption. Under default settings, soft links will be created for BAM files and their index files to avoid redundant storage. If the “*--slice*” parameter is added, the target regions of interest will be extracted from the BAM files, thereby reducing the size of the BAM files.
- If there are many dispensable regions, the “data.json” file may become excessively large, which could slow down web page loading. In such cases, you can group the results by dividing them by chromosomes or other methods to view the data more efficiently.
- If you wish to download the results for local viewing, you must download the entire folder while preserving its structure. Due to the browser's Cross-Origin Resource Sharing (CORS) policy, the website may not display correctly because the “data.json” file cannot be accessed when you open the HTML file directly. We recommend using the web preview function of Dreamweaver to view the report.

The PAV report allows users to query and filter the PAV table. Users can click on the row for specific regions of interest to examine the coverage distribution for both the presence and absence groups. Additionally, users can view the values in a bar plot across all samples. There is also a sample table report that allows users to filter samples based on phenotypes and PAV results. For the filtered samples, users can examine the new PAV analysis result in real-time within a subpopulation.

The reports of the demo data can be viewed in <https://cgm.sjtu.edu.cn/APAV/demo.html>.

**Examples:**

| $ apav callPAV --cov demo1_gene.cov --pheno demo_sample.pheno --thre 0.5  *## Output: demo1_gene_all.pav, demo1_gene_dispensable.pav, demo1_gene_report/*  $ apav callPAV --cov demo1_gene_ele.cov --pheno demo_sample.pheno --thre 0.5  *## Output: demo1_gene_ele_all.pav, demo1_gene_ele_dispensable.pav, demo1_gene_ele_report/* |
| --- |

Here we show the document tree of the result:

| demo/  ├── demo1_gene_all.pav  ├── demo1_gene_dispensable.pav  ├── demo1_gene_reprot/  │ ├── css/  │ ├── js/  │ ├── data.json  │ ├── PAV.html  │ └── sample.html  ├── demo1_gene_ele_all.pav  ├── demo1_gene_ele_dispensable.pav  └── demo1_gene_ele_reprot/  ├── css/  ├── js/  ├── data.json  ├── PAV.html  └── sample.html |
| --- |

**Examples:**

| $ apav callPAV --cov demo1_gene.cov --pheno demo_sample.pheno --thre 0.5 \  --fa demo.fa.gz --gff demo1_gene.gff3 --bamdir bam  *## Output: demo1_gene_all.pav, demo1_gene_dispensable.pav, demo1_gene_report/* |
| --- |

Here we show the tree of the report document:

| demo1_gene_reprot  ├── browser/  │ ├── reference.fa.gz  │ ├── reference.fa.gz.fai  │ ├── reference.fa.gz.gzi  │ ├── reference.gff.gz  │ ├── reference.gff.gz.tbi  │ ├── target.bb  │ ├── sample01.bam  │ ├── sample01.bam.bai  │ └── ...  ├── css/  ├── js/  ├── data.json  ├── PAV.html  └── sample.html |
| --- |

After obtaining the PAV (Presence-Absence Variation) results for genes, you can further calculate the PAV for gene families. If at least one gene within a gene family is present, the gene family is marked as present; otherwise, it is marked as absent.

**Examples:**

| $ apav gFamPAV --pav demo1_gene_all.pav --fam demo1_gene.fam  *## Output: demo1_gene_all.gfpav* |
| --- |

1. **Genome estimation**

The “*pavSize*” command is used to estimate the sizes of both the pangenome and core genome derived from the PAV table. The “*-n*” parameter sets the number of estimations. The estimation can be performed for all groups after adding the “*--group*” option.

**Examples:**

| $ apav pavSize --pav demo1_gene_all.pav  *## Output: demo1_gene_all.size*  *## Estimation in groups*  $ cat demo_sample.pheno \| cut -f 1,2 > demo_sample.group  $ apav pavSize --pav demo1_gene_all.pav --group demo_sample.group --out demo1_gene_all_group.size  *## Output: demo1_gene_all_group.size* |
| --- |

The “*pavPlotSize*” command utilizes the output table from “*pavSize*” to generate a growth curve illustrating the estimation of genome size. It calls R scripts and generates a PDF file. The “*--data_type*” option provides an identity count of the pangenome and core genome, or an increase of the pangenome. It offers three chart types: “errorbar”, “jitter” and “ribbon” set by the “*--chart_type*” option. The usage information and parameter list can be obtained with “*apav pavPlotSize --help*” or “*apav pavPlotSize -h*”.

**Examples:**

| $ apav pavPlotSize --size demo1_gene_all.size  $ apav pavPlotSize --size demo1_gene_all.size \  --path_color '#e38e28,#298022' --ribbon_fill '#e38e28,#298022' --ribbon_alpha 0.2 |
| --- |


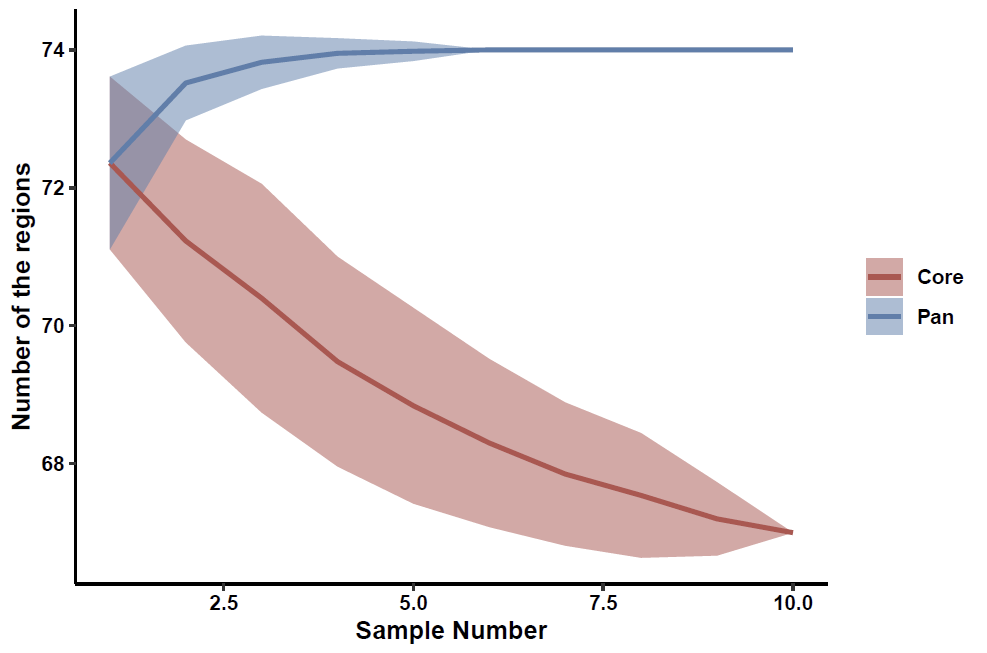

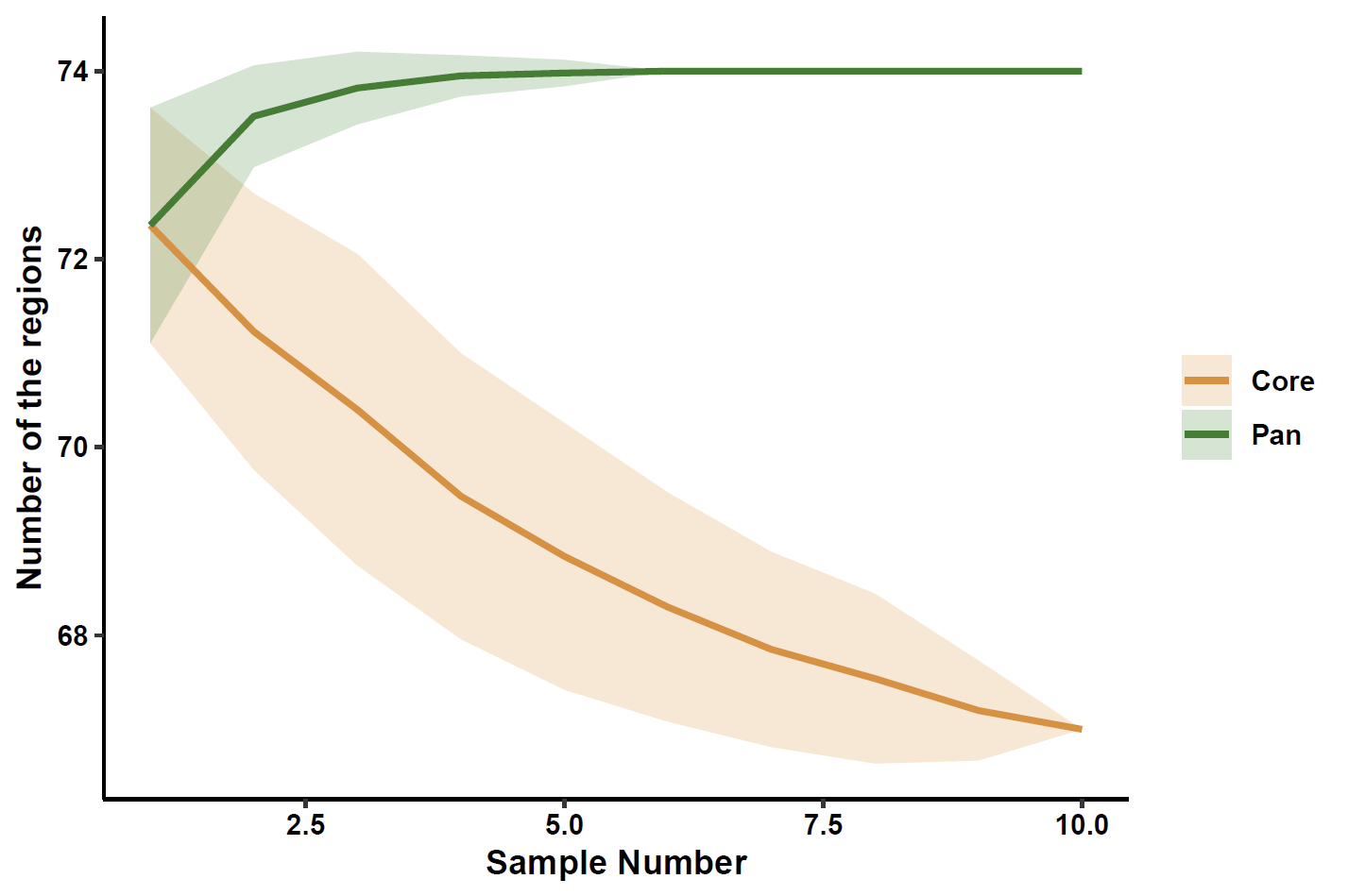


| $ apav pavPlotSize --size demo1_gene_all.size \  --data_type increasing \  --chart_type errorbar |
| --- |


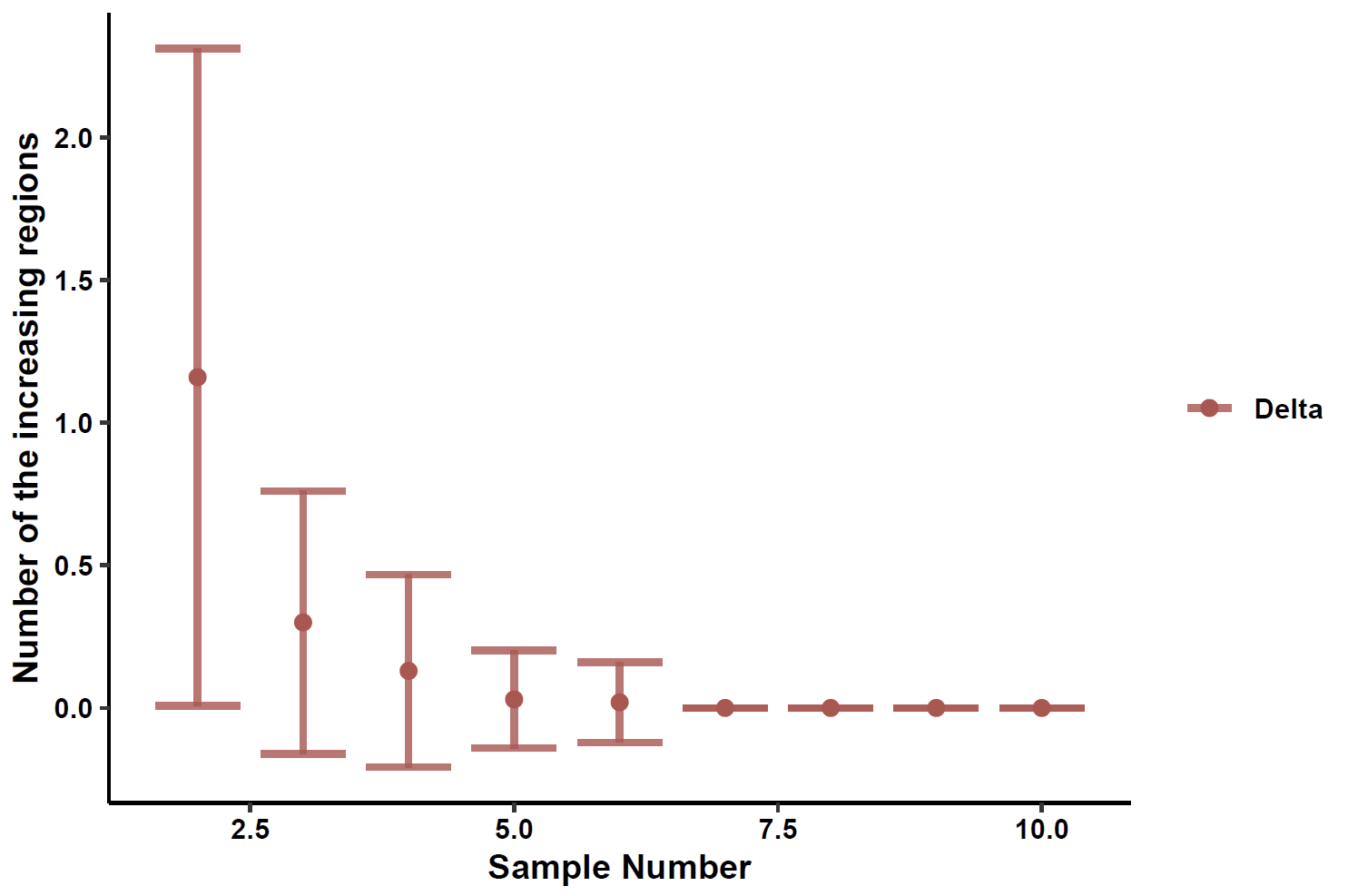


| $ apav pavPlotSize --size demo1_gene_all_group.size |
| --- |


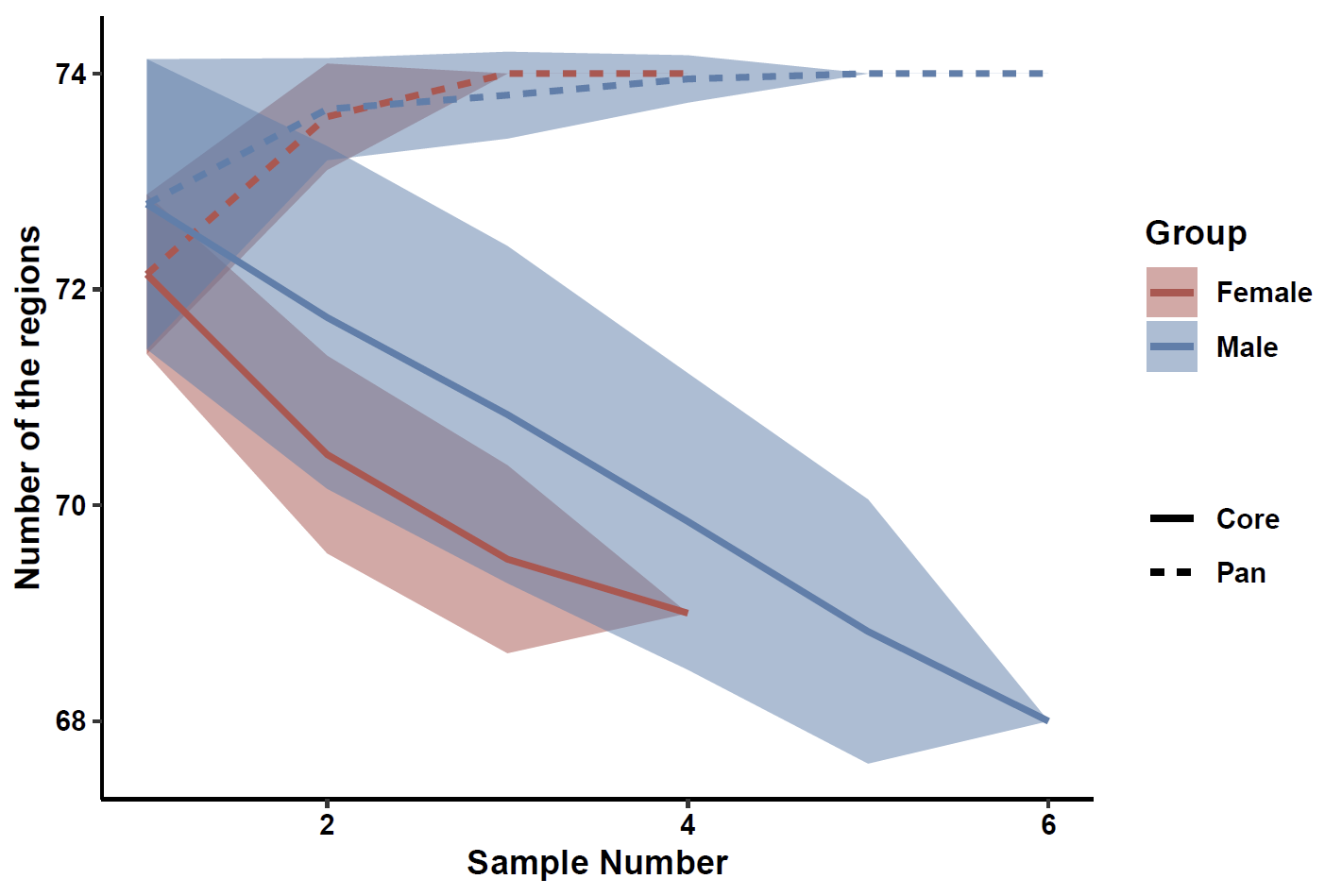


1. **PAV analysis and visualization**

The target regions are categorized into core regions (present in all individuals) and dispensable regions (not present in all individuals). The dispensable regions can be further divided into softcore regions, distributed regions and private regions. Regions with loss rates that do not exceed the “softcore_loss_rate” are classified as “softcore regions”. Regions that are found in only one sample are labeled as “private regions”, while all other regions are referred to as “distributed regions”.

APAV offers a variety of visualization functions. The “*pavPlotStat*” command generates a half-violin plot to show the number of target regions present in samples. The “*pavPlotHist*” command combines a ring chart and a histogram to display the distribution of target regions. The “*pavPlotHeat*” command creates a heatmap along with two summary annotations. The “*pavPlotBar*” command allows users to view the composition of genes across all samples. The “*pavPCA*” command performs PCA analysis for PAV data, and the “*pavCluster*” command clusters samples based on the PAV table. The usage information and parameter list for each command can be shown with the “*--help*” or “*-h*” option after each command name.

**Examples:**

| $ apav pavPlotStat --pav demo1_gene_all.pav --fig_width 4 --fig_height 3  $ apav pavPlotStat --pav demo1_gene_all.pav \  --pheno demo_sample.pheno --add_pheno_info Location \  --pheno_info_colors '#e38e28,#298022' \  --fig_width 4 --fig_height 3 |
| --- |


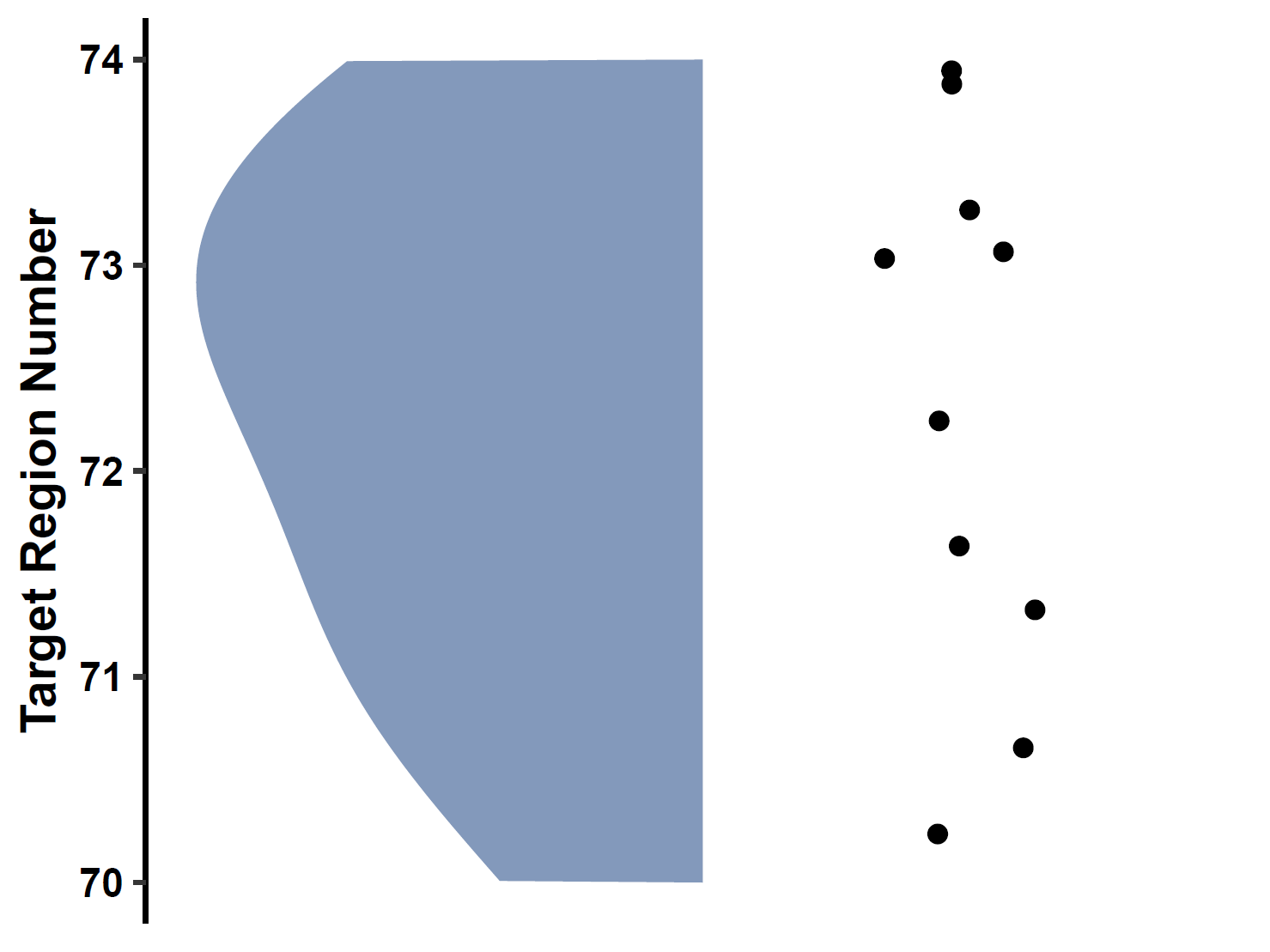

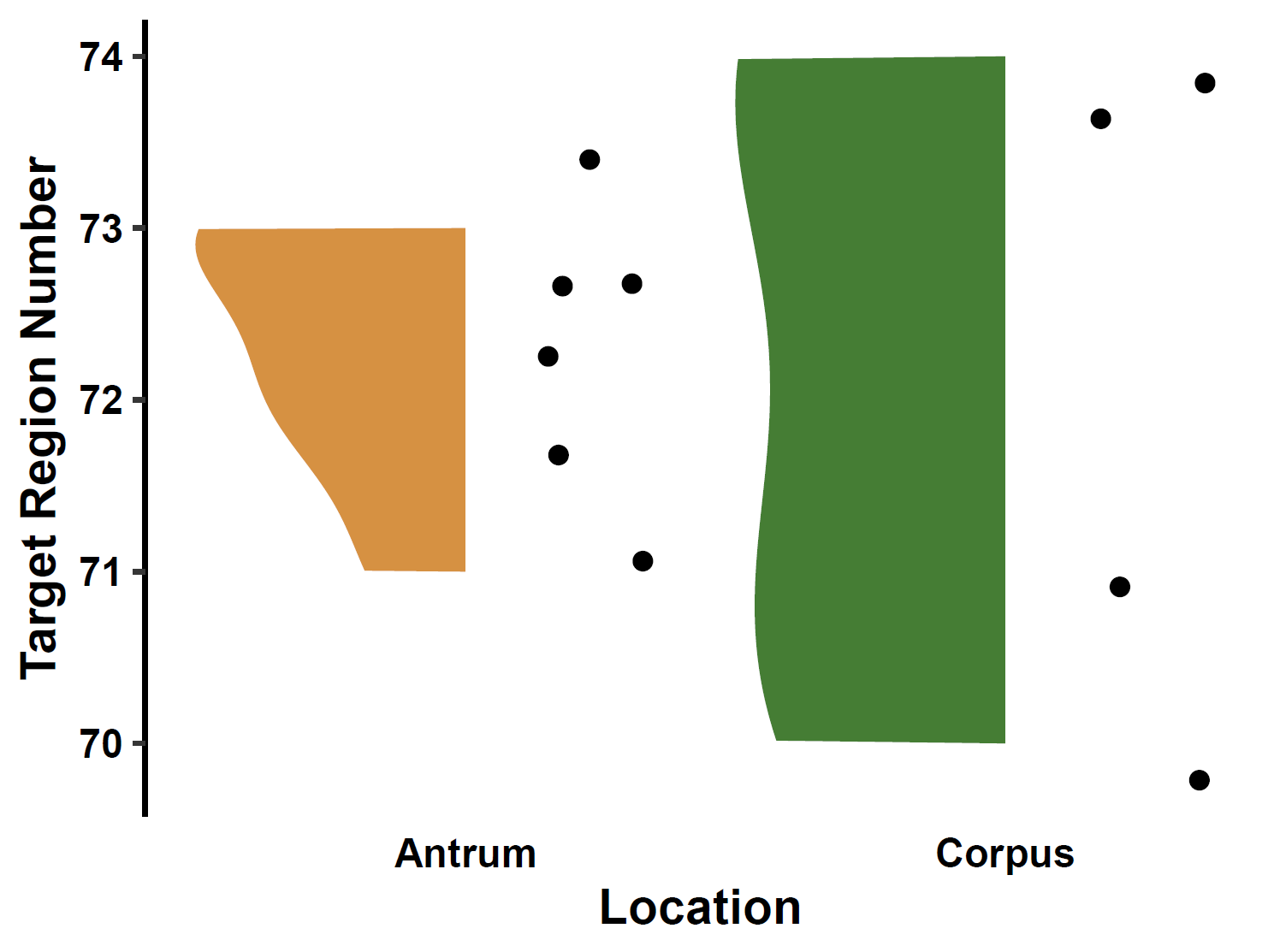


| $ apav pavPlotHist --pav demo1_gene_all.pav --ring_pos_x 0.3 --ring_r 0.4 |
| --- |


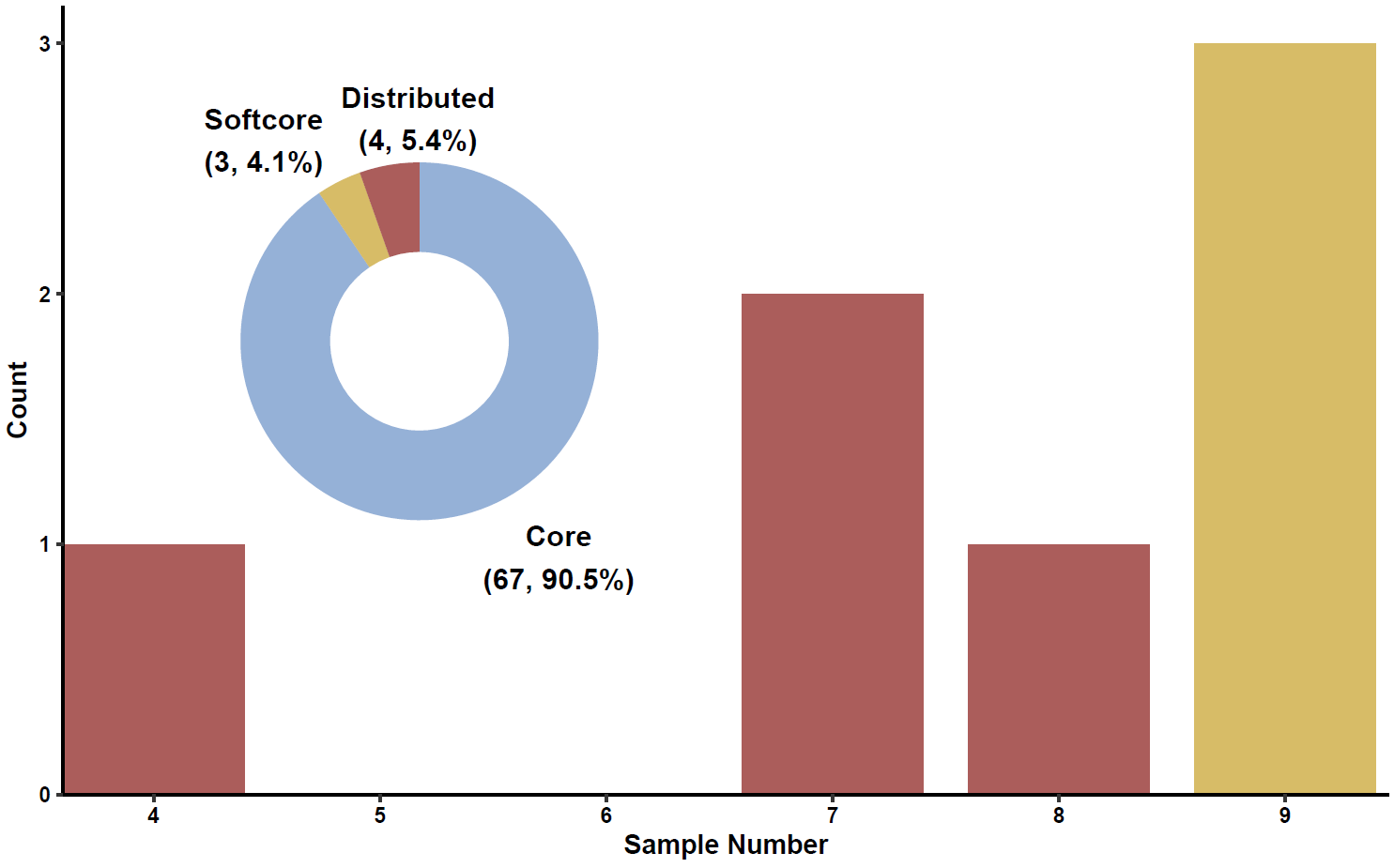


| *## Default palette*  $ apav pavPlotHeat --pav demo1_gene_all.pav --pheno demo_sample.pheno \  --block_name_rot 90 --fig_height 6 --fig_width 8  *## Custom colors*  $ apav pavPlotHeat --pav demo1_gene_all.pav --pheno demo_sample.pheno \  --block_name_rot 90 --fig_height 6 --fig_width 8 \  --pav_colors '#D98D8D,white' \  --type_colors '#A4C9E3,#6CA6CD,#3E759C' \  --pheno_info_color_list Gender=#e38e28,#E8D151 |
| --- |


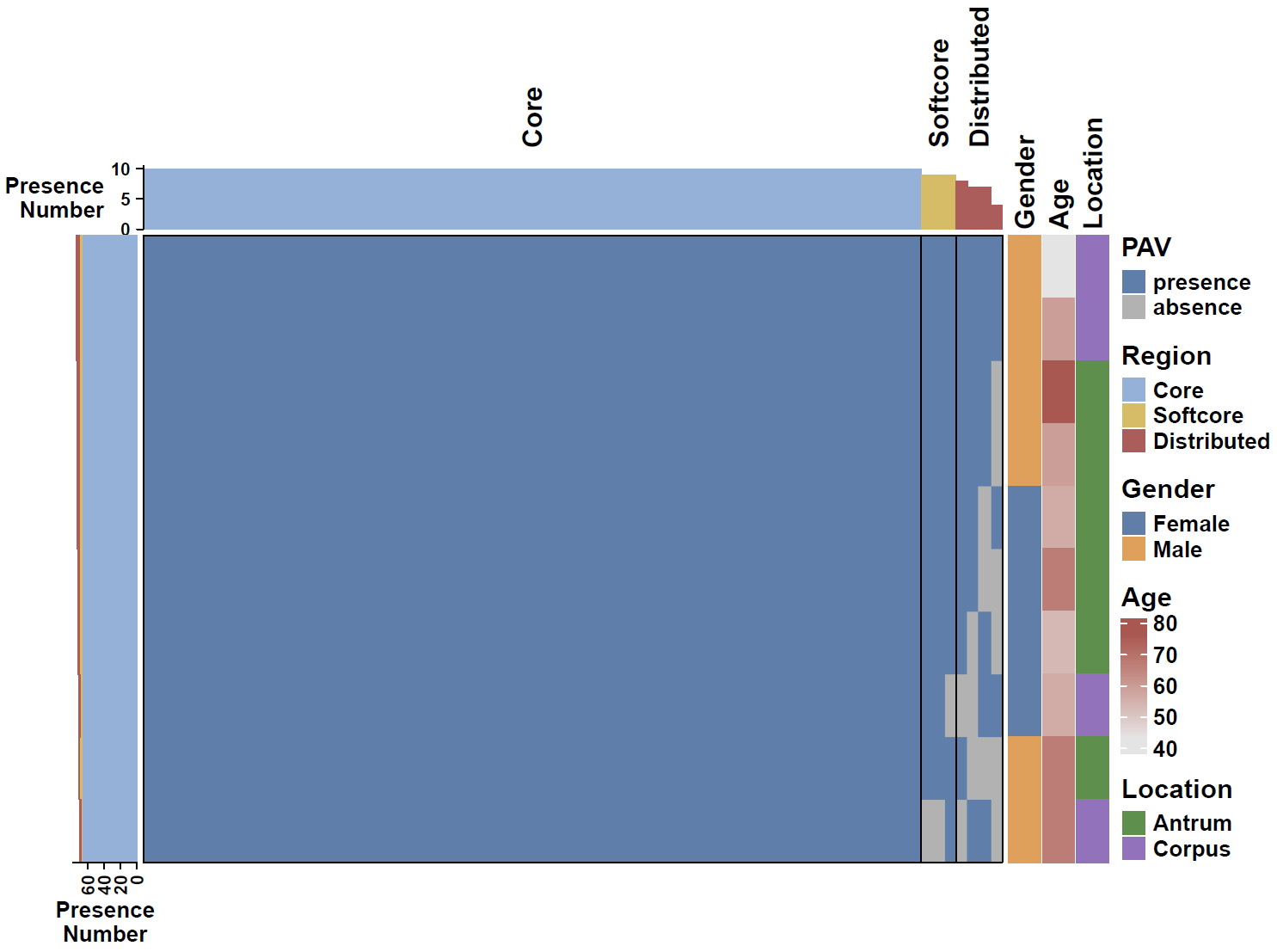

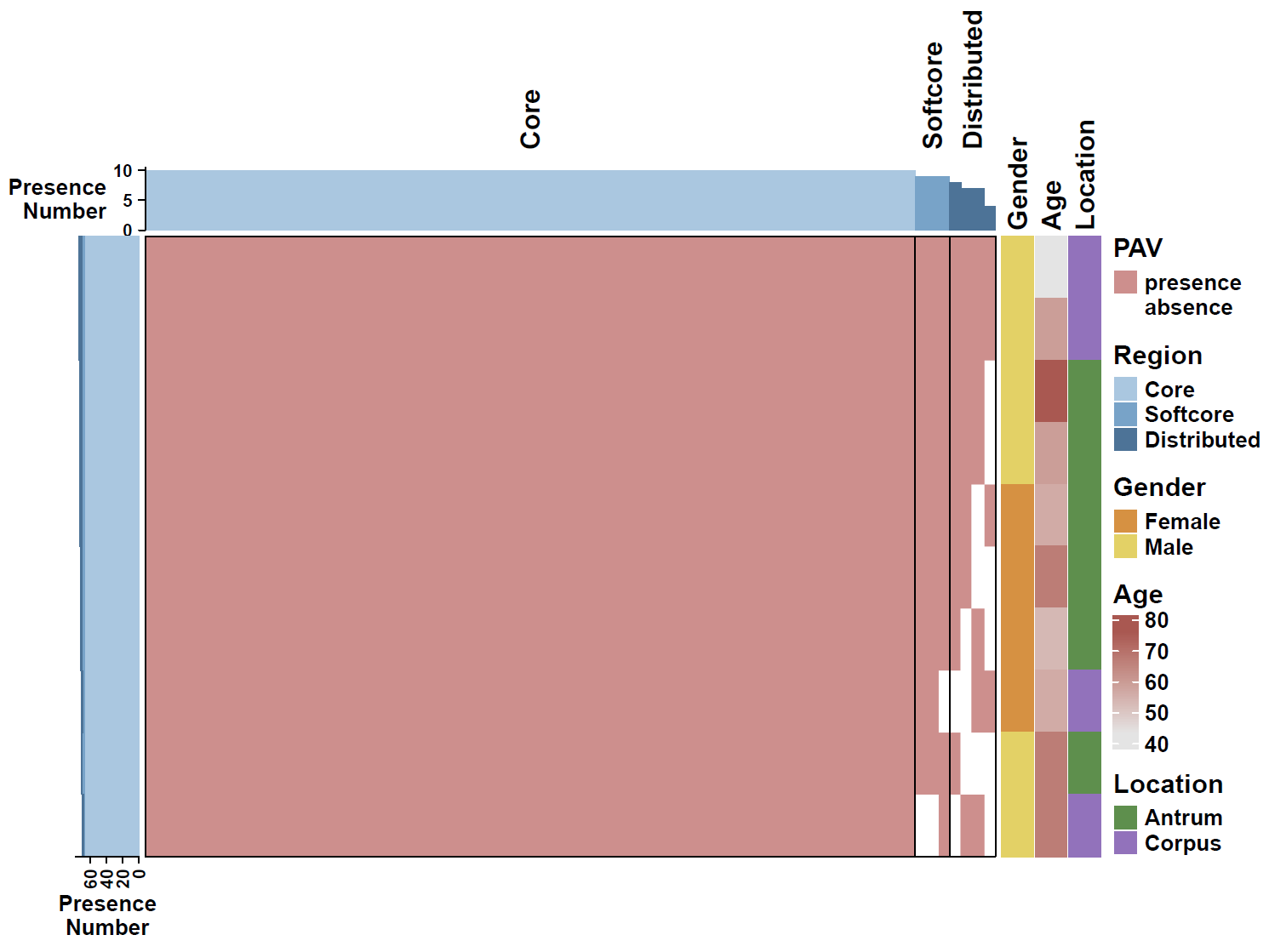


| $ apav pavPlotBar --pav demo1_gene_all.pav \  --pheno demo_sample.pheno --add_pheno_info Gender \  --fig_height 6 --fig_width 8 |
| --- |


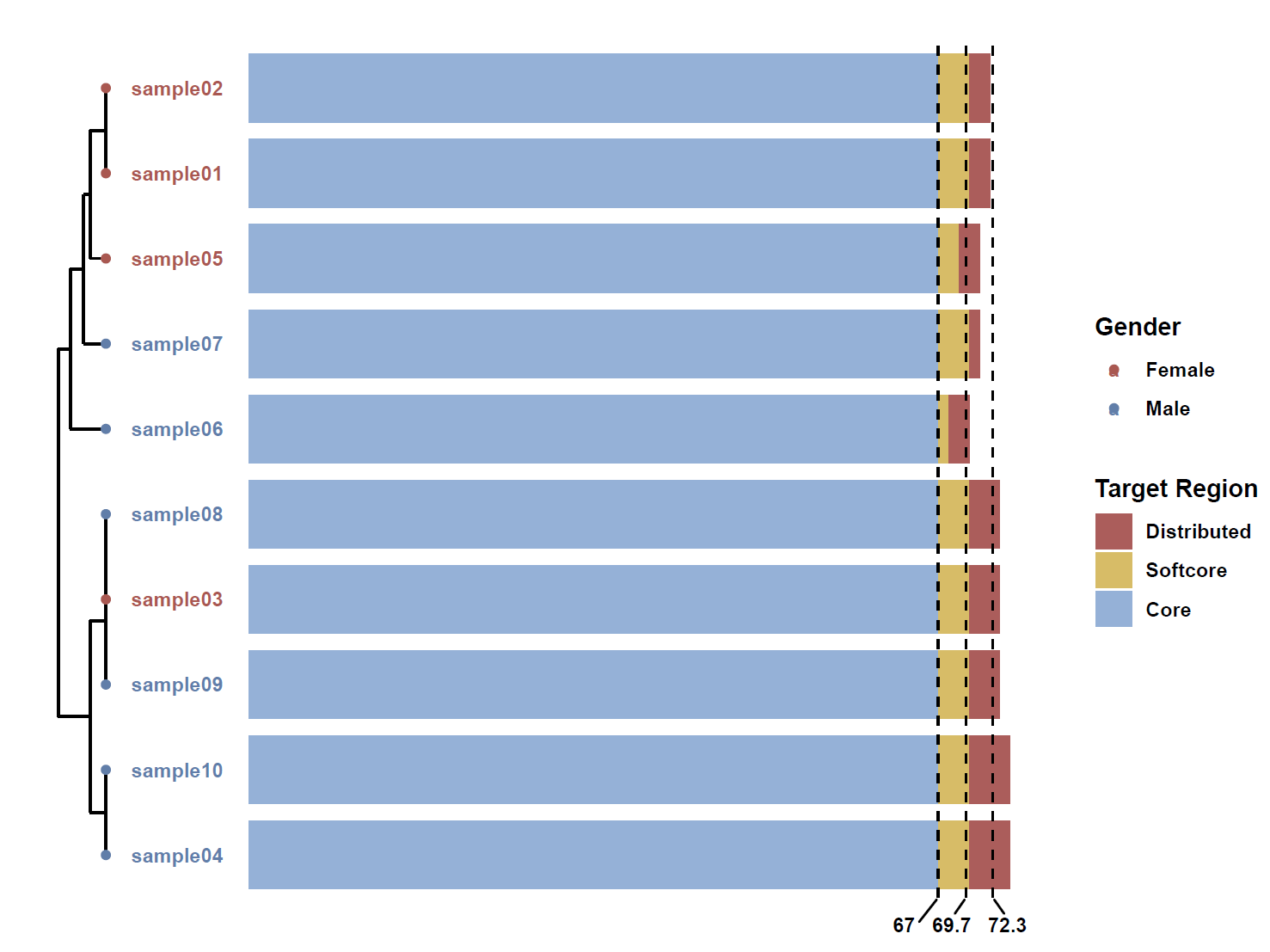


| $ apav pavPCA --pav demo1_gene_dispensable.pav \  --pheno demo_sample.pheno --add_pheno_info Gender \  --fig_height 4 --fig_width 6 |
| --- |


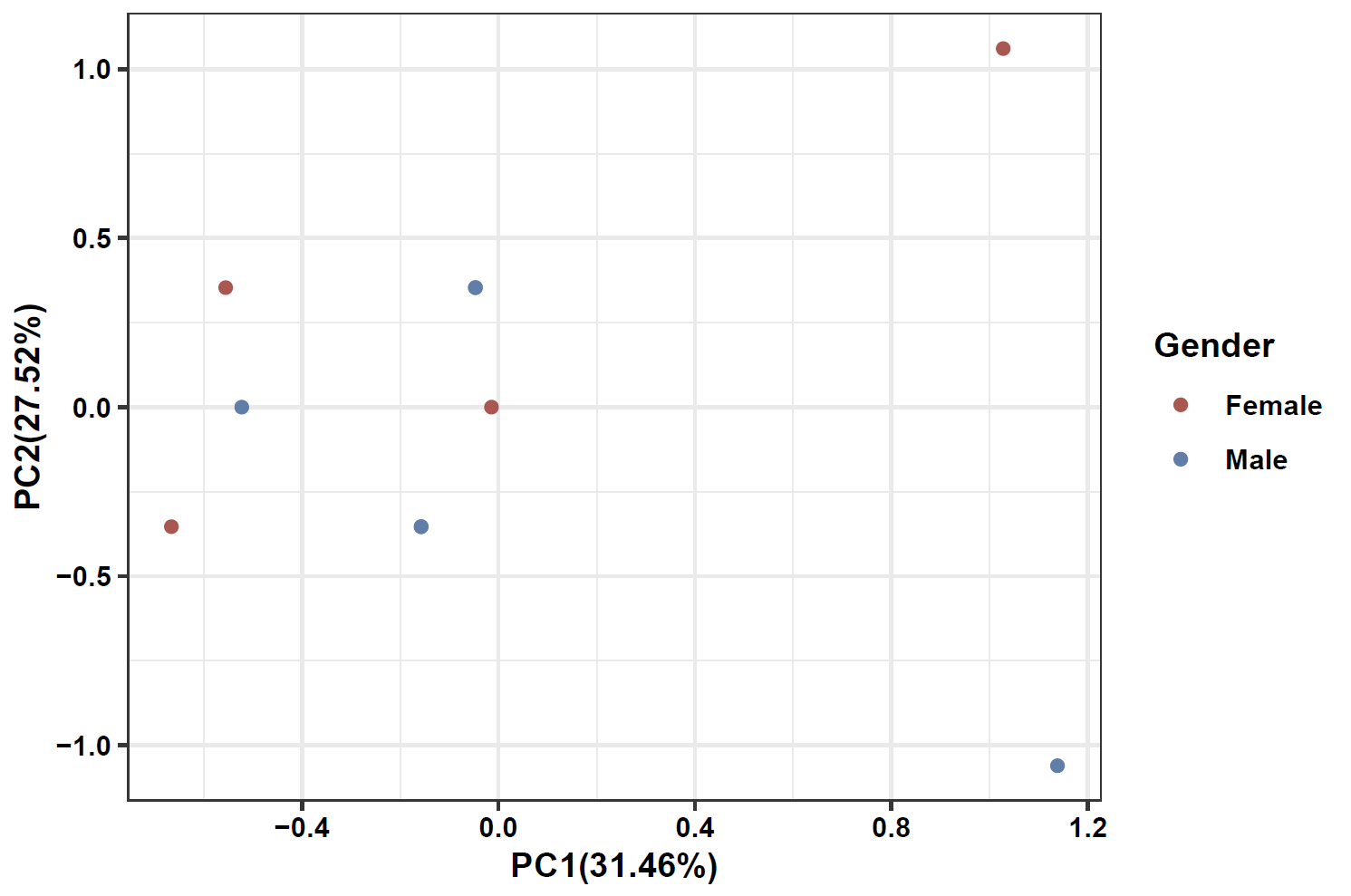


| $ apav pavCluster --pav demo1_gene_dispensable.pav \  --pheno demo_sample.pheno --add_pheno_info Gender \  --pheno_info_colors '#e38e28,#298022' --fig_height 6 --fig_width 8 |
| --- |


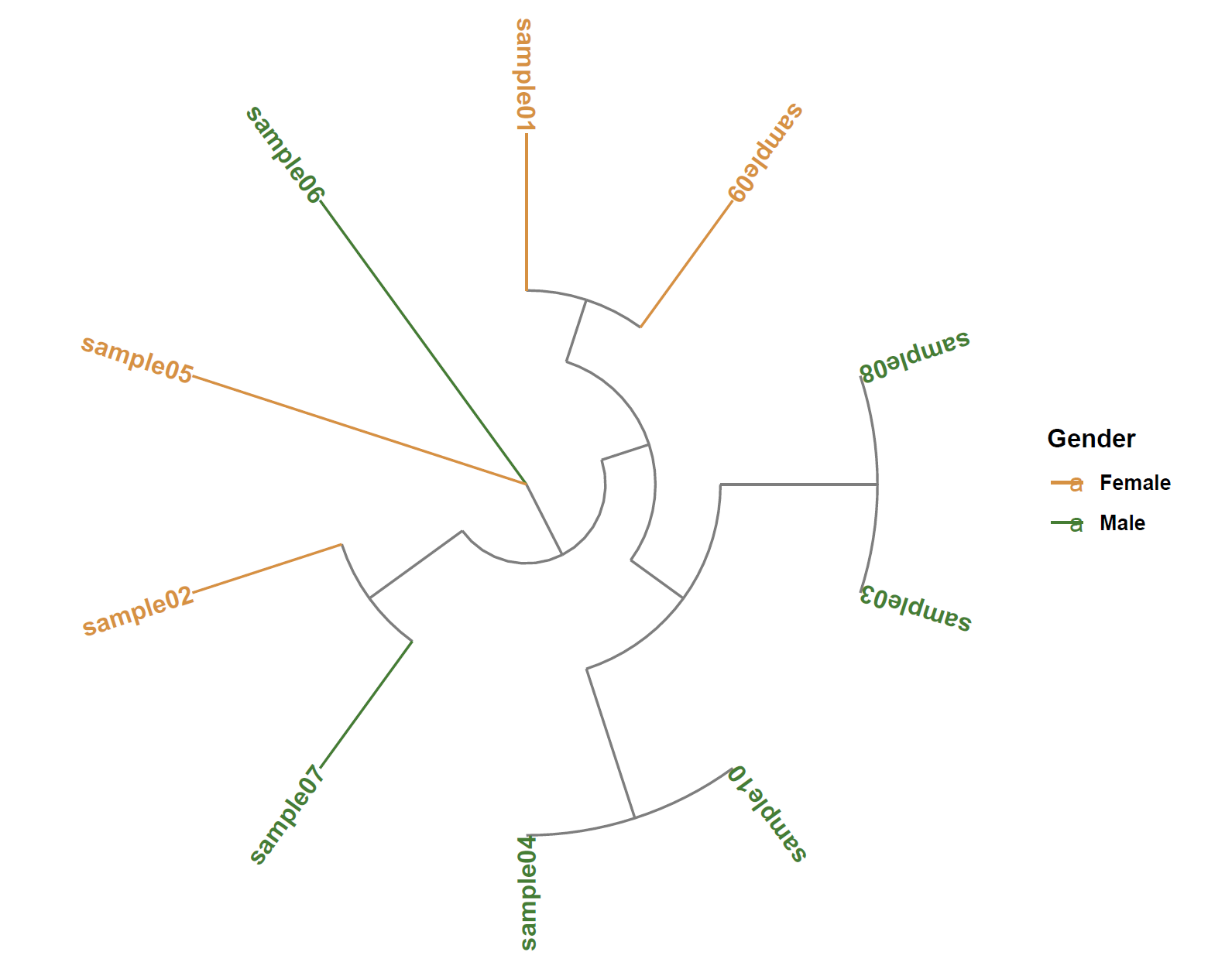


1. **Phenotype association analysis**

Phenotype association can help researchers understand the potential biological functions of PAVs. The “*pavStaPheno*” command performs phenotype association analysis of dispensable target regions. Additionally, the results can be displayed using commands like “*pavPlotPhenoHeat*”, “*pavPlotPhenoBlock*”, “*pavPlotPhenoMan*”, “*pavPlotPhenoBar*”, and “*pavPlotPhenoVio*”. These commands require R to be installed. The usage information and parameter list for each command can be shown with the “*--help*” or “*-h*” option after each command name.

Since the demo data is too small to identify valid correlation results, the figures below are intended solely for visualization purposes.

**Examples:**

| $ apav pavStaPheno --pav demo1_gene_dispensable.pav --pheno demo_sample.pheno  *## Output: demo1_gene_dispensable.phenores* |
| --- |

| $ apav pavPlotPhenoHeat --pav demo1_gene_dispensable.pav \  --pheno_res demo1_gene_dispensable.phenores --p_threshold 0.5 \  --only_show_significant \  --fig_height 4 --fig_width 6 |
| --- |


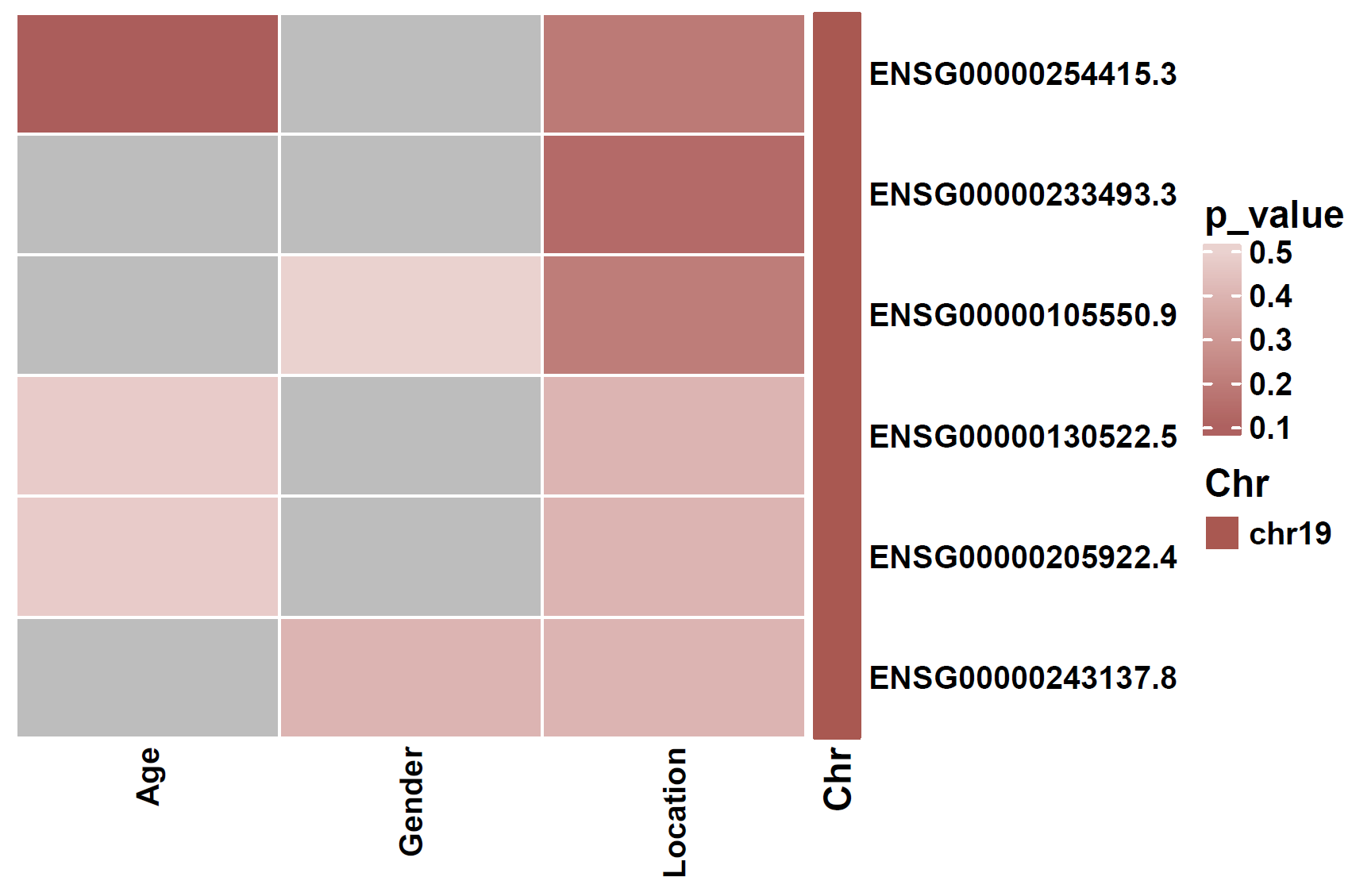


| $ apav pavPlotPhenoBlock --pav demo1_gene_dispensable.pav \  --pheno demo_sample.pheno --pheno_res demo1_gene_dispensable.phenores \  --pheno_name Gender --p_threshold 1 \  --fig_height 4 --fig_width 6 |
| --- |


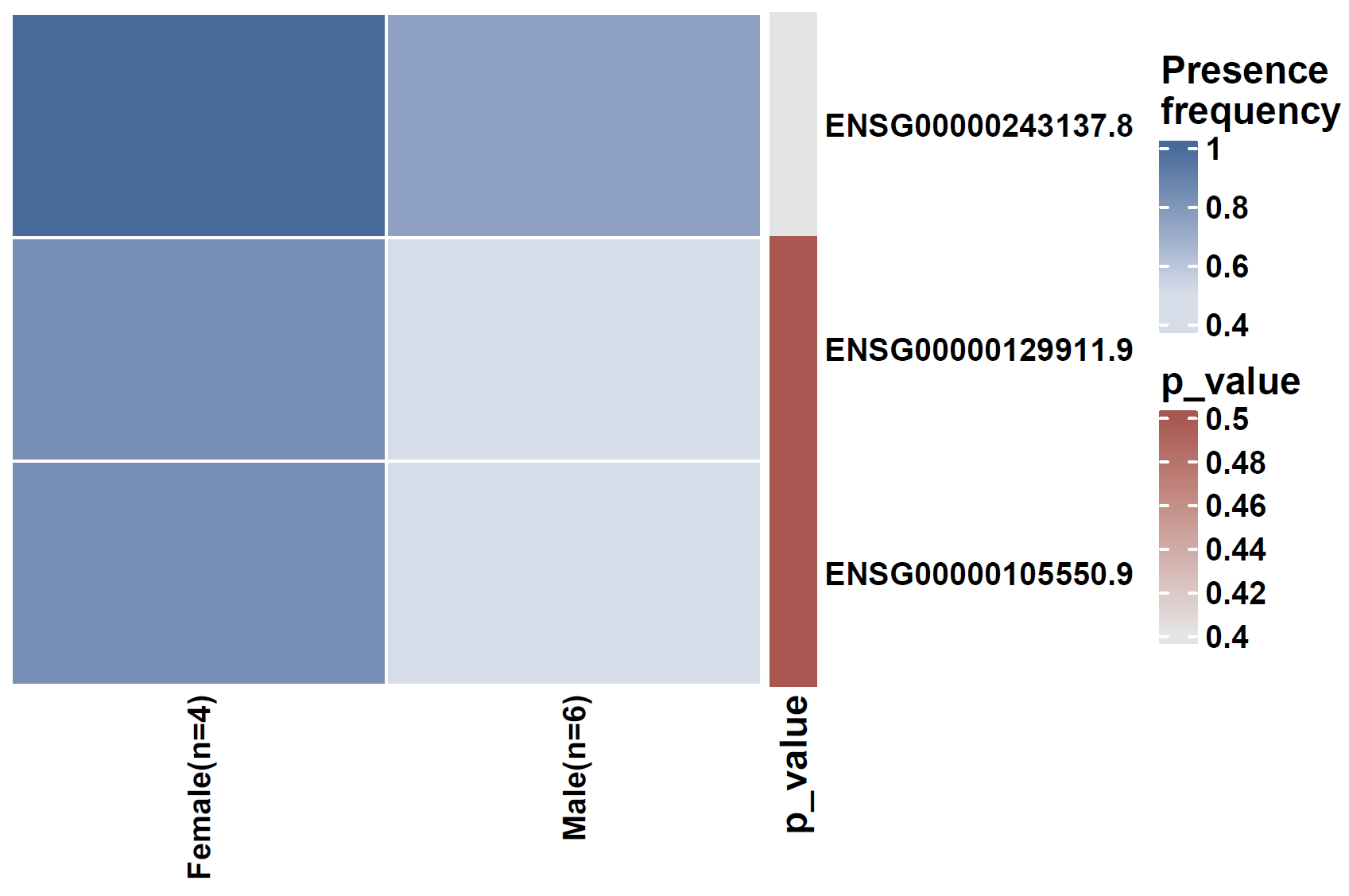


| $ apav pavPlotPhenoMan --pav demo1_gene_dispensable.pav \  --phen demo_sample.pheno --pheno_res demo1_gene_dispensable.phenores \  --pheno_name Gender --highlight_top_n 3 \  --fig_height 4 --fig_width 6 |
| --- |


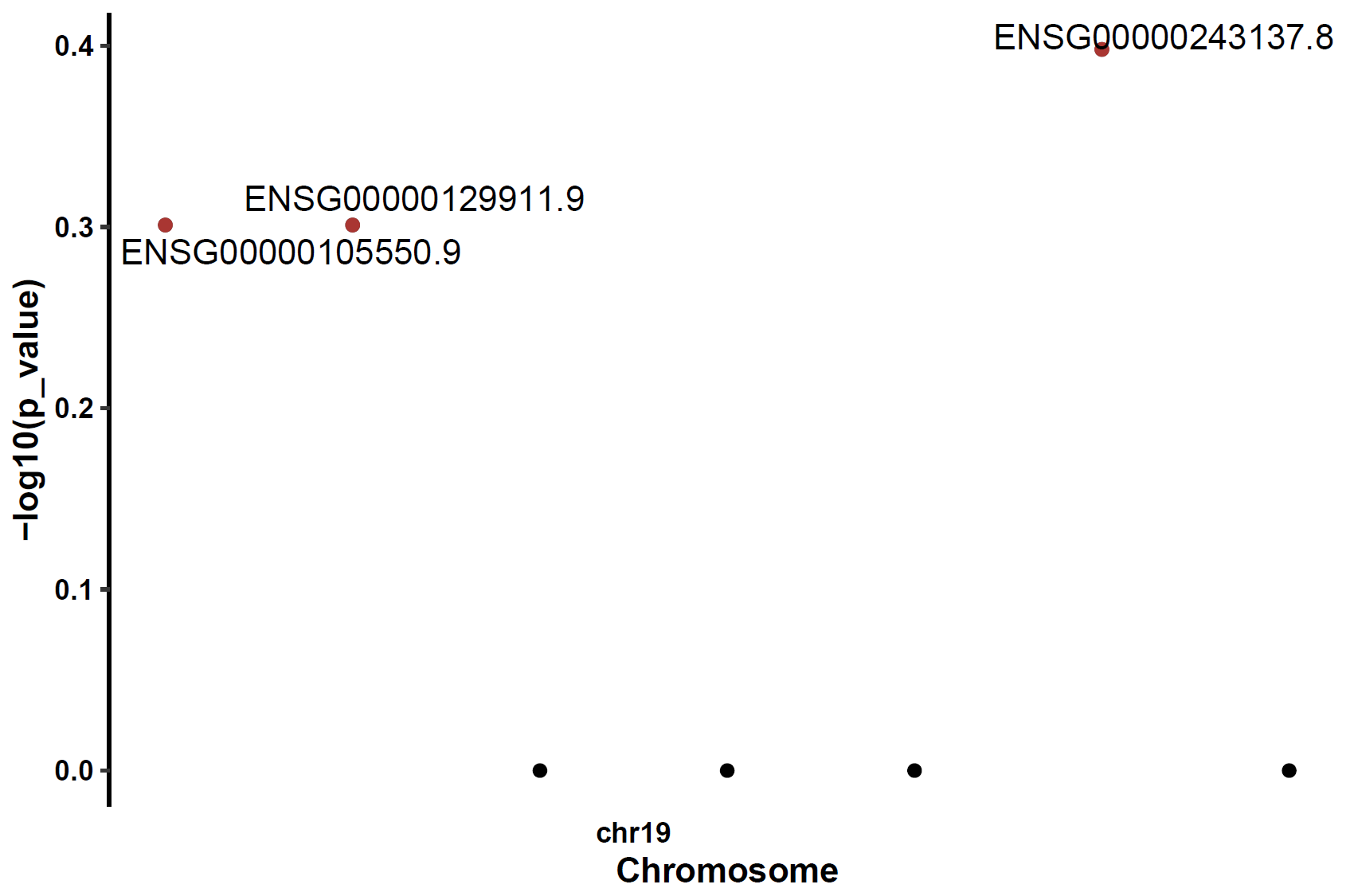


| $ apav pavPlotPhenoBar --pav demo1_gene_all.pav --pheno demo_sample.pheno \  --pheno_name Location --region_name ENSG00000233493.3 \  --fig_height 4 --fig_width 4 |
| --- |


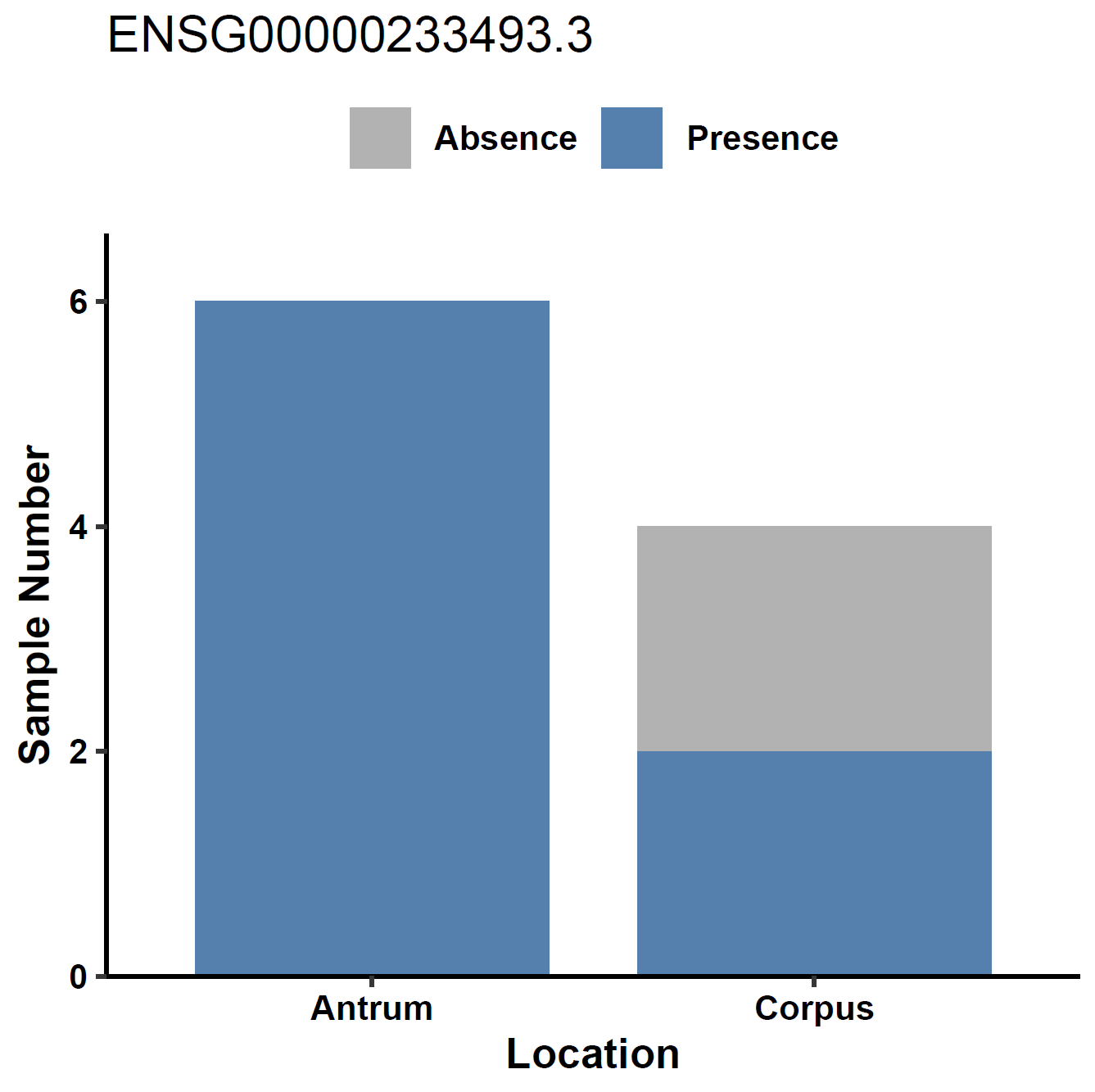


| $ apav pavPlotPhenoVio --pav demo1_gene_all.pav --pheno demo_sample.pheno \  --pheno_name Age --region_name ENSG00000254415.3 \  --fig_height 4 --fig_width 4 |
| --- |


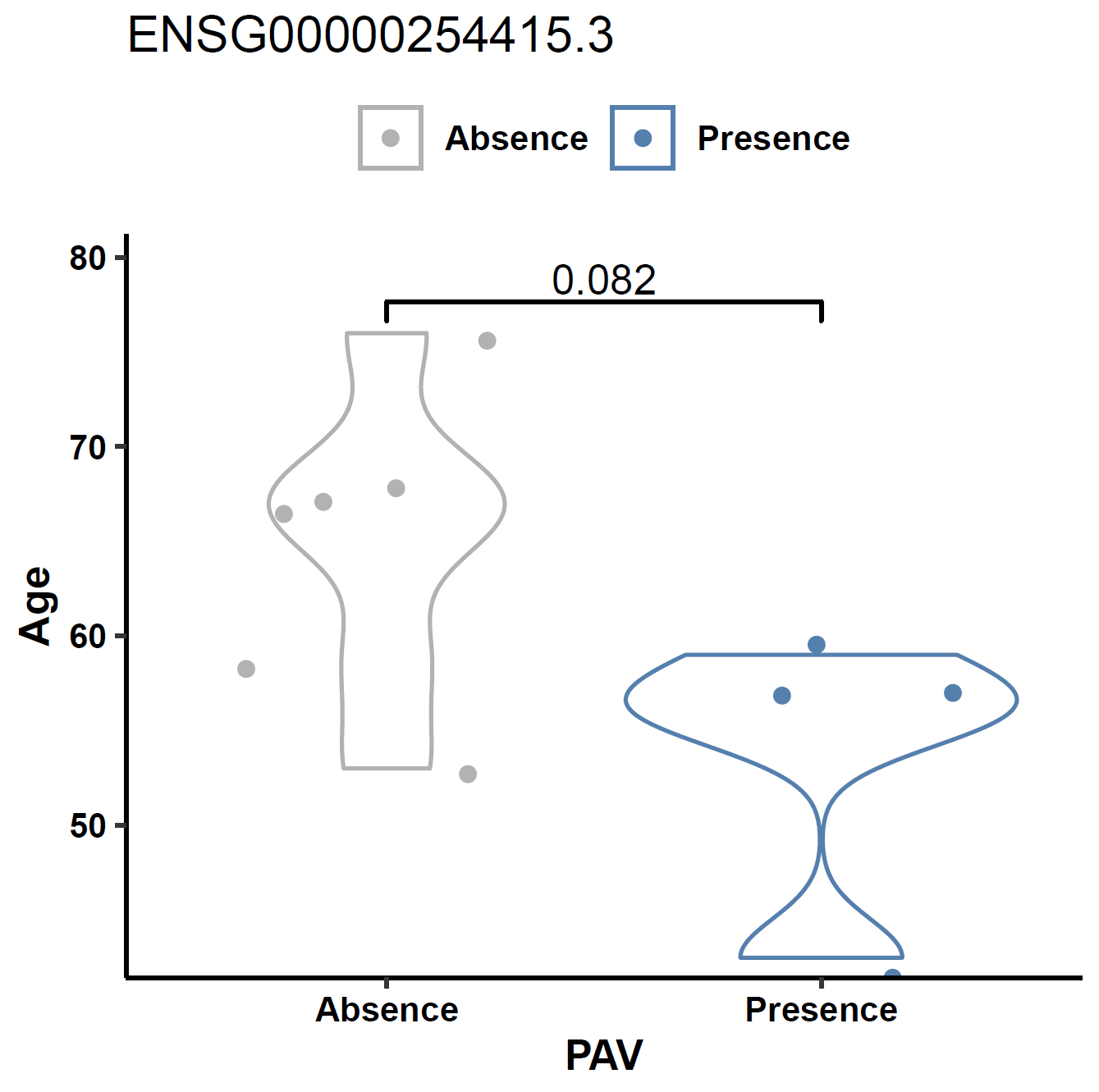


1. **Visualization of elements data**

For the region of interest, you can use the “*elePlotCov*” and “*elePlotPAV*” commands to visualize the coverage and PAV of elements across all samples. The sequence alignment depth for all samples can be examined using the “*elePlotDepth*” command. These commands require R to be installed. The usage information and parameter list for each command can be shown with the “*--help*” or “*-h*” option after each command name.

**“***elePlotCov***” command:**

This command displays coverage for all elements in a specified target region in a composite graph. The graph is divided into two sections: the upper section features bar squares representing the elements, while the lower section contains a heatmap illustrating the coverage. Lines connect these two sections. Each row in the heatmap corresponds to a sample, and the columns represent the elements. You can customize the color palette for coverage using the “--cov_colors” parameter. The color of the element blocks can be adjusted with the “*--ele_color*” parameter, and the color of the lines can be set using the “*--ele_line_color*” parameter. To cluster the samples, use the “*--cluster_samples*” parameter.

The “*--pheno*” option allows you to include phenotype information in the sample display, with the phenotype color determined by the “--pheno_info_color_list” parameter. If you wish to display the gene structure for the corresponding region, use the “*--gff*” parameter, and you can modify the genetic element colors with the “*--gene_colors*” parameter.

**“***elePlotPAV***” command:**

This command replaces input coverage data with PAV data. The parameter settings and usage are identical to those of the “*elePlotCov*” command.

**“***elePlotDepth***” command:**

The parameter settings and usage for this command are similar to those of the previous two commands. The primary distinction is that the lower part of the image features a density map, which displays depth information corresponding to the coordinates above. To calculate the depth of the locus in the target region, you must add the “*--bamdir*” option. The “*--depth_colors*” option sets the color palette for depth, while the “*--lowlight_colors*” parameter can be used to darken non-elemental regions.

**Examples:**

| $ grep 'ENSG00000126251.6' demo1_gene.gff3 > example.gff3  $ grep -E 'Annotation\|ENSG00000126251.6' demo1_gene_ele.cov > example.elecov  $ grep -E 'Annotation\|ENSG00000126251.6' demo1_gene_ele_all.pav > example.elepav  $ apav elePlotCov --elecov example.elecov --pheno demo_sample.pheno --gff example.gff3  $ apav elePlotPAV --elepav example.elepav --pheno demo_sample.pheno --gff example.gff3  $ apav elePlotDepth --ele example.elecov --bamdir bam \  --pheno demo_sample.pheno --gff example.gff3 |
| --- |


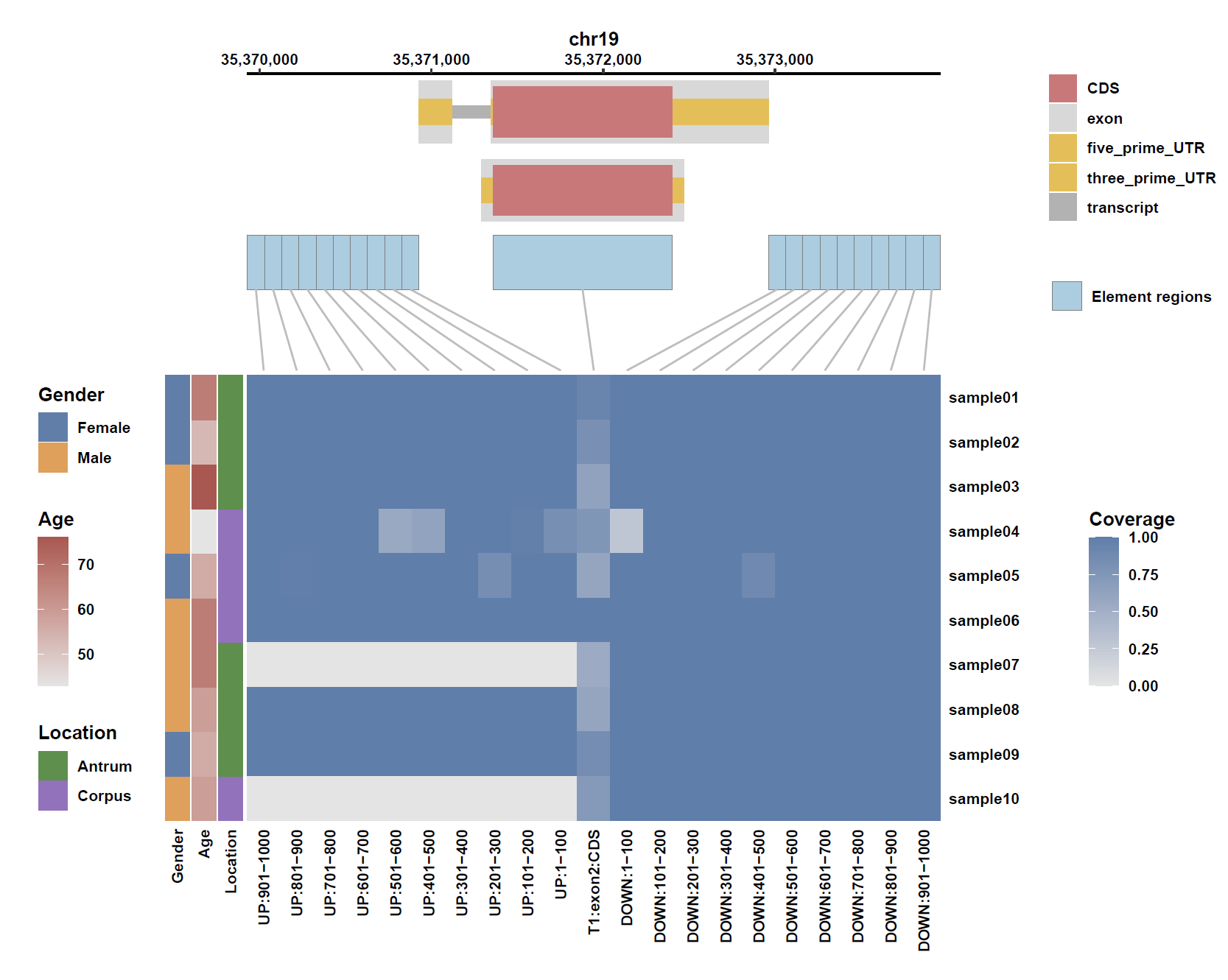

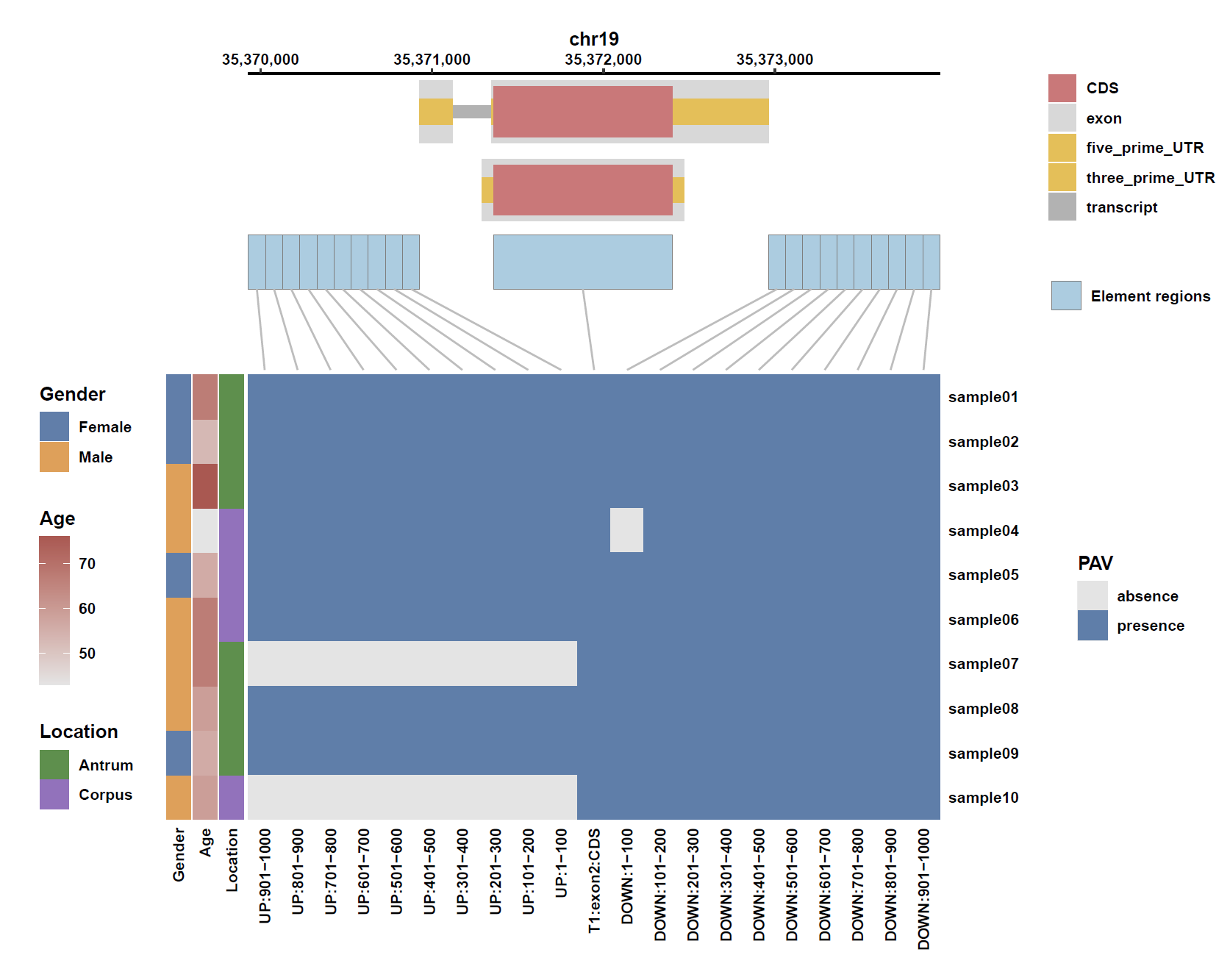


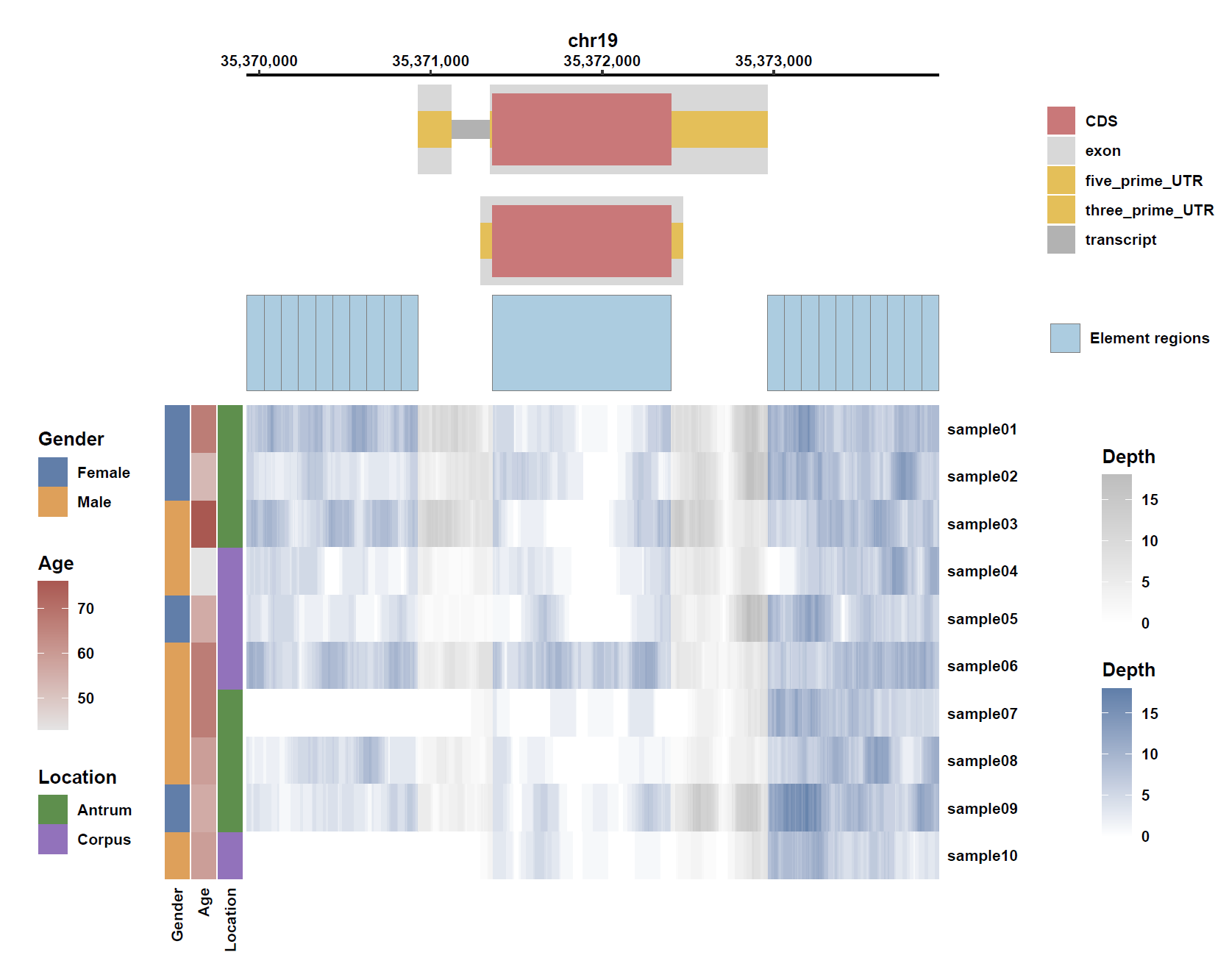


You can batch perform the above steps on genes or other target regions.

**region.list**

| ENSG00000205922.4  ENSG00000129911.9  ENSG00000130522.5 |
| --- |

**plot_ele.sh**

| #!/bin/bash  array=()  while read line; do  array+=(“$line”)  done < region.list  for gene in ${array[@]}  do  grep ${gene} demo1_gene.gff3 > ${gene}.gff  *## element coverage*  grep -E “Annotation\|${gene}” demo1_gene_ele.cov > ${gene}.elecov  apav elePlotCov --elecov ${gene}.elecov --pheno demo_sample.pheno --gff ${gene}.gff  *## element PAV*  grep -E “Annotation\|${gene}” demo1_gene_ele_all.pav > ${gene}.elepav  apav elePlotPAV --elepav ${gene}.elepav --pheno demo_sample.pheno --gff ${gene}.gff  *## element depth*  apav elePlotDepth --ele ${gene}.elecov --bamdir bam \  --pheno demo_sample.pheno --gff ${gene}.gff  done |
| --- |
