# Supplementary material for: APAV: An advanced pangenome analysis and visualization toolkit
Source: PLoS Comput Biol. 2025 Jul 7;21(7):e1013288. doi: 10.1371/journal.pcbi.1013288 (PMC12251200; doi:10.1371/journal.pcbi.1013288)

**S2 Text. Supporting information for “APAV: An advanced pangenome analysis and visualization toolkit”**

**Usage Guide of APAVplot**

For a better plotting experience, we highly recommend APAVplot, an R package specifically designed for visualization of PAV analysis. It provides a greater range of parameters than the visualization command in APAV. It's more efficient to adjust chart details and preview results in the R environment. APAVplot provides the vignette for a long-form guide. More details and code are available at <https://github.com/SJTU-CGM/APAVplot>.

This package includes the following functions:

Coverage Visualization:

- To begin, you need to construct a COV class using the get_cov_obj() function.
- The cov_heatmap() function generates a heatmap that provides a visual overview of the coverage profile across samples.
- The cov_density() function visualizes the coverage distribution of the regions of interest.

PAV statistics and analysis :

- First, you need to create a PAV class using the get_pav_obj() function.
- The pav_heatmap() function produces a sophisticated heatmap offering an overview of the PAV profile. The pav_hist() function generates both a ring chart and a histogram to illustrate the classifications and distribution of target regions.
- The pav_halfviolin() function creates a half-violin chart to showcase the number of regions in each sample group.
- The pav_stackbar() function plots a stacked bar chart that displays the classifications of target regions across all samples.
- The pav_cluster() function clusters samples based on the PAV table and visualizes the clustering results. The pav_pca() function performs PCA analysis on the PAV table and generates corresponding visual representations of the results.

Phenotype association and visualization :

- The pheno_stat() function executes Fisher’s exact test and Wilcoxon test to explore phenotype associations.
- The pheno_heatmap() function creates a heatmap visualizing the primary outcomes of phenotype association analysis.
- The pheno_manhattan() function produces a Manhattan plot to display the results of a specified phenotype. The pheno_block() function generates a block chart that displays the percentage of samples within each group of a discrete phenotype.
- The pheno_bar() function provides a bar plot reflecting PAV labels within each phenotype grouping.
- The pheno_violin() function plots a violin plot that displays the phenotype values for both presence and absence samples.

Drawing growth curves :

- The input is the output table from the APAV “*pavSize*” tool.
- The plot_size() function draws growth curves for genome estimation.

Visualization of elements :

- The plot_ele_cov() function visualizes the coverage of elements within a specified target region.
- The plot_ele_pav() function visualizes the PAV of elements within a specified target region.
- The plot_ele_depth() function visualizes the depth of elements within a specified target region.

**1 Input data**

**1.1 COV class**

The get_cov_obj() function generates an object of the COV class. It requires a numeric matrix or a data.frame containing coverage data, which indicates the percentage of the target region covered by reads. Each row corresponds to a target region, while the columns represent samples. Additionally, the parameters pheno_info and region_info are optional. The pheno_info should be a data.frame that includes phenotype data and any other attributes related to the samples. The region_info should be a data.frame containing information about the target regions, such as whether they are reference or novel, along with their corresponding chromosomes and positions.

**1.2 PAV class**

The get_cov_obj() function is used to generate an object of the PAV class. It requires a numeric PAV table as input, where a numeric value of 0 indicates absence and 1 indicates presence. The row names correspond to the target regions, while the column names represent the sample names. The parameters pheno_info and region_info are consistent with those in the COV class.

The parameters add_softcore and add_private are boolean values that indicate whether to include “softcore” or “private” in classifying the target region.

- If add_softcore is set to TRUE, regions with loss rates that are not significantly higher than softcore_loss_rate will be classified as softcore regions. If use_binomial is also set to TRUE, a binomial test will be conducted for each target region, with the null hypothesis stating that the loss rate is less than softcore_loss_rate. A p-value below softcore_p_value indicates that the target region has a significantly higher loss rate and is classified as a distributed region that exceeds softcore_loss_rate.
- If add_private is set to TRUE, regions that are present in only one sample will be classified as private regions.

**1.3 Demo data**

The demo data is used to demonstrate the functions in APAVplot. It is derived from the Simons Genome Diversity Project (SGDP, Mallick S, 2016). We aligned the reads to a human pan-genome and obtained the coding sequence (CDS) coverage for each gene. We selected 111 samples from Asia, including East Asia, South Asia, and Central Asia/Siberia, focusing on genes located on autosomes. To present the results of the functions more clearly, we excluded genes that exhibited 100% coverage across all samples.

library (APAVplot)

data(“cov_data”)

data(“pav_data”)

data(“gene_info_data”)

data(“pheno_info_data”)

knitr::kable(head(cov_data[, 1:6]))

|  | **ERR1025638** | **ERR1395609** | **ERR1347719** |  | **ERR1395552** | **ERR1395559** | **ERR1347692** |
| --- | --- | --- | --- | --- | --- | --- | --- |
| ENSG00000004948.14 | 1 | 1.0000 | 0.9882 |  | 0.981 | 0.9967 | 0.9804 |
| ENSG00000005884.18 | 1 | 0.9946 | 1.0000 |  | 1.000 | 1.0000 | 1.0000 |
| ENSG00000006007.12 | 1 | 1.0000 | 1.0000 |  | 1.000 | 1.0000 | 1.0000 |
| ENSG00000006016.11 | 1 | 1.0000 | 1.0000 |  | 1.000 | 1.0000 | 1.0000 |
| ENSG00000006432.15 | 1 | 1.0000 | 1.0000 |  | 1.000 | 1.0000 | 1.0000 |
| ENSG00000008323.15 | 1 | 1.0000 | 1.0000 |  | 1.000 | 1.0000 | 1.0000 |

knitr::kable(head(pav_data[, 1:6]))

|  | **ERR1025638** | **ERR1395609** | **ERR1347719** | **ERR1395552** | **ERR1395559** | **ERR1347692** |
| --- | --- | --- | --- | --- | --- | --- |
| ENSG00000004948.14 | 1 | 1 | 1 | 1 | 1 | 1 |
| ENSG00000005884.18 | 1 | 1 | 1 | 1 | 1 | 1 |
| ENSG00000006007.12 | 1 | 1 | 1 | 1 | 1 | 1 |
| ENSG00000006016.11 | 1 | 1 | 1 | 1 | 1 | 1 |
| ENSG00000006432.15 | 1 | 1 | 1 | 1 | 1 | 1 |
| ENSG00000008323.15 | 1 | 1 | 1 | 1 | 1 | 1 |

knitr::kable(head(gene_info_data))

|  | **chr** | **start** | **end** | **length** | **chr_n** |
| --- | --- | --- | --- | --- | --- |
| ENSG00000004948.14 | chr7 | 93424487 | 93574730 | 150244 | 7 |
| ENSG00000005884.18 | chr17 | 50055968 | 50090481 | 34514 | 17 |
| ENSG00000006007.12 | chr16 | 19501693 | 19522123 | 20431 | 16 |
| ENSG00000006016.11 | chr19 | 18572220 | 18607741 | 35522 | 19 |
| ENSG00000006432.15 | chr14 | 70722526 | 70809534 | 87009 | 14 |
| ENSG00000008323.15 | chr12 | 6310436 | 6328506 | 18071 | 12 |

knitr::kable(head(pheno_info_data))

|  | **Genetic_sex** | **DNA_source** | **Region** | **Country** | **Coverage_mean** |
| --- | --- | --- | --- | --- | --- |
| ERR1025638 | XY | Genomic_from_cell_lines | EastAsia | Vietnam | 37.20 |
| ERR1395609 | XY | Genomic_from_blood | SouthAsia | India | 46.13 |
| ERR1347719 | XY | Genomic_from_cell_lines | EastAsia | China | 41.35 |
| ERR1395552 | XY | Genomic_from_blood | SouthAsia | India | 48.87 |
| ERR1395559 | XY | Genomic_from_blood | SouthAsia | India | 51.50 |
| ERR1347692 | XX | Genomic_from_cell_lines | EastAsia | Thailand | 45.61 |

my_cov <- get_cov_obj(cov_data, region_info = gene_info_data, pheno_info = pheno_info_data)

my_pav <- get_pav_obj(pav_data, region_info = gene_info_data, pheno_info = pheno_info_data)

**2 Visualization of coverage**

**2.1 cov_heatmap()**

A heatmap offers an extensive overview of coverage across samples. The color scheme of the heatmap can be customized by setting cov_colors. Both rows and columns can be clustered, with several general settings available for clustering. These settings include options to apply clustering, display dendrograms, specify the side of the dendrograms, and adjust their width.

cov_heatmap(my_cov,

cov_colors = c(“white”, “#9BCD9B”),

cluster_rows = T,

row_dend_width = grid::unit(2, “mm”),

row_dend_side = “right”,

cluster_columns = T)


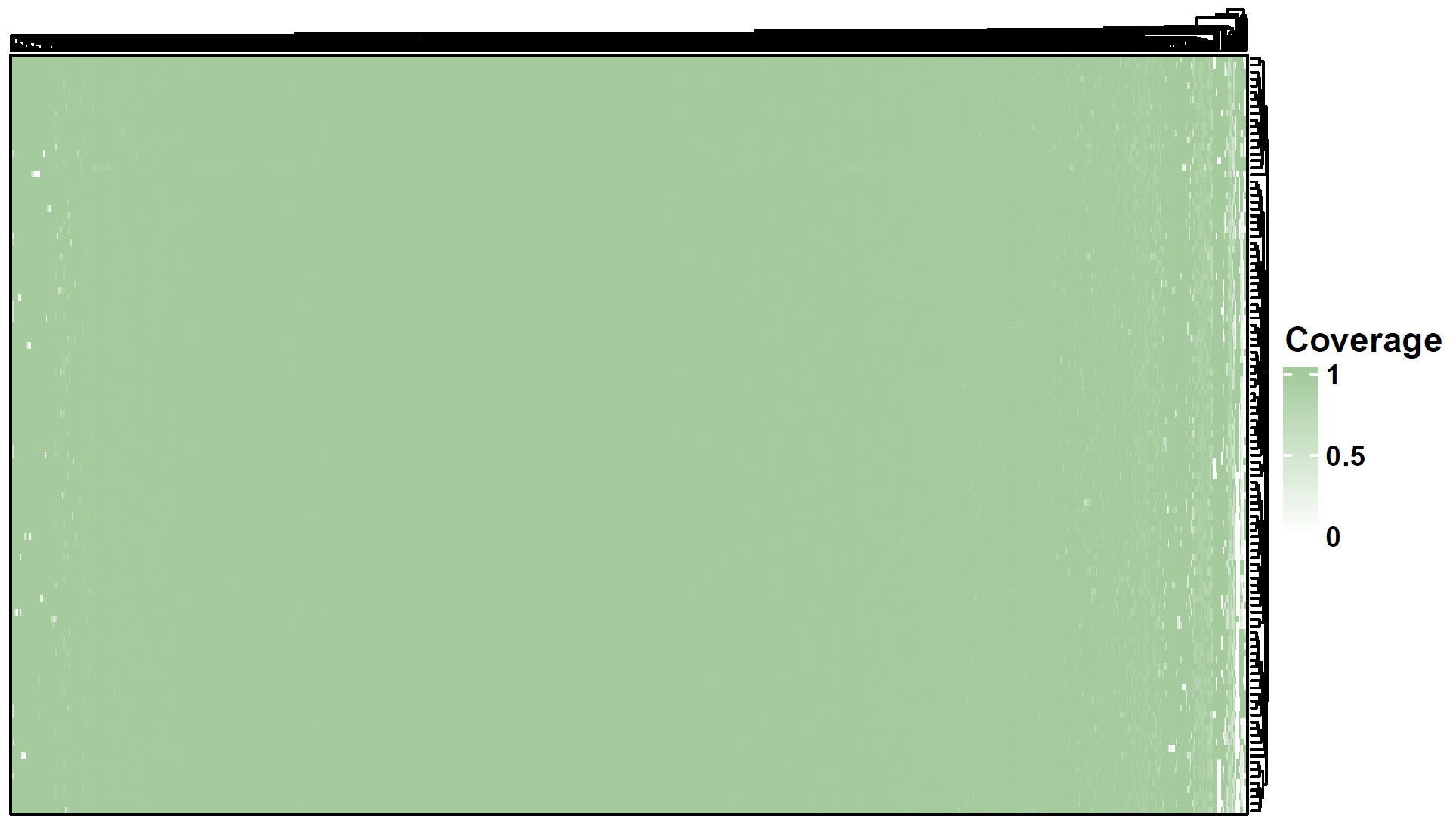


The parameters pheno_info and region_info allow the integration of phenotype information and region annotations into the plot. The anno_param_column_region and anno_param_row_pheno represent the list of parameters. The region_info_color_list and pheno_info_color_list are used to modify the colors of the annotations.

cov_heatmap(my_cov,

cluster_rows = T,

cluster_columns = T,

add_pheno_info = c(“Genetic_sex”, “Region”),

pheno_info_color_list = list(

Genetic_sex = c(“XX” = “#A6D854”, “XY” = “#8DA0CB”, “Not Assigned” = “gray70”),

Region = structure(c(“#66C2A5”, “#FFD92F”, “#FC8D62”),

names = unique(pheno_info_data$Region))),

add_region_info = c(“length”),

region_info_color_list = list(length = c(“#dbebfa”, “#377EB8”)))


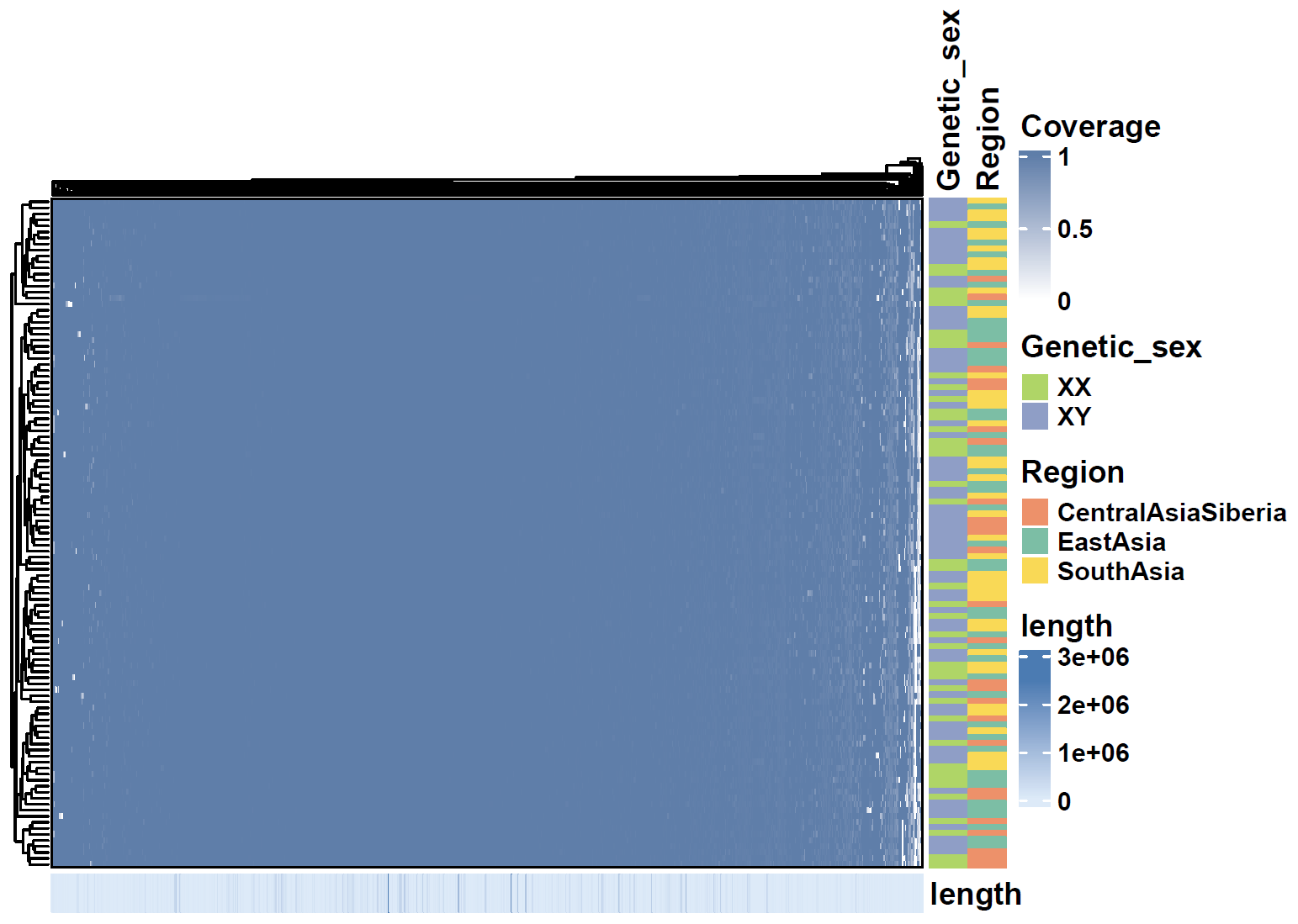


**2.2 cov_density()**

Then, you can focus on several genes of interest.

genes <- names(head(sort(apply(cov_data, 1, median))))

cov_density(my_cov, genes)


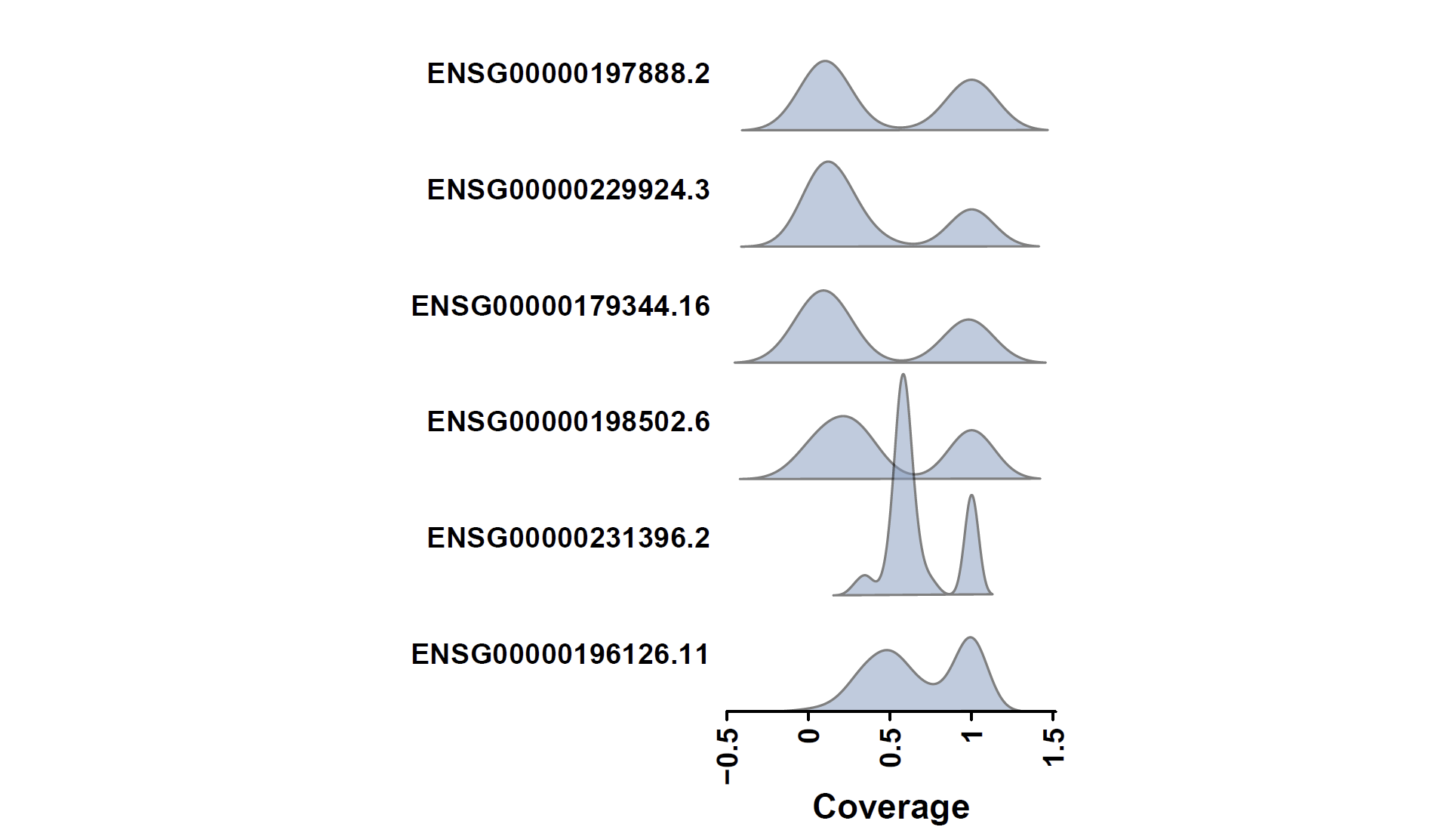


The region_info can be included as well.

cov_density(my_cov, genes,

row_names_side = “right”,

add_region_info = c(“chr”, “length”),

region_info_color_list = list(length = c(“#dbebfa”, “#377EB8”)))


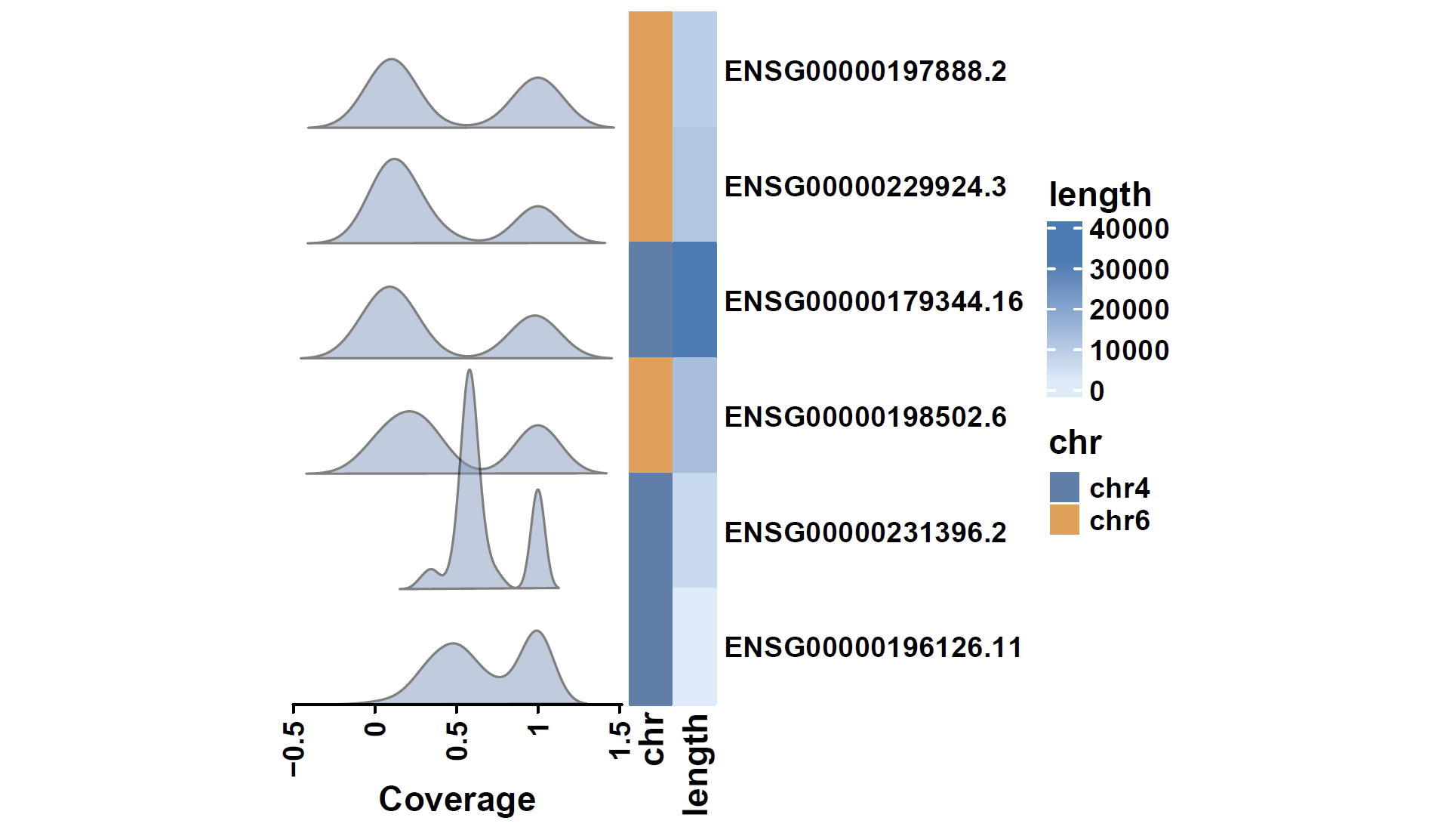


**3 PAV statistics and analysis**

**3.1 cov_halfviolin()**

You can observe the number of target regions present in samples using a half-violin plot. The left half of the plot displays the density estimate, while each point on the right represents an individual sample.

pav_halfviolin(my_pav)


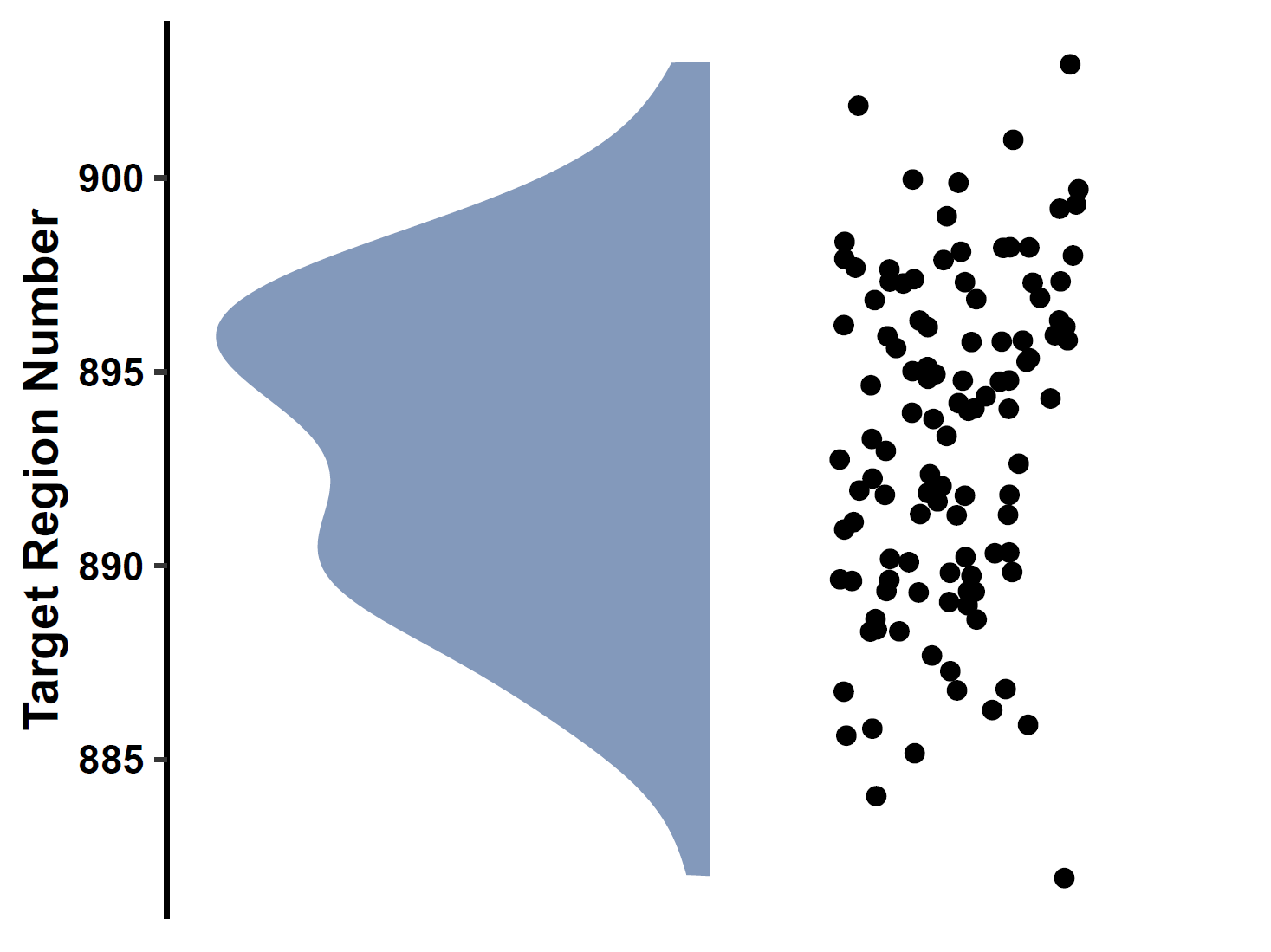


If you add pheno_info, the points will be categorized based on their phenotype.

pav_halfviolin(my_pav,

add_pheno_info = “Region”)


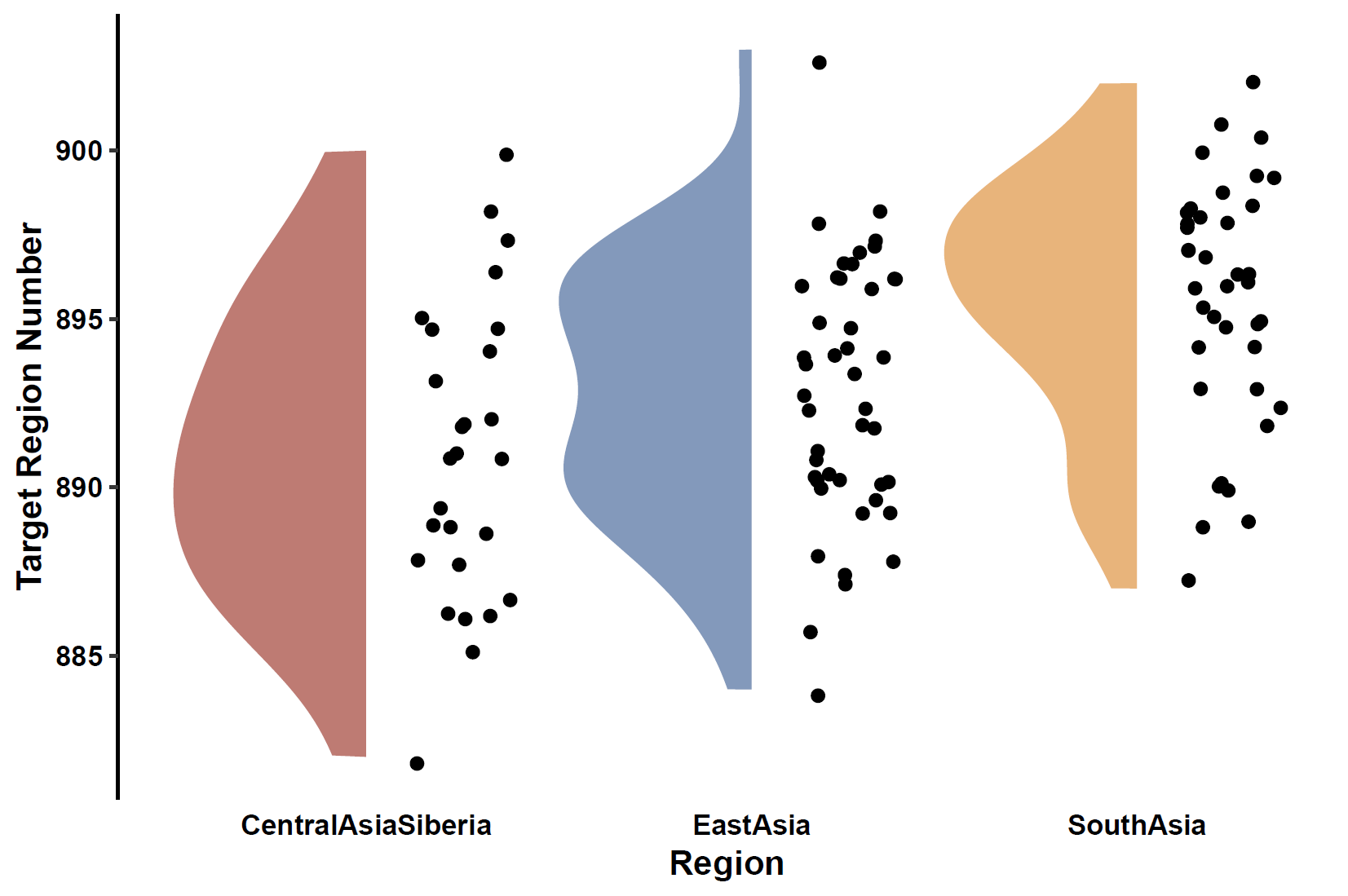


You can set the color for the specified group.

pav_halfviolin(my_pav,

add_pheno_info = “Region”,

pheno_info_color_list = list(

Region = c(CentralAsiaSiberia = “#9BCD9B”)

),

y_title_size = 13)


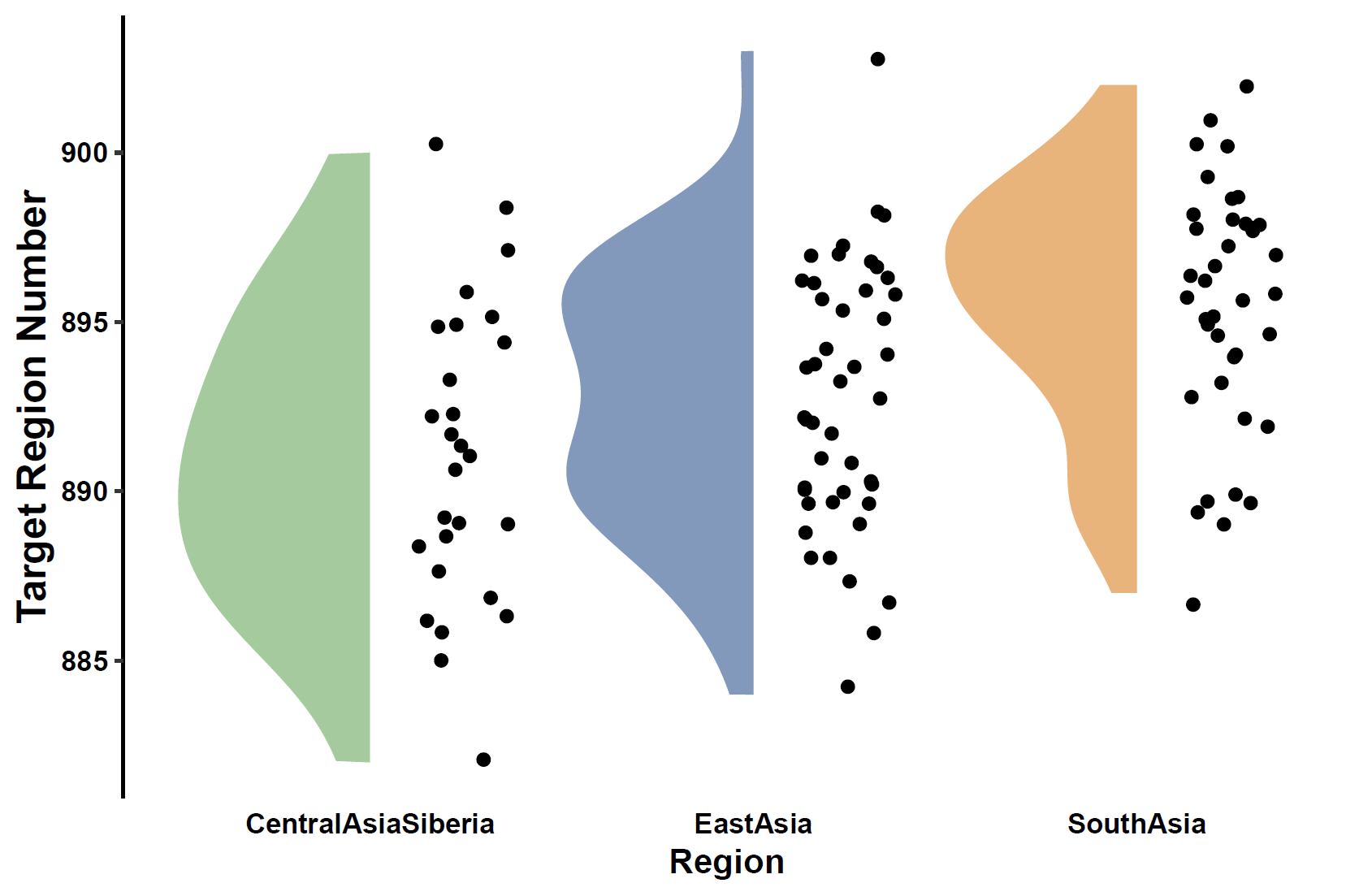


**3.2 pav_hist()**

The target regions can be categorized into multiple types based on the number of samples containing them. The pav_hist() function integrates a ring chart and a histogram to display the number of types. The parameters ring_pos_x, ring_pos_y, and ring_r specify the position and radius of the ring chart. The x-axis of the histogram represents the number of samples, ranging from 1 to the total number of samples. The y-axis indicates the number of regions shared by x samples.

pav_hist(my_pav,

ring_r = .45,

y_title = “Number of genes”)


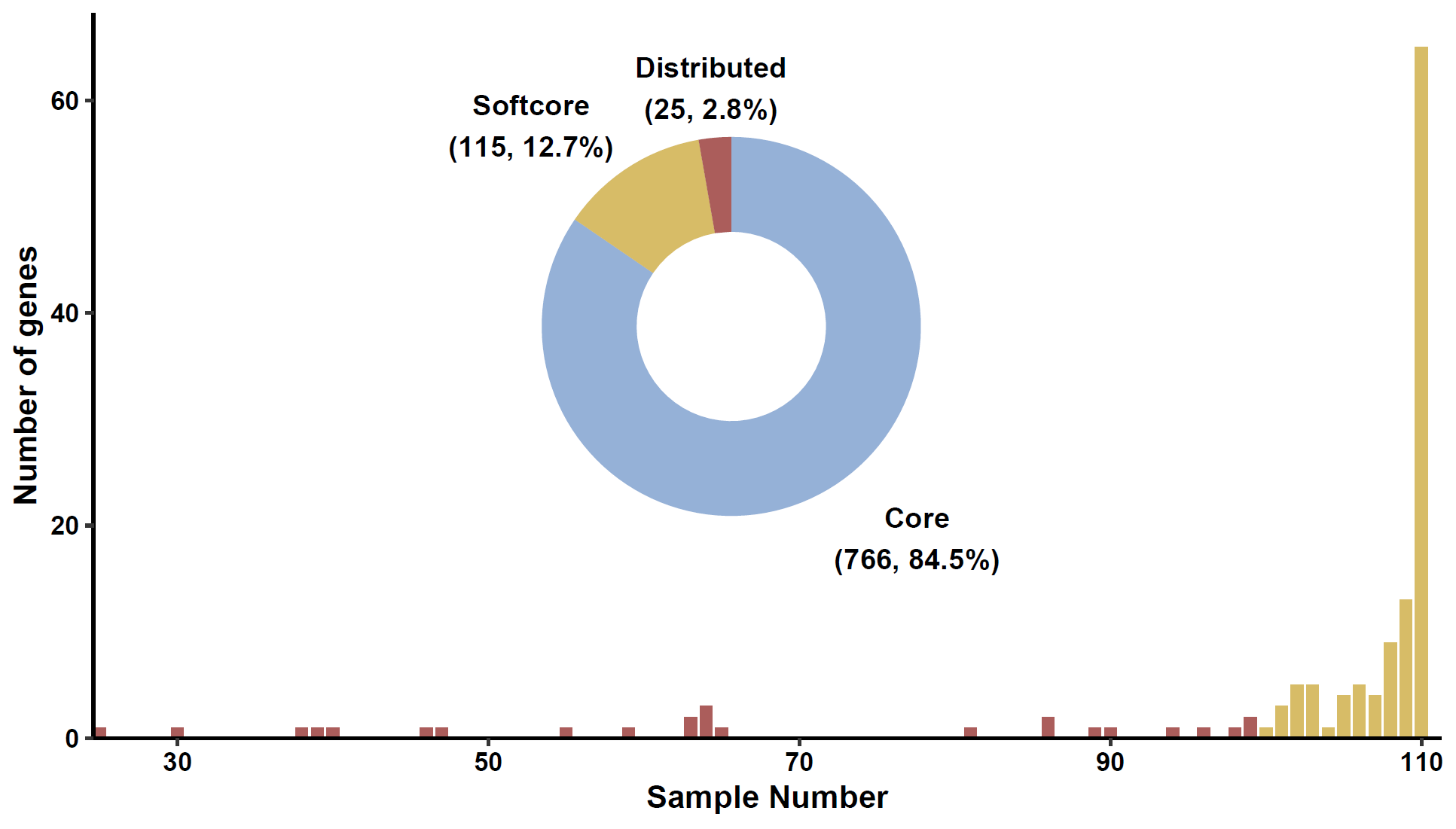


You can adjust the position and size of the ring, as well as the size of the labels.

pav_hist(my_pav,

ring_pos_x = 0.3,

ring_pos_y = 0.7,

ring_r = .4,

ring_label_size = 3,

type_colors = c(Softcore = “#9BCD9B”),

x_breaks = c(1, 99, 110),

y_title = “Number of genes”)


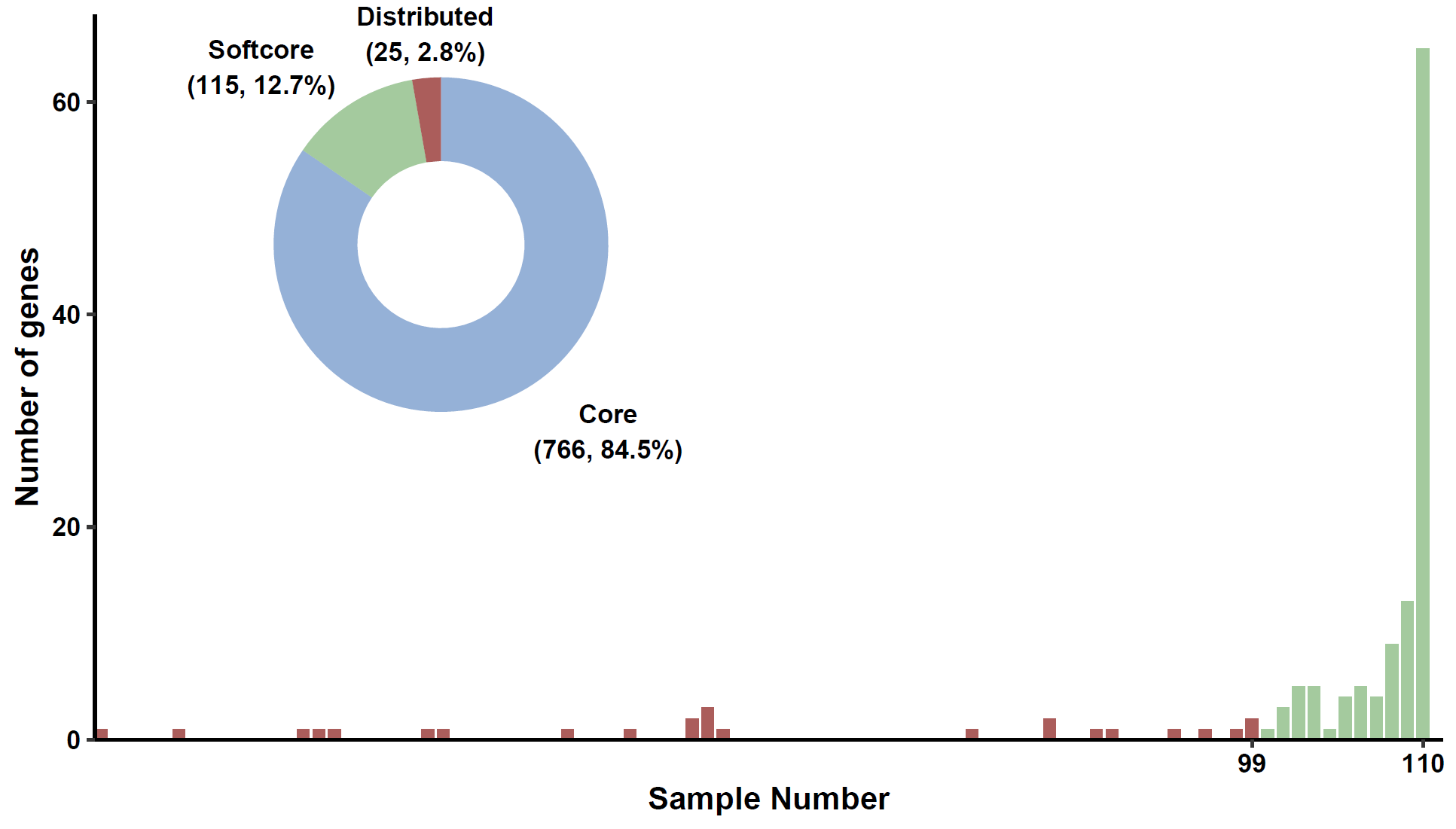


**3.3 pav_stackbar()**

The composition of target regions across all samples can be visualized using the pav_stackbar() function. This chart consists of a hierarchically clustered tree alongside a bar plot. The parameters dend_width and name_width denote the relative widths of the dendrogram and sample names, respectively. The dashed line and numerical labels indicate the mean values of cumulative sums. For example, the first line represents the mean of the core region count, while the second line indicates the mean of both core and soft-core regions.

pav_stackbar(my_pav,

name_width = .17,

dend_width = .1,

sample_name_size = 2)


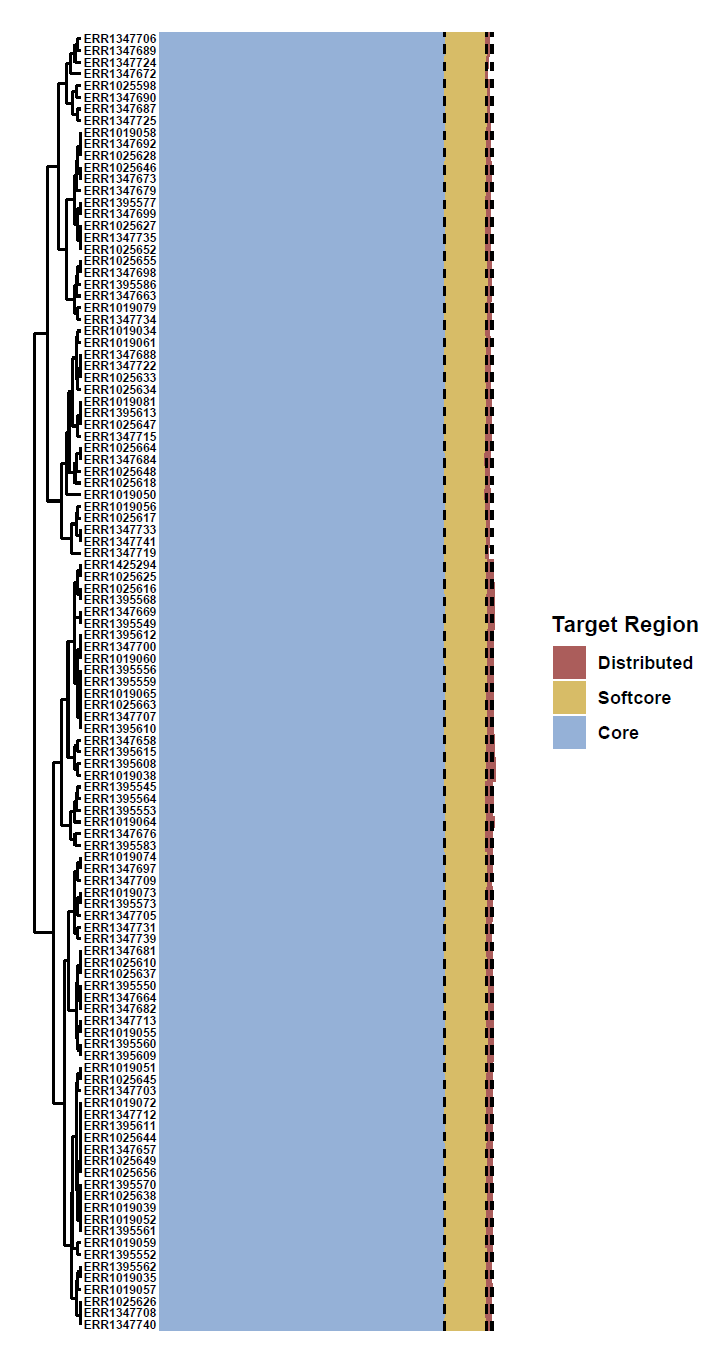


When including pheno_info, the sample names will be displayed in color.

pav_stackbar(my_pav,

name_width = .17,

dend_width = .1,

sample_name_size = 2,

add_pheno_info = “Region”)


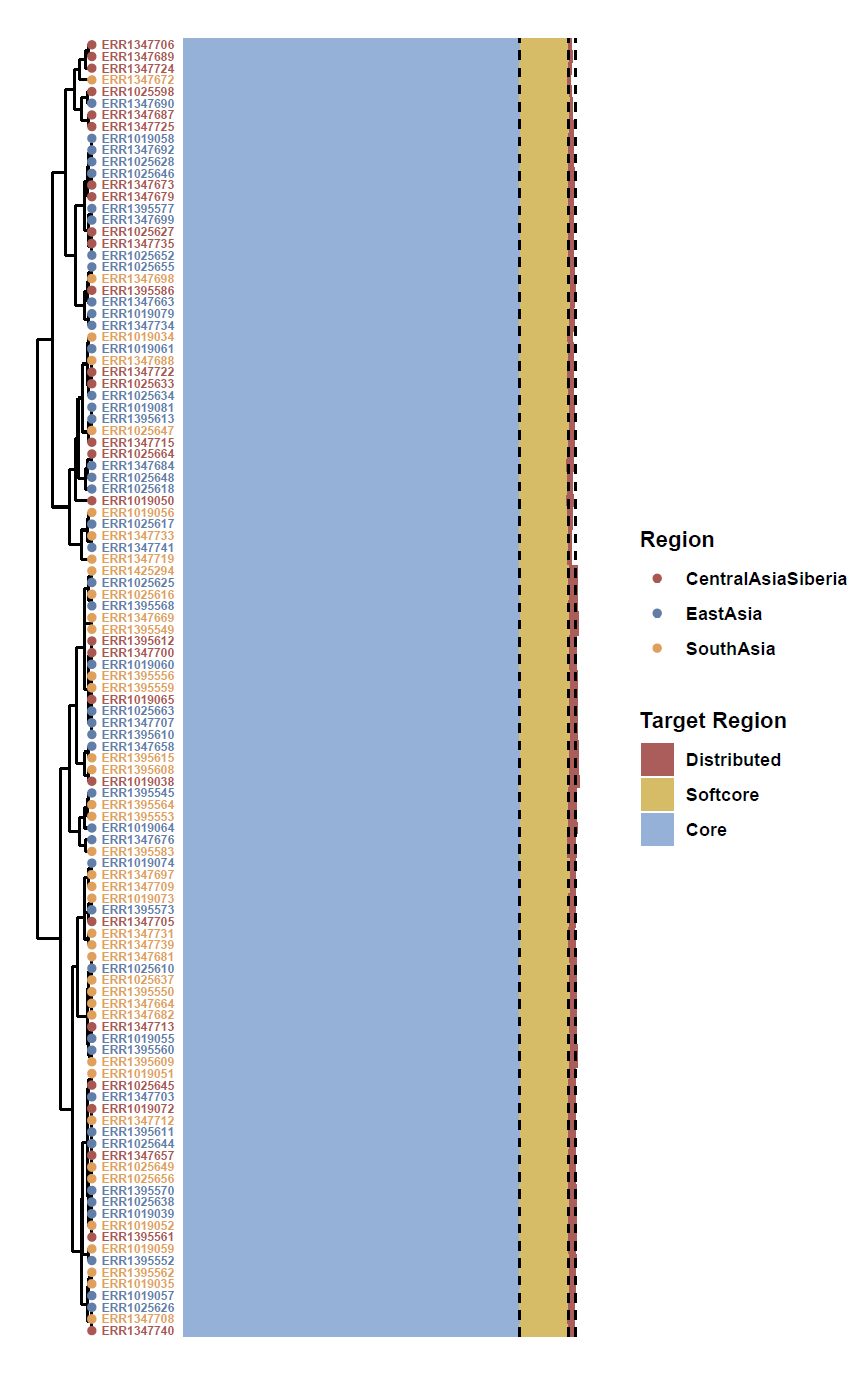


**3.4 pav_heatmap()**

The pav_heatmap() function generates a heatmap that includes two summary annotations. The columns are split into blocks based on region types. If the parameter split_block is set to FALSE, the split line will be removed.

pav_heatmap(my_pav,

region_type = c(“Core”, “Softcore”, “Distributed”),

split_block = F)


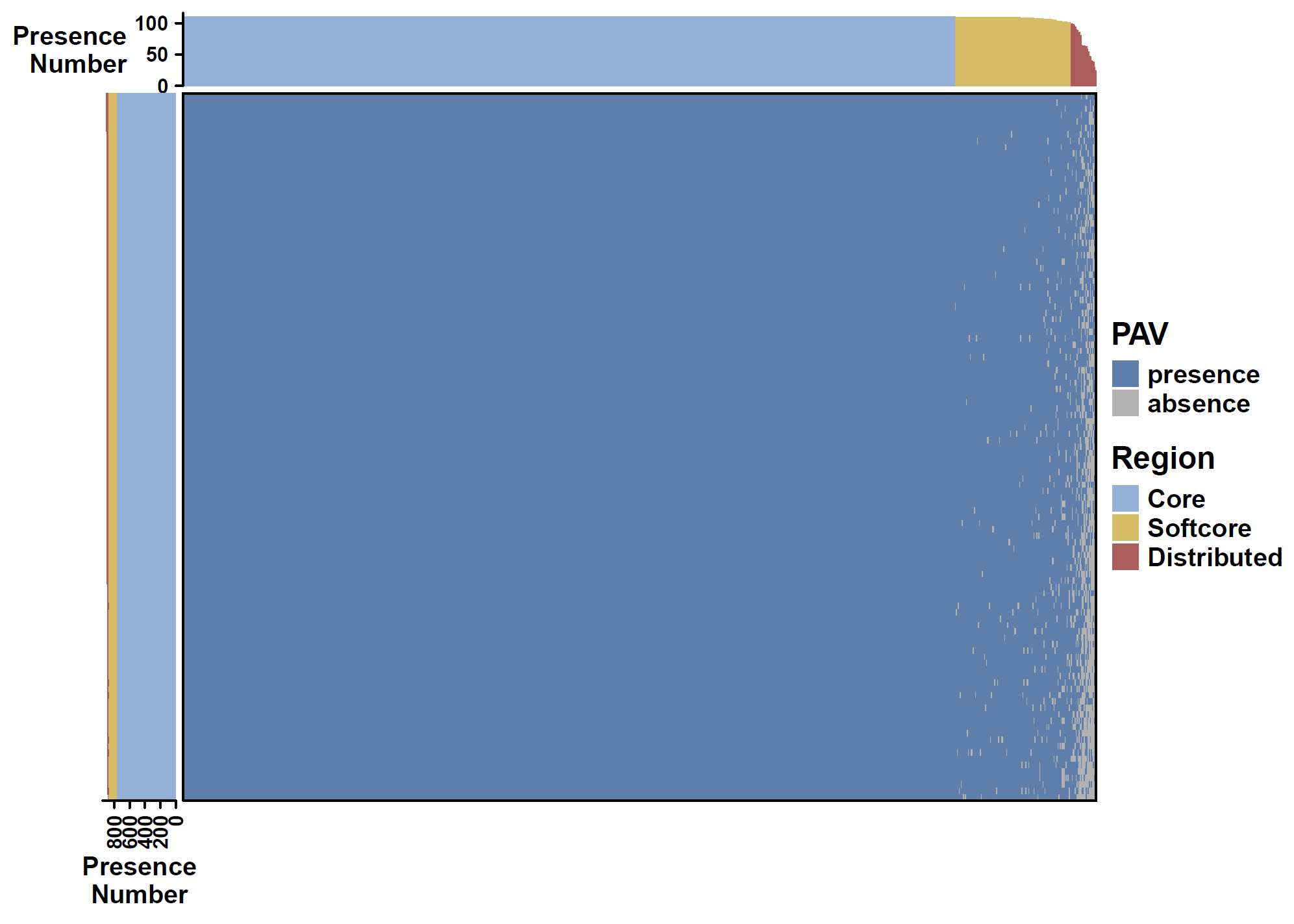


By default, the heatmap is organized into blocks according to categories. You can modify the names of the blocks in the upper panel using the parameters block_name_size and block_name_rot.

pav_heatmap(my_pav,

region_type = c(“Core”, “Softcore”, “Distributed”),

block_name_size = 10,

block_name_rot = 90)


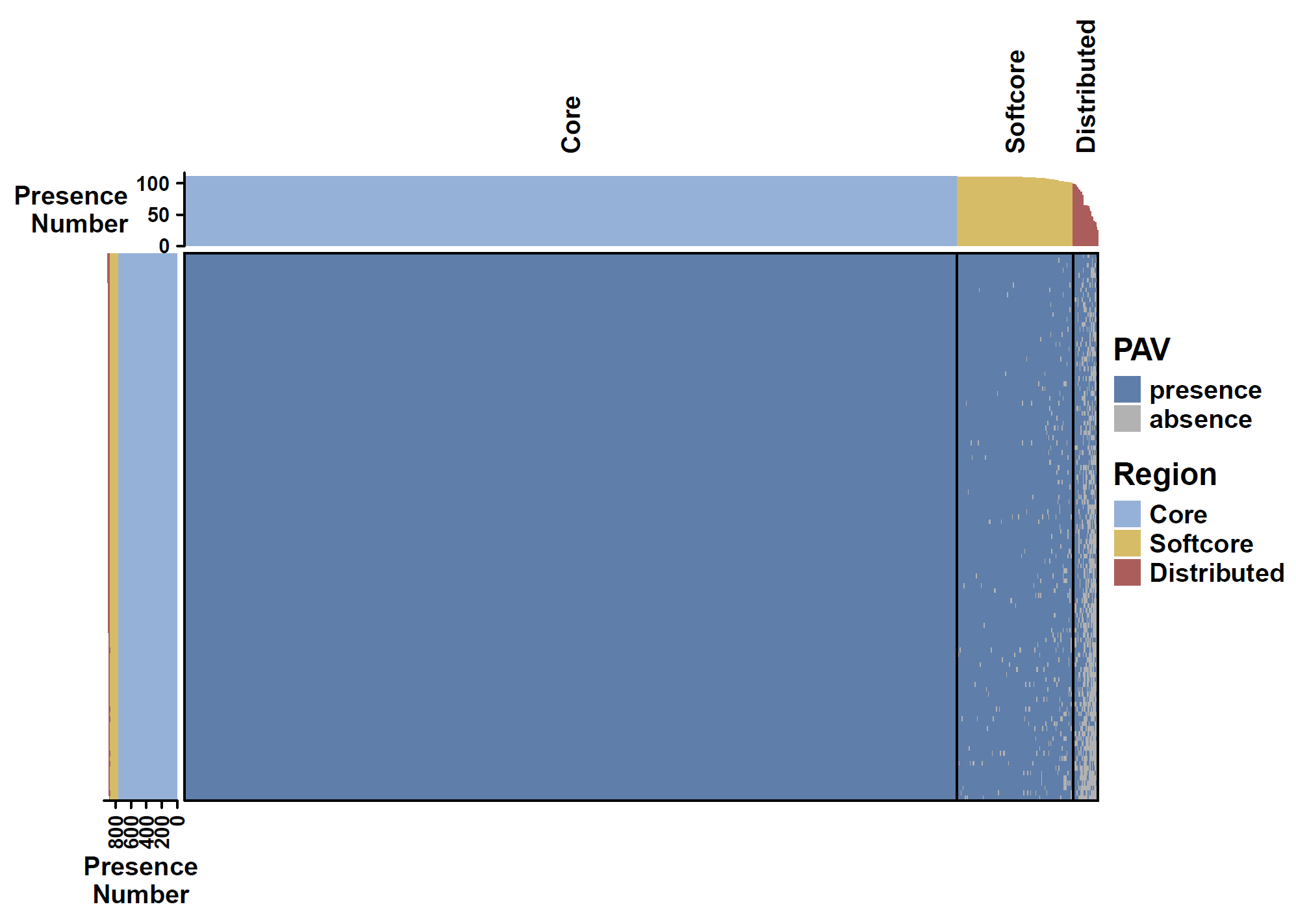


You can also hide the borders of the blocks by setting block = FALSE.

pav_heatmap(my_pav,

region_type = c(“Core”, “Softcore”, “Distributed”),

block_name_size = 10,

block_name_rot = 90,

border = F)


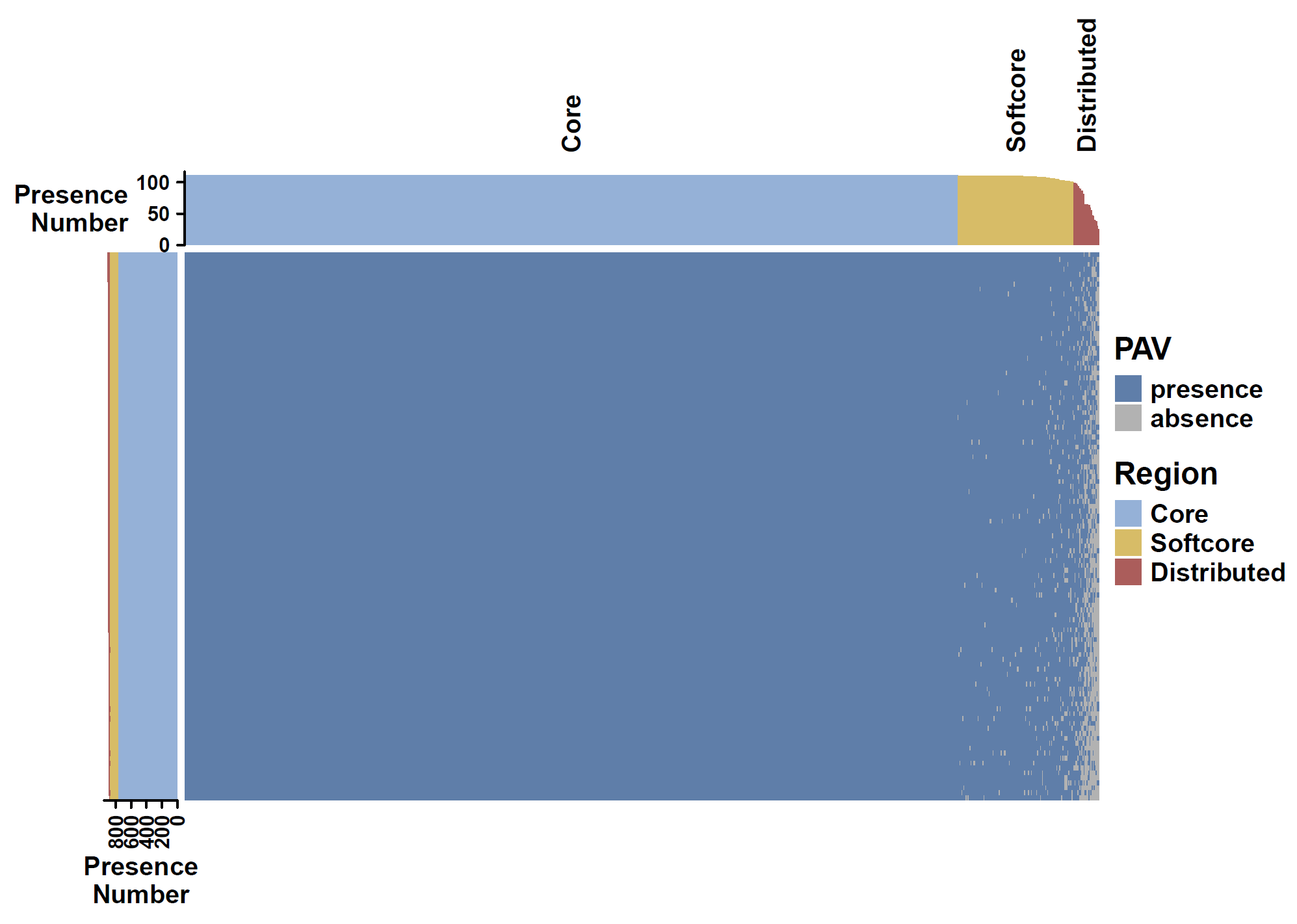


The heatmap can be configured to display specific categories only.

pav_heatmap(my_pav,

region_type = c(“Distributed”),

show_column_names = T,

column_names_size = 8,

anno_param_row_stat = list(title_size = 9),

anno_param_column_stat = list(title_size = 9))


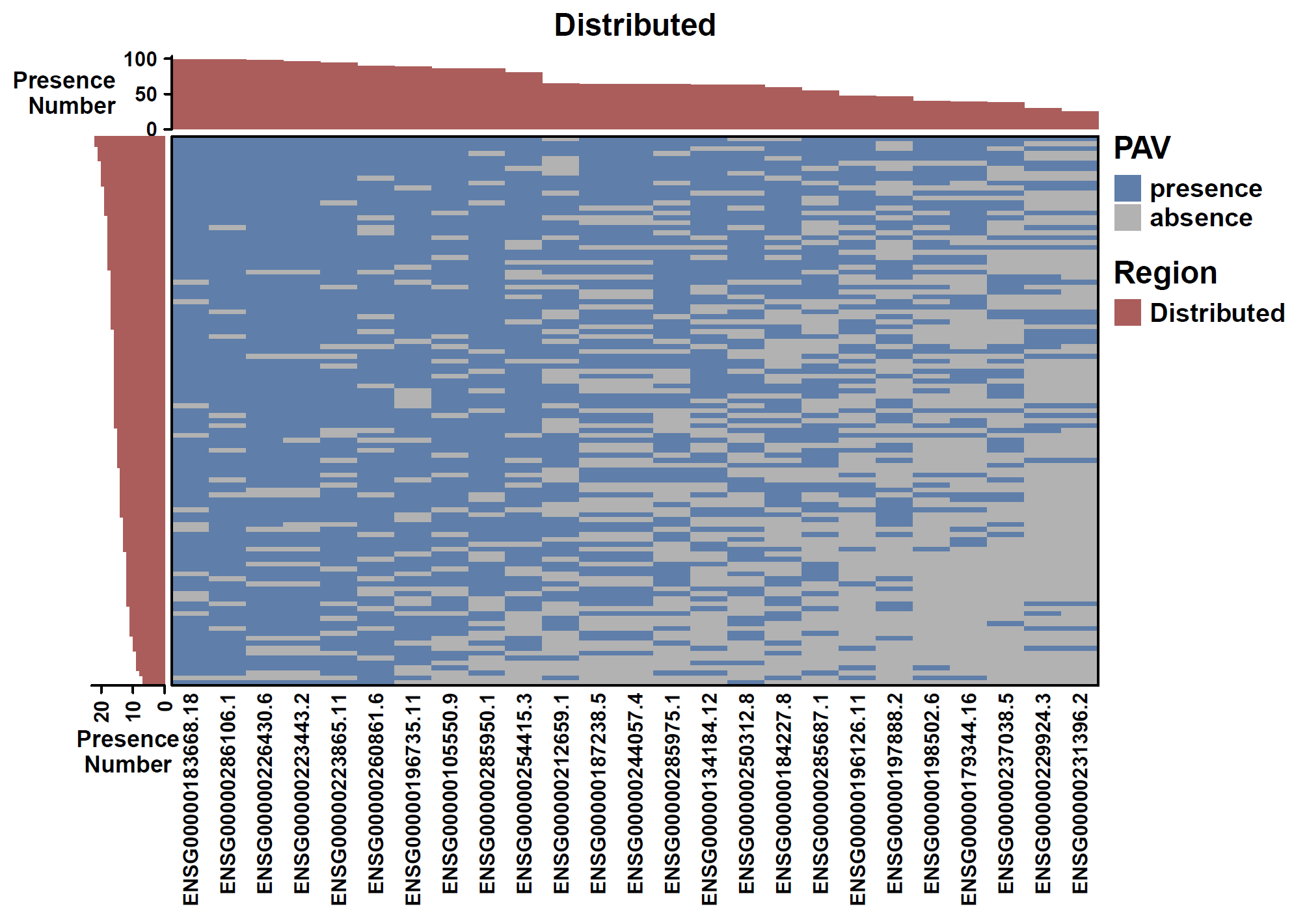


The rows and columns can be clustered. Please note that when the number of rows or columns is large, the process may take a long time. The methods for computing the distance matrix are specified by clustering_distance_rows and clustering_distance_columns. The methods for performing hierarchical clustering are defined by clustering_method_rows and clustering_method_columns. The position and height/width of the dendrogram can be adjusted using column_dend_side/row_dend_side and column_dend_height/row_dend_width.

pav_heatmap(my_pav,

region_type = c(“Softcore”, “Distributed”),

split_block = FALSE,

cluster_columns = TRUE,

clustering_distance_columns = “binary”,

clustering_method_columns = “average”,

cluster_rows = TRUE,

clustering_distance_rows = “binary”,

clustering_method_rows = “average”,

column_dend_side = “bottom”,

column_dend_height = grid::unit(3, “mm”),

row_dend_side = “right”,

row_dend_width = grid::unit(3, “mm”))


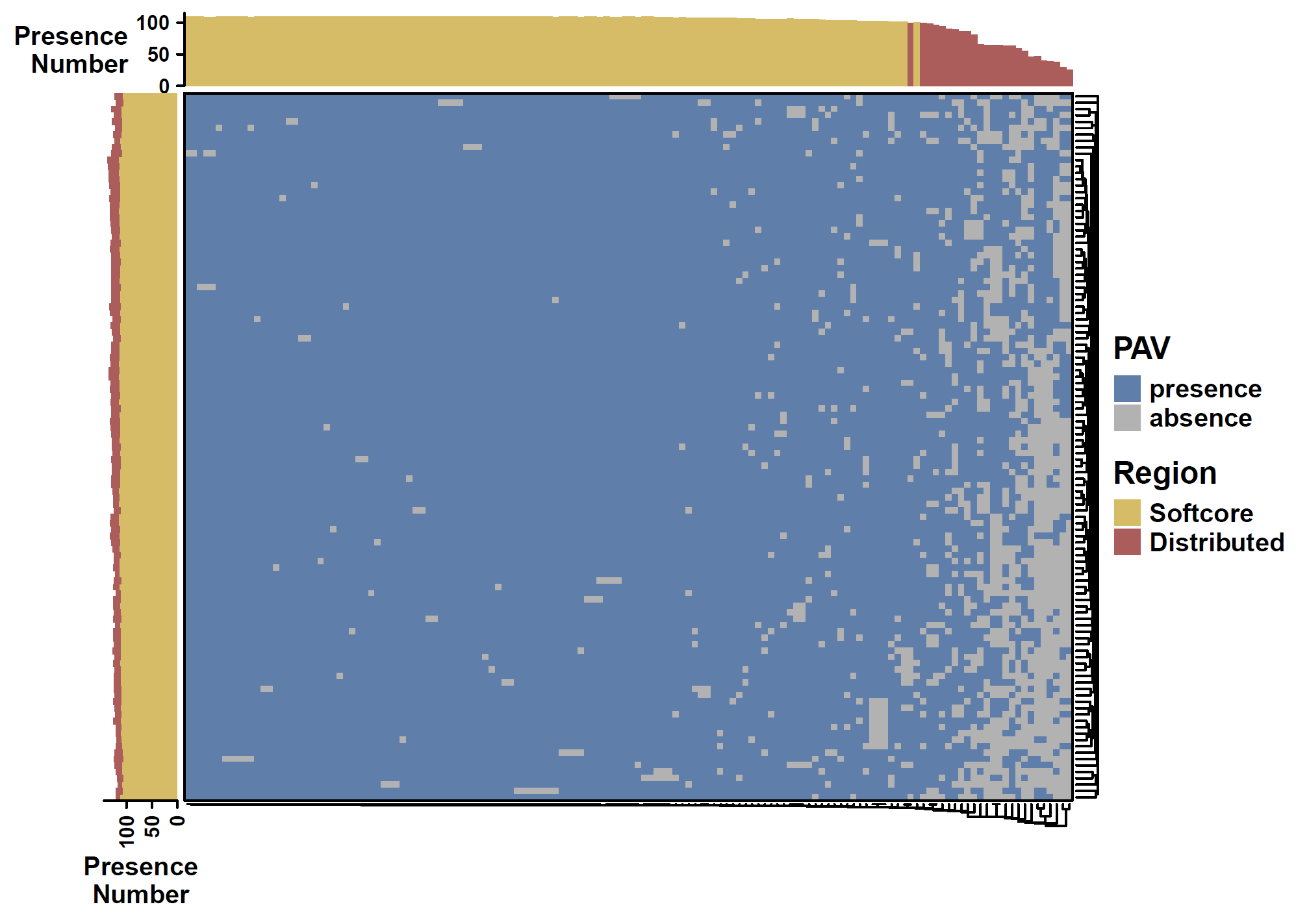


If split_block is set to TRUE and cluster_columns is also set to TRUE, clustering will be performed within each block.

pav_heatmap(my_pav,

region_type = c(“Softcore”, “Distributed”),

split_block = TRUE,

cluster_columns = TRUE,

column_dend_side = “bottom”,

cluster_rows = TRUE,

row_dend_side = “right”)


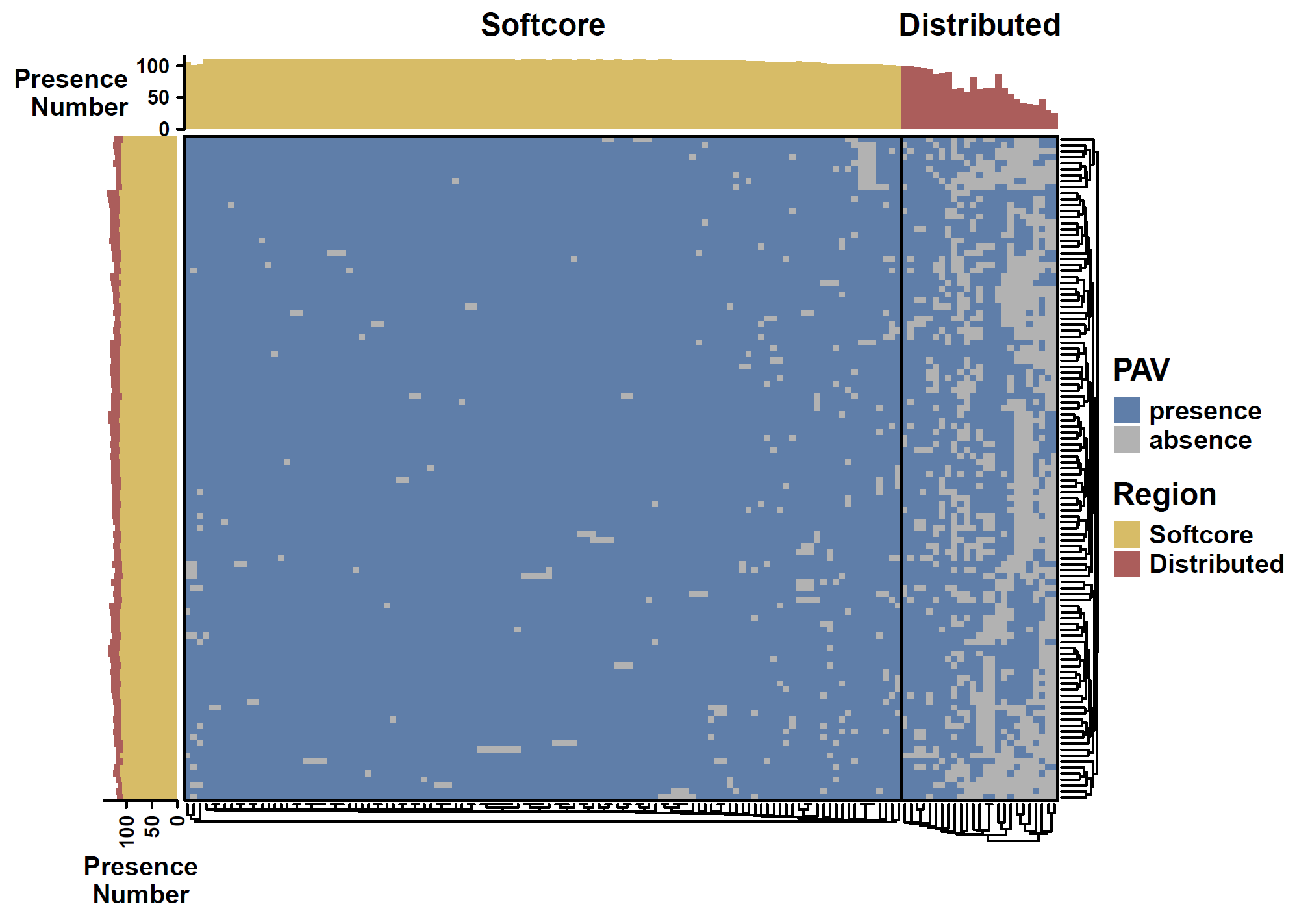


If you want to specify the order of rows or columns, you can set the parameters row_sorted or column_sorted. Please ensure that both cluster_columns and split_block are set to FALSE to achieve the desired outcome.

pav_heatmap(my_pav,

region_type = c(“Softcore”, “Distributed”),

add_pheno_info = c(“Genetic_sex”),

row_sorted = my_pav@sample$name[order(my_pav@sample$pheno$Genetic_sex)])


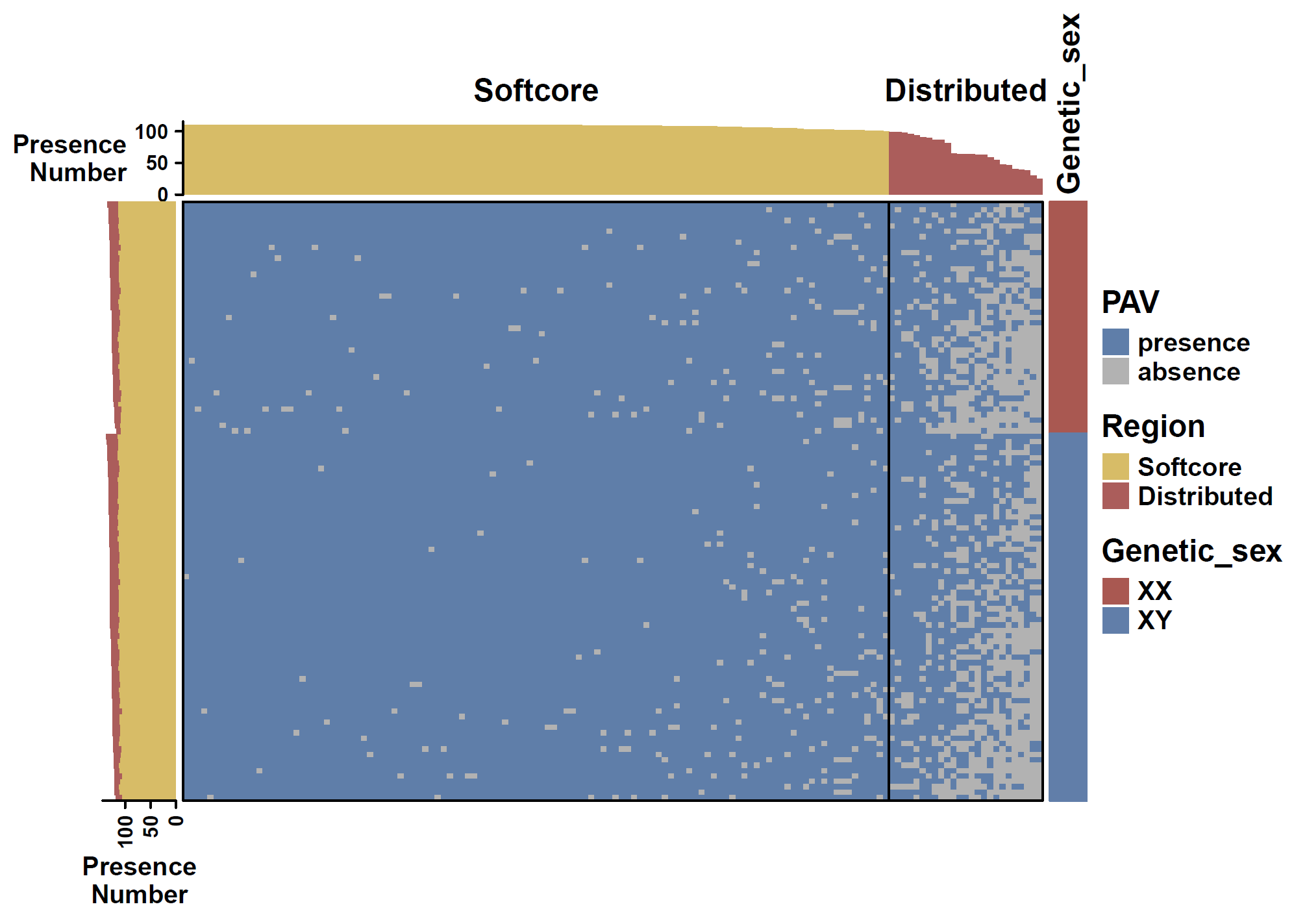


You can hide annotations by setting list(show = FALSE).

pav_heatmap(my_pav,

region_type = c(“Softcore”, “Distributed”),

anno_param_column_stat = list(show = F),

anno_param_row_stat = list(show=F))


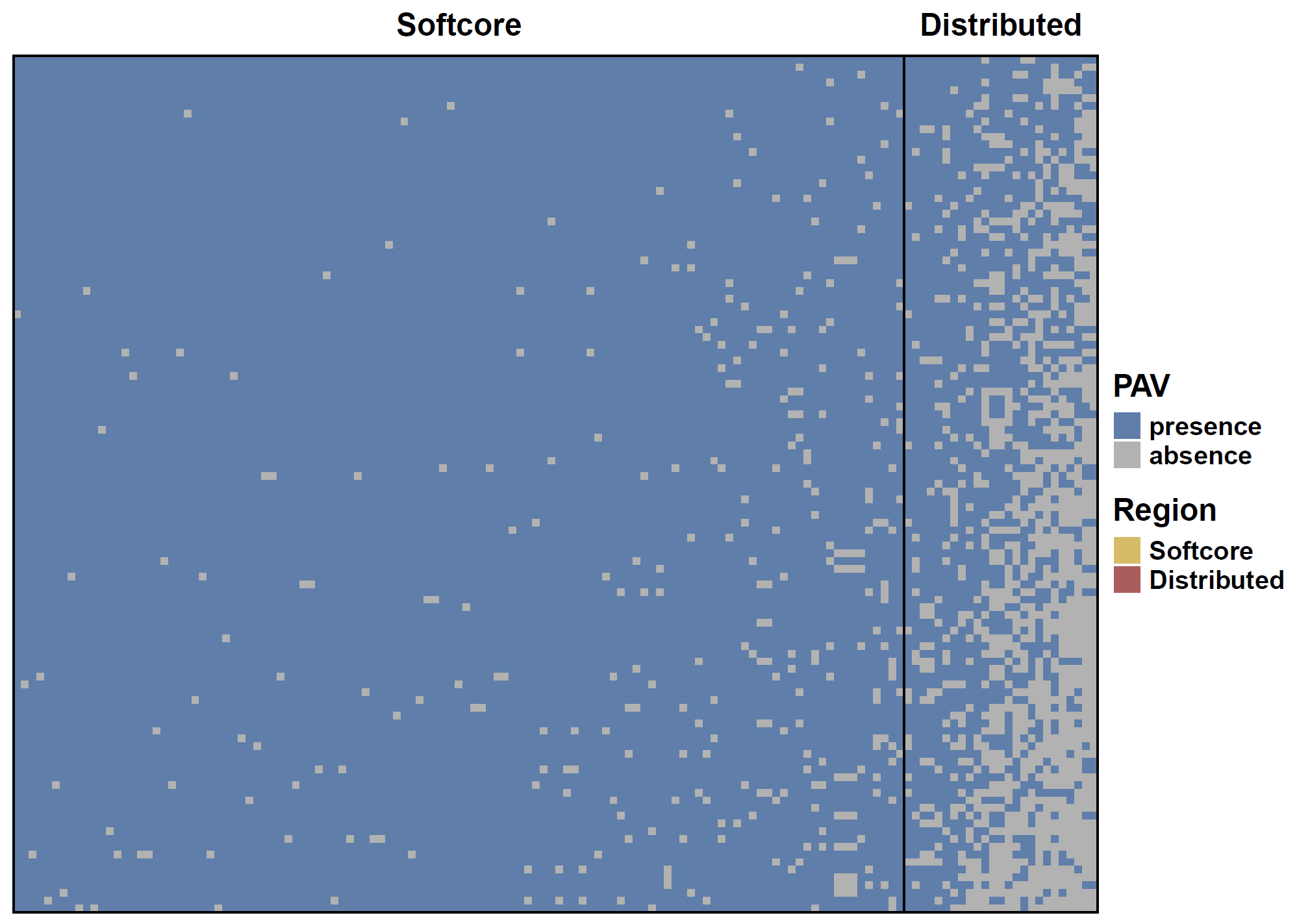


The parameters anno_param_row_stat and anno_param_column_stat are lists that control various annotation features.

pav_heatmap(my_pav,

region_type = c(“Softcore”, “Distributed”),

split_block = TRUE,

column_dend_side = “bottom”,

anno_param_column_stat = list(title = “Gene\nNumber”,

title_size = 10,

height = 6,

axis_at = c(55.5, 111),

axis_labels = c(“50%”, “100%”),

axis_labels_size = 8))


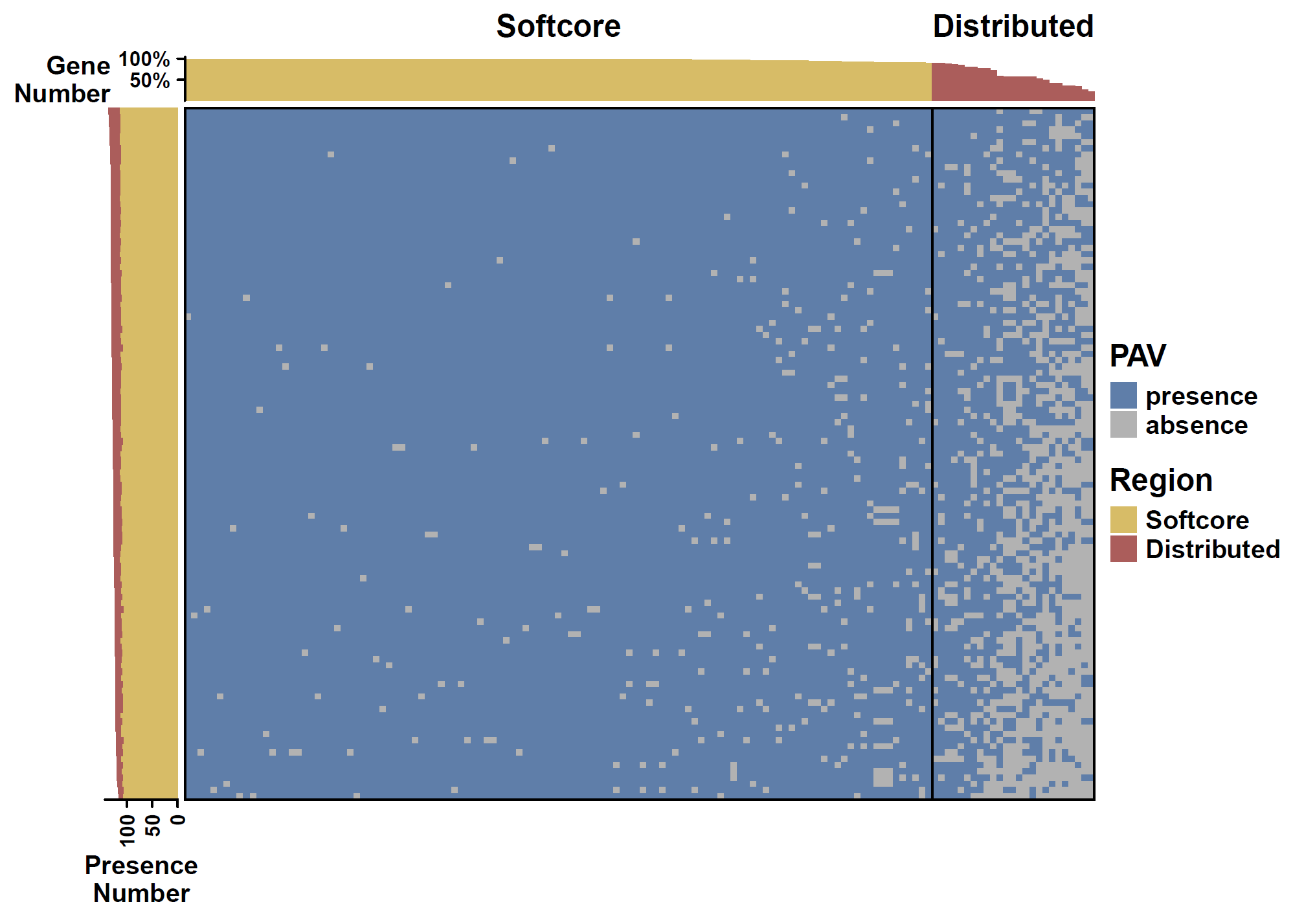


The pheno_info and region_info can be integrated into the figure. The parameters anno_param_row_pheno and anno_param_column_region dictate the appearance of these annotations.

pav_heatmap(my_pav,

region_type = c(“Softcore”, “Distributed”),

add_pheno_info = c(“Genetic_sex”, “Region”),

anno_param_row_pheno = list(width = 2, border = F, name_size = 8, name_rot = 60),

add_region_info = c(“length”),

anno_param_column_region = list(height = 3, border = T, name_size = 8, name_side = “right”))


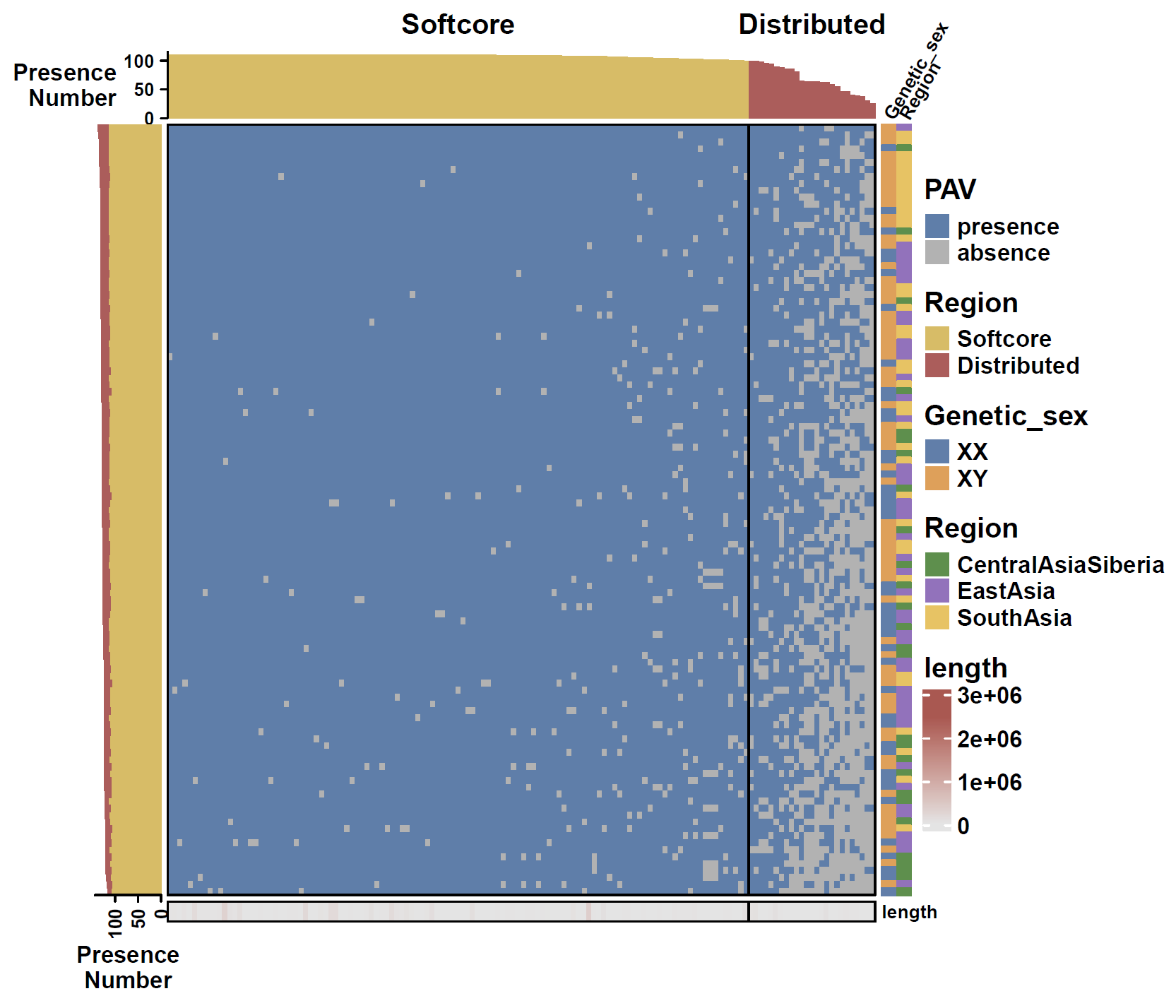


You can adjust the position and size of the legends as needed.

pav_heatmap(my_pav,

region_type = c(“Softcore”, “Distributed”),

add_pheno_info = c(“Genetic_sex”, “Region”),

legend_side = “top”,

legend_title = list(pav = “PAV”, type = “Gene”),

legend_title_size = 10,

legend_text_size = 8)


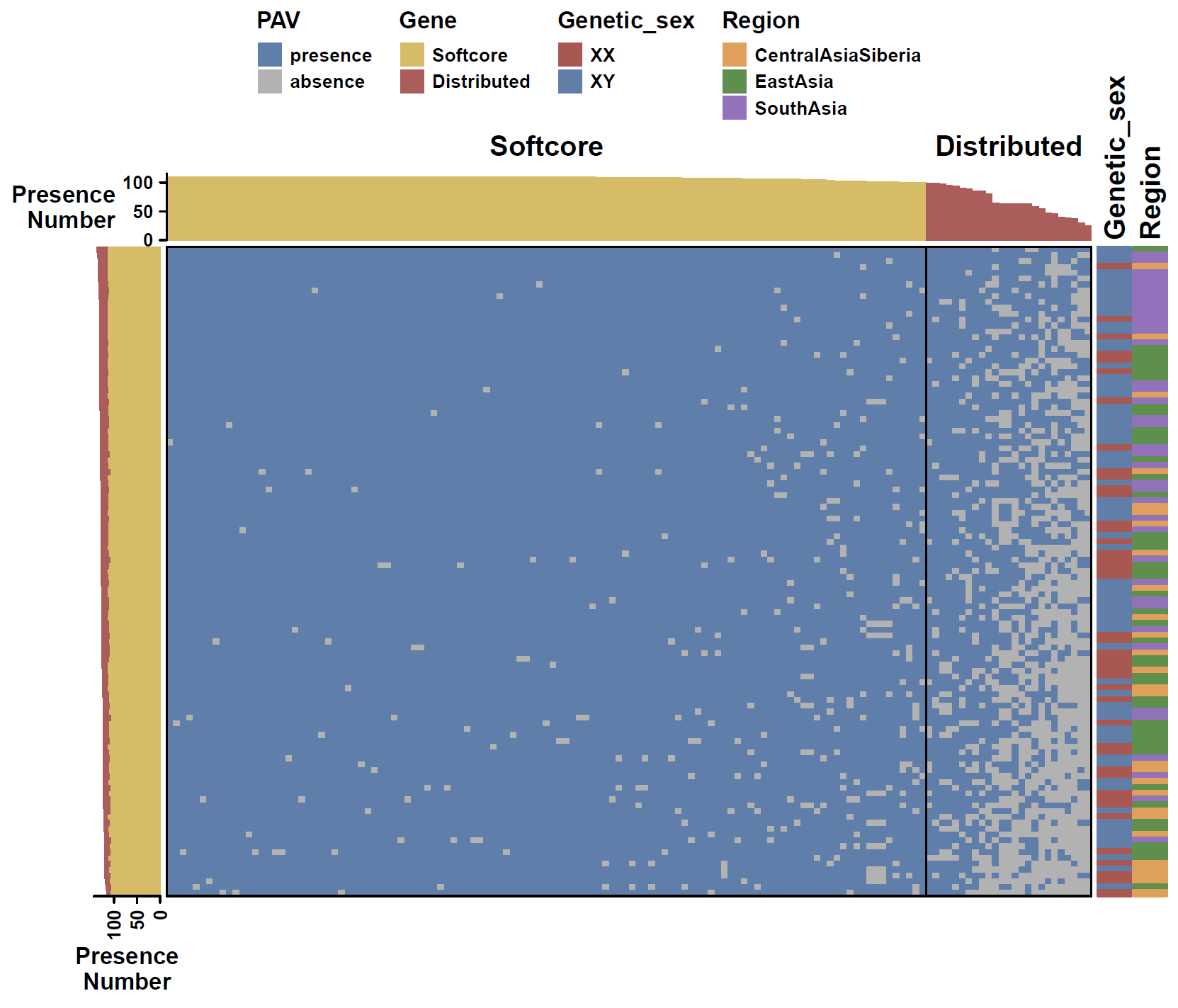


All colors used in your heatmap can be customized. You can provide named vectors for specific color assignments, while unnamed vectors will be displayed in the order they are provided.

pav_heatmap(my_pav,

region_type = c(“Softcore”, “Distributed”),

pav_colors = c(“#CC6E6E”, “gray”),

type_colors = c(Softcore = “#5891BA”, Distributed = “#AFC7E0”),

add_pheno_info = c(“Genetic_sex”, “Region”),

pheno_info_color_list = list(

Genetic_sex = c(“#A6D854”, “#8DA0CB”),

Region = structure(c(“#66C2A5”, “#FFD92F”, “#FC8D62”),

names = unique(pheno_info_data$Region))),

add_region_info = c(“length”),

region_info_color_list = list(length = c(“#dbebfa”, “#377EB8”)))


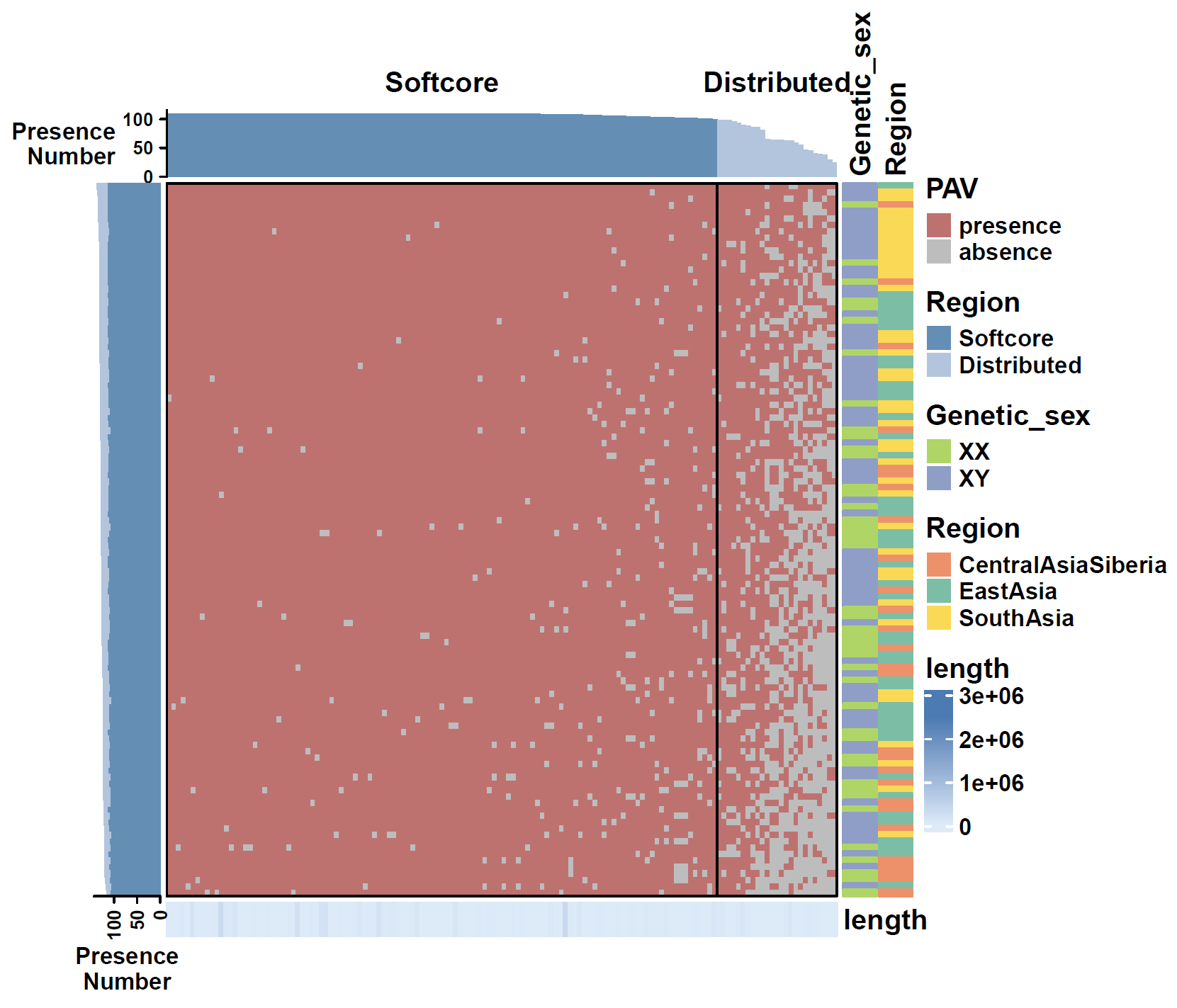


The size of all text elements can also be modified.

pav_heatmap(my_pav,

region_type = c(“Softcore”, “Distributed”),

add_pheno_info = c(“Genetic_sex”, “Region”),

add_region_info = c(“length”),

block_name_size = 2,

show_row_names = T,

row_names_size = 2,

show_column_names = T,

column_names_size = 2,

anno_param_row_pheno = list(name_size = 2),

anno_param_column_region = list(name_size = 2),

anno_param_column_stat = list(title_size = 2, axis_labels_size = 2),

anno_param_row_stat = list(title_size = 2, axis_labels_size = 2),

legend_title_size = 2,

legend_text_size = 2)


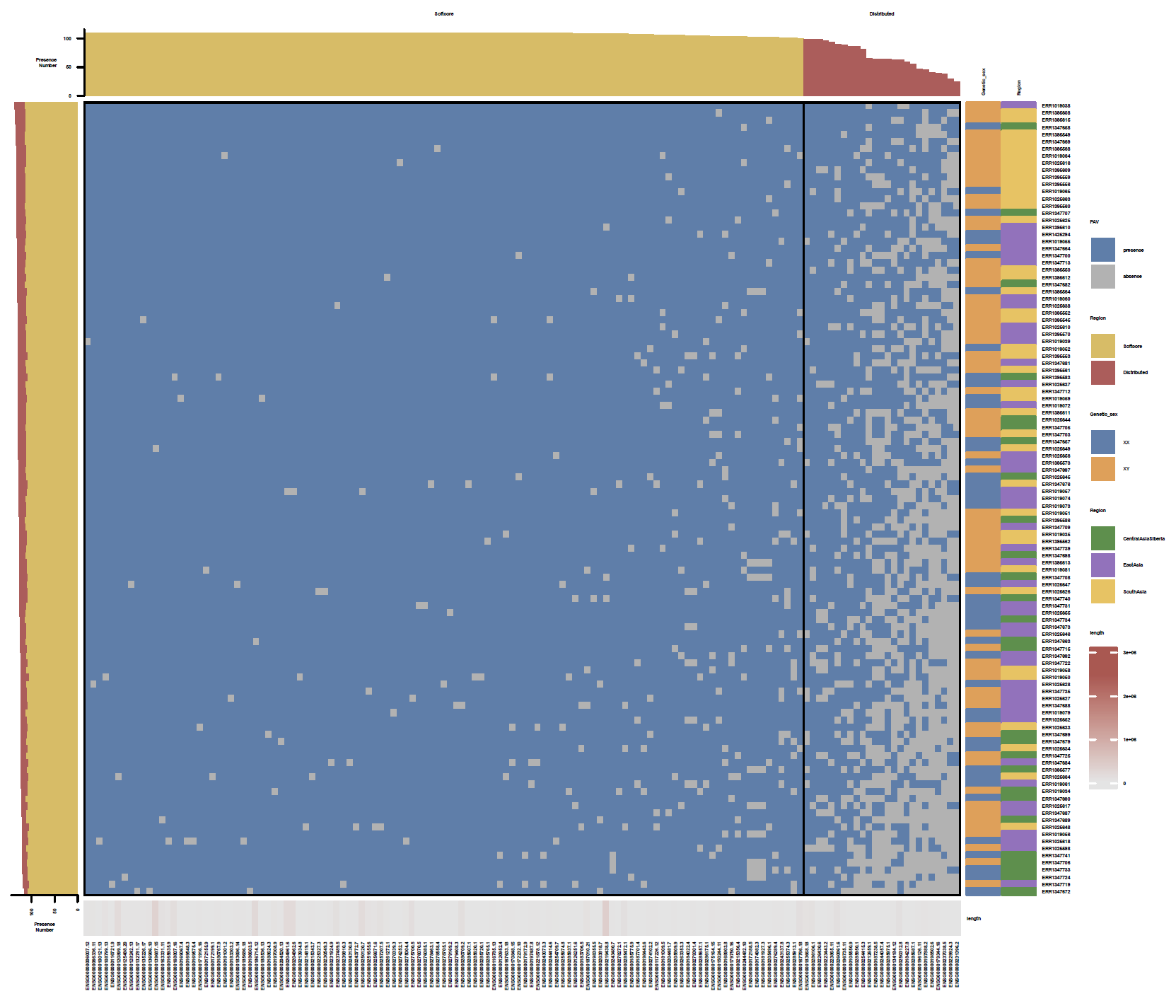


**3.5 pav_ccluster()**

If you want to display the clustering of samples without using a complex heatmap, consider utilizing the pav_cluster() function.

pav_cluster(my_pav,

mult = .3,

sample_name_size = 2)


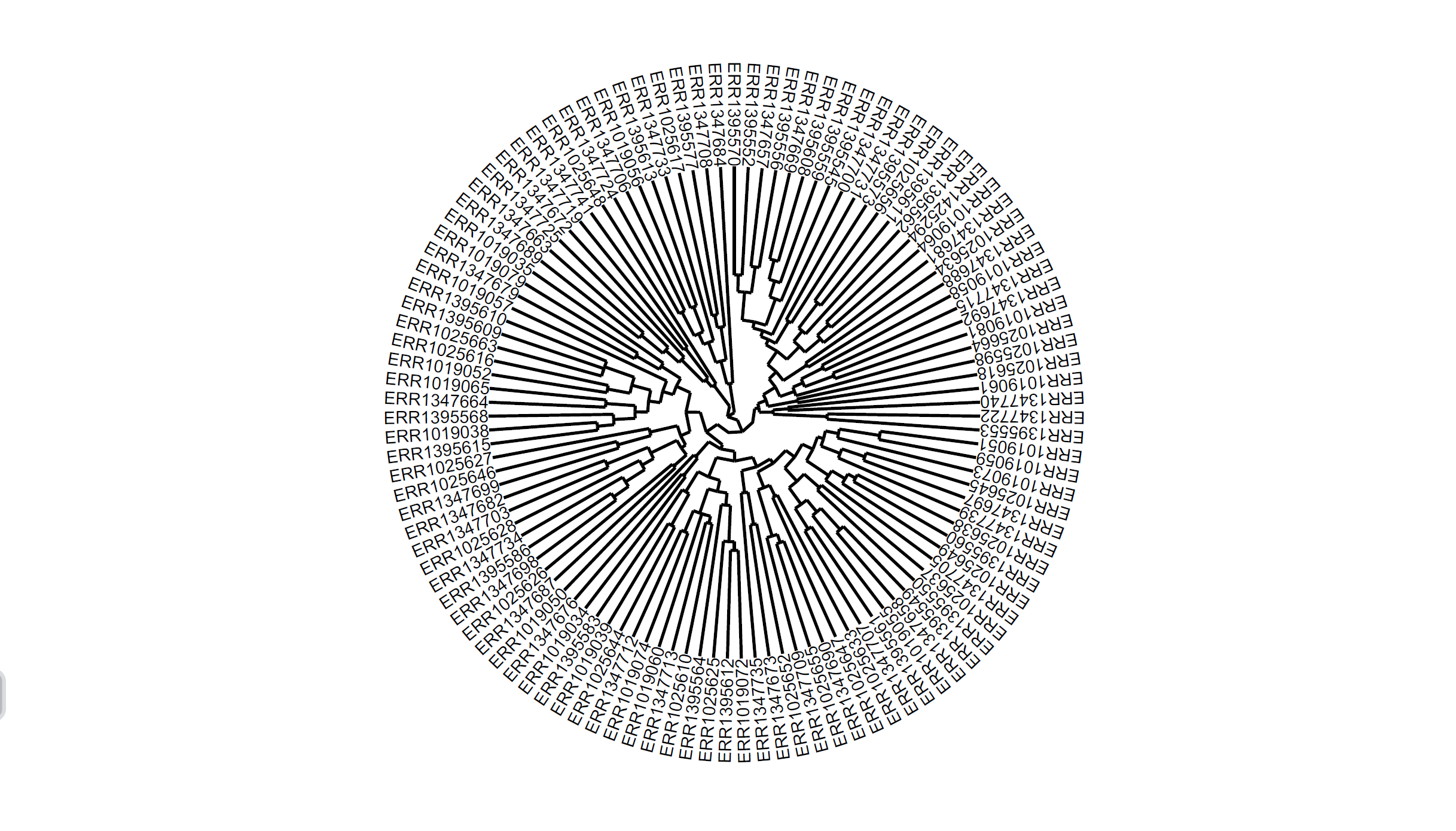


Including pheno_info will allow sample names and lines to be displayed in color.

pav_cluster(my_pav,

mult = .3,

sample_name_size = 2,

add_pheno_info = “Region”,

pheno_info_color_list = list(

Region = structure( c(“#b7514d”, “#5880ae”, “#ea9e4a”),

names = unique(pheno_info_data$Region))))


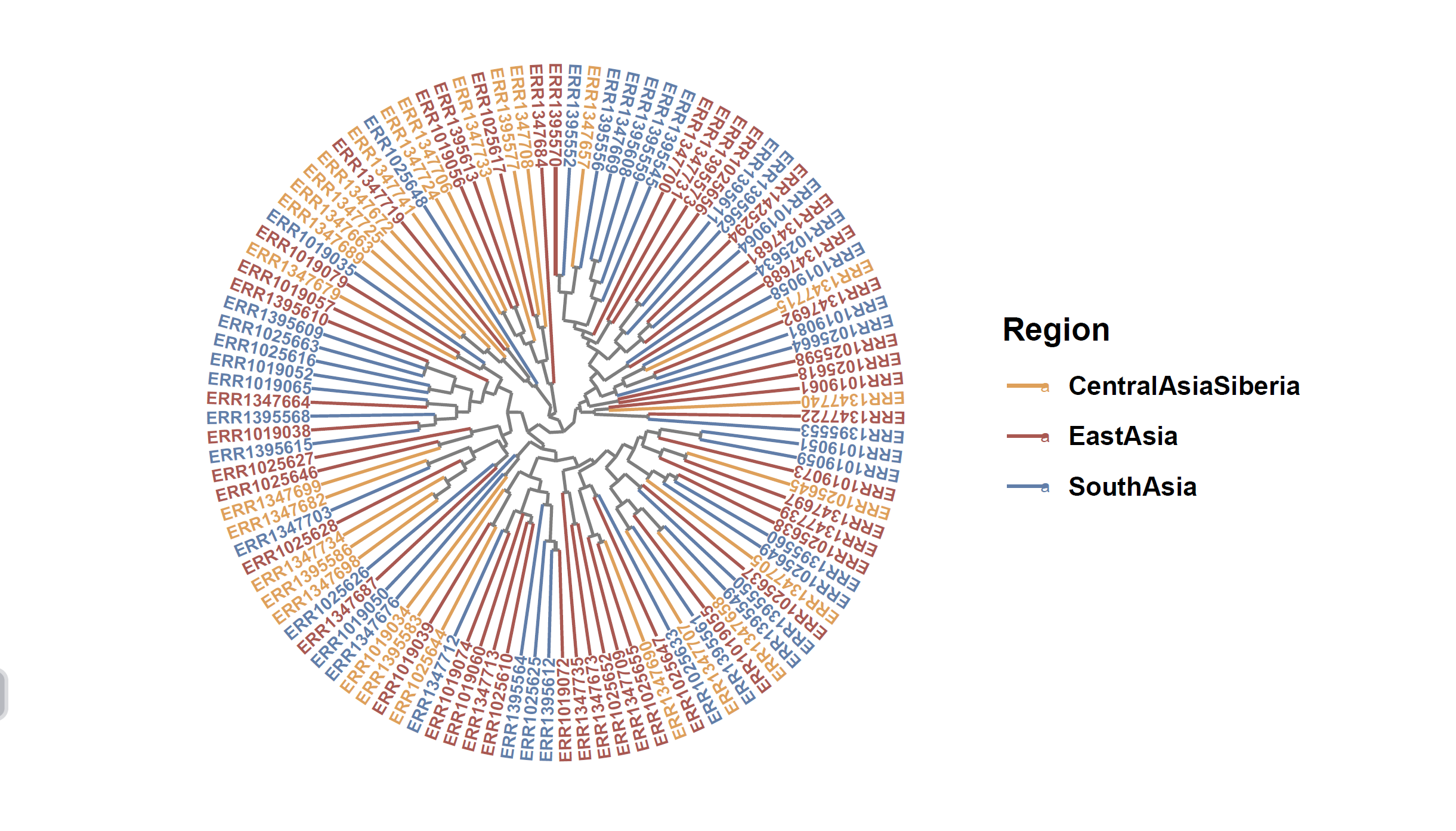


**3.6 pav_pca()**

The pav_pca() function performs Principal Component Analysis (PCA) on PAV data using the prcomp() function. The parameters center, scale, and rank will be passed to prcomp().

pav_pca(my_pav)


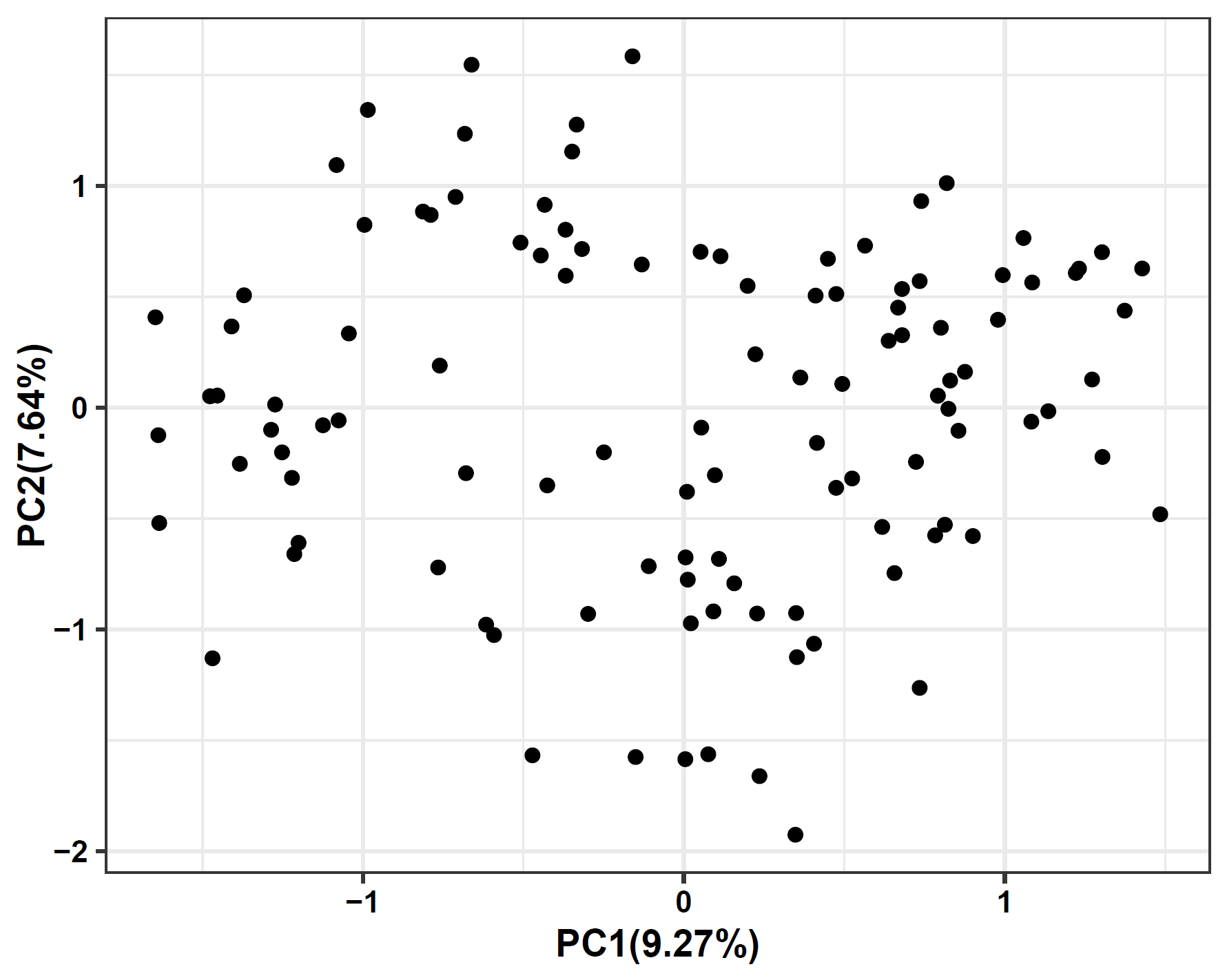


Again, if you include pheno_info, the sample points in your PCA will be displayed in color.

pav_pca(my_pav,

add_pheno_info = “Region”)


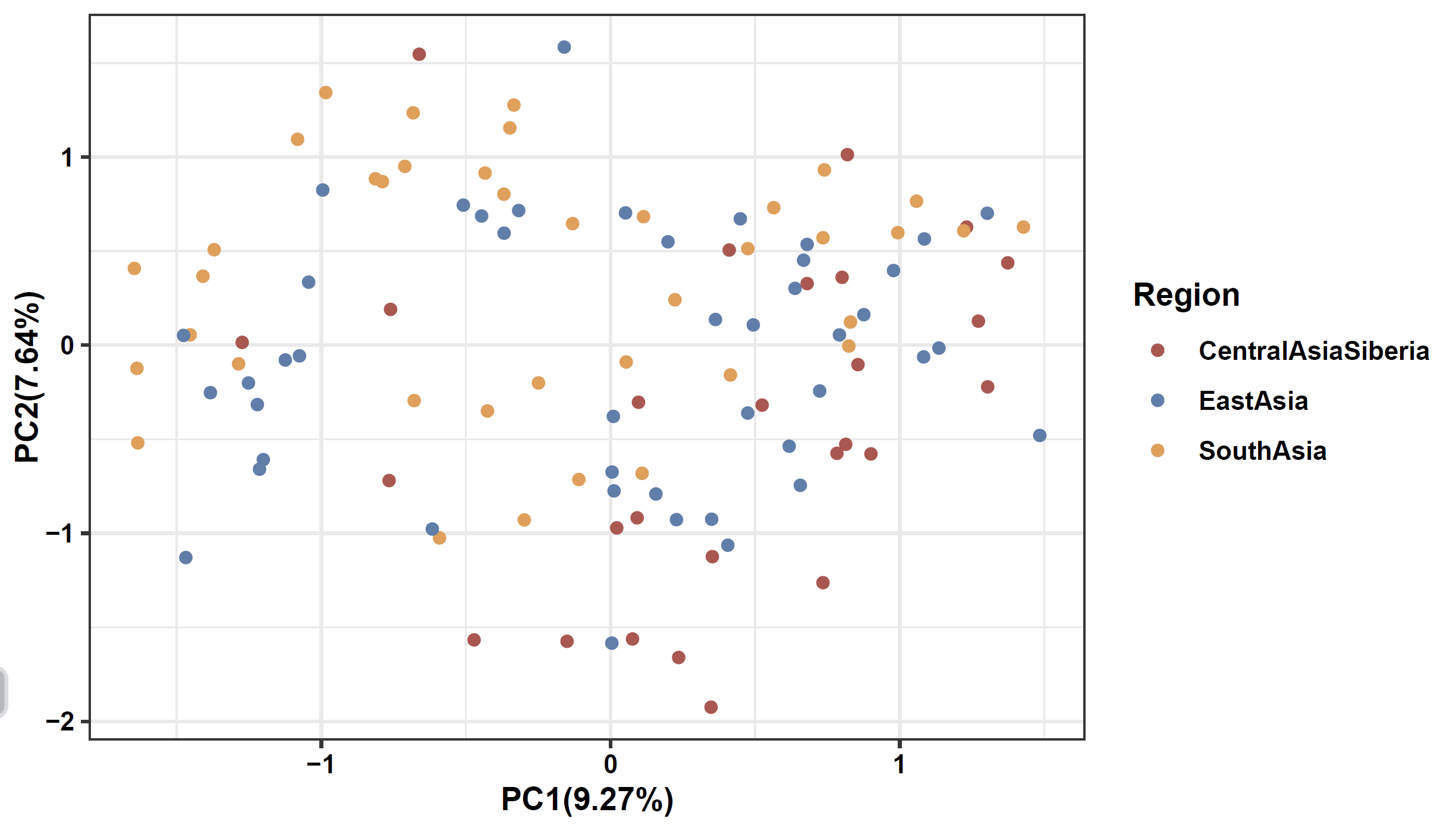


**4 Phenotype Association**

**4.1 pheno_stat()**

Phenotype association can assist researchers in understanding the potential biological functions of PAVs. For discrete values, Fisher’s exact test (fisher.test()) will be used to determine whether the distribution of each target region is uniform. For continuous values, Wilcoxon tests (wilcox.test()) will be performed.

my_pheno <- pheno_stat(my_pav,

c(“Genetic_sex”, “DNA_source”, “Region”, “Coverage_mean”),

p_adjust_method = “fdr”)

**4.2 pheno_heatmap()**

The pheno_heatmap() function visualizes the primary results of phenotype association analysis as a heatmap. It requires a PAV object and the results obtained from the pheno_stat() function. In the heatmap, rows represent regions and columns represent phenotypes. You can flip the coordinates by using the flip option. If adjust_p is set to TRUE, the adjusted p-values will be used; otherwise, the p-values will be displayed. Regions with at least one p-value or adjusted p-value below the specified threshold, p_threshold, will be included in the heatmap. The color scheme for the p-values and adjusted p-values is defined by the cov_colors parameter.

pheno_heatmap(my_pav,

my_pheno,

cell_border_color = “white”,

na_col = “gray90”,

flip = T,

adjust_p = F,

p_threshold = 0.1,

column_names_size = 7)


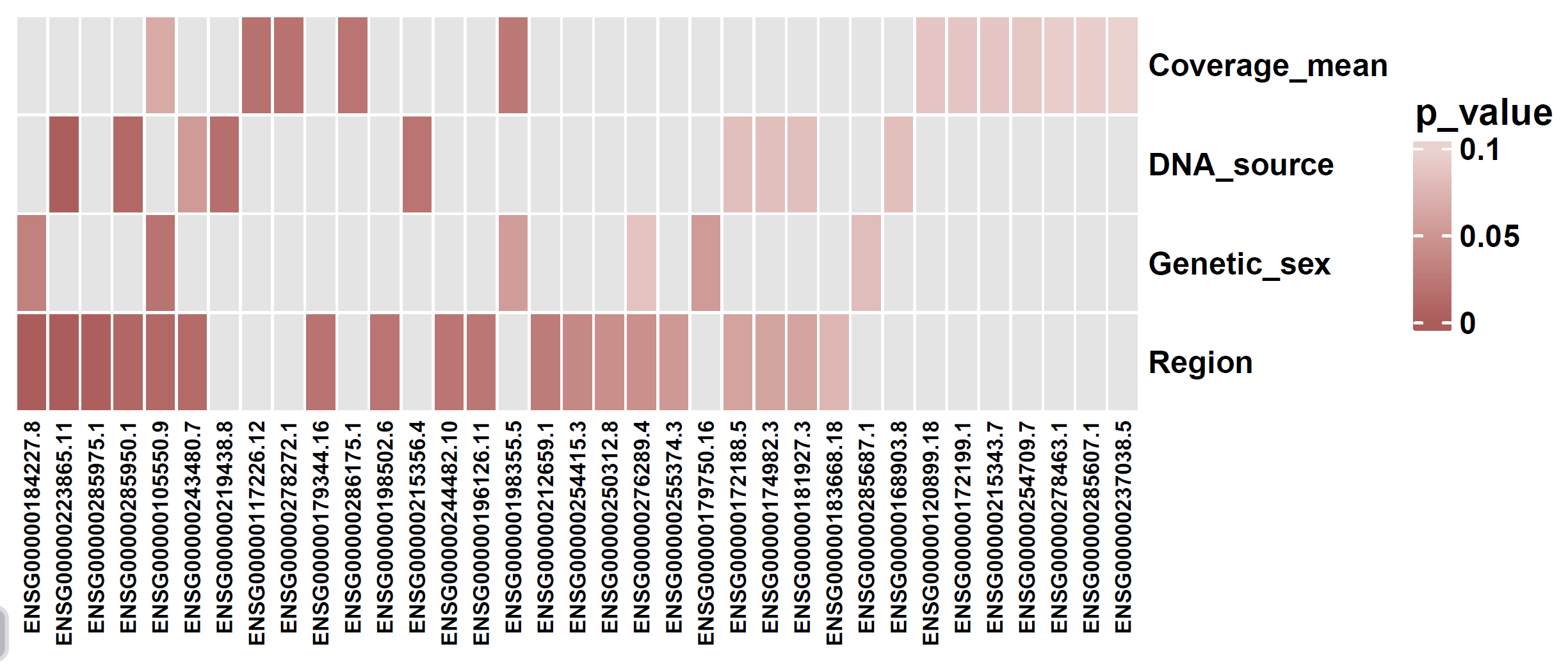


By default, only the significant cells will be highlighted, while the color of other cells is set to na_col. You can override this setting by changing only_show_significant to FALSE.

pheno_heatmap(my_pav,

my_pheno,

cell_border_color = “white”,

na_col = “gray90”,

flip = T,

adjust_p = F,

p_threshold = 0.1,

column_names_size = 7,

only_show_significant = FALSE)


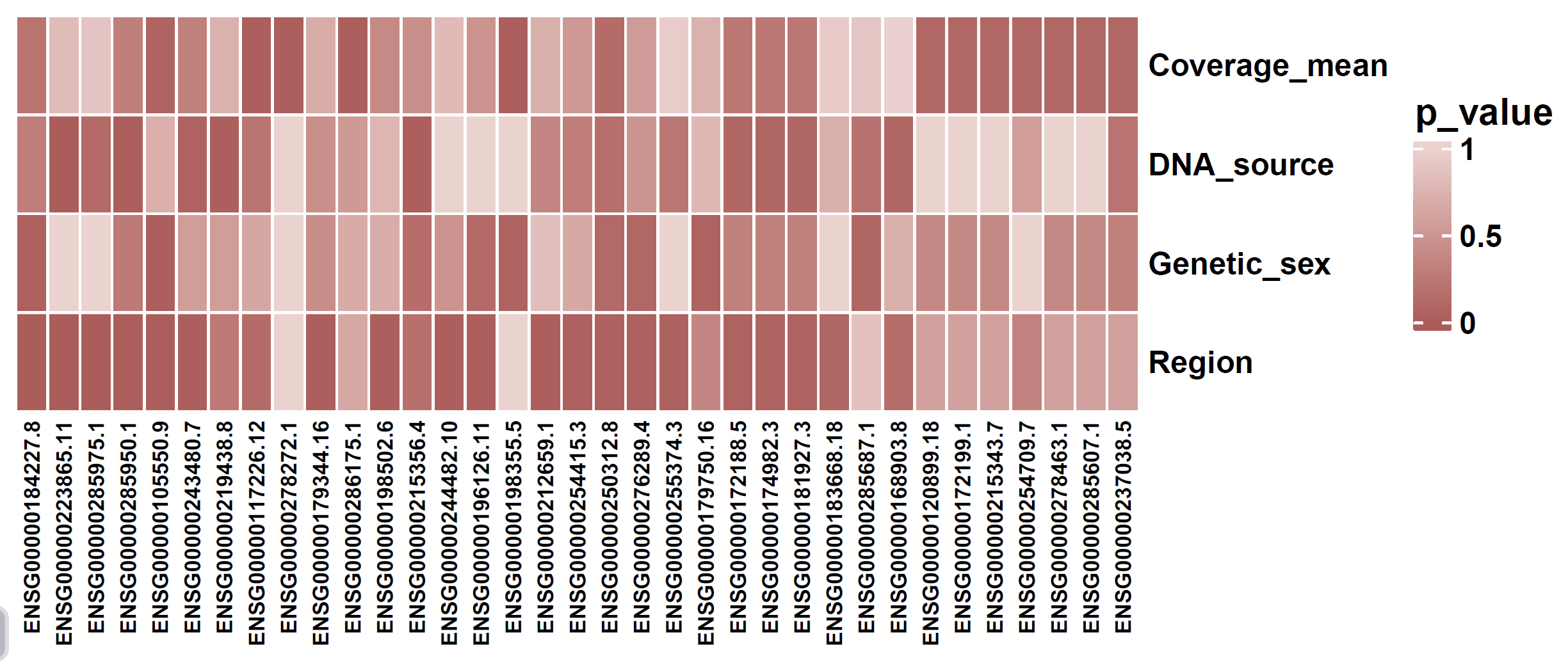


The region_info can also be integrated into your figure as needed.

pheno_heatmap(my_pav,

my_pheno,

cell_border_color = “white”,

na_col = “gray90”,

flip = T,

adjust_p = F,

p_threshold = 0.1,

column_names_size = 7,

add_region_info = c(“chr”, “length”),

anno_param_region = list(name_rot = 0))


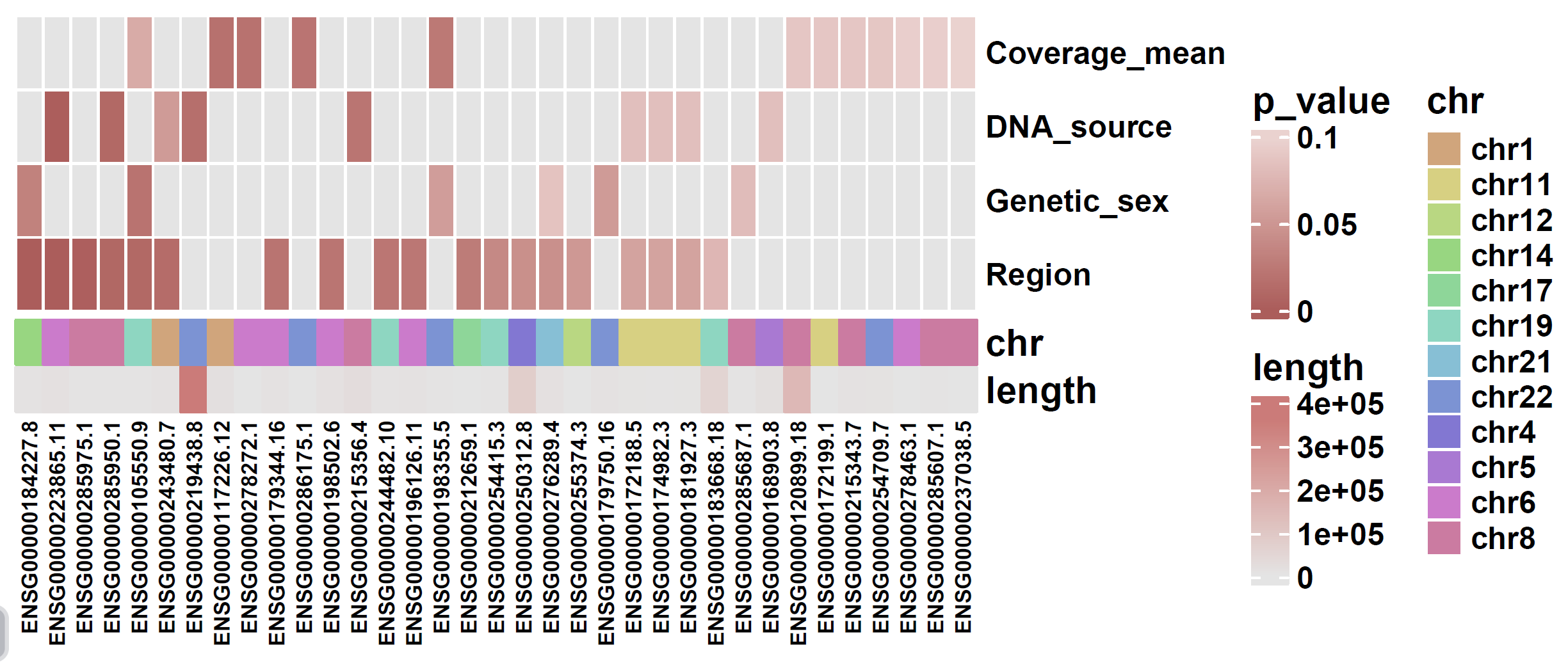


**4.3 pheno_manhattan()**

If you want to investigate a phenotype further, you can create a Manhattan plot using the pheno_manhattan() function. This requires the chromosomes and positions to be included in the region_info of the PAV object. You can select the p-value and adjusted p-value by using the adjust_p parameter. The most significant n regions will be highlighted and labeled.

pheno_manhattan(my_pav, my_pheno, “Region”, “chr_n”, “start”,

highlight_top_n = 10,

highlight_text_size = 2,

x_text_size = 5)


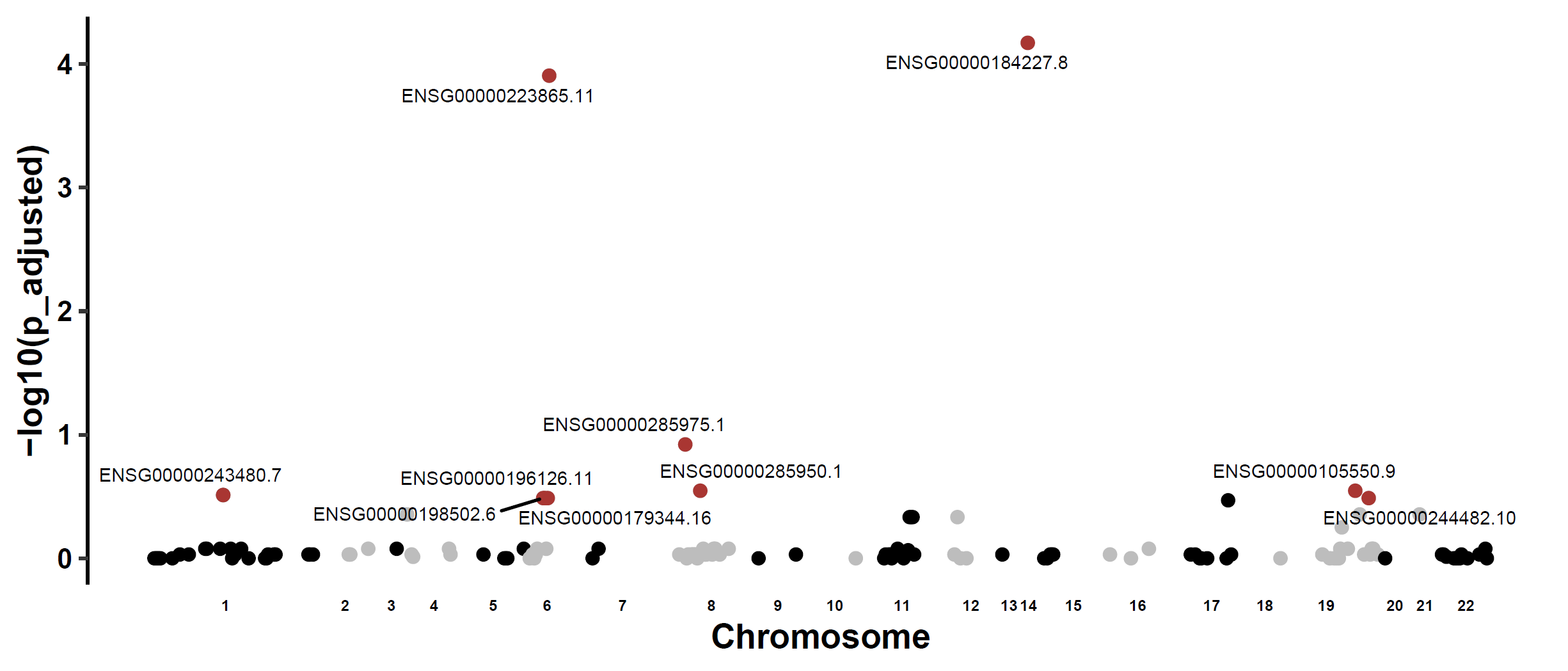


**4.4 pheno_block()**

If the phenotype is represented by discrete values, the pheno_block() function can be used to observe the percentage of individuals possessing the regions within each group. The number in brackets indicates the sample size for each group.

pheno_block(my_pav, my_pheno, “Region”,

adjust_p = F,

p_threshold = .1,

row_names_size = 6,

cell_border_color = “black”)


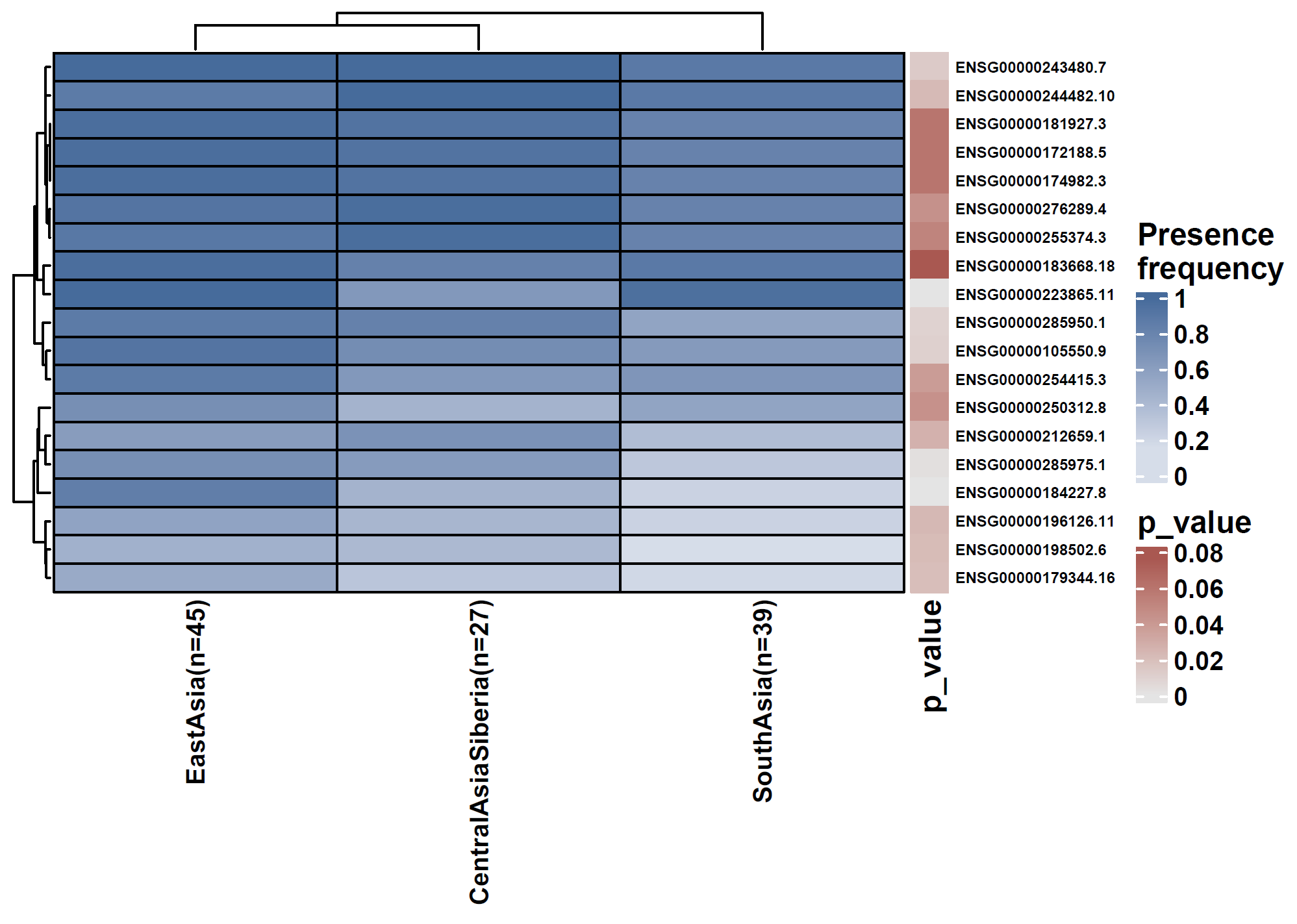


By default, the p-value and adjusted p-value are displayed in an annotation. If you would like to view additional information in region_info, you can include it using the add_region_info parameter.

pheno_block(my_pav, my_pheno, “Region”,

adjust_p = F,

p_threshold = .1,

row_names_size = 6,

cell_border_color = “black”,

add_region_info = c(“p”, “chr”))


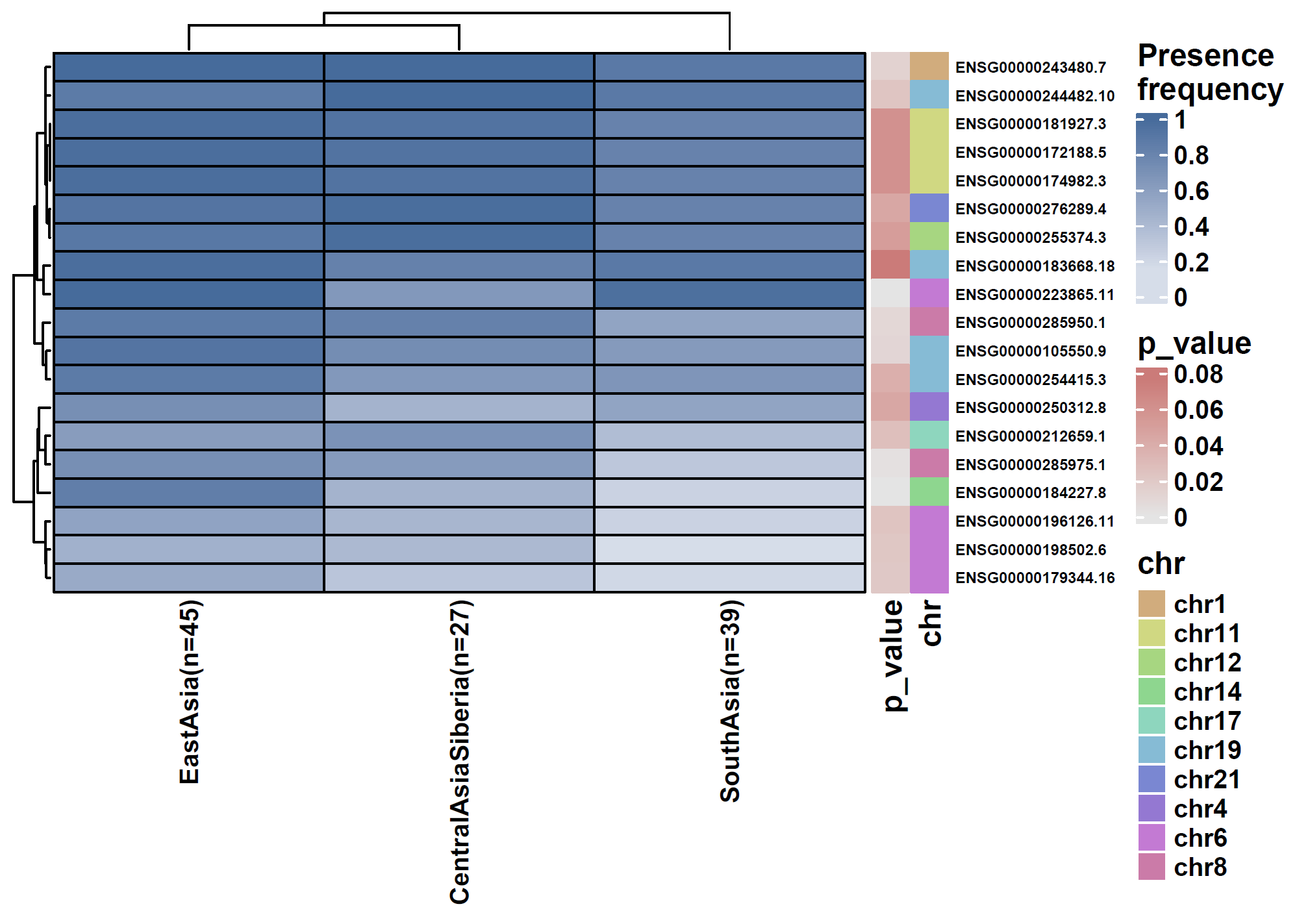


**4.5 pheno_bar() and pheno_violin()**

The pheno_bar() function illustrates the relationship between a specific genomic region and a phenotype for discrete values, while the pheno_violin() function is intended for continuous values.

pheno_bar(my_pav, “Region”, “ENSG00000226430.6”)


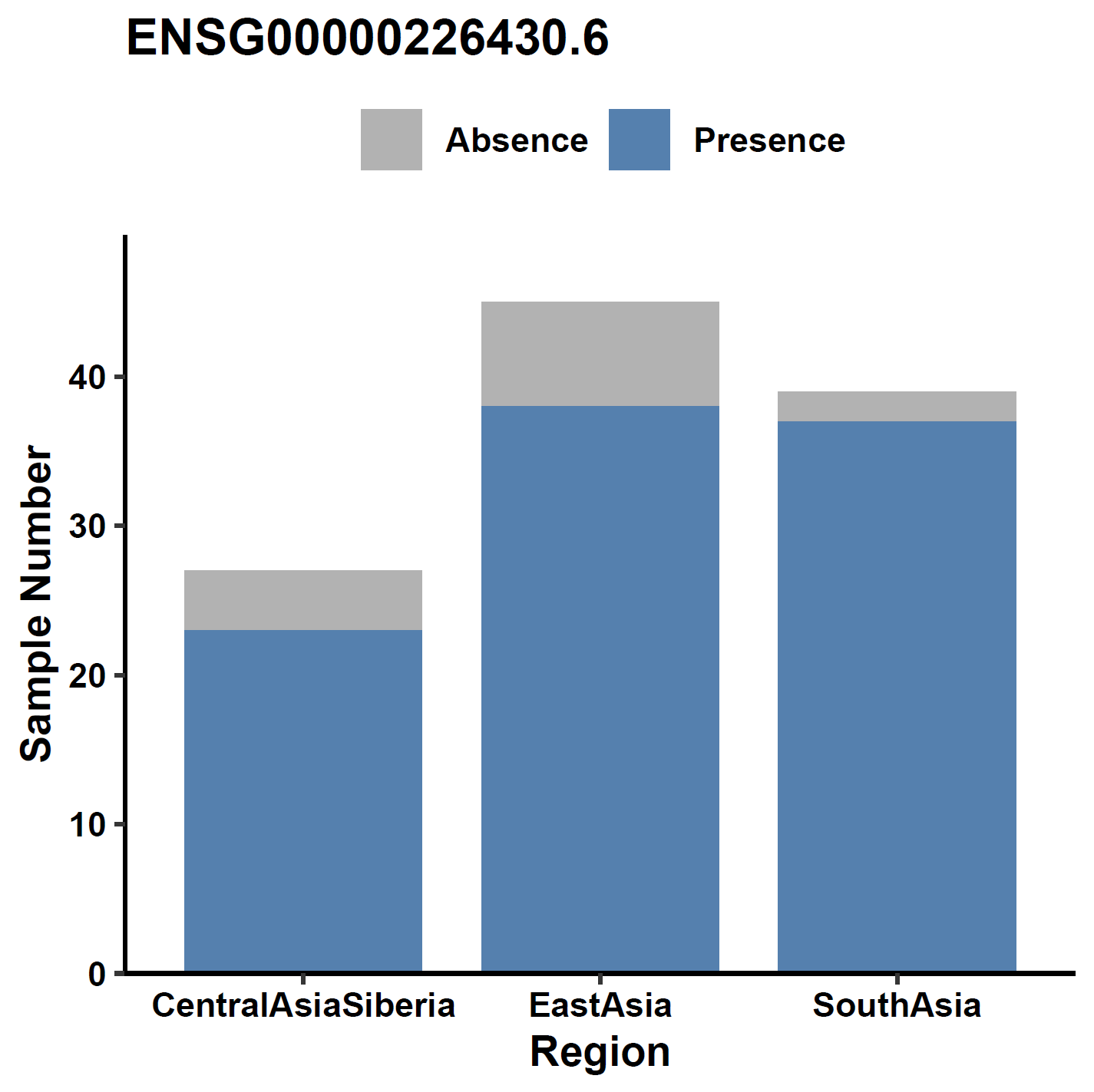


pheno_violin(my_pav, “Coverage_mean”, “ENSG00000226430.6”)


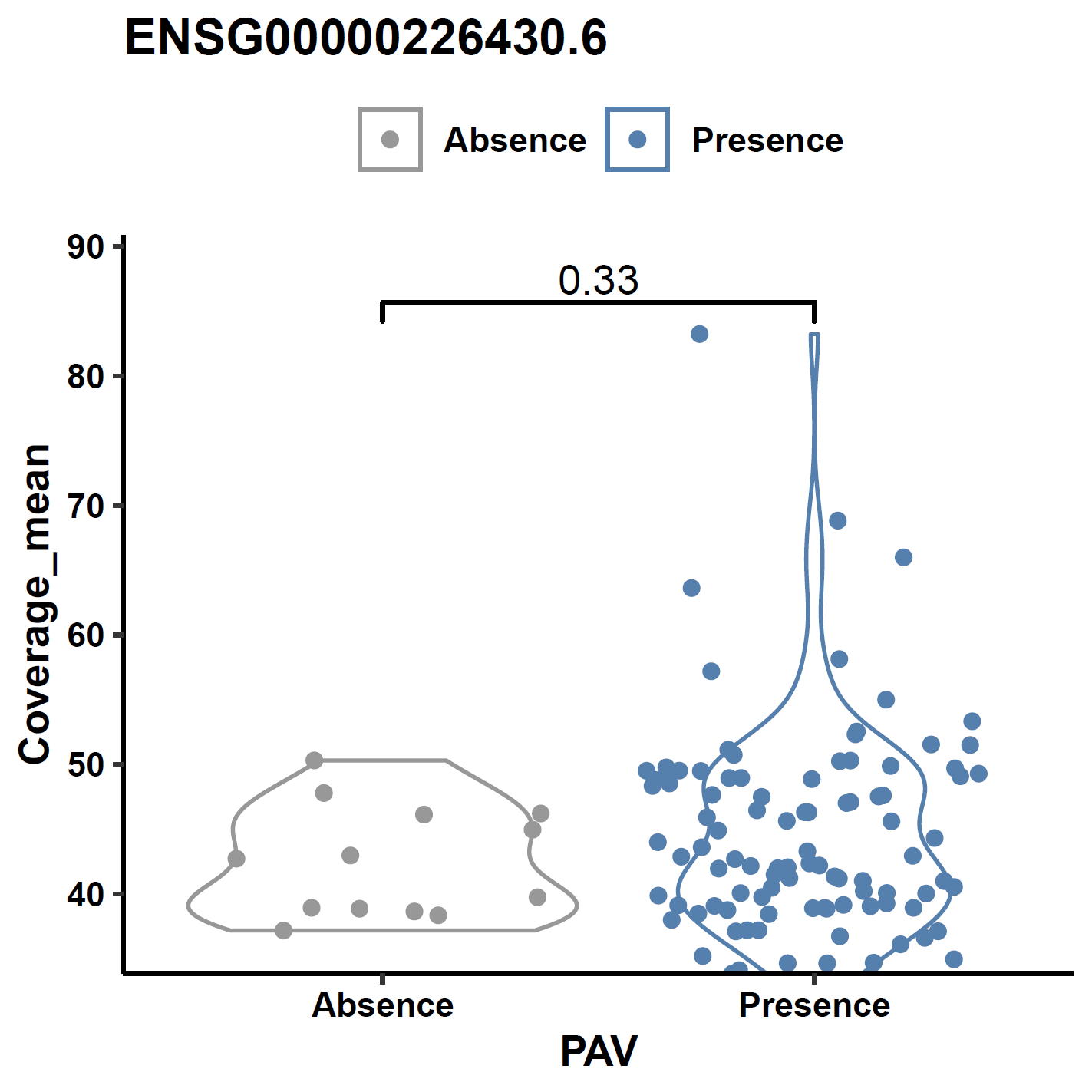


Several result charts can be displayed at the same time.

p <- lapply(sample(my_pav@region$name[my_pav@region$type == “Distributed”], 4), **function**(x){

pheno_bar(my_pav, “Region”, x, legend_title_size = 9, legend_text_size = 8)

})

library(patchwork)

*#> Warning: 程序包'patchwork'是用R版本4.4.1 来建造的*

p[[1]] + p[[2]] + p[[3]] + p[[4]]


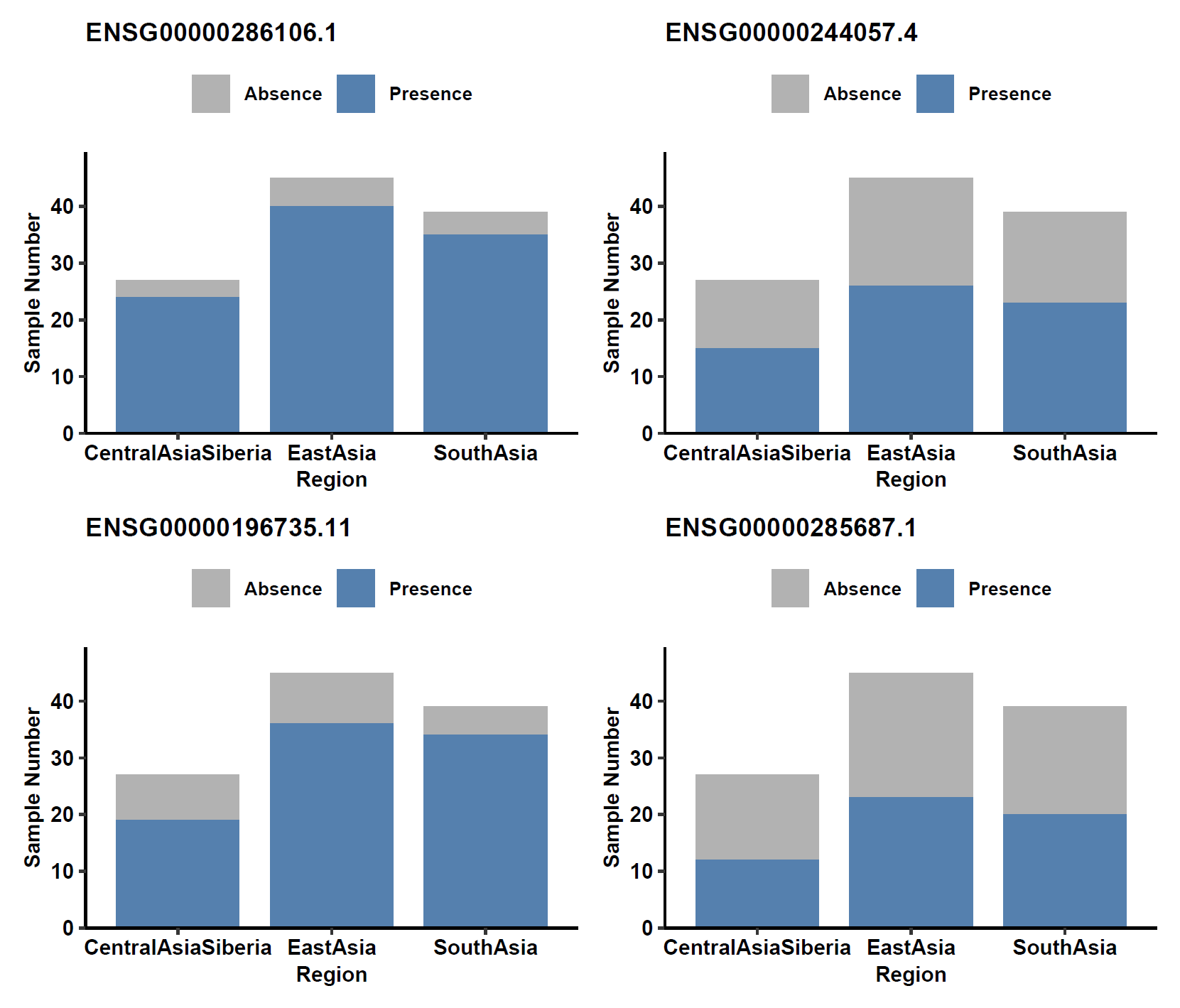


p <- lapply(sample(my_pav@region$name[my_pav@region$type == “Distributed”], 4), **function**(x){

pheno_violin(my_pav, “Coverage_mean”, x, legend_title_size = 9, legend_text_size = 8)

})

p[[1]] + p[[2]] + p[[3]] + p[[4]]


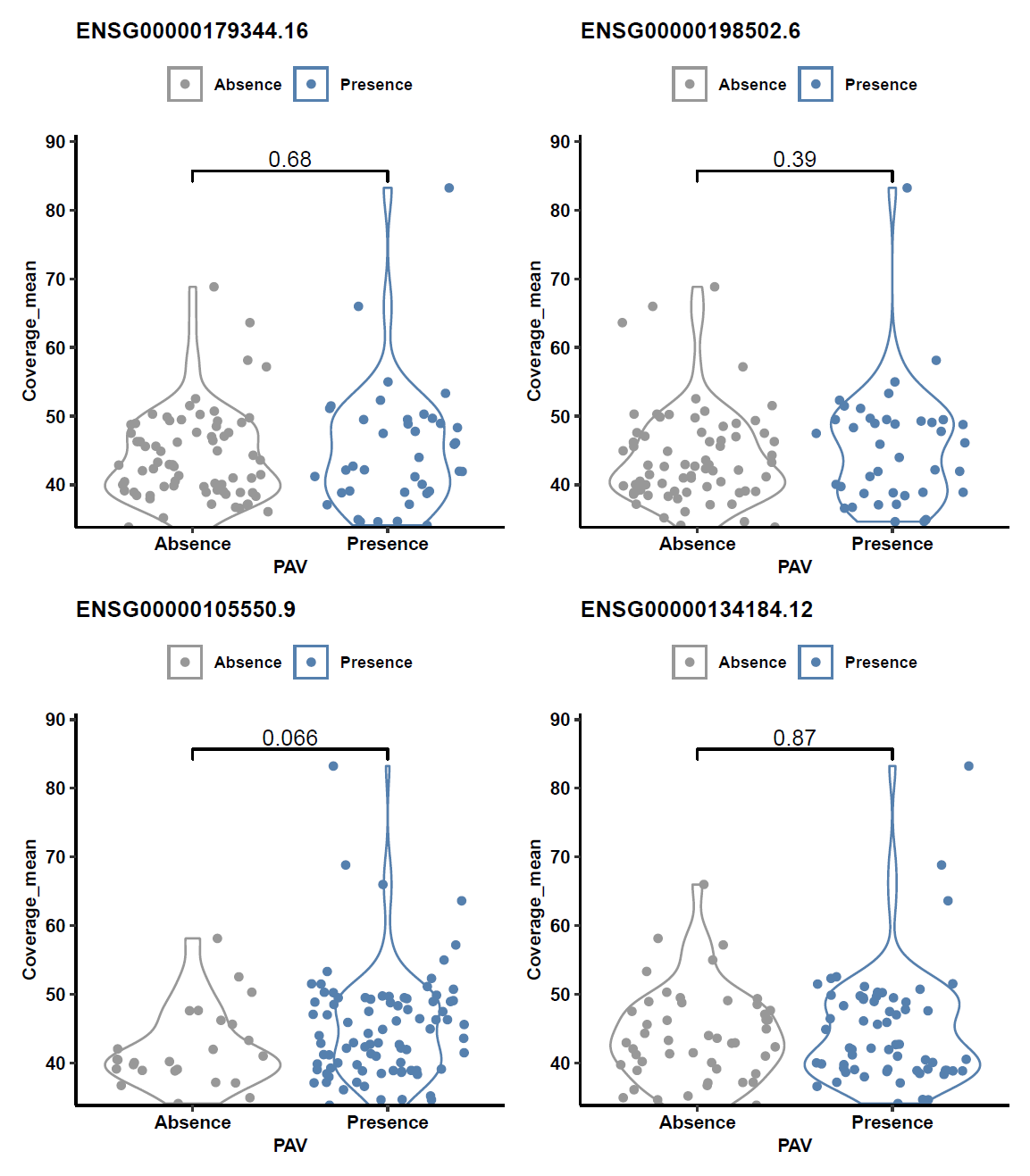


**5 Estimation**

**5.1 plot_size()**

A simulation can effectively estimate genome size, and this can be achieved through the APAV “*pavSize*” tool, which generates required input data. The plot_size() function can plot the growth curve of the estimation results. It offers three distinct chart types: “jitter,” “ribbon,” and “errorbar,” allowing for versatile visual representations of the data.

data(“est_res”)

plot_size(est_res, y_title = “Gene Number”)


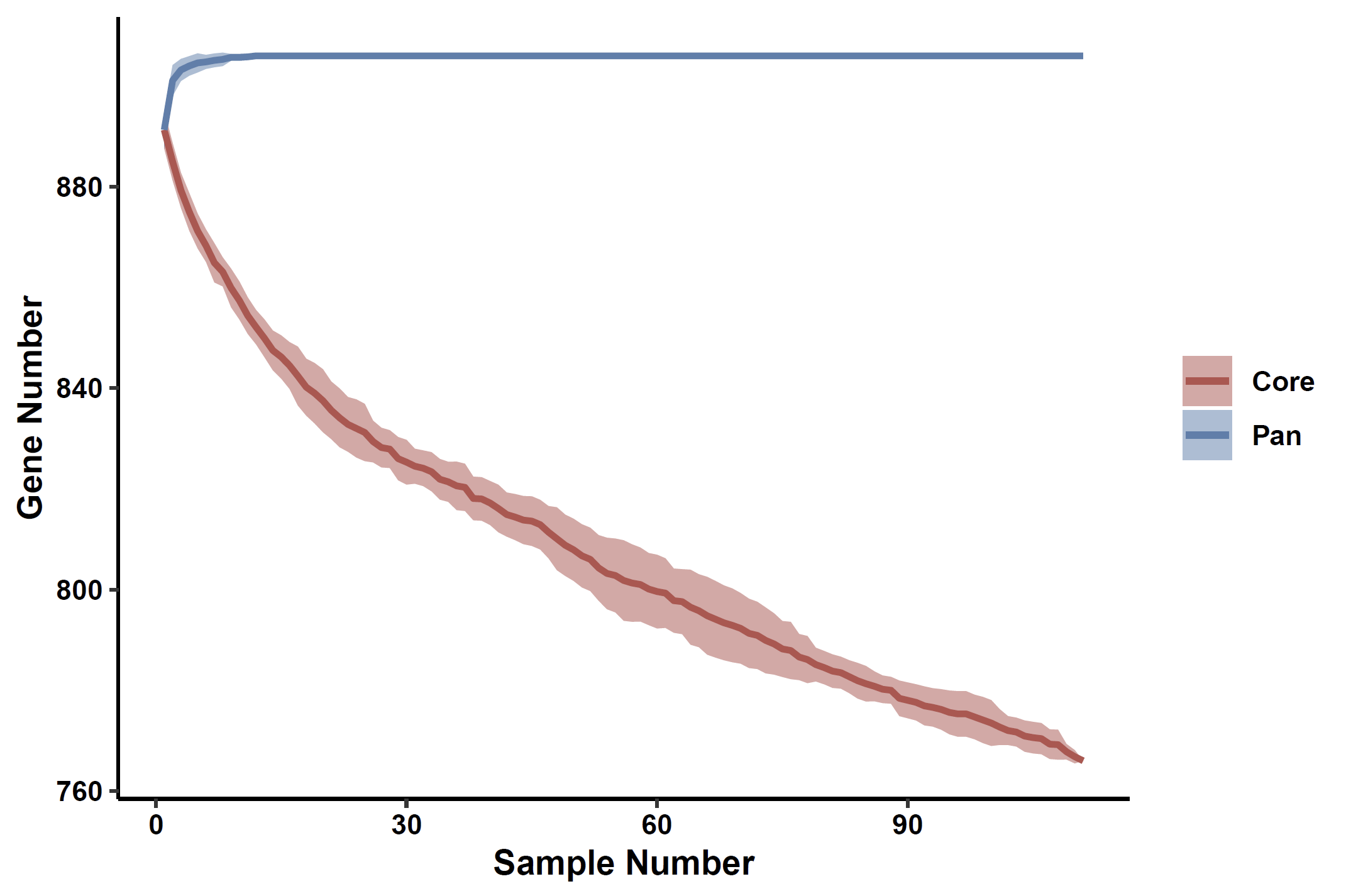


plot_size(est_res, chart_type = “jitter”, y_title = “Gene Number”)


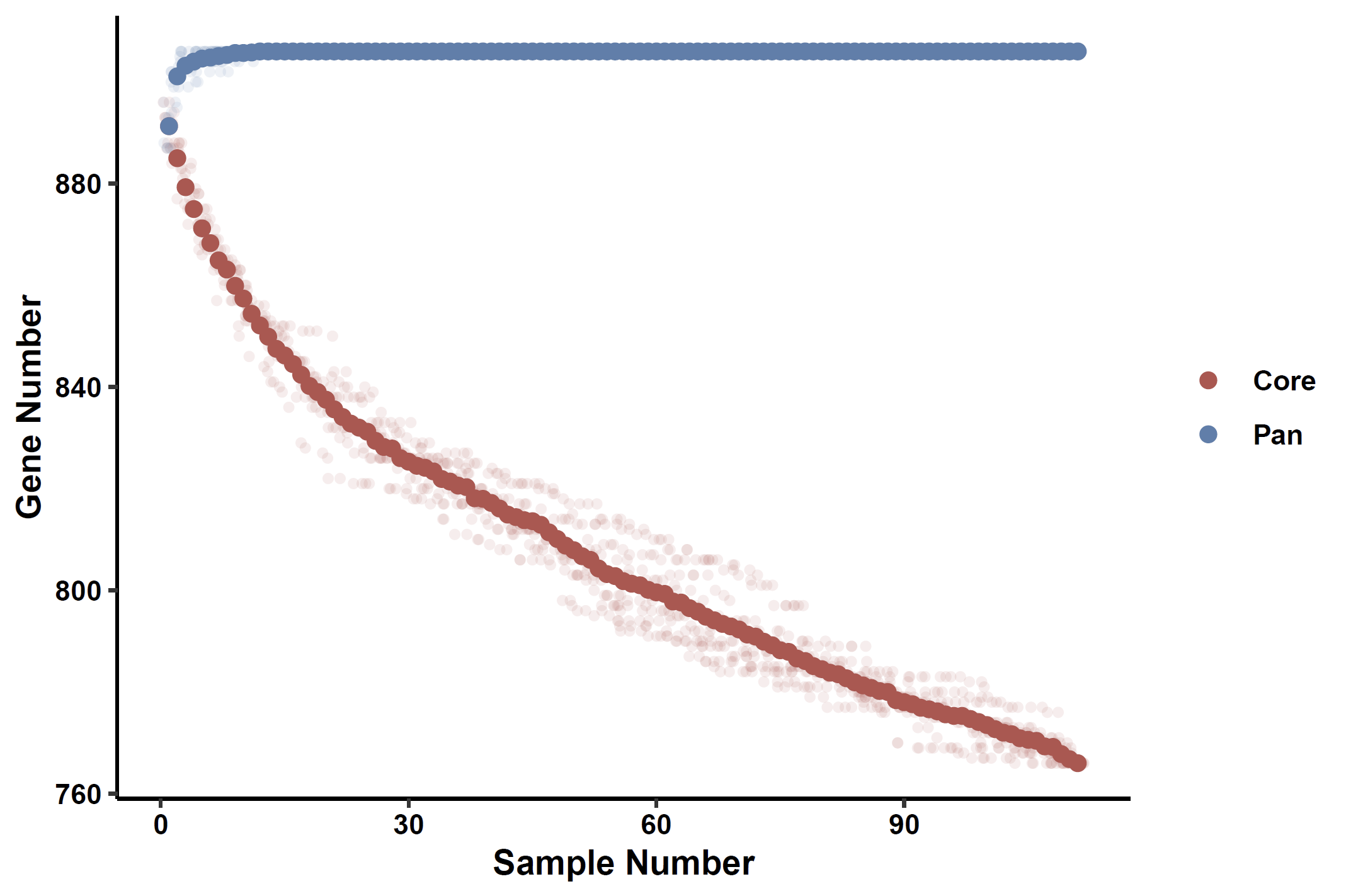


plot_size(est_res, chart_type = “errorbar”, y_title = “Gene Number”)


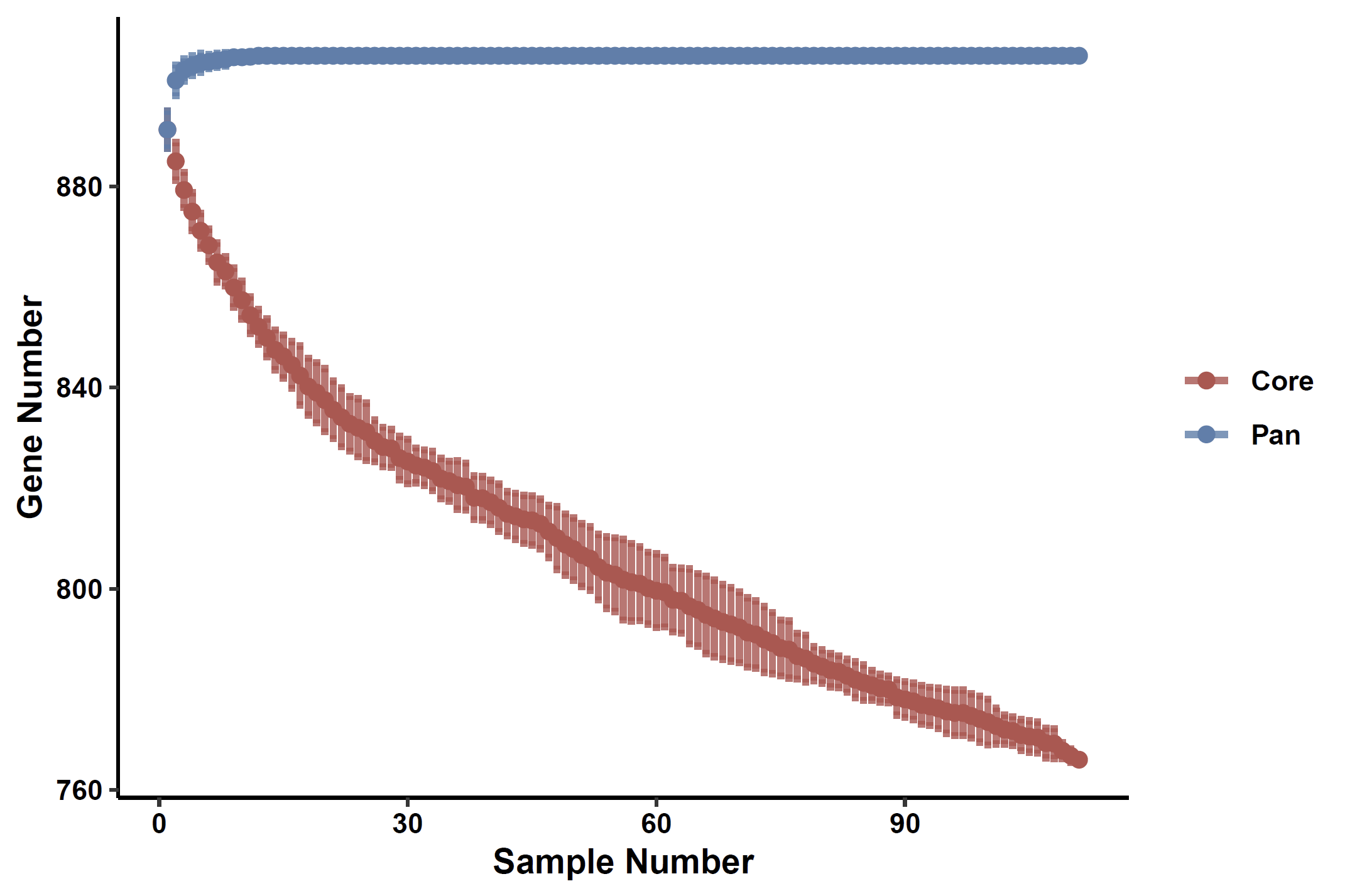


Moreover, you have the option to plot increasing values alongside these charts.

plot_size(est_res, data_type = “increasing”,

y_title = “Number of the increasing gene”)


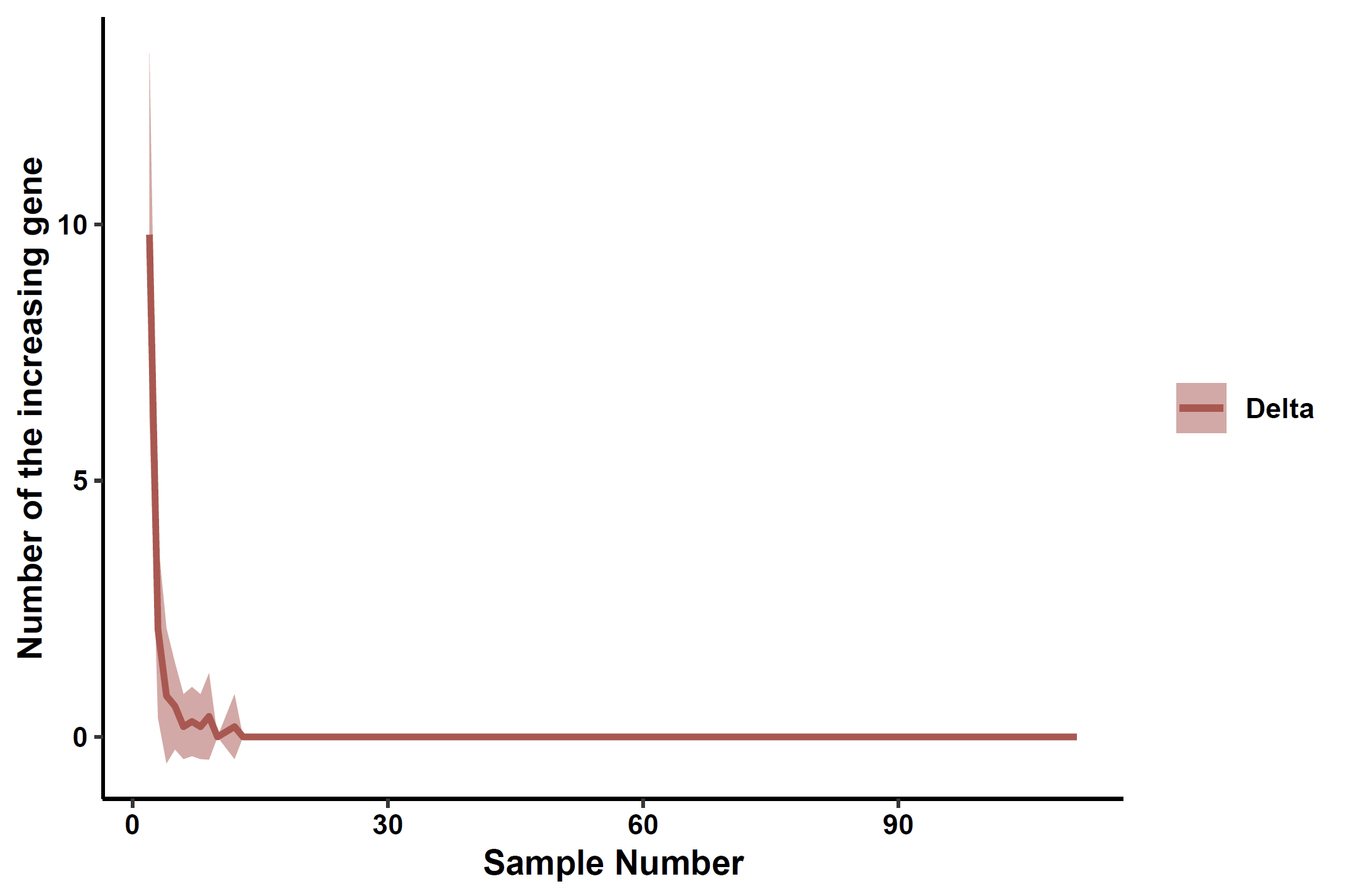


plot_size(est_res, data_type = “increasing”, chart_type = “jitter”,

y_title = “Number of the increasing gene”)


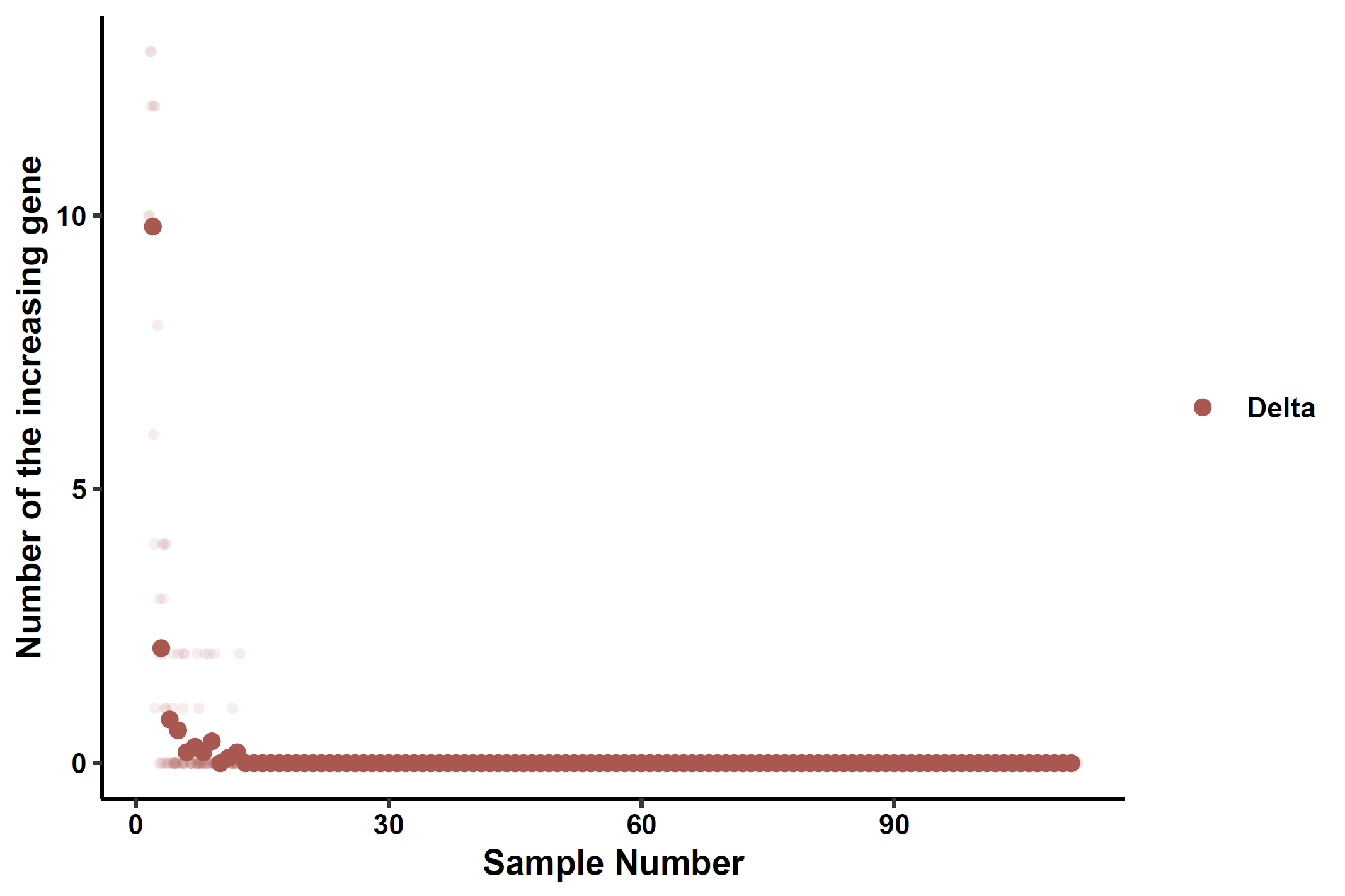


plot_size(est_res, data_type = “increasing”, chart_type = “errorbar”,

y_title = “Number of the increasing gene”)


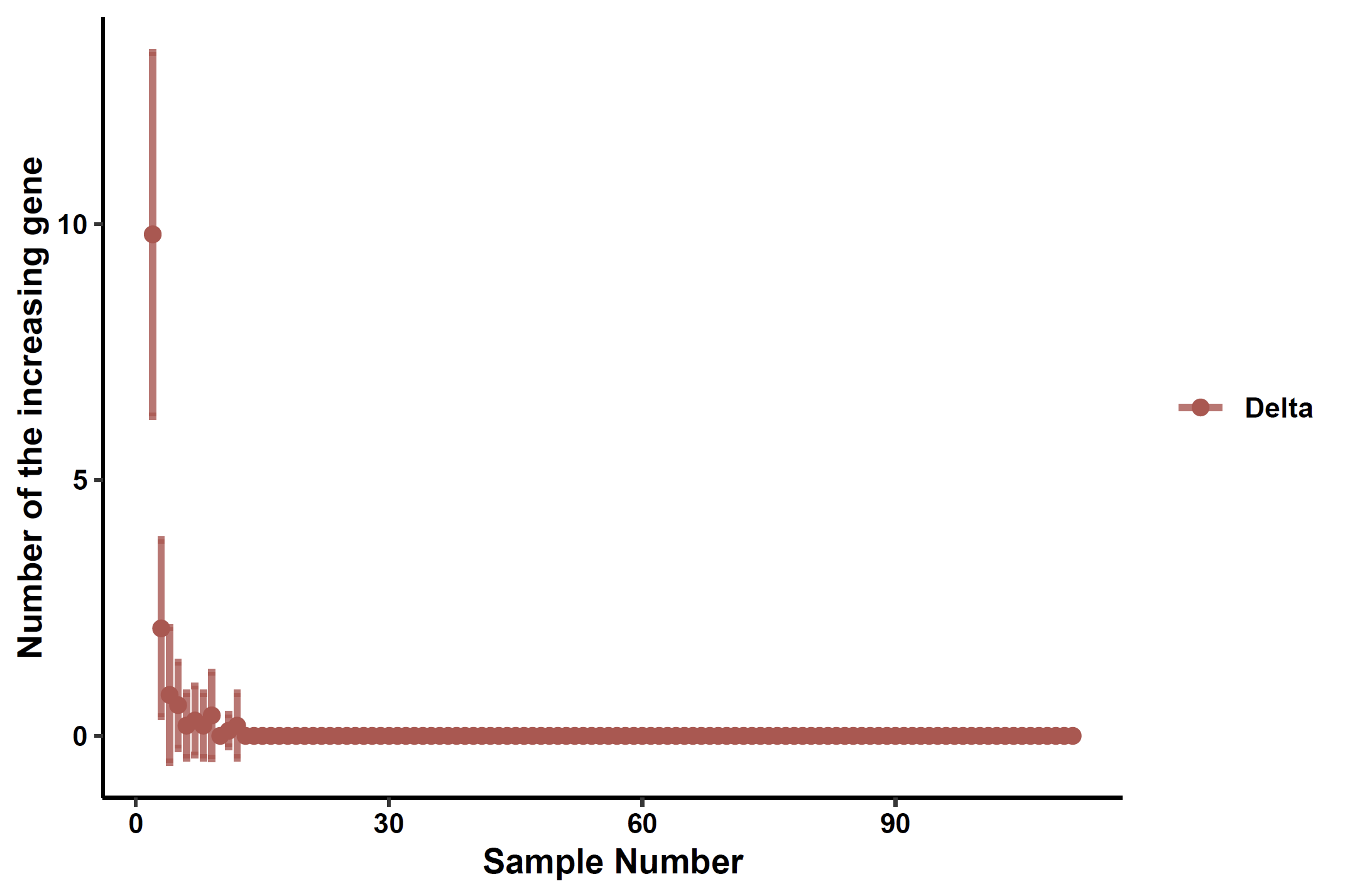


Each chart type is fully customizable, enabling adjustments to color, size, and transparency to suit specific presentation needs.

plot_size(est_res,

chart_type = “ribbon”,

path_size = .5,

path_color = c(Pan = “black”, Core = “black”),

ribbon_fill = c(Pan = “#e38e28”, Core = “#298022”),

ribbon_alpha = .7)


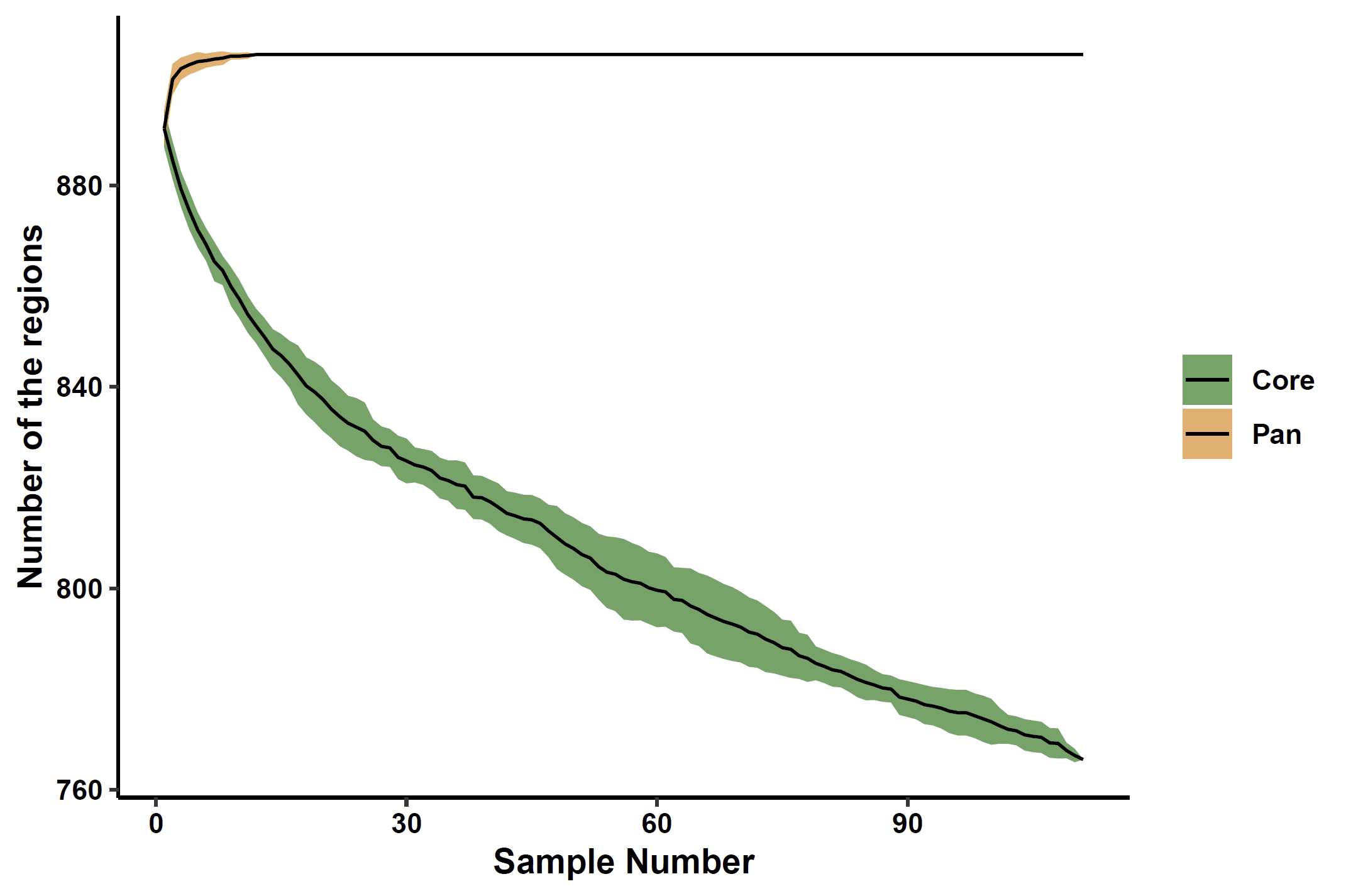


plot_size(est_res,

chart_type = “errorbar”,

errorbar_width = .1,

errorbar_size = 1,

errorbar_color = c(Pan = “#e38e28”, Core = “#298022”),

errorbar_alpha = .4,

errorbar_point_size = 1,

errorbar_point_color = c(Pan = “#e38e28”, Core = “#298022”))


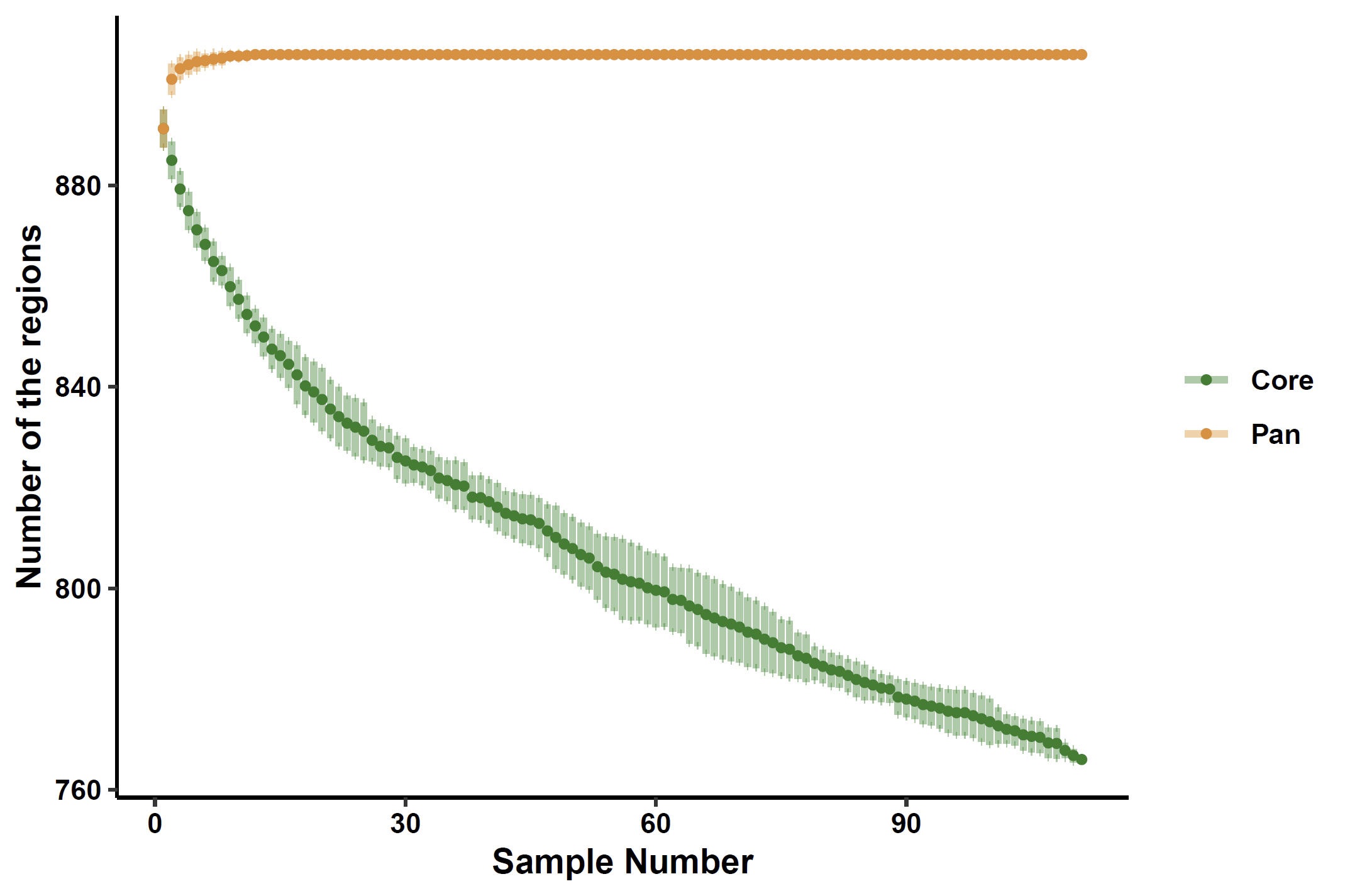


plot_size(est_res,

chart_type = “jitter”,

jitter_width = .1,

jitter_size = 1,

jitter_color = c(Pan = “#e38e28”, Core = “#298022”),

jitter_alpha = .2,

jitter_point_size = 1,

jitter_point_color = c(Pan = “#e38e28”, Core = “#298022”))


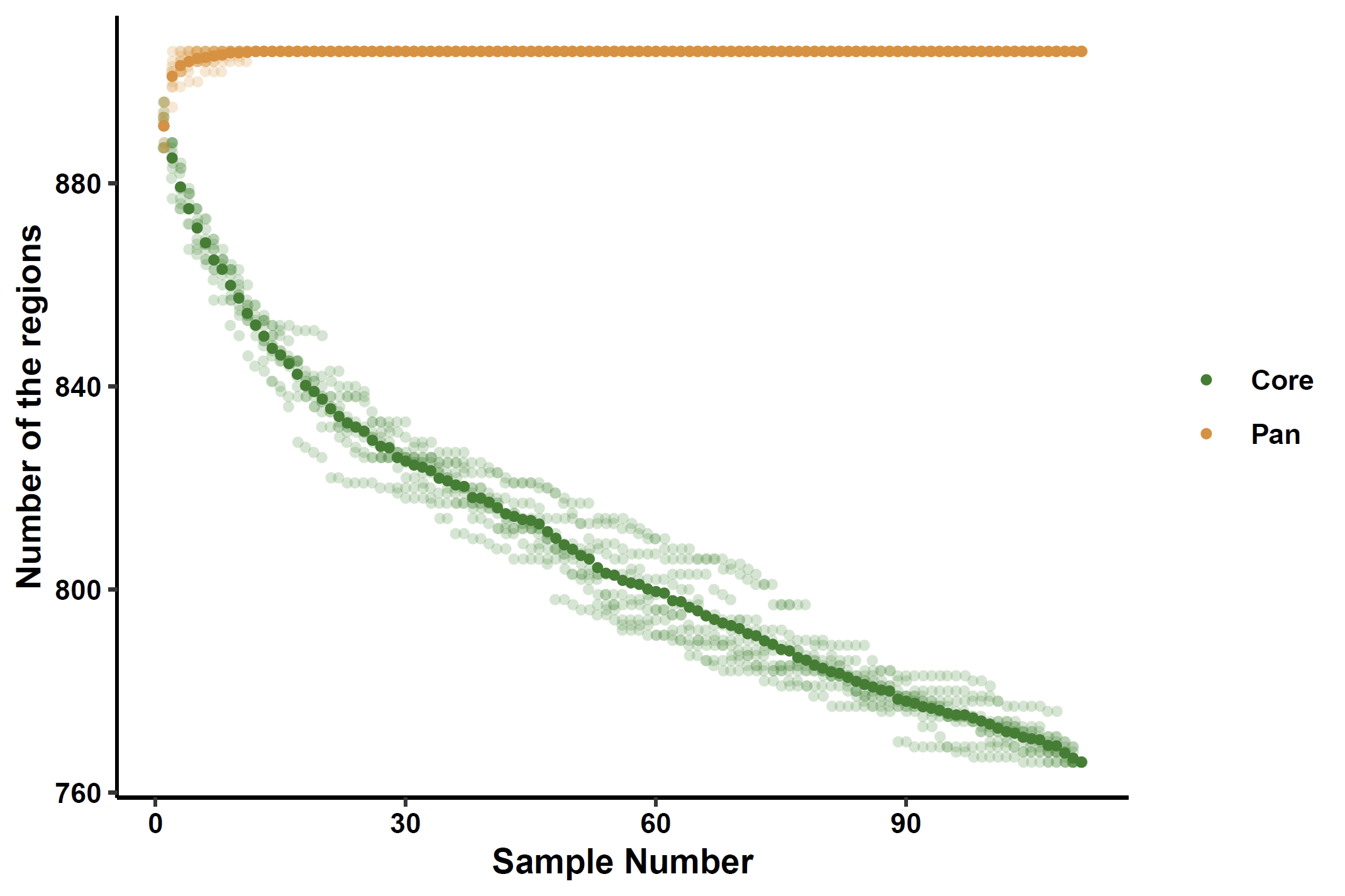


When working with grouped samples, the plot_size() function can be employed to create individualized growth curves for each group, facilitating a more nuanced analysis.

data(“est_group_res”)

plot_size(est_group_res,

path_color = c(CentralAsiaSiberia = “#4d9242”),

ribbon_fill = c(CentralAsiaSiberia = “gray”))


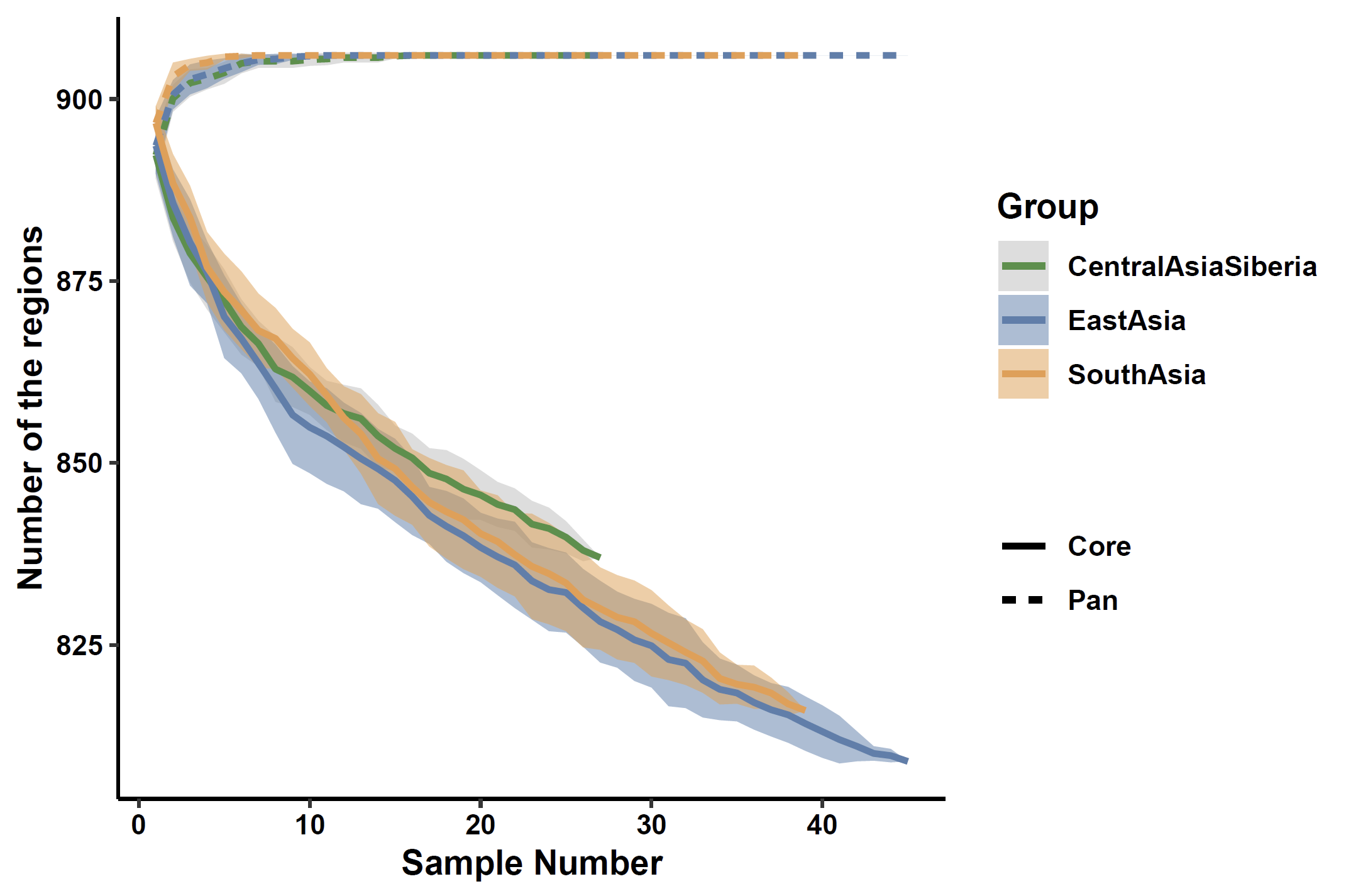


plot_size(est_group_res,

chart_type = “jitter”,

jitter_alpha = .3,

jitter_point_color = c(CentralAsiaSiberia = “#4d9242”),

jitter_color = c(CentralAsiaSiberia = “gray”))


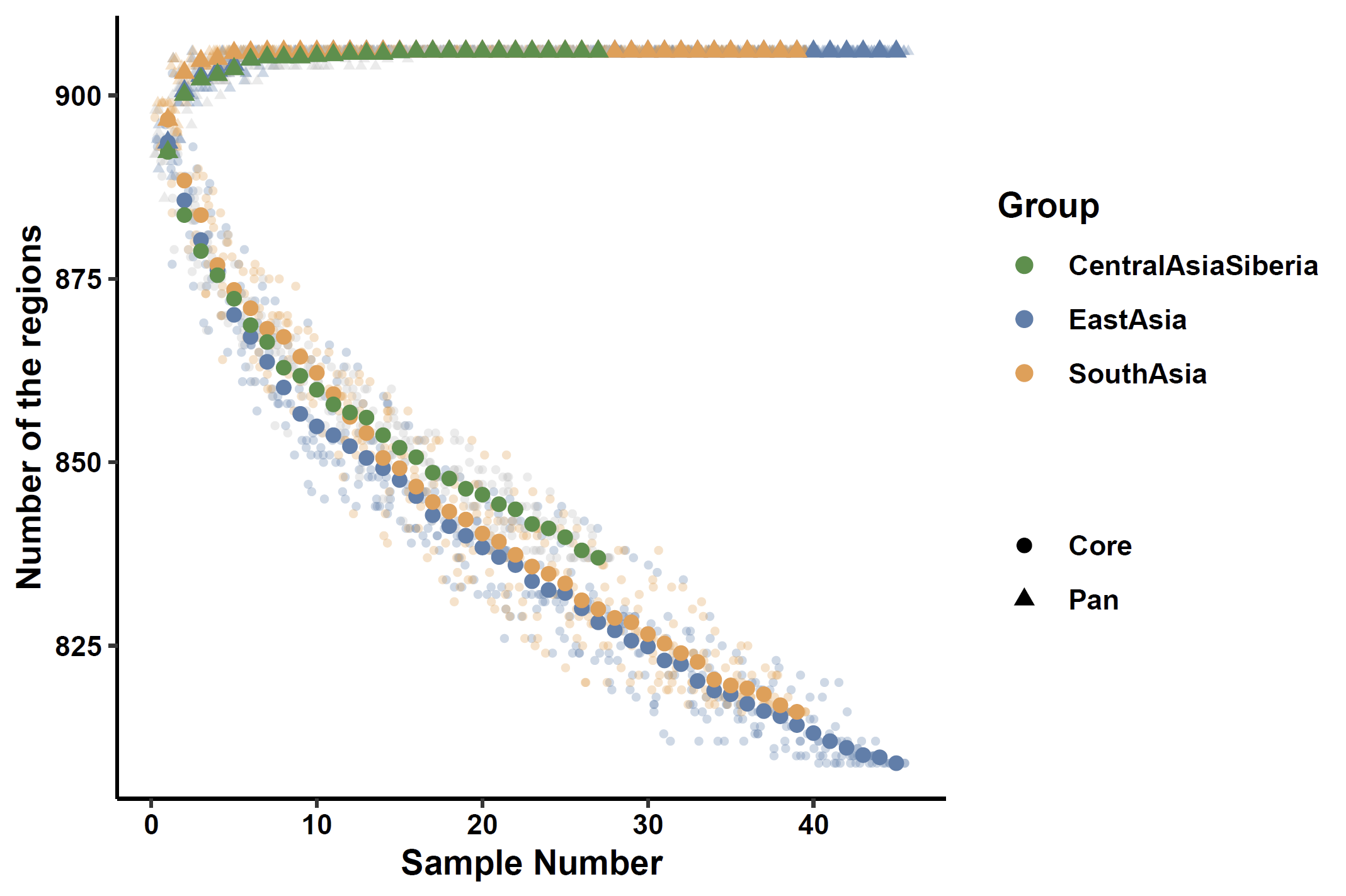


plot_size(est_group_res,

chart_type = “errorbar”,

errorbar_point_color = c(CentralAsiaSiberia = “#4d9242”),

errorbar_color = c(CentralAsiaSiberia = “gray”))


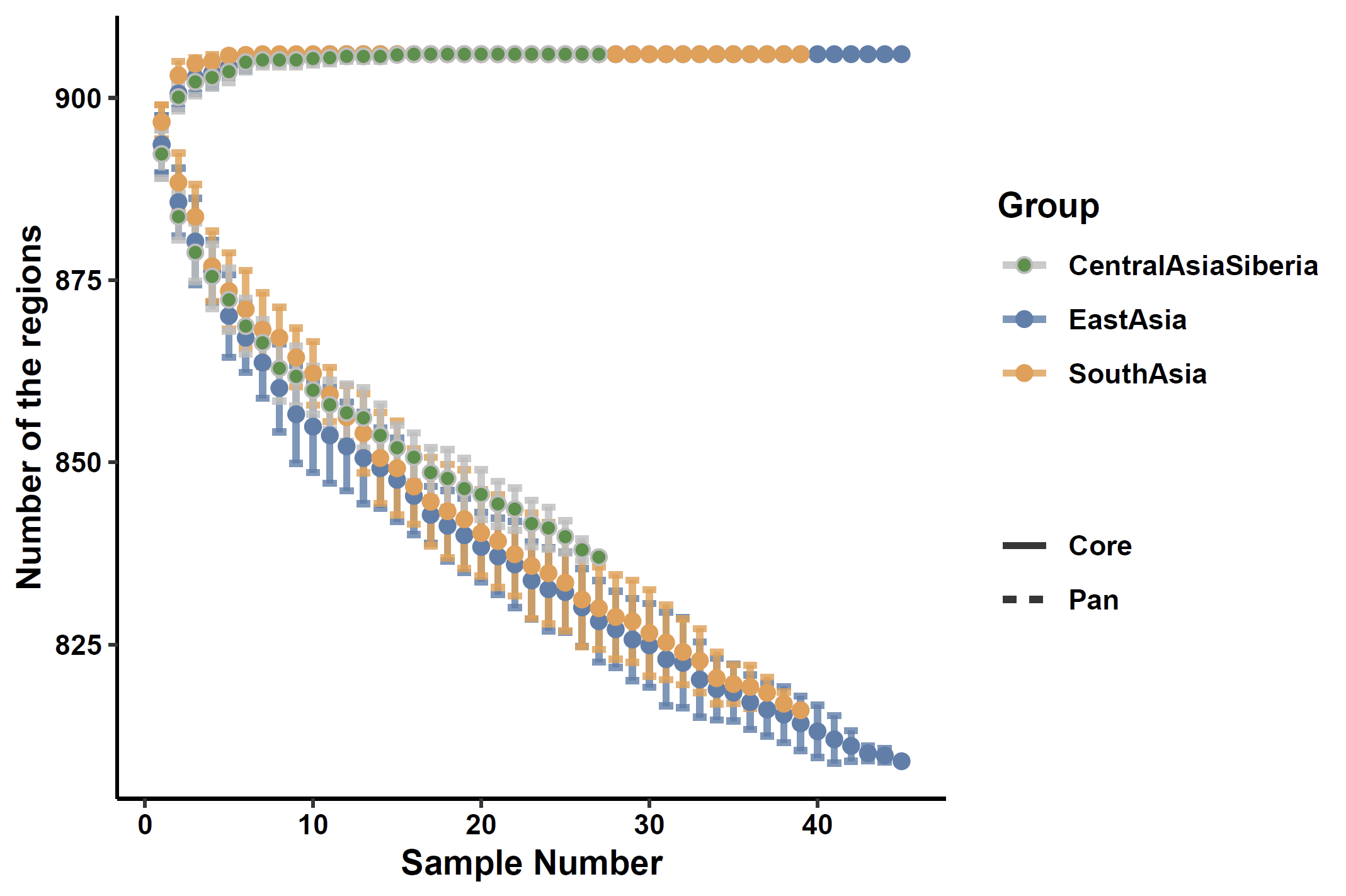


**6 Visualization of elements**

To visualize the absence of elements in a target region, three effective functions are available: plot_ele_cov() for displaying the coverage of elements, plot_ele_pav() for showcasing the PAV of elements, and plot_ele_depth() for illustrating the depth of elements.

data(“ele_cov”)

data(“ele_pav”)

data(“ele_depth”)

data(“ele_gff”)

data(“ele_pheno”)

plot_ele_pav(ele_pav)


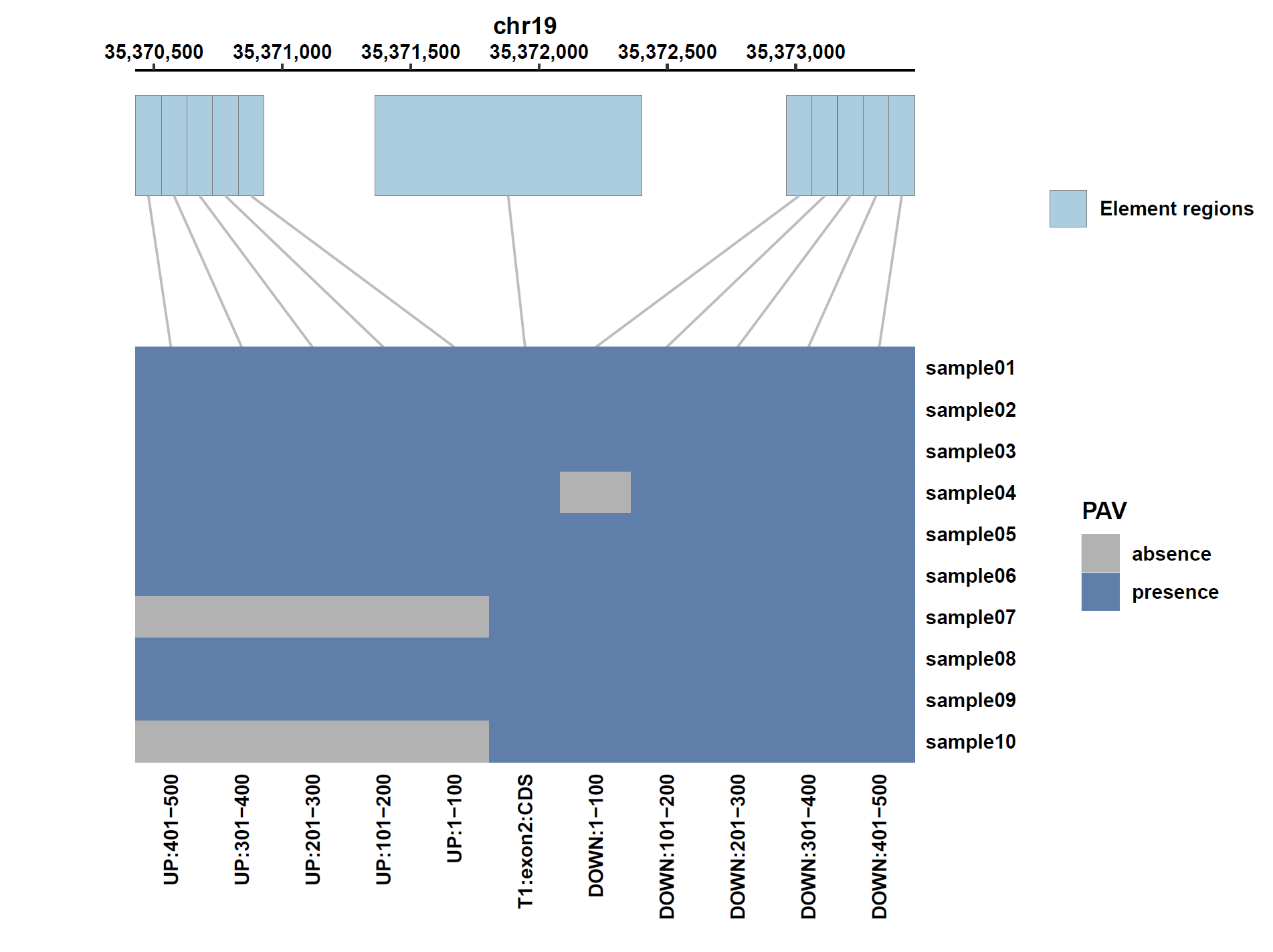


plot_ele_cov(ele_cov)


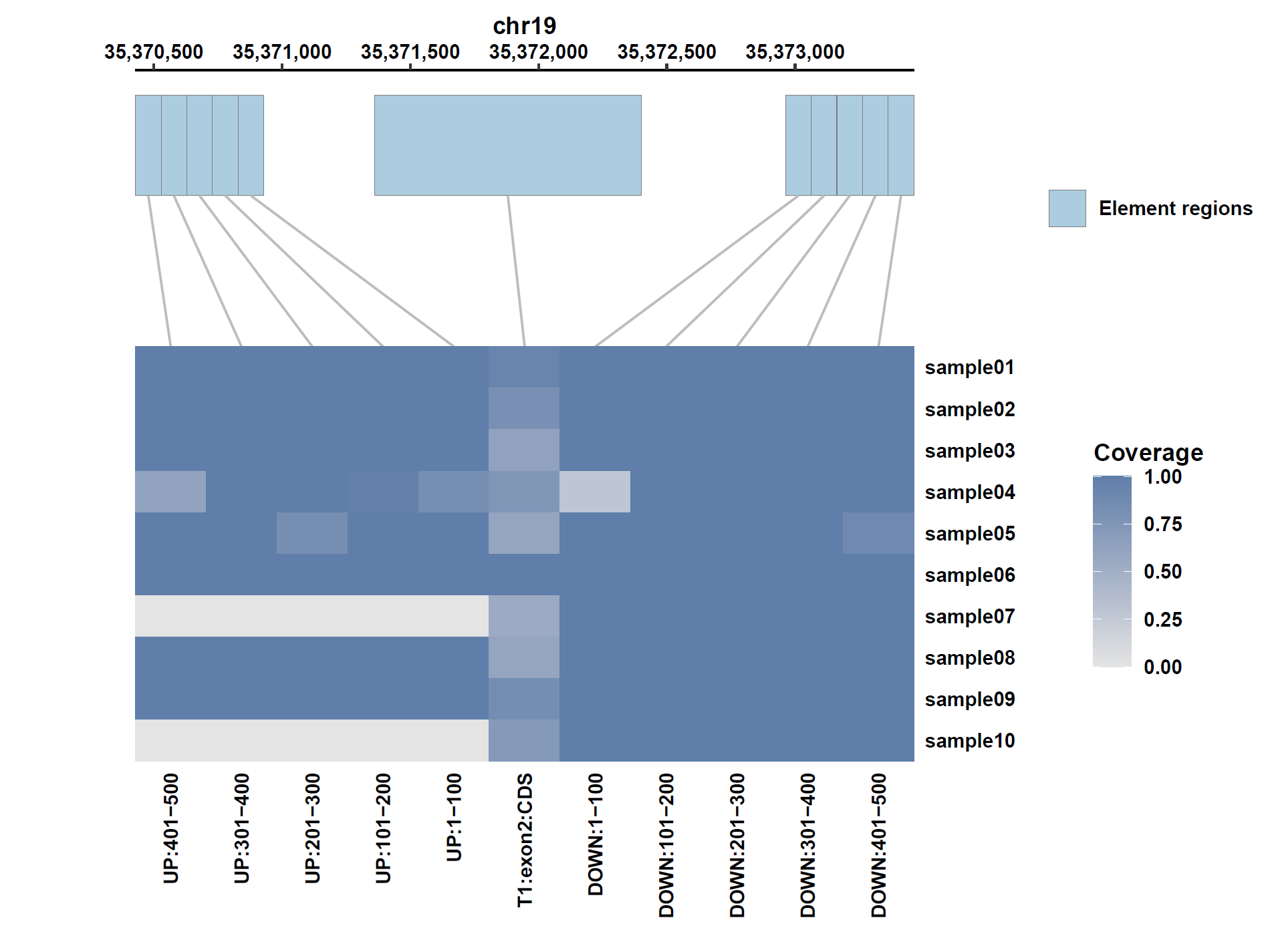


plot_ele_depth(ele_depth, ele_cov, top_anno_height = 0.2)


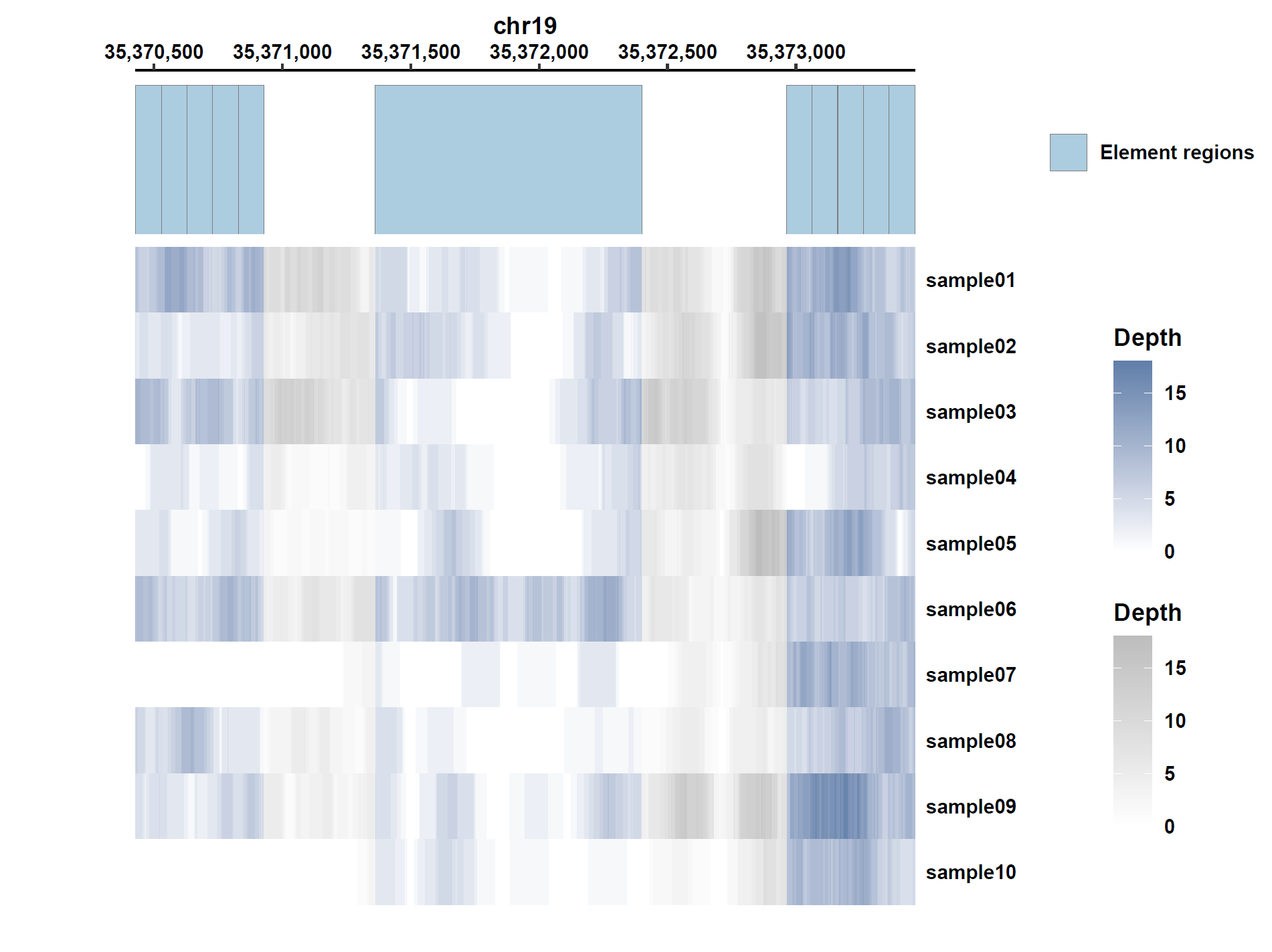


Samples can also be annotated with phenotypic information alongside the heatmap.

plot_ele_pav(ele_pav, pheno_data = ele_pheno, cell_border = “white”, pheno_border = “white”)


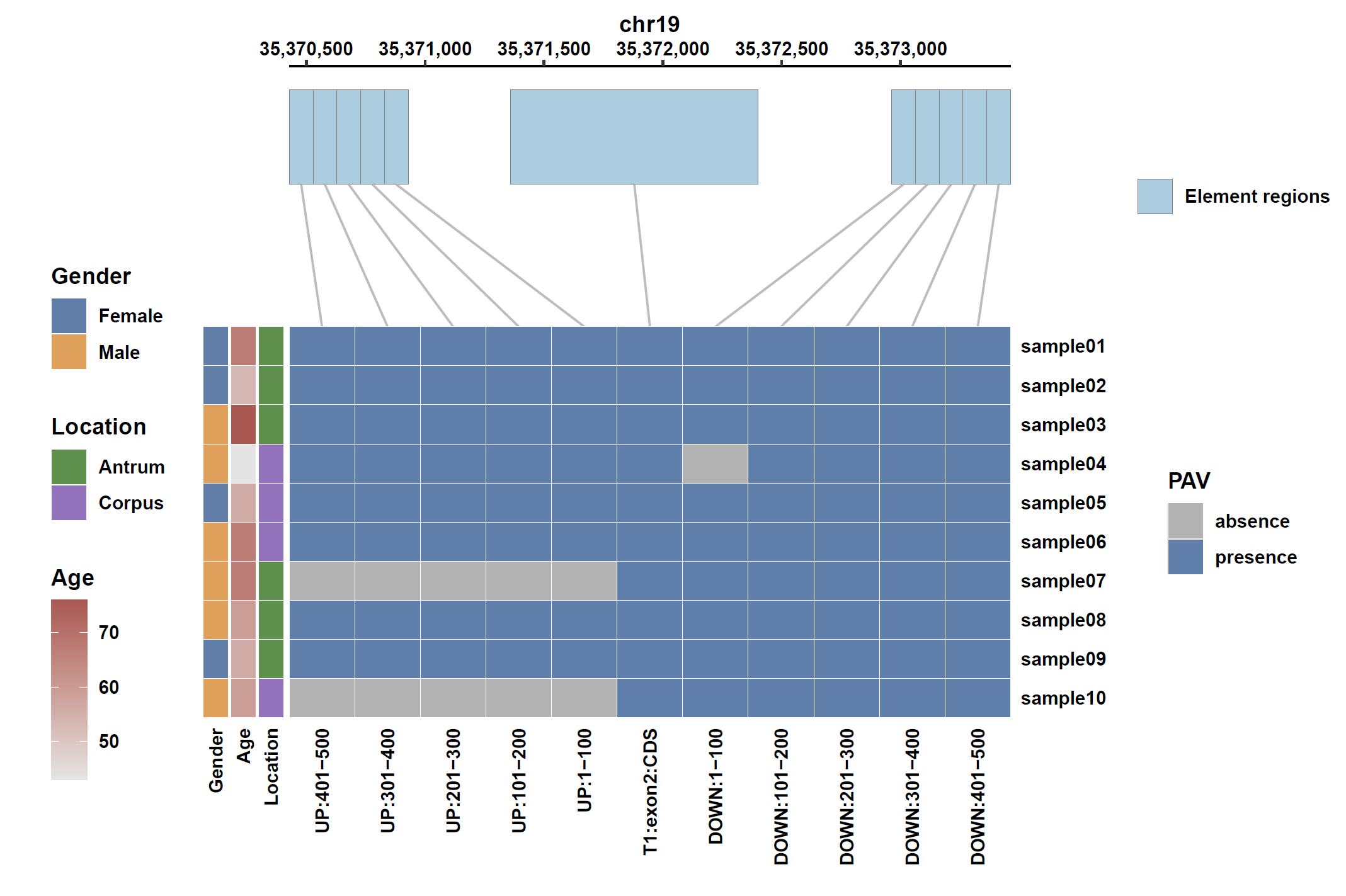


plot_ele_cov(ele_cov, pheno_data = ele_pheno, cell_border = “white”, pheno_border = “white”)


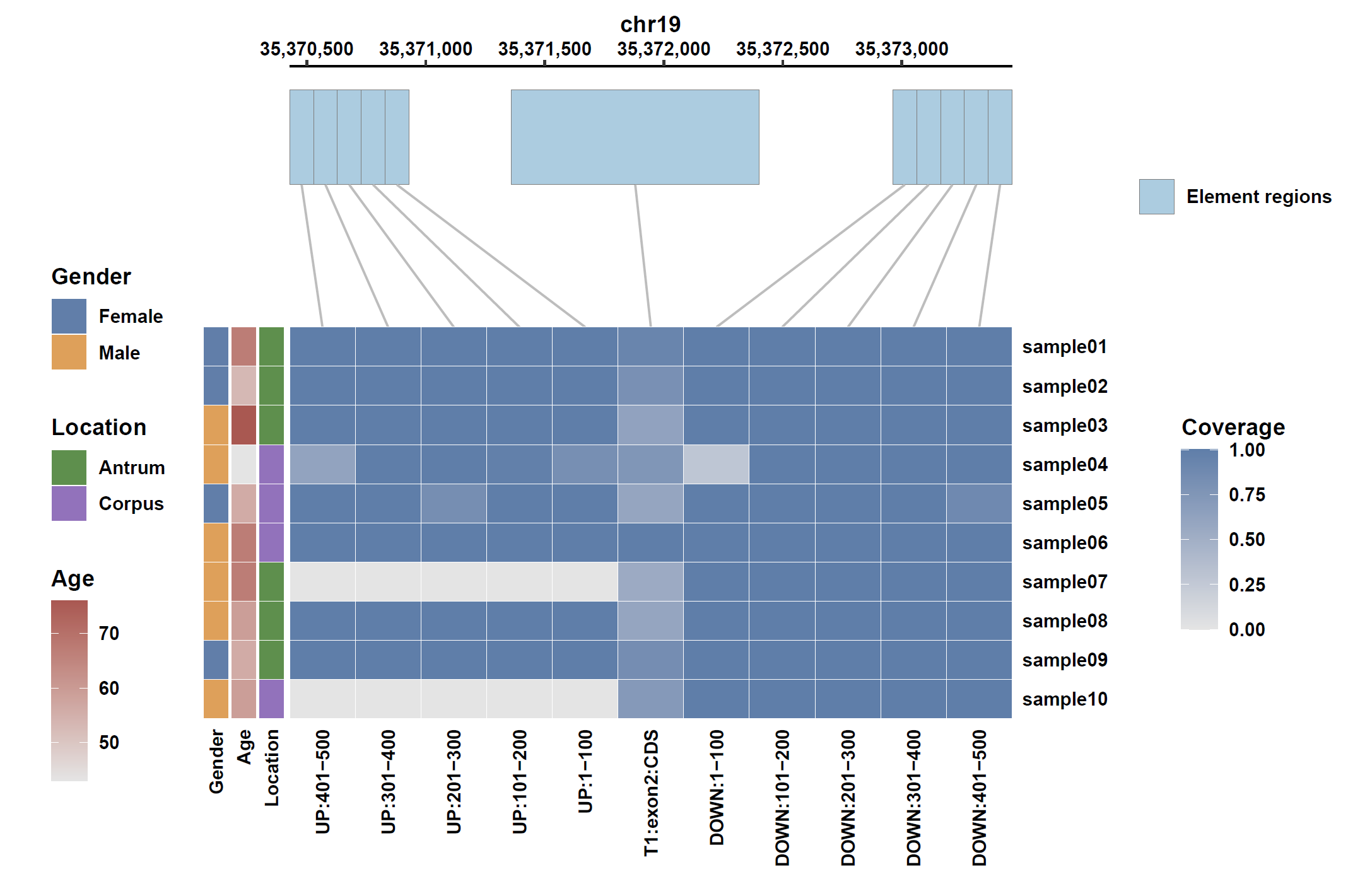


plot_ele_depth(ele_depth, ele_cov, pheno_data = ele_pheno, top_anno_height = 0.2)


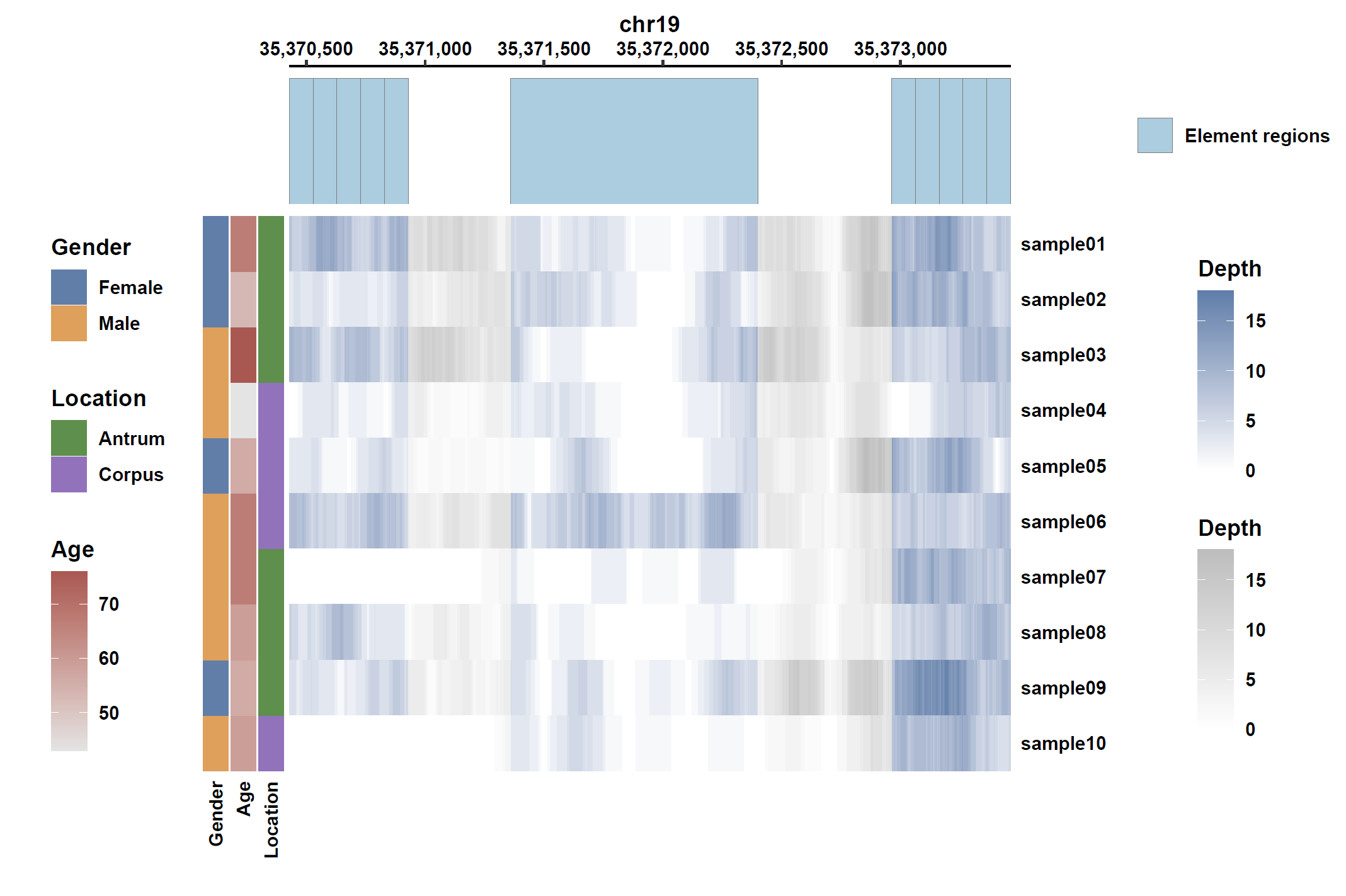


Additionally, if your target region pertains to a gene, you can incorporate gene annotation information to provide further insight.

plot_ele_pav(ele_pav, gff_data = ele_gff, pheno_data = ele_pheno,

cell_border = “white”, pheno_border = “white”)


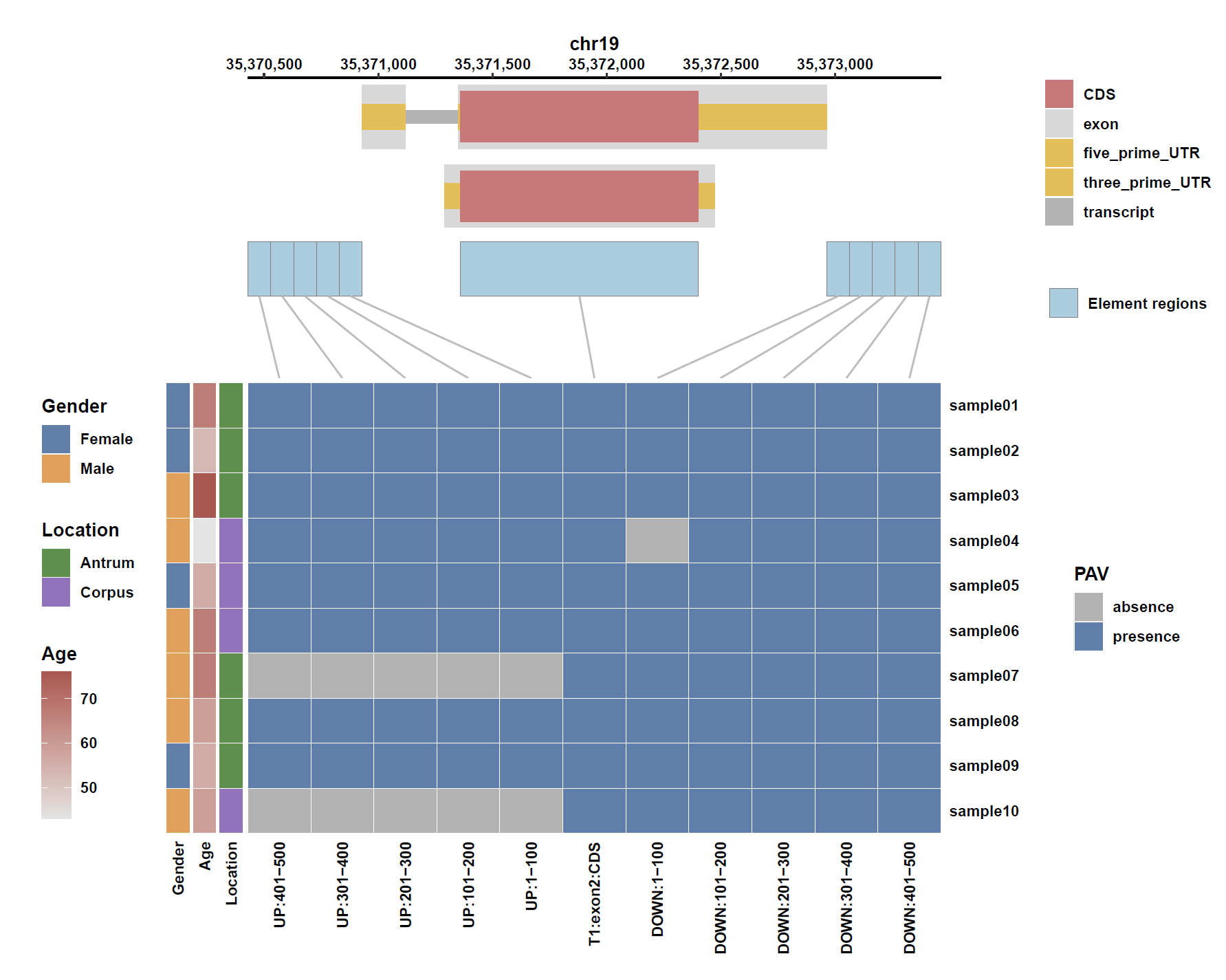


plot_ele_cov(ele_cov, gff_data = ele_gff, pheno_data = ele_pheno,

cell_border = “white”, pheno_border = “white”)


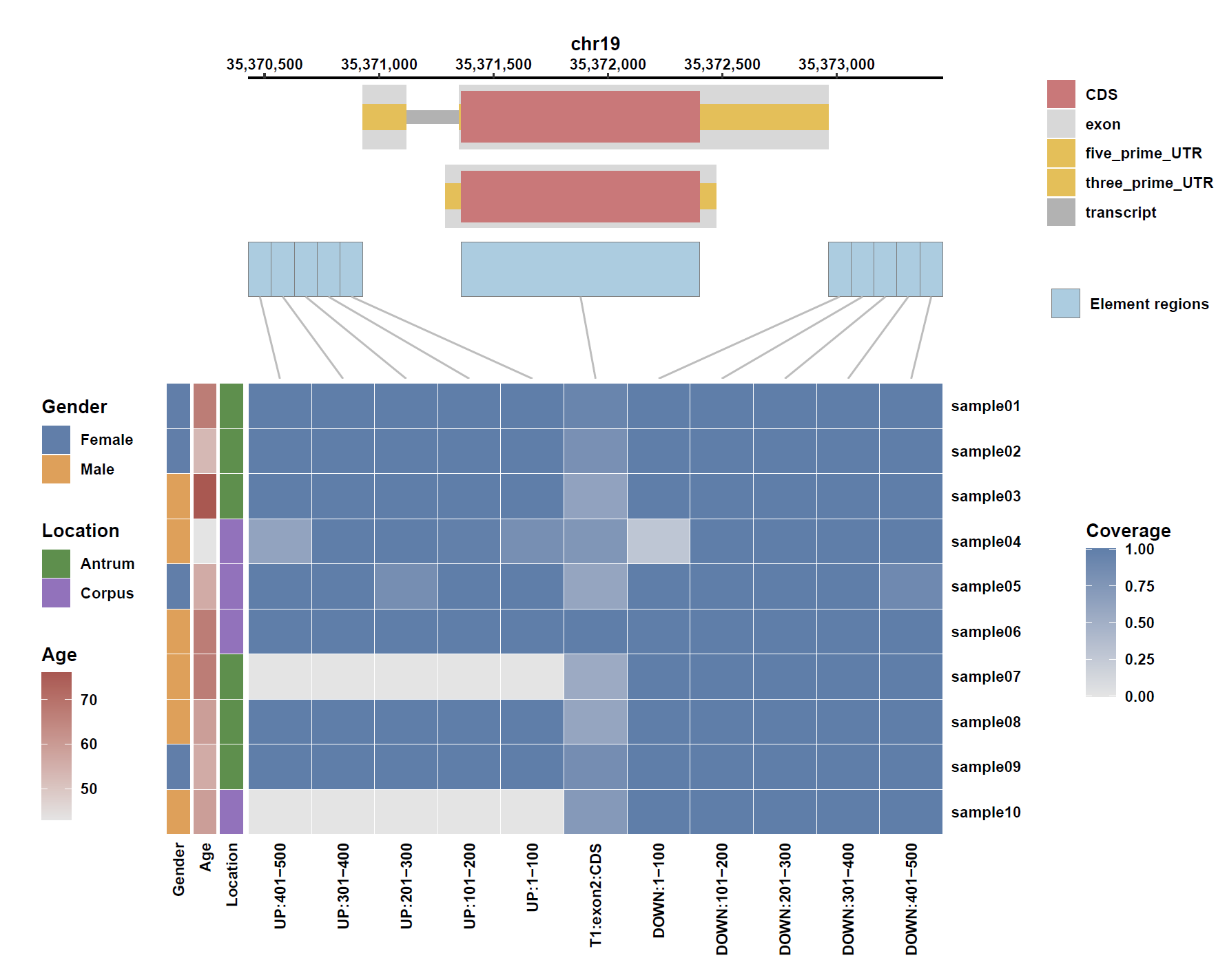


plot_ele_depth(ele_depth, ele_cov, gff_data = ele_gff, pheno_data = ele_pheno)


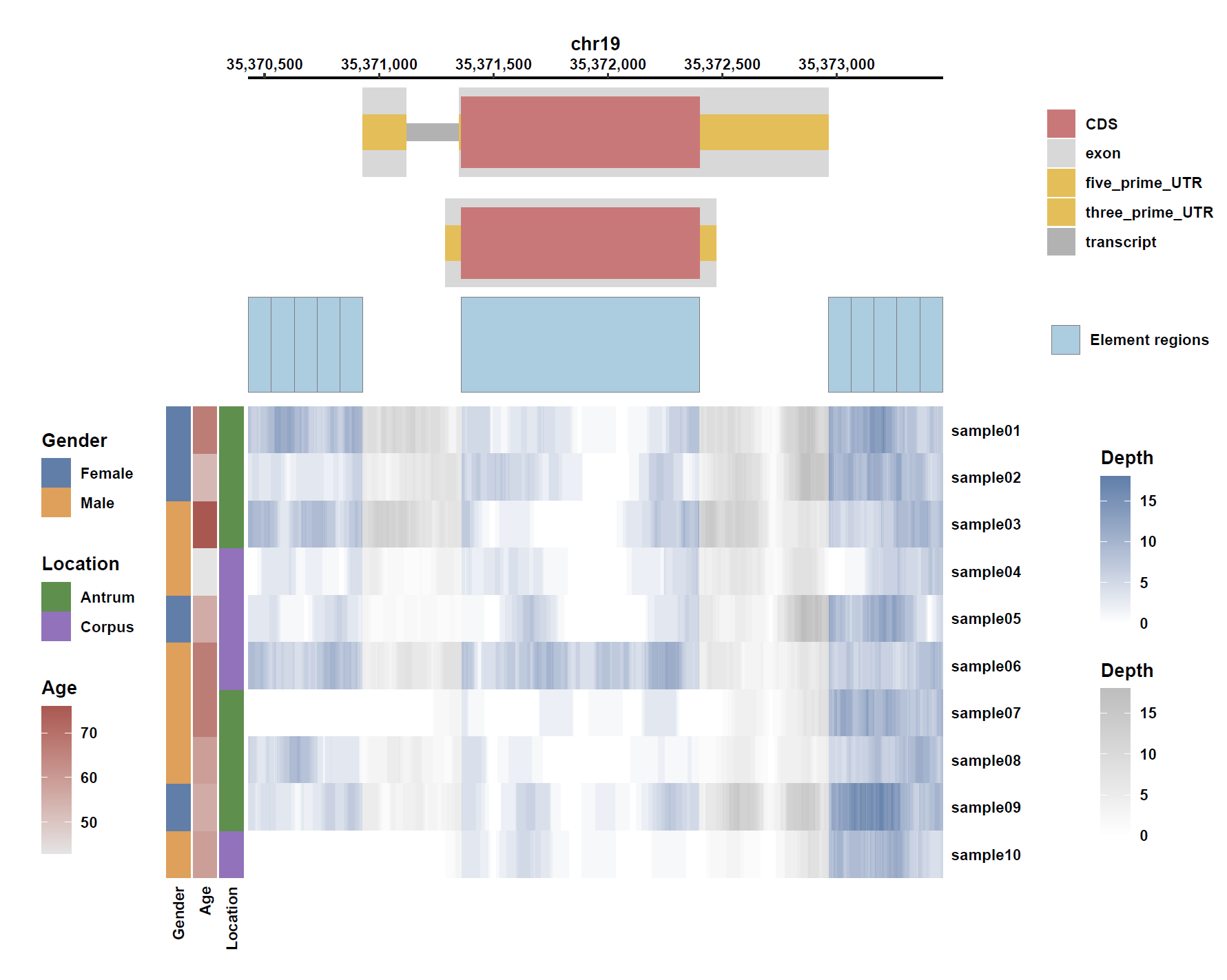

Supplement: S2 Text — (DOCX) [file pcbi.1013288.s002.docx]
