# Supplementary material for: APAV: An advanced pangenome analysis and visualization toolkit
Source: PLoS Comput Biol. 2025 Jul 7;21(7):e1013288. doi: 10.1371/journal.pcbi.1013288 (PMC12251200; doi:10.1371/journal.pcbi.1013288)
Supplement: S3 Text — (DOCX) [file pcbi.1013288.s003.docx]

**S3 Text. Supporting information for “APAV: An advanced pangenome analysis and visualization toolkit”**

**The code for the case study analysis on rice and cancer datasets.**

## **Rice**

**Input data description**

ricepan.gff (35M, 50995 genes): Annotation file for the rice pan-genome.

ricepan.chrl (0.4K): File recording chromosome lengths of the rice pan-genome.

rice.pheno (13K, 453 samples, 3 phenotypes): Phenotype information file for rice samples.

rice_bam/ (5.44TB, 453 samples): Directory storing alignment results.

**Data download**

| $ mkdir data && cd data;  $ wget https://cgm.sjtu.edu.cn/APAV/data/rice/ricepan.gff.gz && gunzip ricepan.gff.gz  $ wget "https://cgm.sjtu.edu.cn/APAV/data/rice/ricepan.chrl"  $ wget "https://cgm.sjtu.edu.cn/APAV/data/rice/rice.pheno"  $ cd ..  *## The BAM files are too large to download. These files are primarily used for calculating coverage in the 1.2 section. You can directly download the coverage calculation results for subsequent analysis.*  $ wget https://cgm.sjtu.edu.cn/APAV/case/rice/ricepan.cov  $ wget https://cgm.sjtu.edu.cn/APAV/case/rice/ricepan_ele.cov |
| --- |

### **1.1 Coordinate Extraction**

Extract genomic coordinates for genes and genetic elements from GFF annotation files:

| $ apav gff2bed --gff data/ricepan.gff --chrl data/ricepan.chrl  *## Output: ricepan.bed* |
| --- |

### **1.2 Coverage Calculation**

Calculate coverage for gene and element regions across all samples based on alignment results, using the transcript with the longest CDS region as the representative transcript for the gene:

| *## This step will consume a lot of time and you can download the results directly:*  *## wget https://cgm.sjtu.edu.cn/APAV/case/rice/ricepan.cov*  *## wget https://cgm.sjtu.edu.cn/APAV/case/rice/ricepan_ele.cov*  $ apav staCov --bed ricepan.bed --bamdir data/rice_bam --asgene --rep cdslen  *## Output: ricepan.cov, ricepan_ele.cov* |
| --- |

### **1.3 PAV Determination**

Determine gene PAV at 50% threshold (genes with coverage ≥50% are classified as "presence", otherwise "absence"):

| $ apav callPAV --cov ricepan.cov --pheno data/rice.pheno --thre 0.5  *## Output: ricepan_all.pav, ricepan_dispensable.pav* |
| --- |

Determine element PAV at 50% threshold:

| $ apav callPAV --cov ricepan_ele.cov --pheno data/rice.pheno --thre 0.5  *## Output: ricepan_ele_all.pav, ricepan_ele_dispensable.pav* |
| --- |

Remove genes absent across all samples:

| $ awk '{if ($1 ~ /^#/) {print; next}; for (i=6; i<=NF; i++) if ($i != 0) {print; next};}' ricepan_all.pav > ricepan.pav  $ awk '{if ($1 ~ /^#/) {print; next}; for (i=6; i<=NF; i++) if ($i != 0) {print; next};}' ricepan_dispensable.pav > ricepan_dis.pav  *## Output: ricepan.pav, ricepan_dis.pav* |
| --- |

### **1.4 Pangenome Size Estimation**

Extract grouping information from the phenotype file:

| $ cat data/rice.pheno \| cut -f 1,2 > rice.group  *## Output: rice.group* |
| --- |

Estimate pan-genome size by group:

| $ apav pavSize --pav ricepan.pav --group rice.group --out rice_grouped.size  *## Output: rice_grouped.size* |
| --- |

Plot the size curve:

| $ apav pavPlotSize --size rice_grouped.size \  --path_color '#9a71c1,#ea9e4a,#4d9242,#5880ae,#b7514d' \  --ribbon_fill '#9a71c1,#ea9e4a,#4d9242,#5880ae,#b7514d' \  --y_title "Gene Number" --fig_height 3 --fig_width 5  *## Output: rice_grouped_size_curve.pdf* |
| --- |


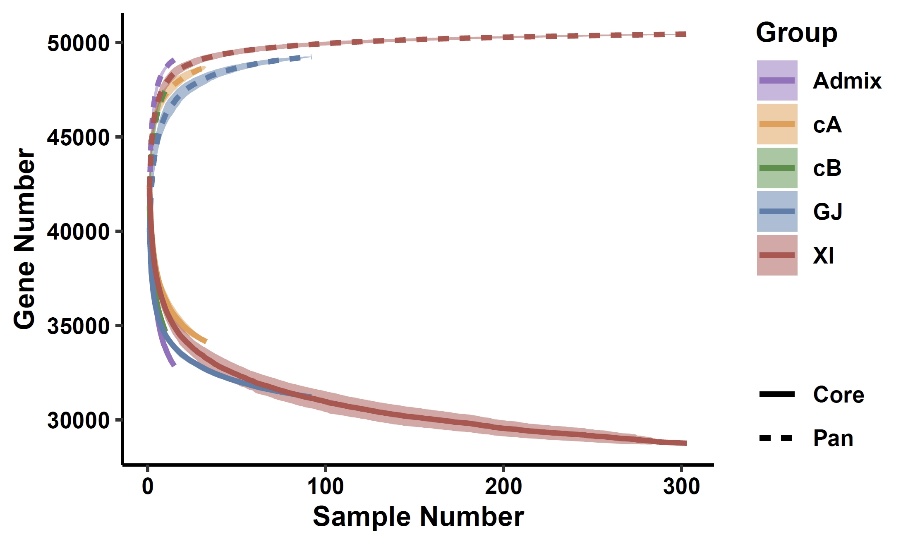


### **1.5 PAV analysis**

Classify genes into core, soft-core, distributed, and private categories:

| $ apav pavPlotHist --pav ricepan.pav --y_title "Gene Number" \  --ring_r 0.45 --ring_label_size 3 --fig_height 2.5 --fig_width 4  *## Output: ricepan_pav_hist.pdf* |
| --- |


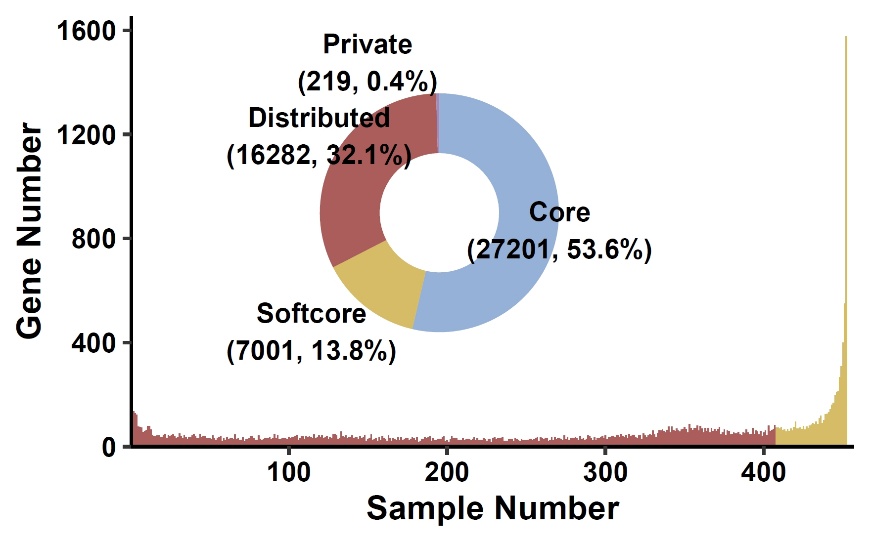


Compare gene counts across phenotype groups:

| $ apav pavPlotStat --pav ricepan.pav --pheno data/rice.pheno \  --add_pheno_info Group --y_title "Gene Number" \  --pheno_info_colors '#aa88cb,#f2b372,#76aa6a,#7e9bc0,#cb7871' \  --fig_width 4 --fig_height 2.5  *## Output: ricepan_pav_sta.pdf* |
| --- |


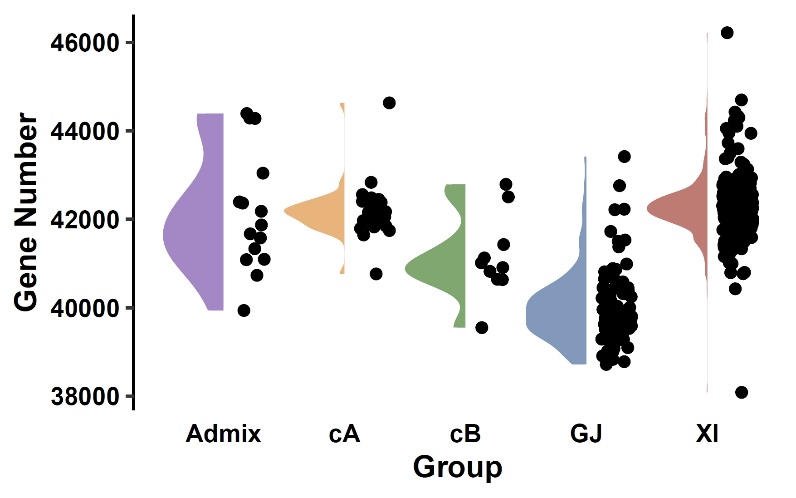


Plot heatmap for non-core genes:

| $ cat data/rice.pheno \| cut -f 1,2,3 > rice.anno  $ apav pavPlotHeat --pav ricepan_dis.pav --pheno rice.anno \  --cluster_rows --row_dend_side right \  --pheno_info_color_list Group=#9F79EE,#FFD700,#7CCD7C,#6CA6CD,#D66B6B \  --pheno_info_color_list Subpopulation=#AD98D6,#F0E68C,#B4EEB4,#36648B,#6CA6CD,#87CEFF,#CAE1FF,#8B2500,#CD3700,#EE5C42,#F08080,#FFAEB9 \  --fig_width 10 --fig_height 6.5  *## Output: ricepan_dis_pav_heatmap.pdf* |
| --- |


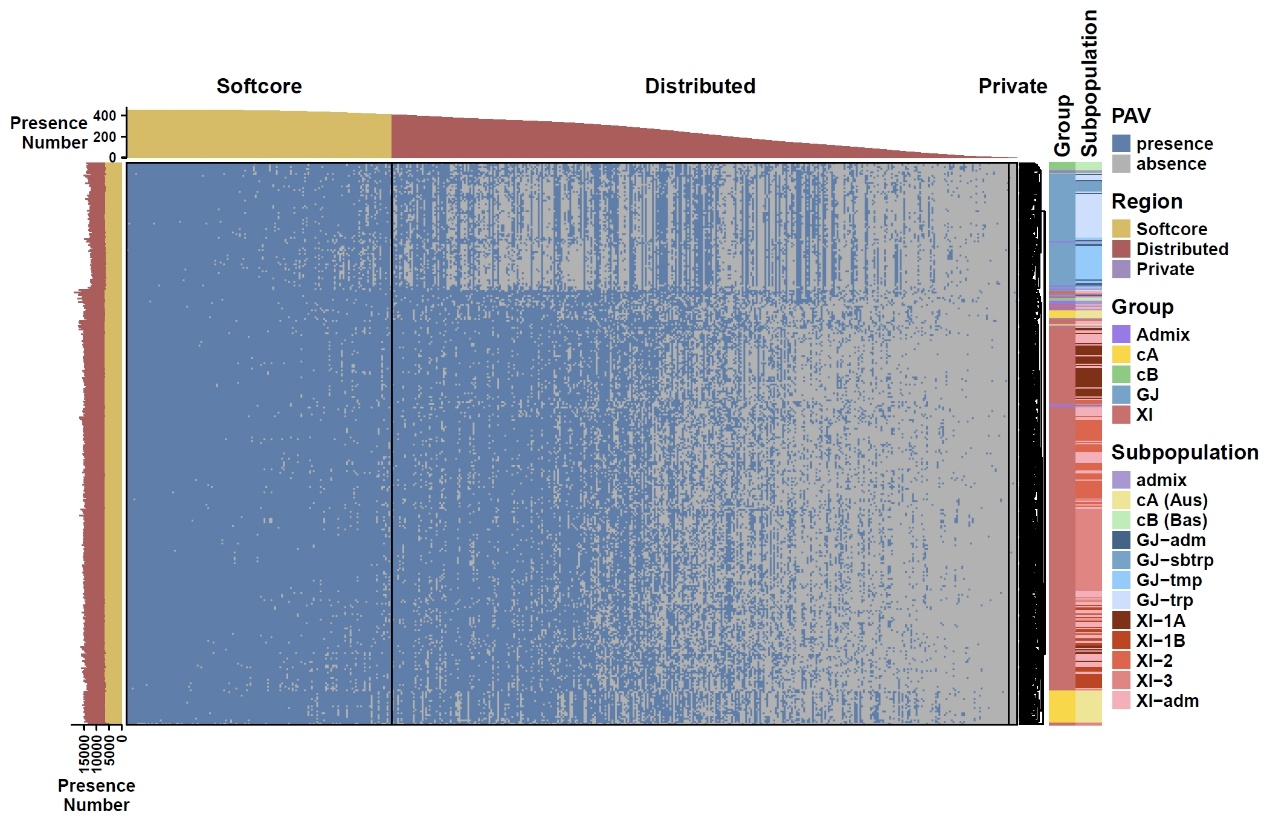


PCA based on non-core gene PAV:

| $ apav pavPCA --pav ricepan_dis.pav --pheno data/rice.pheno --add_pheno_info Group \  --pheno_info_colors '#9a71c1,#ea9e4a,#4d9242,#5880ae,#b7514d' \  --fig_height 3 --fig_width 4  *## Output: ricepan_dis_pav_pca.pdf* |
| --- |


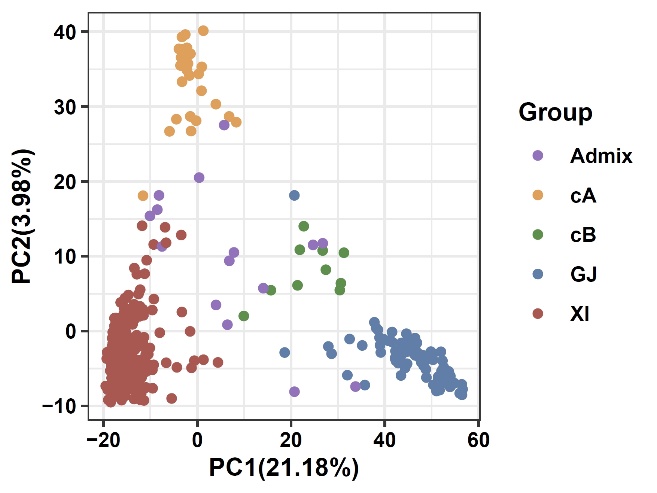


### **1.6 Phenotype Association Analysis**

Calculate associations between non-core genes and phenotypes:

| $ apav pavStaPheno --pav ricepan_dis.pav --pheno data/rice.pheno  *## Output: ricepan_dis.phenores* |
| --- |

Manhattan plot for continuous phenotypes (e.g., plant height):

| $ apav pavPlotPhenoMan --pav ricepan_dis.pav --pheno data/rice.pheno \  --pheno_res ricepan_dis.phenores --pheno_name Height \  --x_text_angle 90 --fig_height 4 --fig_width 7  *## Output: ricepan_dis_pheno_Height_manhattan.pdf* |
| --- |


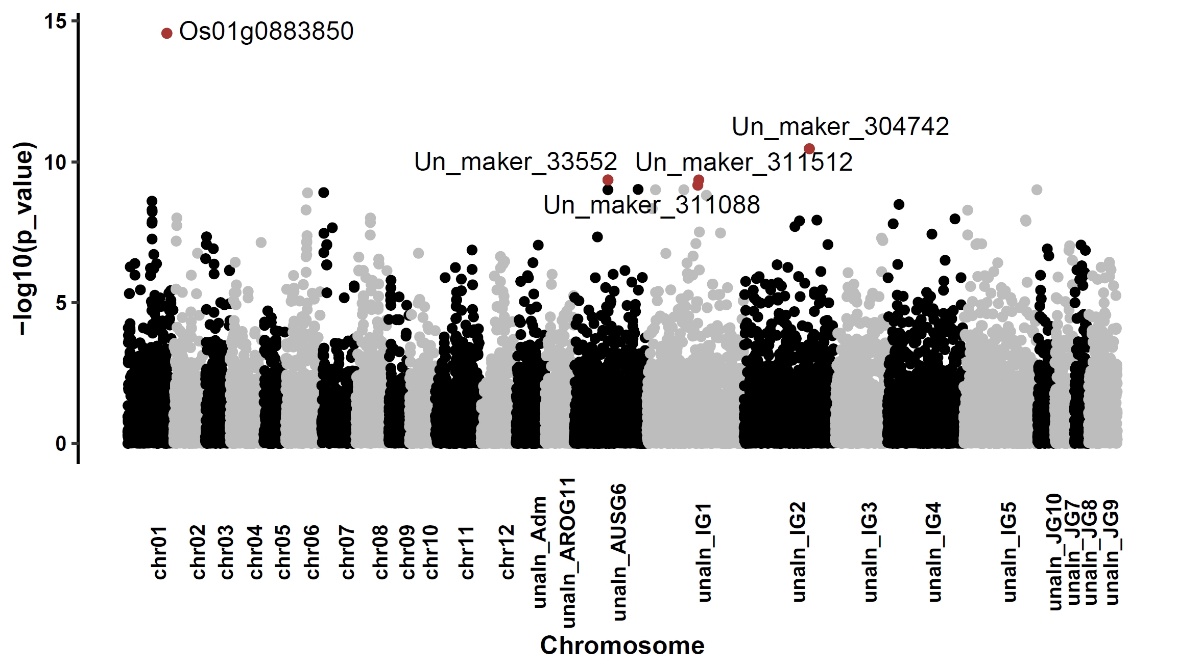


Visualize the association between gene PAV and a continuous phenotype (e.g., the *Os01g0883850* gene and plant height) in a violin plot:

| $ apav pavPlotPhenoVio --pav ricepan.pav --pheno data/rice.pheno \  --pheno_name Height --region_name Os01g0883850 --fig_height 4 --fig_width 3  *## Output: ricepan_pheno_Height_Os01g0883850_violin.pdf* |
| --- |


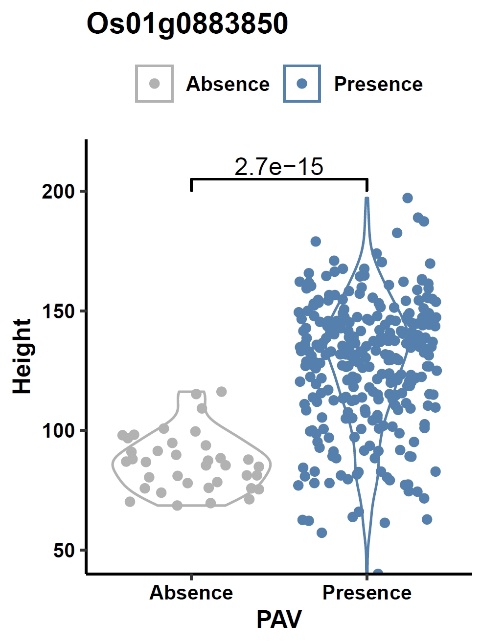


Visualize the association between a gene and a discrete phenotype (e.g., the *Un_maker_208231* gene and accession group) in a bar plot:

| $ apav pavPlotPhenoBar --pav ricepan.pav --pheno data/rice.pheno \  --pheno_name Group --region_name Un_maker_208231 --fig_height 4 --fig_width 3  *## Output: ricepan_pheno_Group_Un_maker_208231_bar.pdf* |
| --- |


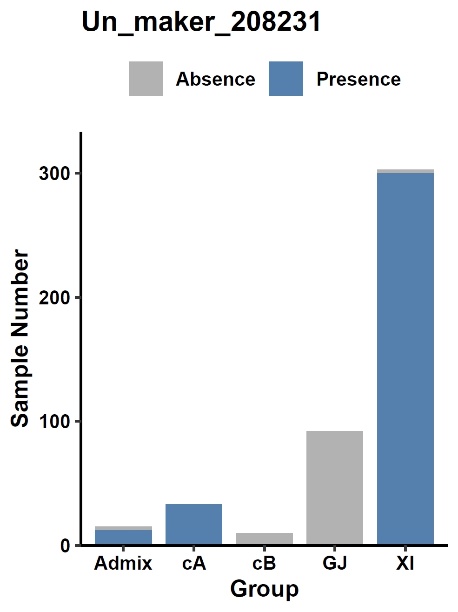


### **1.7 Element Visualization (*Os04g0373400*)**

Prepare annotation and data files:

| $ mkdir ele_example_mate && cd ele_example_mate  $ grep 'Os04g0373400' ../data/ricepan.gff > Os04g0373400.gff  $ grep -E 'Annotation\|Os04g0373400' ../ricepan_ele.cov > Os04g0373400.elecov  $ grep -E 'Annotation\|Os04g0373400' ../ricepan_ele_all.pav > Os04g0373400.elepav  *## Output: Os04g0373400.gff, Os04g0373400.elecov, Os04g0373400.elecov* |
| --- |

Plot coverage, PAV and depth:

| $ apav elePlotCov --elecov Os04g0373400.elecov --gff Os04g0373400.gff \  --cluster_samples --hide_sample_name --top_anno_height 0.3 --fig_width 7.5 --fig_height 7.5  $ apav elePlotPAV --elepav Os04g0373400.elepav --gff Os04g0373400.gff \  --cluster_samples --hide_sample_name --top_anno_height 0.3 --fig_width 7.5 --fig_height 7.5  $ apav elePlotDepth --ele Os04g0373400.elecov \  --bamdir ../data/rice_bam/ --gff Os04g0373400.gff \  --cluster_samples --hide_sample_name --log10 --top_anno_height 0.3 \  --fig_width 7.5 --fig_height 7.5  *## Output: Os04g0373400.elecov.pdf, Os04g0373400.elepav.pdf, Os04g0373400.elecov.depth.pdf* |
| --- |


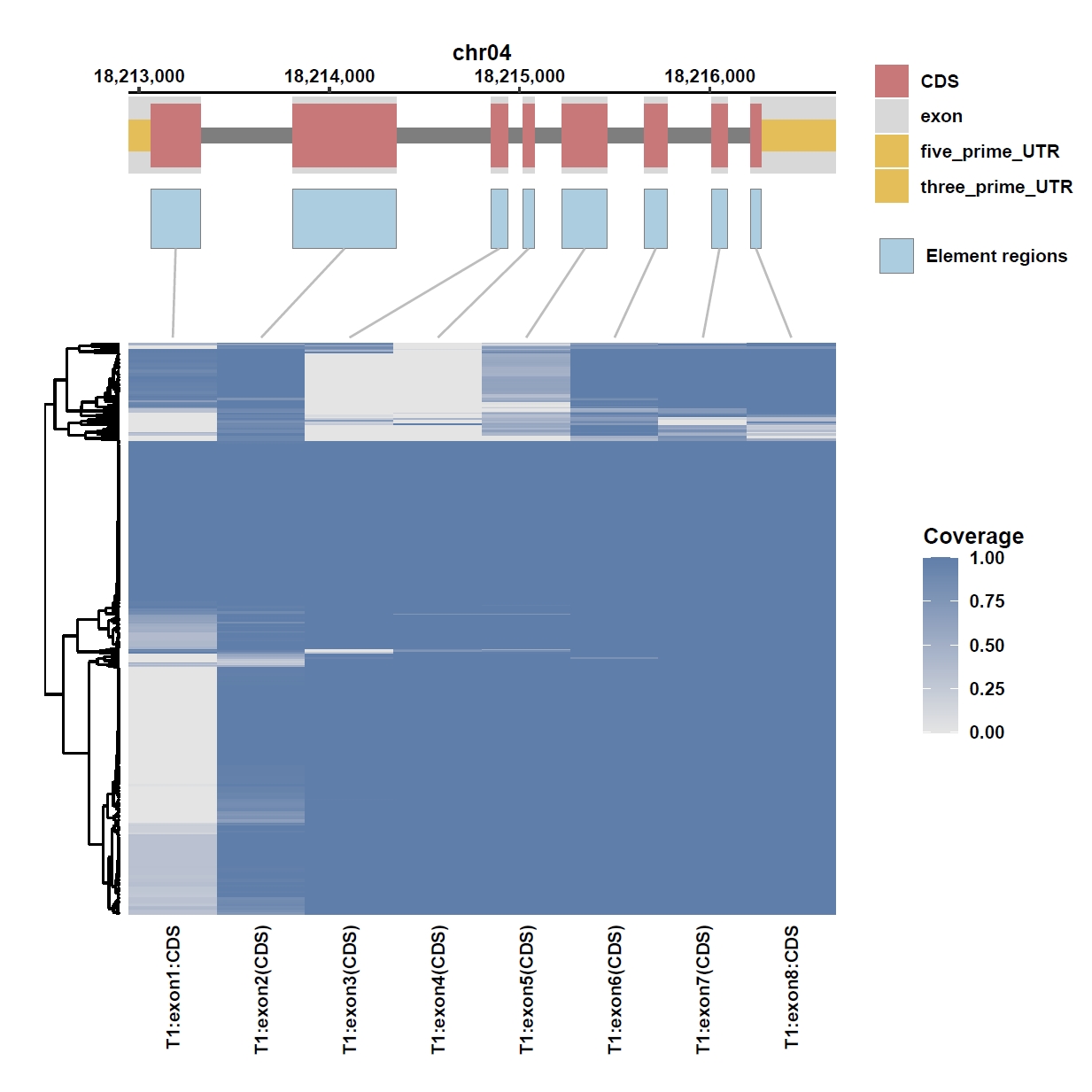

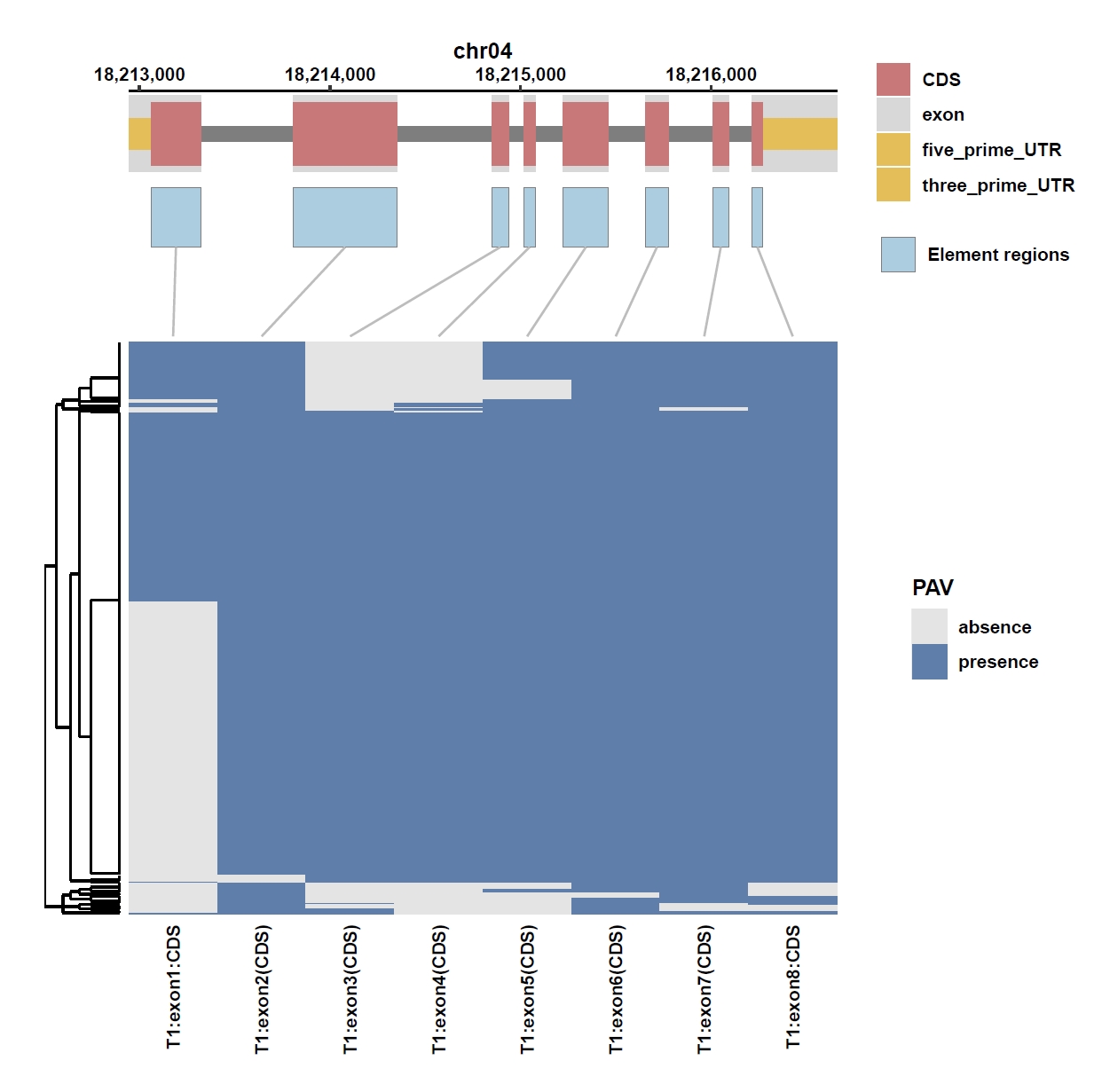

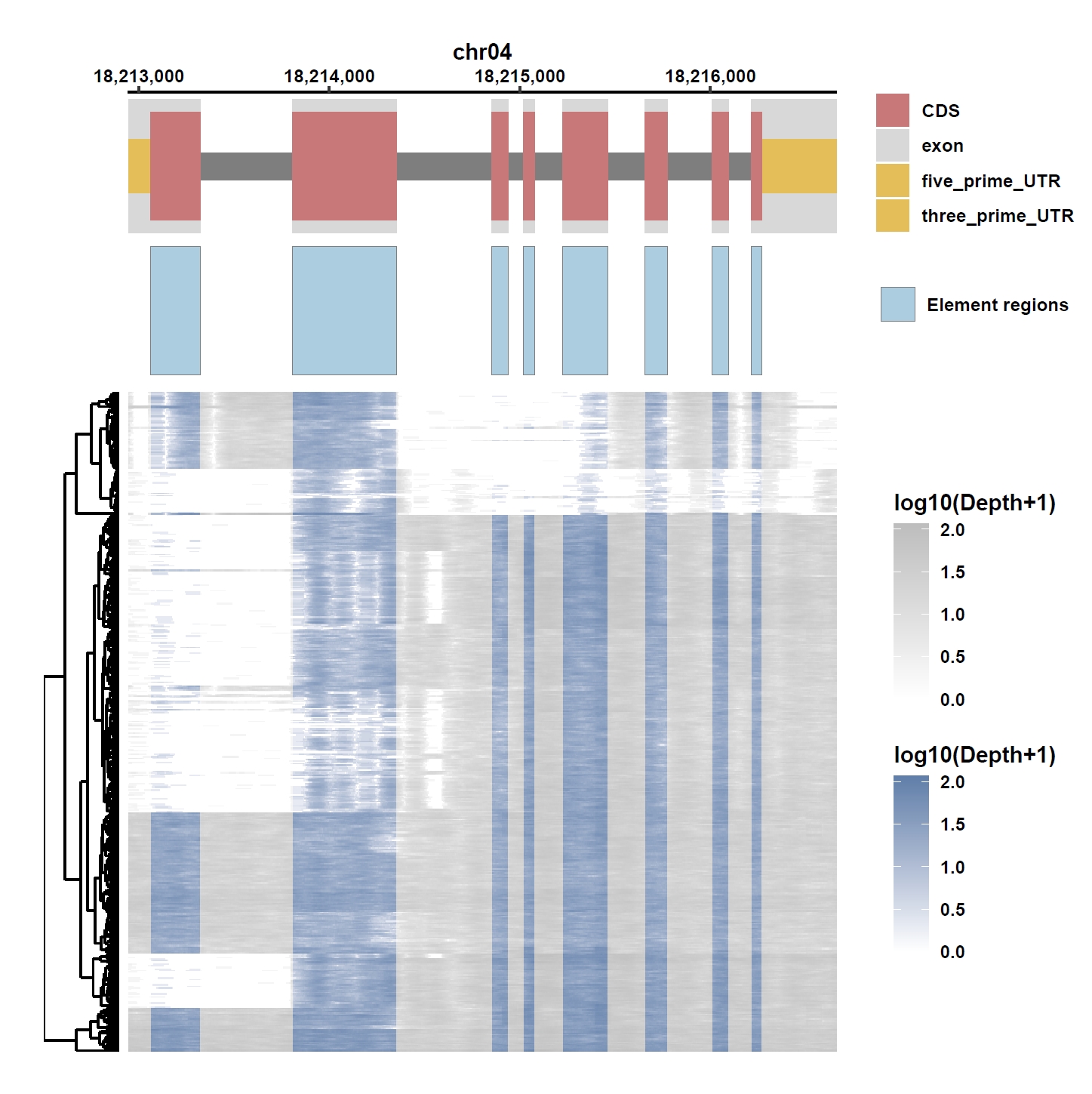


View phenotype association with group classification for gene and specific exons:

| $ apav pavPlotPhenoBar --pav ../ricepan.pav --pheno ../data/rice.pheno \  --pheno_name Group --region_name Os04g0373400 --fig_width 4 \  --fig_height 4 --out Os04g0373400_group  $ apav pavPlotPhenoBar --pav Os04g0373400.elepav --pheno ../data/rice.pheno \  --pheno_name Group --region_name 'Os04g0373400:[T1:exon1:CDS]' \  --fig_width 4 --fig_height 4 --out Os04g0373400_group_exon1  $ apav pavPlotPhenoBar --pav Os04g0373400.elepav --pheno ../data/rice.pheno \  --pheno_name Group --region_name 'Os04g0373400:[T1:exon3(CDS)]' \  --fig_width 4 --fig_height 4 --out Os04g0373400_group_exon3  $ apav pavPlotPhenoBar --pav Os04g0373400.elepav --pheno ../data/rice.pheno \  --pheno_name Group --region_name 'Os04g0373400:[T1:exon4(CDS)]' \  --fig_width 4 --fig_height 4 --out Os04g0373400_group_exon4  $ cd ..  *## Output: Os04g0373400_group.pdf, Os04g0373400_group_exon1.pdf, Os04g0373400_group_exon3.pdf, Os04g0373400_group_exon4.pdf* |
| --- |


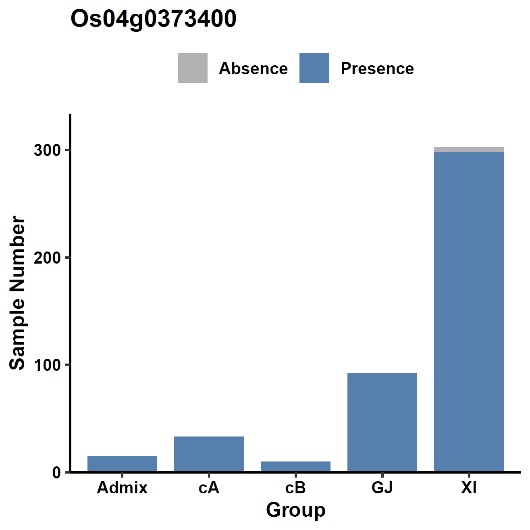

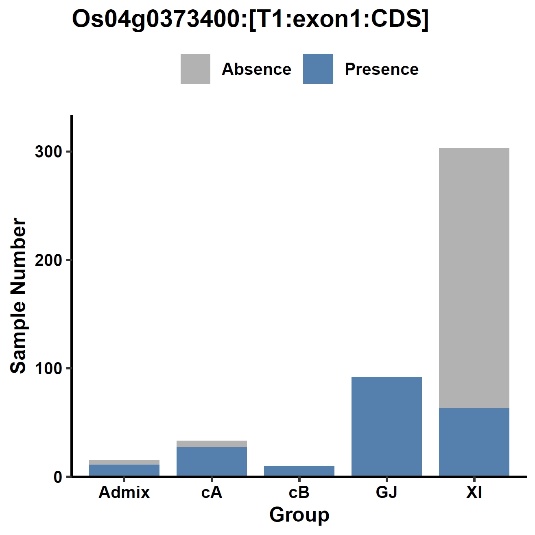

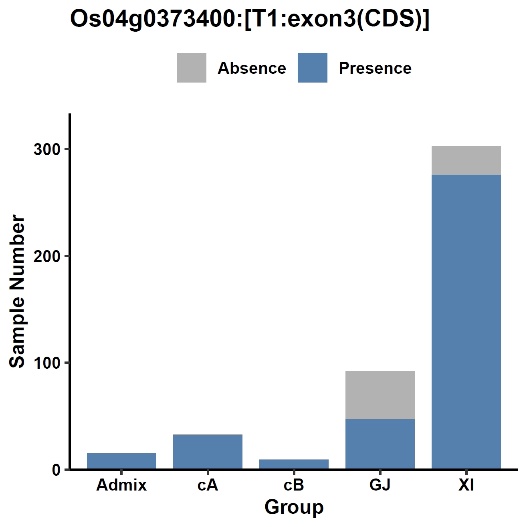

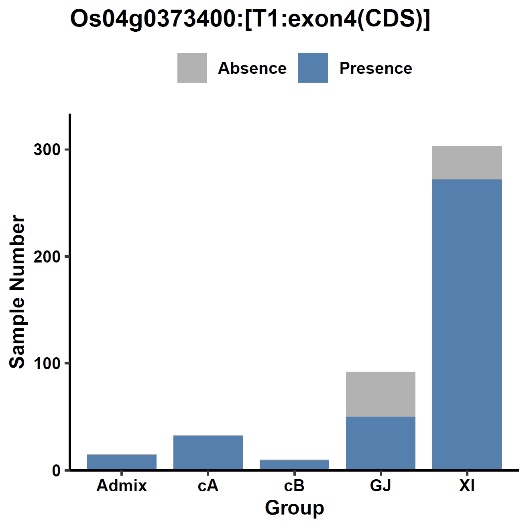


### **1.8 Element Visualization (*sd1*)**

Prepare annotation and data files:

| $ mkdir ele_example_sd1 && cd ele_example_sd1  $ grep 'Os01g0883800' ../data/ricepan.gff > Os01g0883800.gff  $ grep -E 'Annotation\|Os01g0883800' ../ricepan_ele.cov > Os01g0883800.elecov  $ grep -E 'Annotation\|Os01g0883800' ../ricepan_ele_all.pav > Os01g0883800.elepav  *## Output: Os01g0883800.gff, Os01g0883800.elecov, Os01g0883800.elecov* |
| --- |

Plot coverage, PAV and depth:

| $ apav elePlotCov --elecov Os01g0883800.elecov --gff Os01g0883800.gff \  --hide_sample_name --top_anno_height 0.25 --fig_width 7.5 --fig_height 6  $ apav elePlotPAV --elepav Os01g0883800.elepav --gff Os01g0883800.gff \  --hide_sample_name --top_anno_height 0.25 --fig_width 7.5 --fig_height 6  $ apav elePlotDepth --ele Os01g0883800.elecov \  --bamdir ../data/rice_bam/ --gff Os01g0883800.gff --hide_sample_name \  --log10 --top_anno_height 0.25 --fig_width 7.5 --fig_height 6  *## Output: Os01g0883800.elecov.pdf, Os01g0883800.elepav.pdf, Os01g0883800.elecov.depth.pdf* |
| --- |


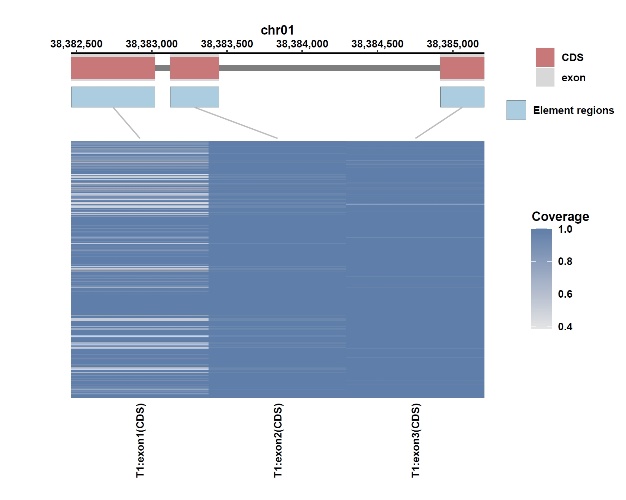

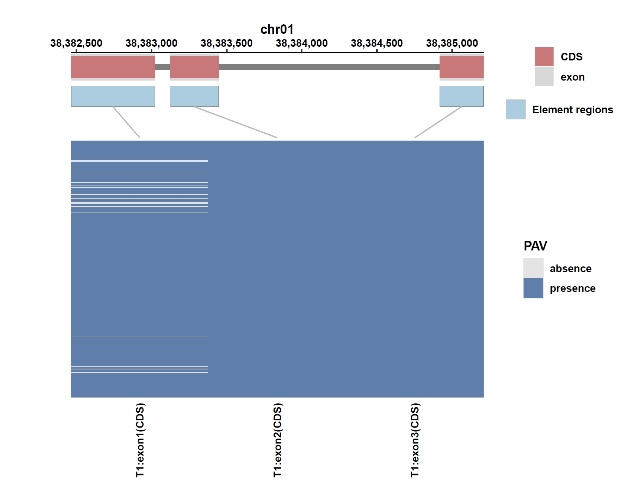

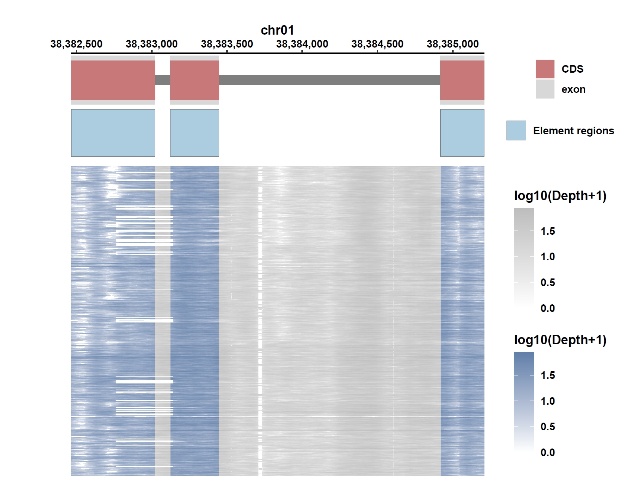


View phenotype association with plant height for gene and exons:

| $ apav pavPlotPhenoVio --pav ../ricepan.pav --pheno ../data/rice.pheno \  --pheno_name Height --region_name Os01g0883800 \  --fig_width 4 --fig_height 4 --out Os01g0883800_height  $ apav pavPlotPhenoVio --pav Os01g0883800.elepav --pheno ../data/rice.pheno \  --pheno_name Height --region_name 'Os01g0883800:[T1:exon1(CDS)]' \  --fig_width 4 --fig_height 4 --out Os01g0883800_height_exon1  $ apav pavPlotPhenoVio --pav Os01g0883800.elepav --pheno ../data/rice.pheno \  --pheno_name Height --region_name 'Os01g0883800:[T1:exon2(CDS)]' \  --fig_width 4 --fig_height 4 --out Os01g0883800_height_exon2  $ apav pavPlotPhenoVio --pav Os01g0883800.elepav --pheno ../data/rice.pheno \  --pheno_name Height --region_name 'Os01g0883800:[T1:exon3(CDS)]' \  --fig_width 4 --fig_height 4 --out Os01g0883800_height_exon3  *## Output: Os01g0883800_height.pdf, Os01g0883800_height_exon1.pdf, Os01g0883800_height_exon2.pdf, Os01g0883800_height_exon3.pdf* |
| --- |


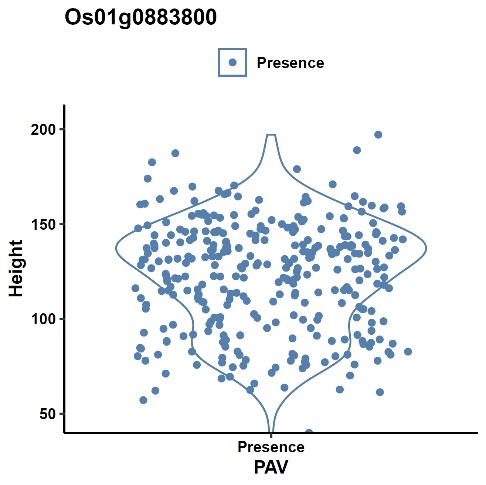

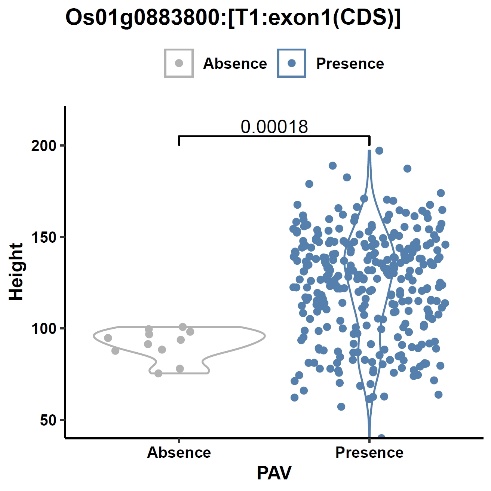

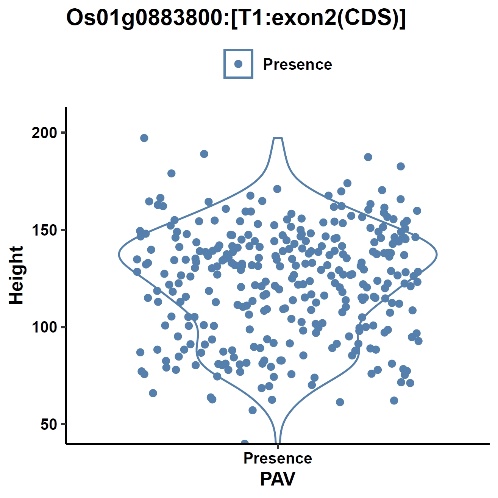

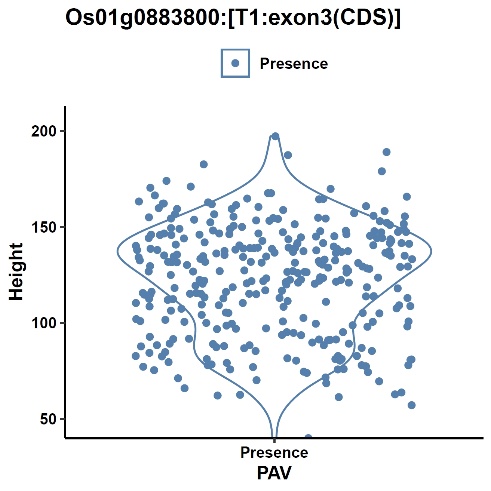


### **Run time**

The computational experiments were performed on an x86-64 server equipped with dual Intel Xeon Gold 6248 processors.

- 1 CPU core, 4GB RAM
- Samtools version: 1.16.1
- R version: 4.3.2

| **Step** | **Input data** | **Run time** |
| --- | --- | --- |
| gff2bed | ricepan.gff(35M) | 5.0s |
| staCov | 453 BAM files and index files (5.44TB), ricepan.bed(15M) | 73h 5min (~9min 40s for one sample) |
| callPAV | ricepan.cov(75M) | 57.4s |
| callPAV | ricepan_ele.cov(180M) | 1min 29.2s |
| pavSize | ricepan.pav(48M) | 2min 1.5s |
| pavPlotSize | rice_grouped.size(1.1M) | 20.9s |
| pavPlotHist | ricepan.pav(48M) | 11.9s |
| pavPlotStat | ricepan.pav(48M) | 11.6s |
| pavPlotHeat | ricepan_dis.pav(22M) | 67.1s |
| pavPCA | ricepan_dis.pav(22M) | 10.8s |
| pavStaPheno | ricepan_dis.pav(22M), rice.pheno(13K) | 2min 33.3s |
| pavPlotPhenoMan | ricepan_dis.phenores(3.6M) | 9.4s |
| pavPlotPhenoVio | ricepan.pav(48M), rice.pheno(13K) | 11.6s |
| pavPlotPhenoBar | ricepan.pav(48M), rice.pheno(13K) | 11.5s |
| elePlotCov, elePlotPAV, elePlotDepth | Os04g0373400.elecov(16K), Os04g0373400.elepav(14K) | 5.6s, 5.6s, 1min 22.5s |
| pavPlotPhenoBar | Os04g0373400.elepav(14K), rice.pheno(13K) | 11.5s, 4.9s, 4.9s, 4.9s |
| elePlotCov, elePlotPAV, elePlotDepth | Os01g0883800.elecov(9.9K), Os01g0883800.elepav(8.4K) | 5.3s, 5.3s, 42.7s |
| pavPlotPhenoVio | Os01g0883800.elepav(8.4K), rice.pheno(13K) | 12.0s, 5.0s, 5.0s, 5.0s |

## **Gastric tumor**

**Input data description**

gcpan.gff3 (1.2G, 58839 genes): Annotation file for the gastric cancer pangenome.

gcpan.chrl (638K): File recording chromosome lengths of the gastric cancer pangenome.

gc.pheno (5.5K, 127samples, 7phenotypes): Phenotype information file for gastric cancer samples.

gc_bam/ (11.94TB, 127 samples): Directory storing alignment results.

**Data download**

| $ mkdir data && cd data;  $ wget https://cgm.sjtu.edu.cn/APAV/data/gc/gcpan.gff3.gz && gunzip gcpan.gff3.gz  $ wget "https://cgm.sjtu.edu.cn/APAV/data/gc/gcpan.chrl"  $ wget "https://cgm.sjtu.edu.cn/APAV/data/gc/gc.pheno"  $ cd ..  *## The BAM files are too large to download. These files are primarily used for calculating coverage in the 2.2 section. You can directly download the coverage calculation results for subsequent analysis.*  $ wget https://cgm.sjtu.edu.cn/APAV/case/gc/gcpan.cov  $ wget https://cgm.sjtu.edu.cn/APAV/case/gc/gcpan_ele.cov |
| --- |

### **2.1 Coordinate Extraction**

Extract genomic coordinates for genes, genetic elements, and flanking regions (1,000 bp upstream/downstream partitioned into 10 × 100 bp intervals) from GFF3 annotation files:

| $ apav gff2bed --gff data/gcpan.gff3 --chrl data/gcpan.chrl \  --up_n 10 --up_bin 100 --down_n 10 --down_bin 100  *#Output: gcpan.bed* |
| --- |

### **2.2 Coverage Calculation**

Calculate coverage for gene and element regions across all samples based on alignment results, using the transcript with the longest CDS region as the representative transcript for the gene:

| *## This step will consume a lot of time and you can download the results directly:*  *## wget https://cgm.sjtu.edu.cn/APAV/case/gc/gcpan.cov*  *## wget https://cgm.sjtu.edu.cn/APAV/case/gc/gcpan_ele.cov*  $ apav staCov --bed gcpan.bed --bamdir data/gc_bam --asgene --rep cdslen  *#Output: gcpan.cov, gcpan_ele.cov* |
| --- |

### **2.3 PAV Determination**

Determine gene PAV at 80% threshold (genes with coverage ≥80% are classified as "presence", otherwise "absence"):

| $ apav callPAV --cov gcpan.cov --pheno data/gc.pheno --thre 0.8  *#Output: gcpan_all.pav, gcpan_dispensable.pav* |
| --- |

Determine element PAV at 80% threshold:

| $ apav callPAV --cov gcpan_ele.cov --pheno data/gc.pheno --thre 0.8  *#Output: gcpan_ele_all.pav, gcpan_ele_dispensable.pav* |
| --- |

### **2.4 Pangenome Size Estimation**

Extract grouping information from the phenotype file:

| $ cat data/gc.pheno \| cut -f 1,3 > gc.group  $ apav pavSize --pav gcpan_all.pav --group gc.group --out gcpan_grouped.size  *#Output: gcpan_grouped.size* |
| --- |

| $ apav pavPlotSize --size gcpan_grouped.size --fig_height 3 --fig_width 5  *#Output: gcpan_grouped_size_curve.pdf* |
| --- |


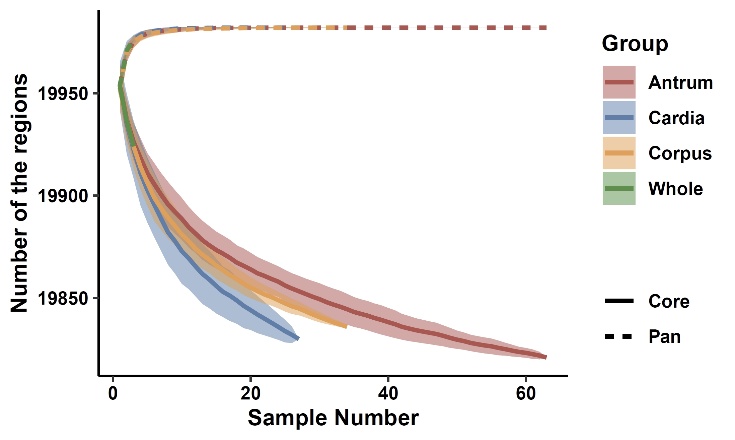


### **2.5 PAV analysis**

Classify genes into core, soft-core, distributed, and private categories:

| $ apav pavPlotHist --pav gcpan_all.pav --ring_r 0.45 --ring_label_size 2.5 \  --fig_height 3 --fig_width 5  *#Output: gcpan_all_pav_hist.pdf* |
| --- |


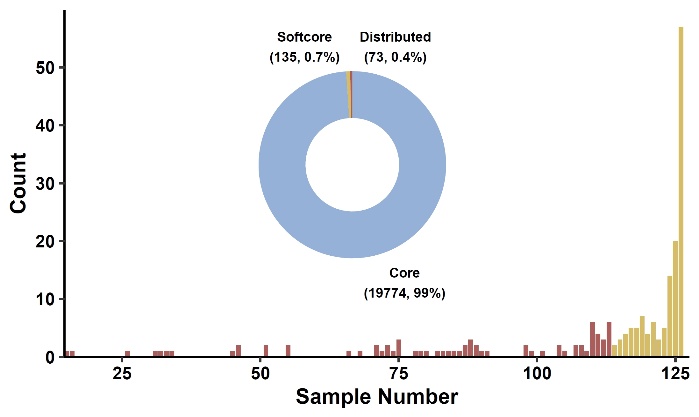


Compare gene counts across phenotype groups:

| $ apav pavPlotStat --pav gcpan_all.pav --pheno data/gc.pheno --add_pheno_info Location \  --fig_width 5 --fig_height 3  *#Output: gcpan_all_pav_sta.pdf* |
| --- |


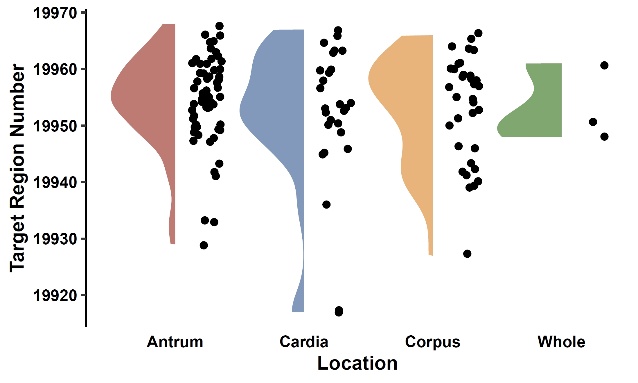


Plot heatmap for non-core genes:

| $ cat data/gc.pheno \| cut -f 1,2,3 > gc.anno  $ apav pavPlotHeat --pav gcpan_dispensable.pav --pheno gc.anno \  --anno_param_row_pheno name_side=bottom --fig_width 10 --fig_height 6  *#Output: gcpan_dispensable_pav_heatmap.pdf* |
| --- |

*
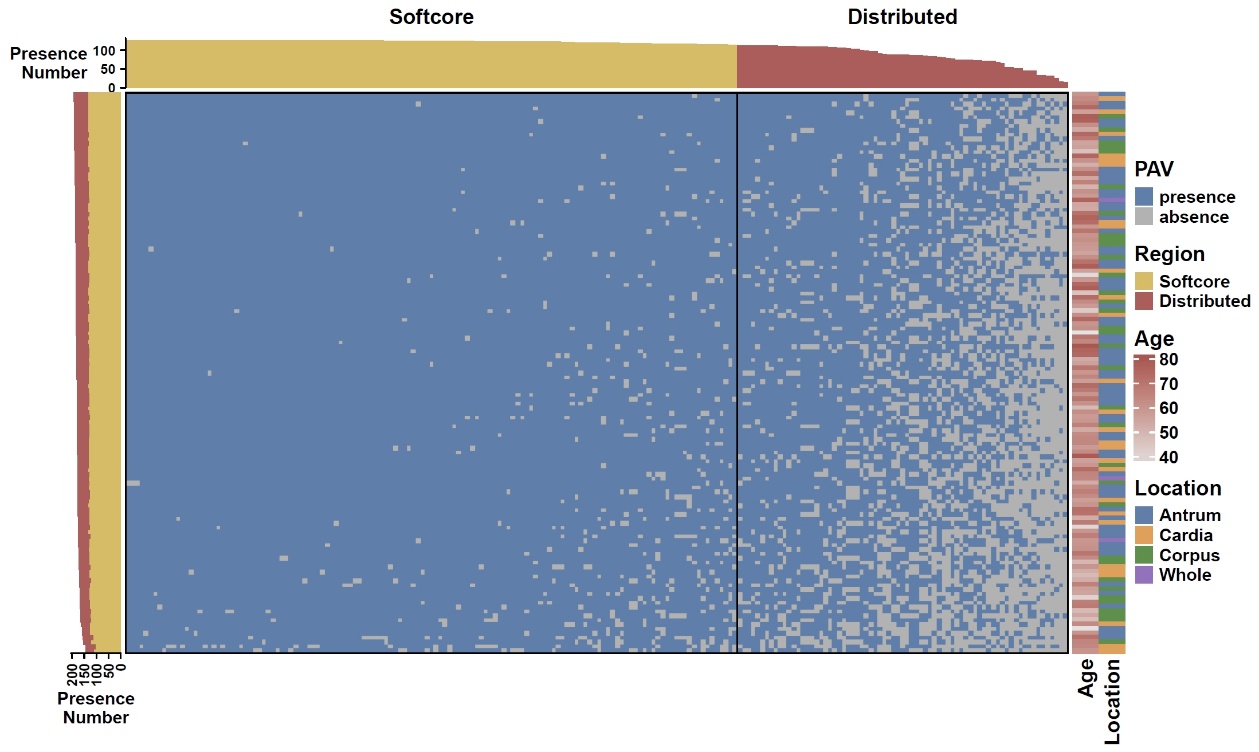
*

### **2.6 Phenotype Association Analysis**

Calculate associations between non-core genes and phenotypes:

| $ apav pavStaPheno --pav gcpan_dispensable.pav --pheno data/gc.pheno  *#Output: gcpan_dispensable.phenores* |
| --- |

| $ apav pavPlotPhenoHeat --pav gcpan_dispensable.pav --pheno data/gc.pheno \  --pheno_res gcpan_dispensable.phenores --only_show_significant --fig_height 5 --fig_width 7  *#Output: gcpan_dispensable_pheno_heatmap.pdf* |
| --- |


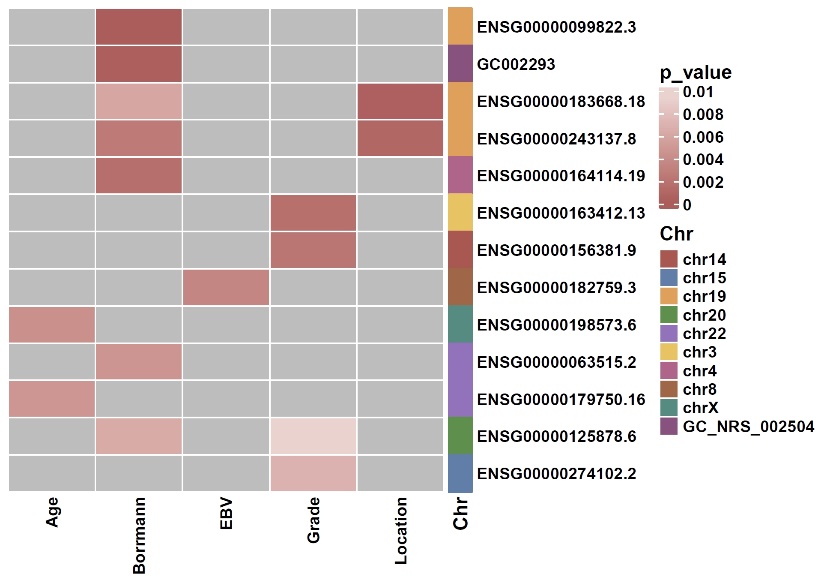


Visualize the association between a gene and a discrete phenotype (e.g., the GC002293 gene and Borrmann classification) in a bar plot:

| $ apav pavPlotPhenoBar --pav gcpan_all.pav --pheno data/gc.pheno \  --pheno_name Borrmann --region_name GC002293 --fig_height 4 --fig_width 3  *#Output: gcpan_all_pheno_Borrmann_GC002293_bar.pdf* |
| --- |


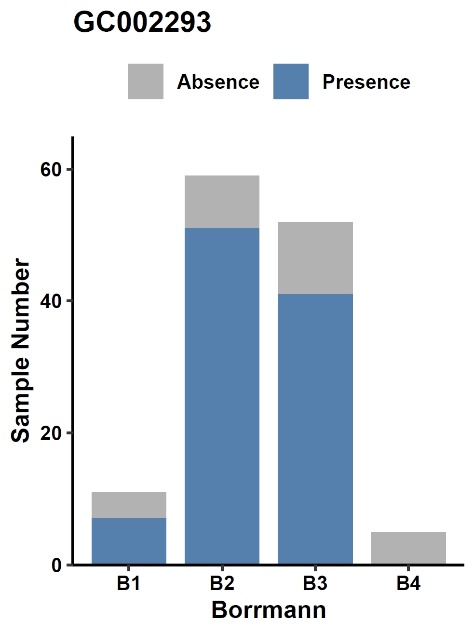


Visualize the association between gene PAV and a continuous phenotype (e.g., the ENSG00000198573.6 gene and age) in a violin plot:

| $ apav pavPlotPhenoVio --pav gcpan_all.pav --pheno data/gc.pheno \  --pheno_name Age --region_name ENSG00000198573.6 --fig_height 4 --fig_width 3  *#Output: gcpan_all_pheno_Age_ENSG00000198573.6_violin.pdf* |
| --- |

*
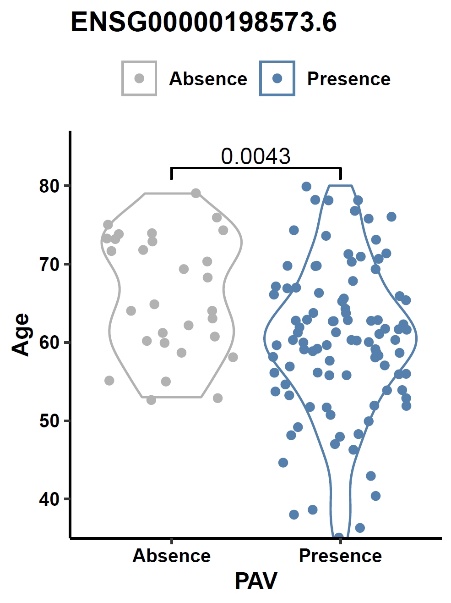
*

### **2.7 Element Visualization (*ACOT1*)**

Prepare annotation and data files:

| $ mkdir ele_example_acot1 && cd ele_example_acot1  $ grep 'ENSG00000184227.8' ../data/gcpan.gff3 > ENSG00000184227.8.gff  $ grep -E 'Annotation\|ENSG00000184227.8' ../gcpan_ele.cov > ENSG00000184227.8.elecov  $ grep -E 'Annotation\|ENSG00000184227.8' ../gcpan_ele_all.pav > ENSG00000184227.8.elepav  *#Output: ENSG00000184227.8.gff, ENSG00000184227.8.elecov, ENSG00000184227.8.elecov* |
| --- |

Plot coverage, PAV and depth:

| $ apav elePlotCov --elecov ENSG00000184227.8.elecov --gff ENSG00000184227.8.gff \  --cluster_samples --hide_sample_name --top_anno_height 0.25 --fig_width 7.5 --fig_height 6.5  $ apav elePlotPAV --elepav ENSG00000184227.8.elepav --gff ENSG00000184227.8.gff \  --cluster_samples --hide_sample_name --top_anno_height 0.25 --fig_width 7.5 --fig_height 6.5  $ apav elePlotDepth --ele ENSG00000184227.8.elecov \  --bamdir ../data/gc_bam/ --gff ENSG00000184227.8.gff --cluster_samples \  --hide_sample_name --log10 --top_anno_height 0.25 --fig_width 7.5 --fig_height 5.5  $ cd ..  *#Output: ENSG00000184227.8.elecov.pdf, ENSG00000184227.8.elepav.pdf, ENSG00000184227.8.elecov.depth.pdf* |
| --- |


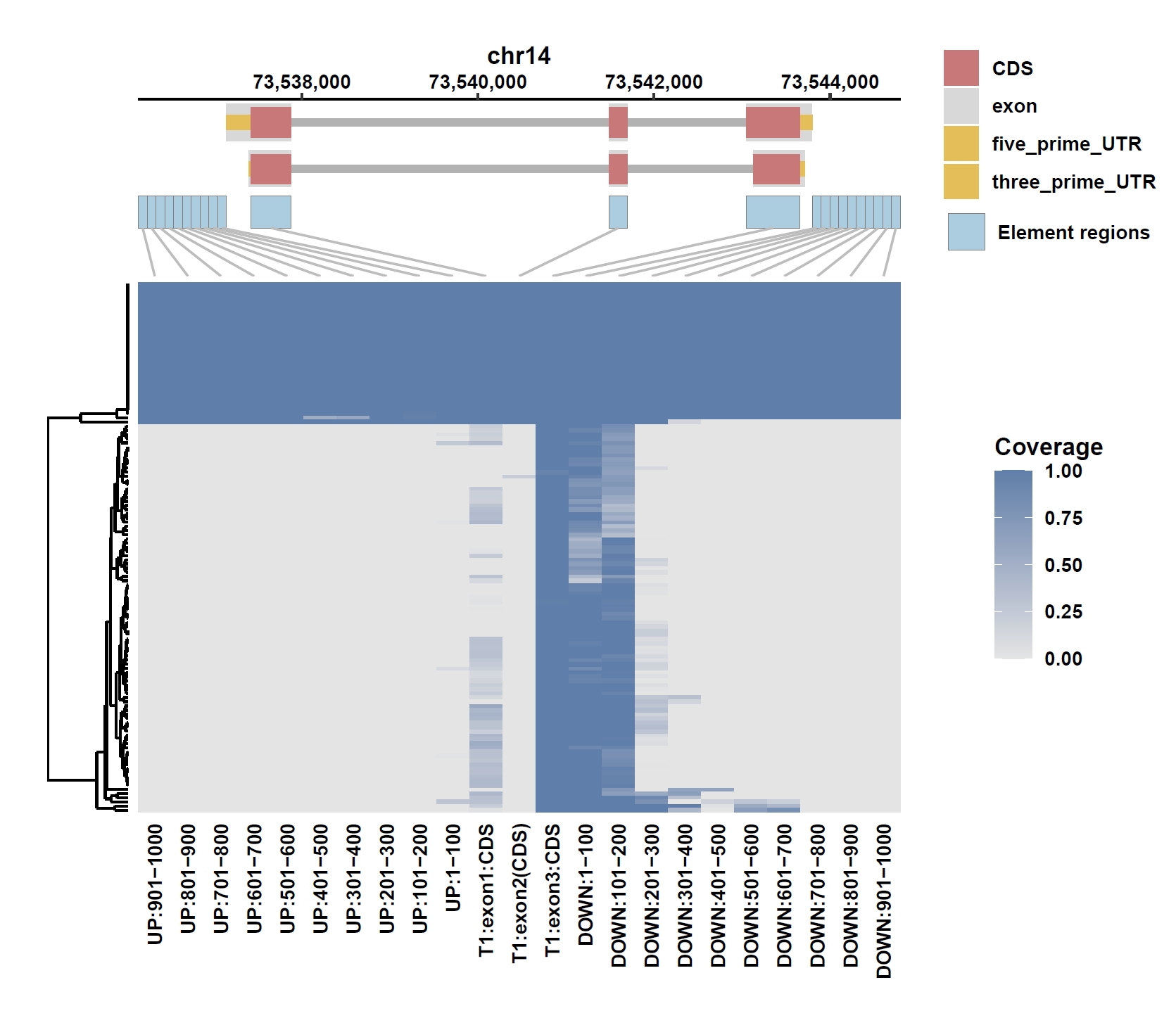

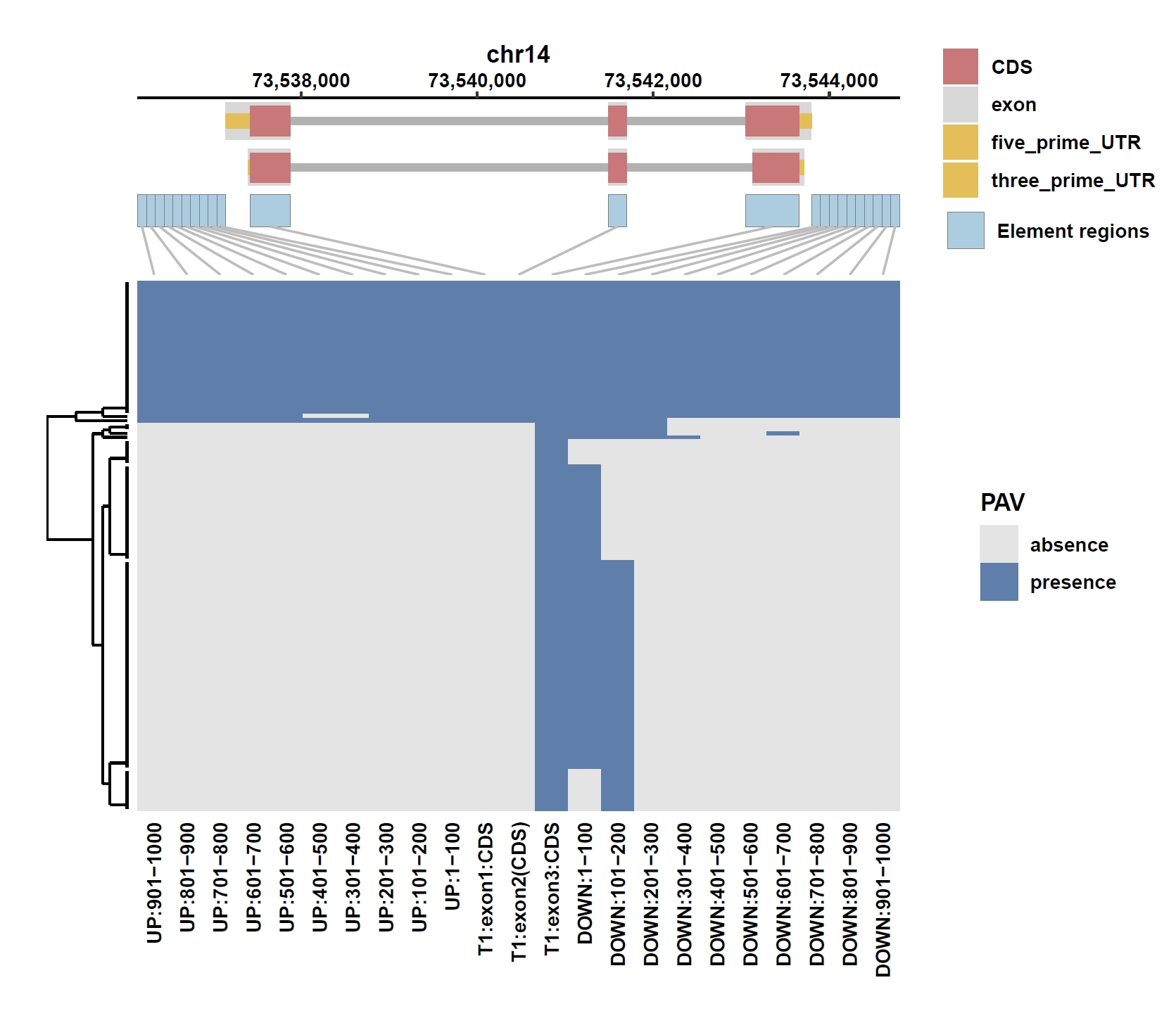

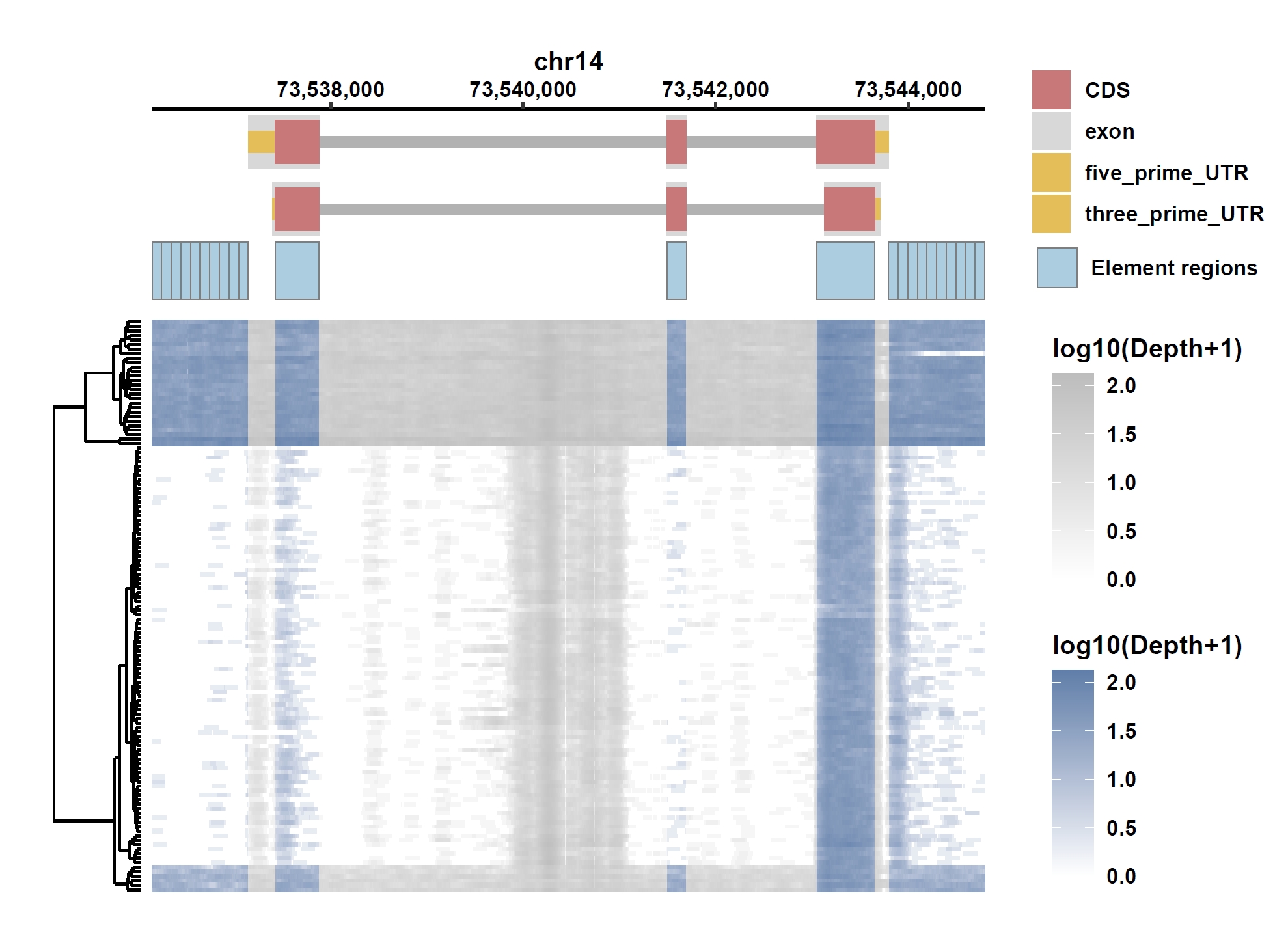


### **2.8 Element Visualization (*PIM3*)**

Prepare annotation and data files:

| $ mkdir ele_example_pim3 && cd ele_example_pim3  $ grep 'ENSG00000198355.5' ../data/gcpan.gff3 > ENSG00000198355.5.gff  $ grep -E 'Annotation\|ENSG00000198355.5' ../gcpan_ele.cov > ENSG00000198355.5.elecov  $ grep -E 'Annotation\|ENSG00000198355.5' ../gcpan_ele_all.pav > ENSG00000198355.5.elepav  *#Output: ENSG00000198355.5.gff, ENSG00000198355.5.elecov, ENSG00000198355.5.elecov* |
| --- |

Plot coverage, PAV and depth:

| $ apav elePlotCov --elecov ENSG00000198355.5.elecov --gff ENSG00000198355.5.gff \  --cluster_samples --hide_sample_name --top_anno_height 0.25 --fig_width 7.5 --fig_height 6.5  $ apav elePlotPAV --elepav ENSG00000198355.5.elepav --gff ENSG00000198355.5.gff \  --cluster_samples --hide_sample_name --top_anno_height 0.25 --fig_width 7.5 --fig_height 6.5  $ apav elePlotDepth --ele ENSG00000198355.5.elecov \  --bamdir ../data/gc_bam/ --gff ENSG00000198355.5.gff --cluster_samples \  --hide_sample_name --log10 --top_anno_height 0.25 --fig_width 7.5 --fig_height 5.5  *#Output: ENSG00000198355.5.elecov.pdf, ENSG00000198355.5.elepav.pdf, ENSG00000198355.5.elecov.depth.pdf* |
| --- |


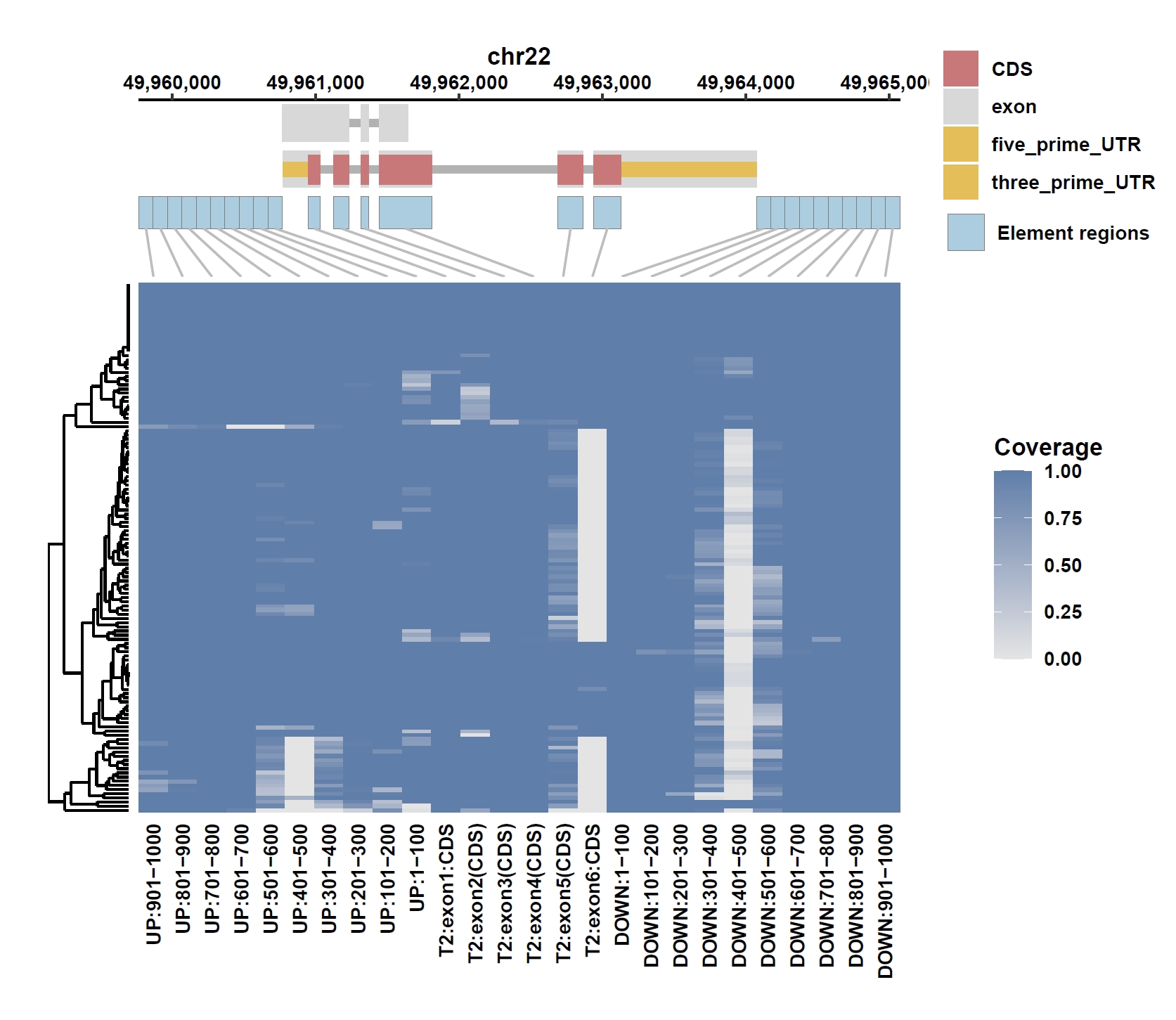

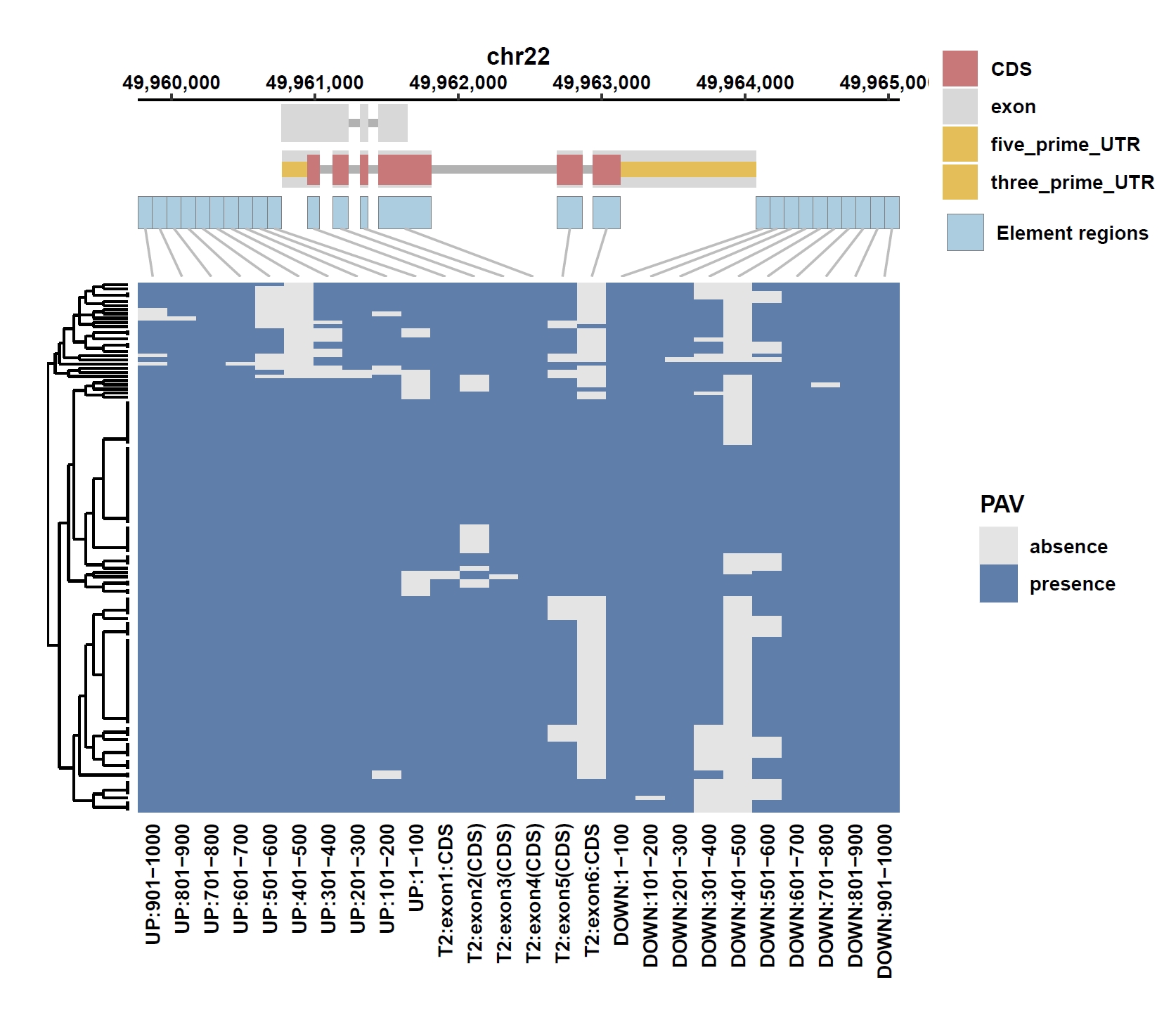

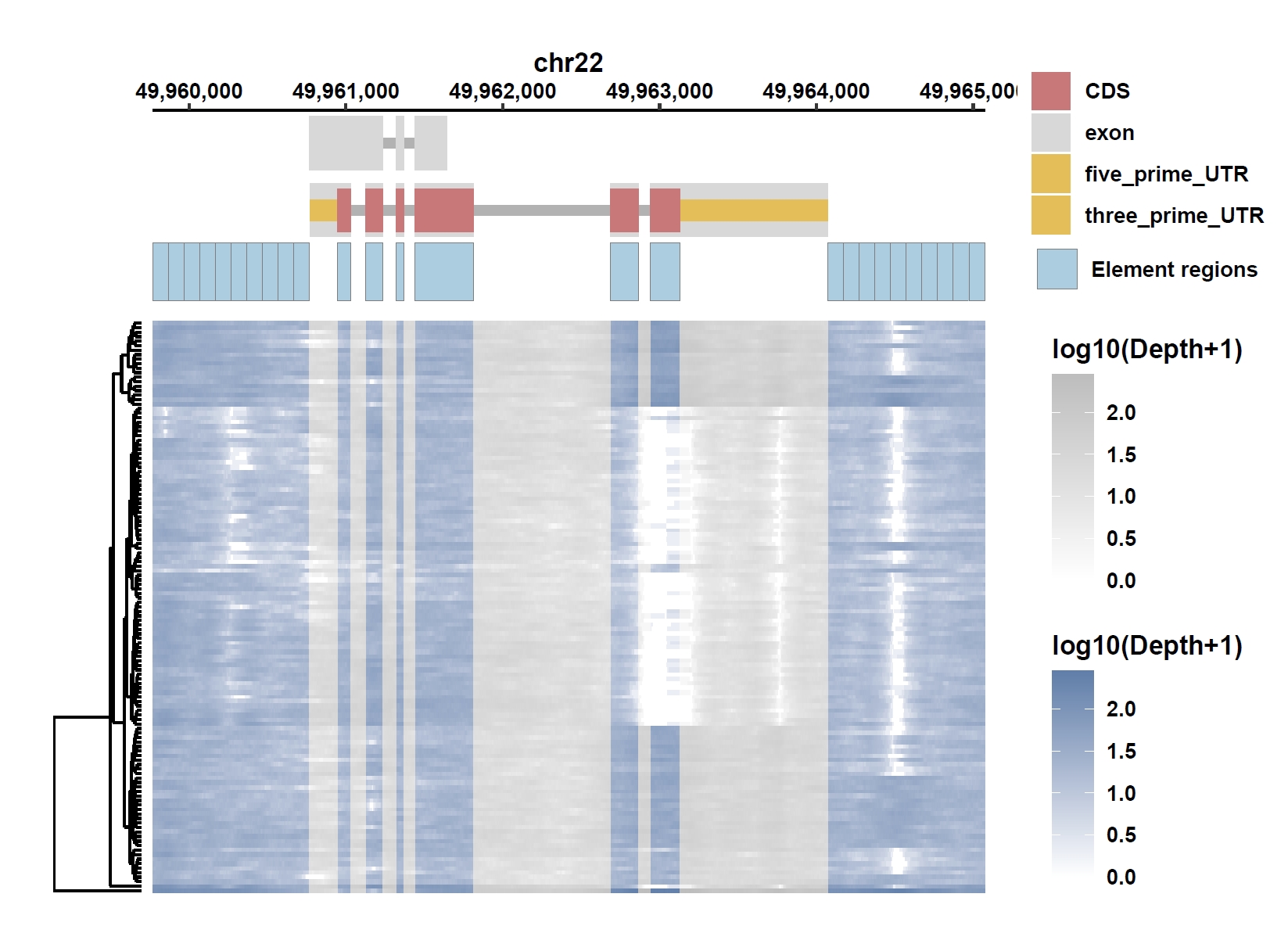


View phenotype association with phenotypes for gene and exons:

| $ apav pavPlotPhenoBar --pav ../gcpan_all.pav --pheno ../data/gc.pheno \  --pheno_name EBV --region_name ENSG00000198355.5 \  --fig_width 4 --fig_height 4 --out ENSG00000198355.5_ebv  $ apav pavPlotPhenoBar --pav ENSG00000198355.5.elepav --pheno ../data/gc.pheno \  --pheno_name EBV --region_name 'ENSG00000198355.5:[T2:exon6:CDS]' \  --fig_width 4 --fig_height 4 --out ENSG00000198355.5_ebv_exon6  $ apav pavPlotPhenoBar --pav ENSG00000198355.5.elepav --pheno ../data/gc.pheno \  --pheno_name HP --region_name 'ENSG00000198355.5:[UP:501-600]' \  --fig_width 4 --fig_height 4 --out ENSG00000198355.5_hp_up  $ apav pavPlotPhenoBar --pav ENSG00000198355.5.elepav --pheno ../data/gc.pheno \  --pheno_name Borrmann --region_name 'ENSG00000198355.5:[DOWN:401-500]' \  --fig_width 4 --fig_height 4 --out ENSG00000198355.5_borrmann_down  $ cd ..  *#Output: ENSG00000198355.5.elecov.pdf, ENSG00000198355.5.elepav.pdf, ENSG00000198355.5.elecov.depth.pdf* |
| --- |


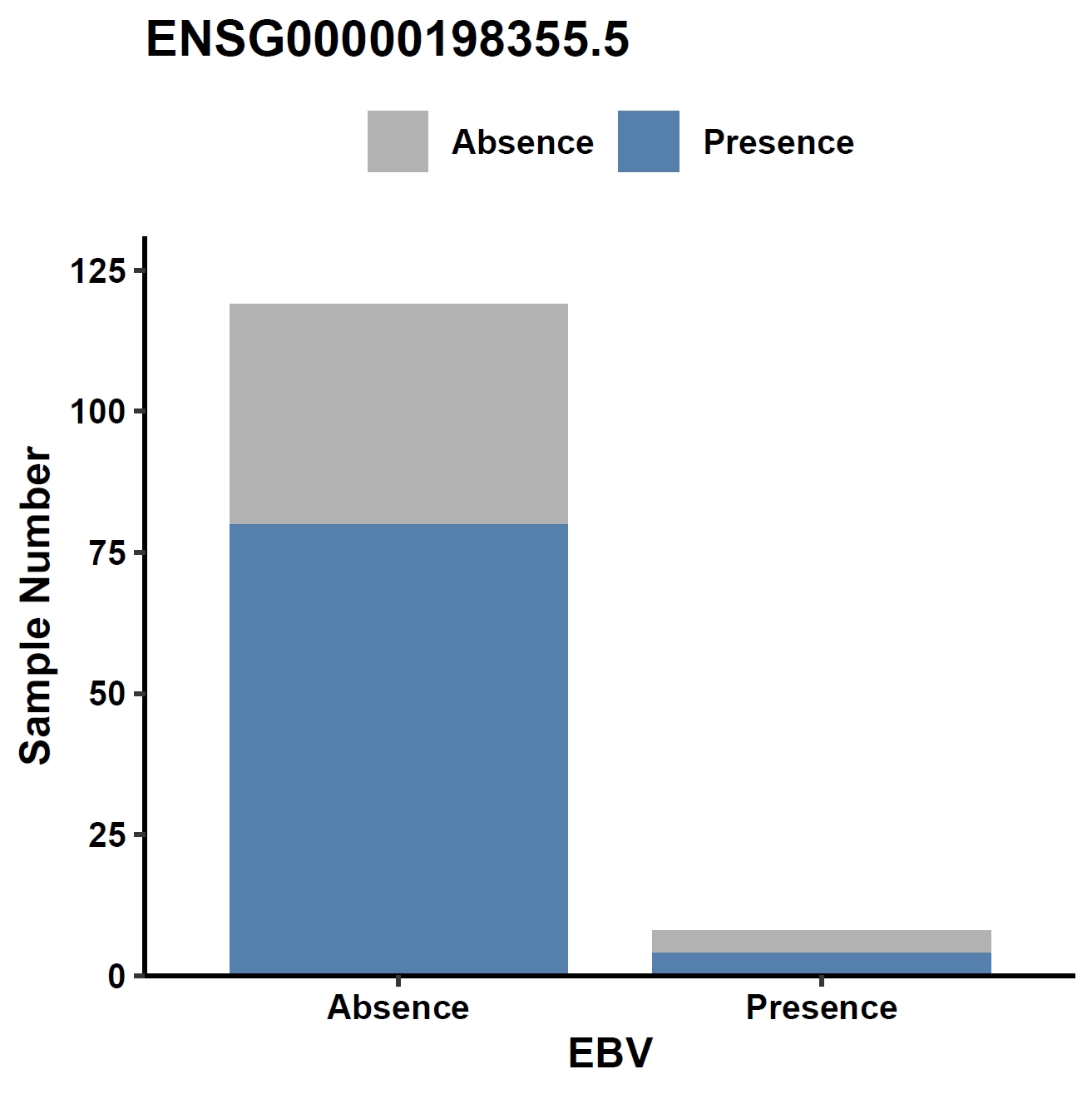

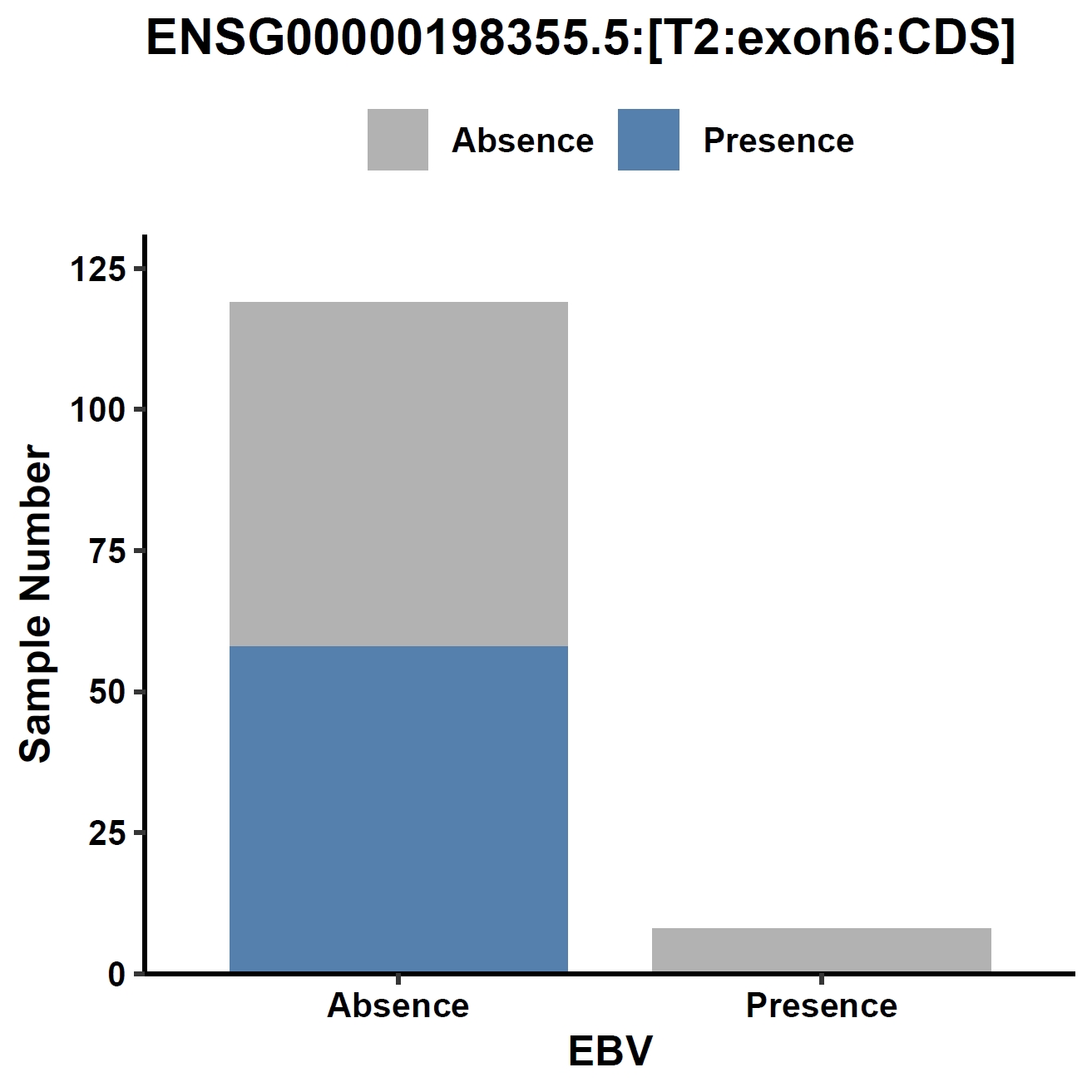

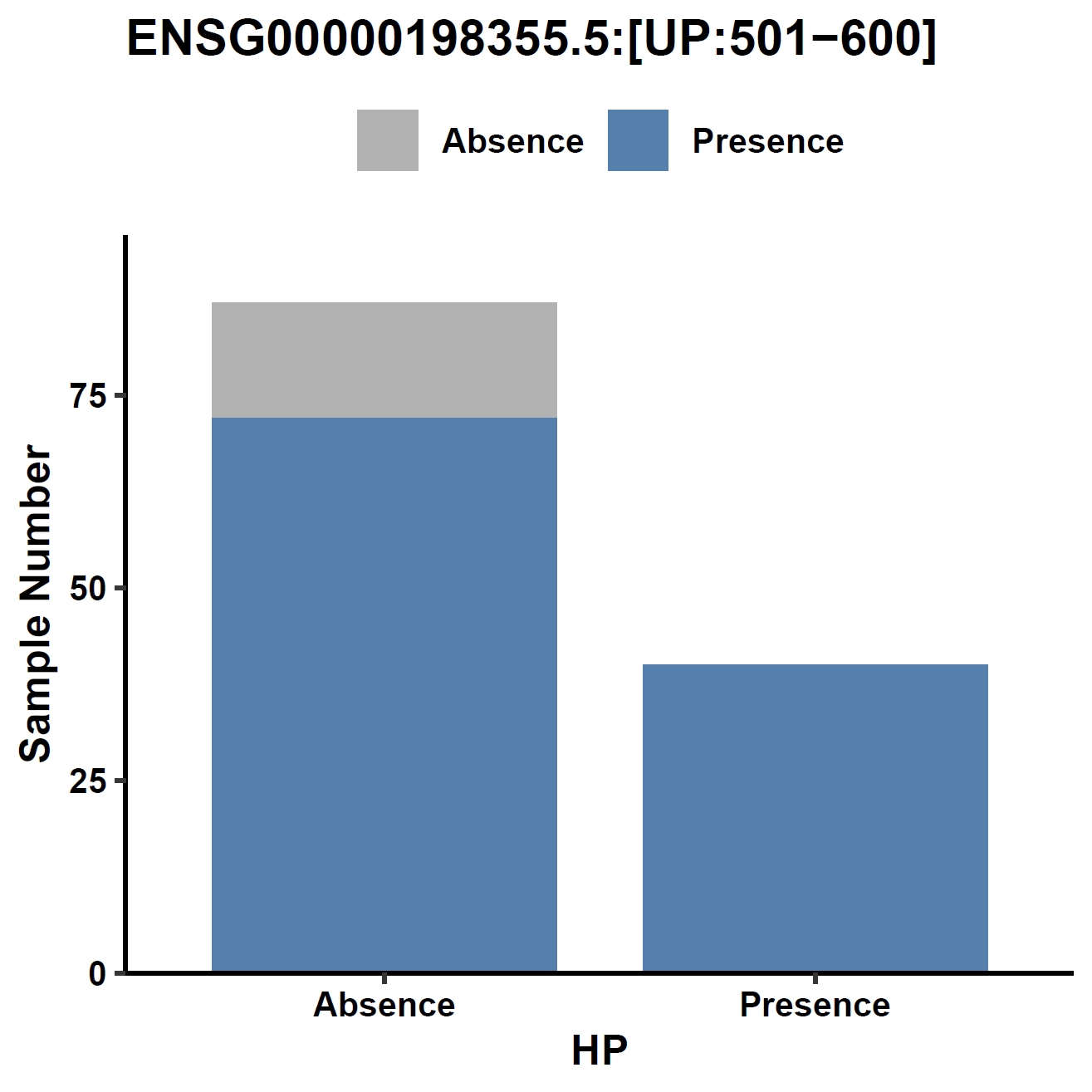

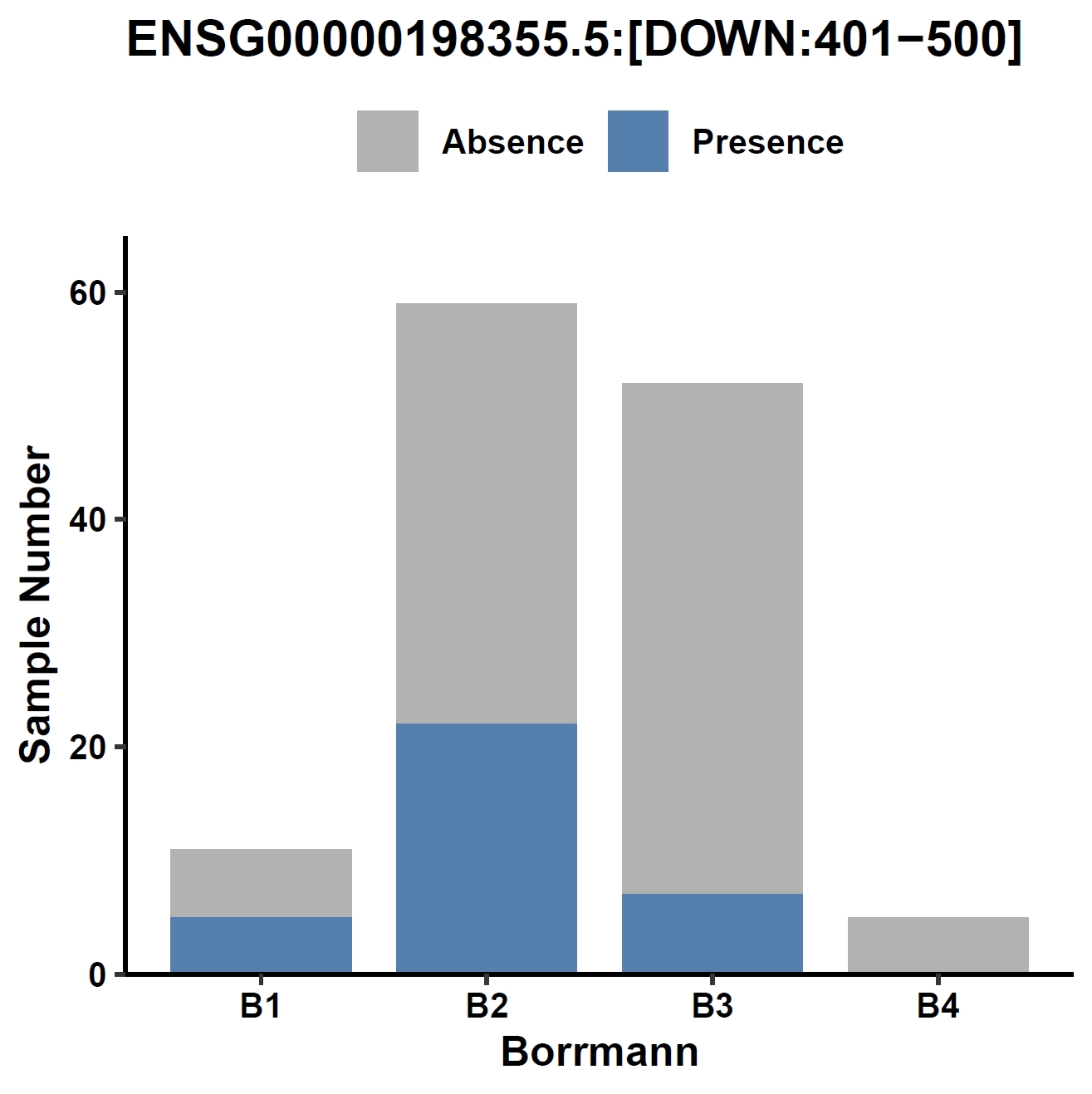


### **Run time**

The computational experiments were performed on an x86-64 server equipped with dual Intel Xeon Gold 6248 processors.

- 1 CPU core, 4GB RAM
- Samtools version: 1.16.1
- R version: 4.3.2

| **Step** | **Input data** | **Run time** |
| --- | --- | --- |
| gff2bed | gcpan.gff3(1.2G) | 21.6s |
| staCov | 127 BAM files and index files (11.94TB), gcpan.bed(113M) | 120h 7min (~1h 9min for one sample) |
| callPAV | gcpan.cov(9.1M) | 2.9s |
| callPAV | gcpan_ele.cov(181M) | 40.0s |
| pavSize | gcpan_all.pav(8.9M) | 1.9s |
| pavPlotSize | gcpan_grouped.size(331K) | 5.0s |
| pavPlotHist | gcpan_all.pav(8.9M) | 6.0s |
| pavPlotStat | gcpan_all.pav(8.9M) | 5.7s |
| pavPlotHeat | gcpan_dispensable.pav(76K) | 8.6s |
| pavStaPheno | gcpan_dispensable.pav(76K), gc.pheno(4.3K) | 5.3s |
| pavPlotPhenoHeat | gcpan_dispensable.phenores(54K) | 5.0s |
| pavPlotPhenoBar | gcpan_all.pav(8.9M), gc.pheno(4.3K) | 5.7s |
| pavPlotPhenoVio | gcpan_all.pav(8.9M), gc.pheno(4.3K) | 5.9s |
| elePlotCov, elePlotPAV, elePlotDepth | ENSG00000184227.8.elecov(9.2K), ENSG00000184227.8.elepav(8.4K) | 5.5s, 5.4s, 25.1s |
| elePlotCov, elePlotPAV, elePlotDepth | ENSG00000198355.5.elecov(11K), ENSG00000198355.5.elepav(9.3K) | 5.5s, 5.4s, 22.5s |
