# Supplementary material for: APAV: An advanced pangenome analysis and visualization toolkit
Source: PLoS Comput Biol. 2025 Jul 7;21(7):e1013288. doi: 10.1371/journal.pcbi.1013288 (PMC12251200; doi:10.1371/journal.pcbi.1013288)
Supplement: S1 Fig — (DOCX) [file pcbi.1013288.s004.docx]

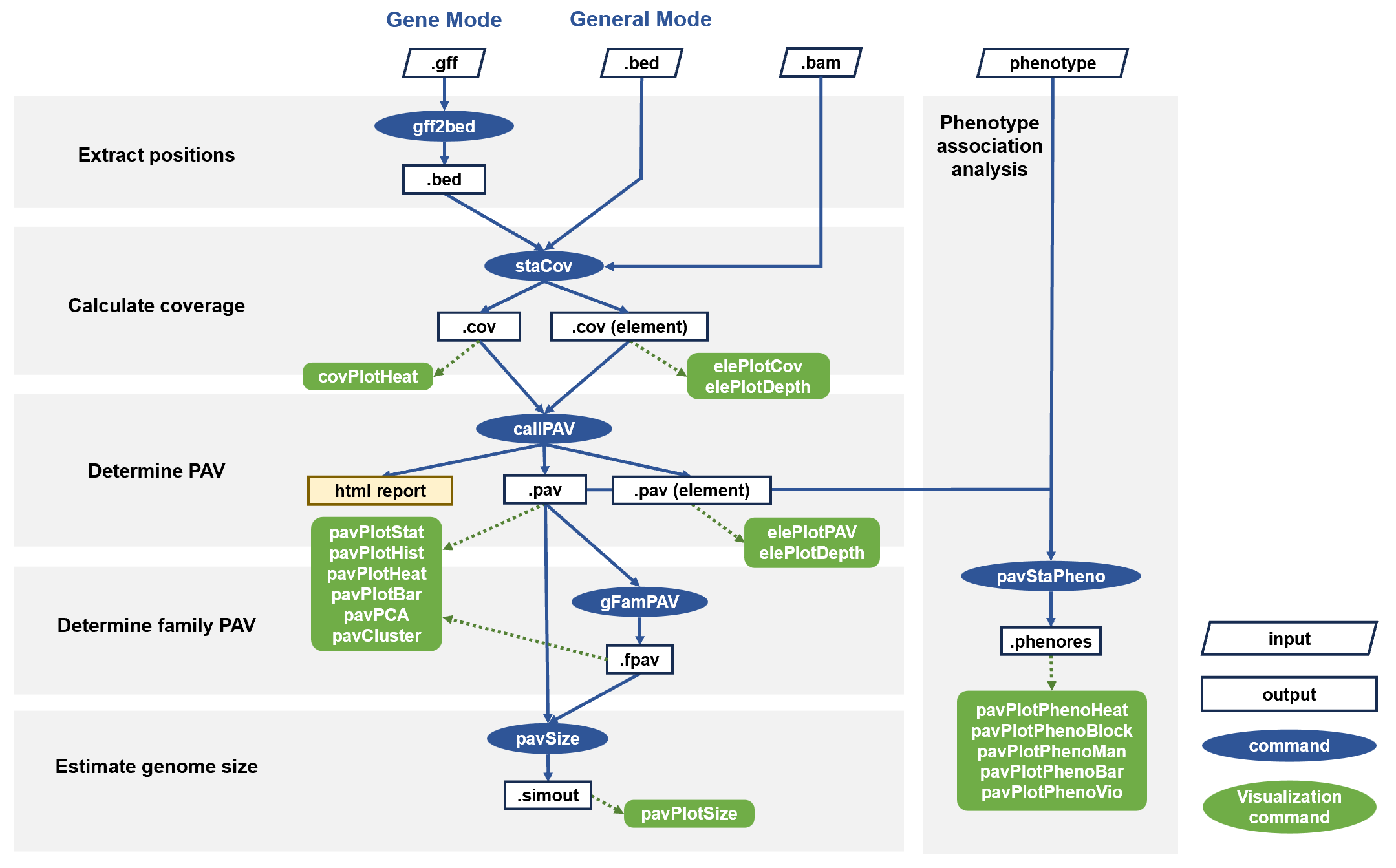


**S1 Fig. The workflow of APAV.** The blue commands represent the main steps of the analysis and calculation, while the green commands are the visualization tools. Input files include a GFF file (for gene structures), a BED file (for arbitrary genomic regions), BAM files (for read alignment results), and a table of phenotypic information (for genotype-phenotype association study). The GFF or BED file is used to define the regions for statistical analysis. The “*staCov*” command computes the coverage based on the BAM files within the regions defined by the GFF or BED file. The “*callPAV*” command determines PAV based on the coverage data. The “*gFamPAV*” command determines the PAV information for gene families. The “*pavSize*” command estimates the genome size. The “*pavStaPheno*” command performs phenotypic association analysis. The results from each analysis step are accompanied by corresponding visualization methods.
