# Supplementary material for: APAV: An advanced pangenome analysis and visualization toolkit
Source: PLoS Comput Biol. 2025 Jul 7;21(7):e1013288. doi: 10.1371/journal.pcbi.1013288 (PMC12251200; doi:10.1371/journal.pcbi.1013288)
Supplement: S2 Fig — (DOCX) [file pcbi.1013288.s005.docx]

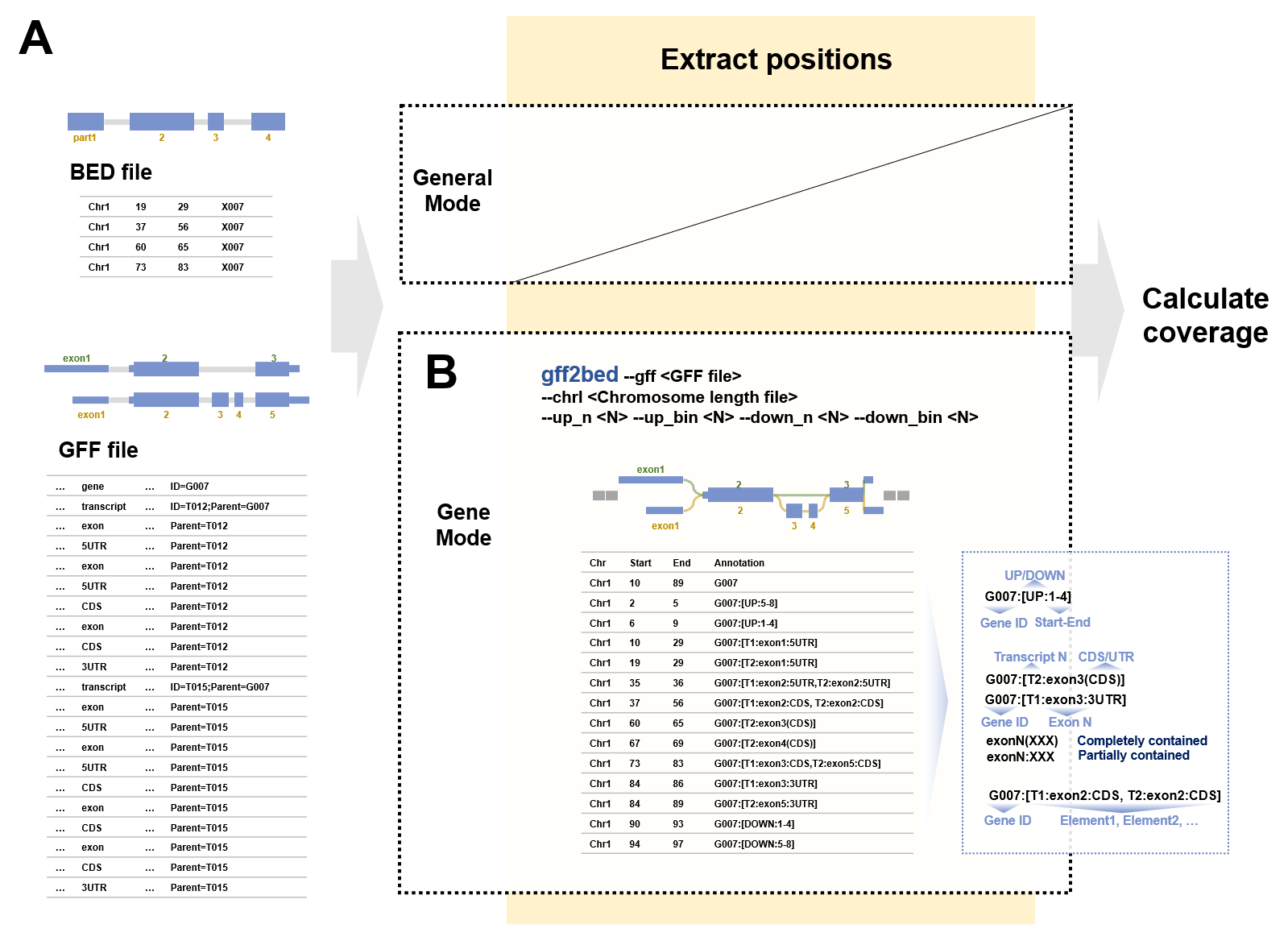


**S2 Fig. Schematic diagram of the “*gff2bed*” command. (A) Examples of input file formats.** The BED file is used for arbitrary regions in general mode. The GFF file records annotation information for genes. **(B) Overview of the parameters for the “*gff2bed*” command, along with the output generated from the example data.** The diagonal line in the figure indicates that this step is skipped in the general mod. The blue dashed box shows the format of the gene element annotations. These annotations consist of two parts: the gene ID and the element list. Elements in the list are separated by commas, with each element detailing the transcript number, exon number, and the respective CDS or UTR category. Colons in the element annotations signify inclusion relationships, while parentheses indicate full inclusion.
