# Supplementary material for: APAV: An advanced pangenome analysis and visualization toolkit
Source: PLoS Comput Biol. 2025 Jul 7;21(7):e1013288. doi: 10.1371/journal.pcbi.1013288 (PMC12251200; doi:10.1371/journal.pcbi.1013288)
Supplement: S3 Fig — (DOCX) [file pcbi.1013288.s006.docx]

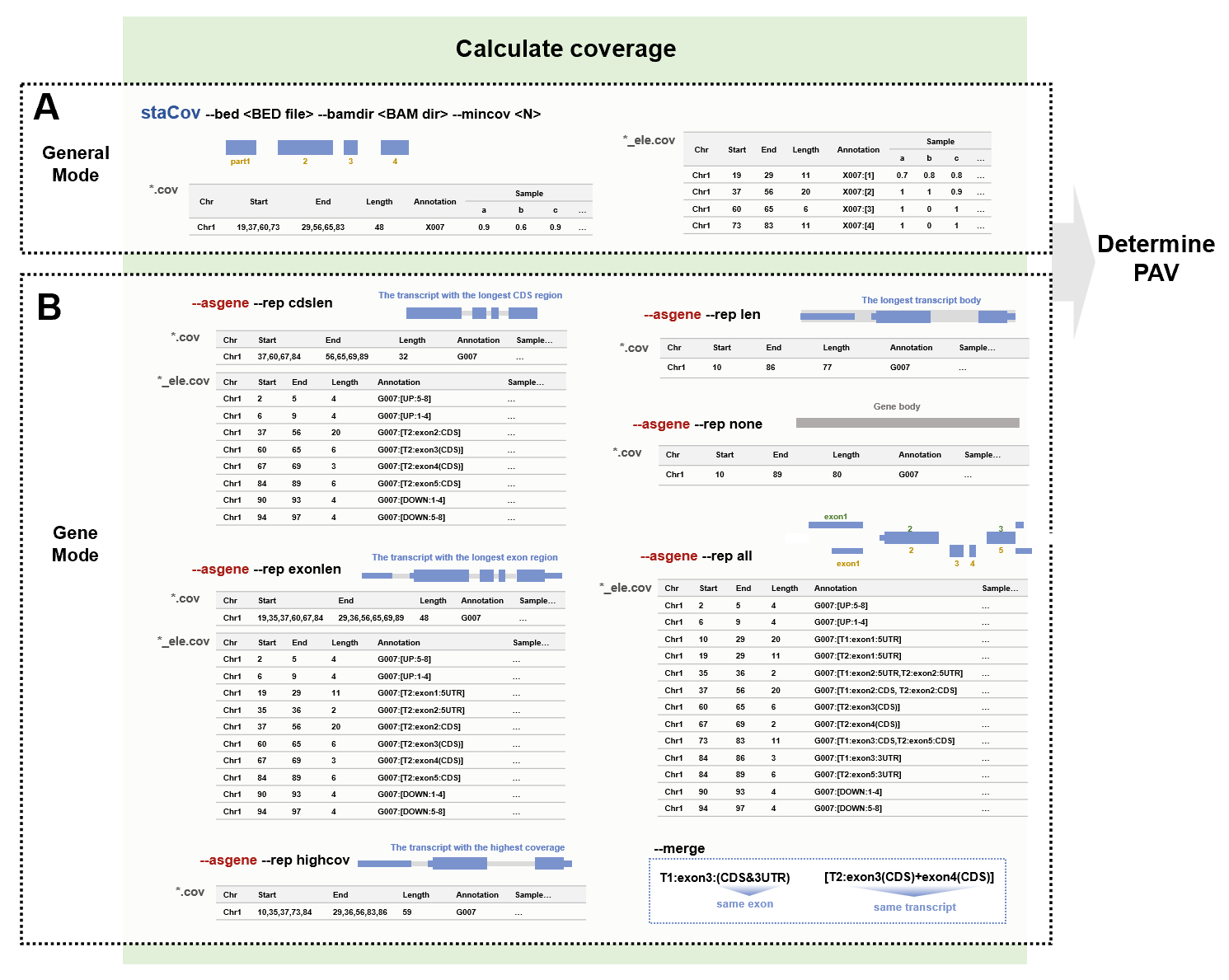


**S3 Fig. Description of the main parameters of the “*staCov*” command.**

**(A) Key parameters in general mode.** The user must provide the BED file and the directory containing the BAM file, and specify the coverage threshold (default value is 0.5). After running the command, two types of files will be generated. The file with suffixes “.cov” records the coverage of the whole region, while the file with suffixes “_ele.cov” specifically records the coverage of the elements. The results presented in the table within the figure are based on the example data from Supplementary Figure 2. **(B) Key parameters in gene mode.** Similarly, the user must pass in the BED file (which can be generated using the “*gff2bed*” command), the directory containing the BAM file, and the coverage threshold (default value is also 0.5). Additionally, the “--asgene” parameter must be included to specify the target region to be treated as a gene. The “--rep” parameter allows for the selection of the region to be calculated. The “cdslen” option selects the transcript with the longest coding sequence (CDS) region, while “exonlen” selects the longest transcript in the exon region. The “highcov” option identifies the transcript with the highest coverage. The “len” option selects the longest transcript body, while ‘none’ indicates the selection of the gene body. The “all” option calculates the coverage of all elements without outputting the entire region.
