# Supplementary material for: APAV: An advanced pangenome analysis and visualization toolkit
Source: PLoS Comput Biol. 2025 Jul 7;21(7):e1013288. doi: 10.1371/journal.pcbi.1013288 (PMC12251200; doi:10.1371/journal.pcbi.1013288)
Supplement: S4 Fig — (DOCX) [file pcbi.1013288.s007.docx]

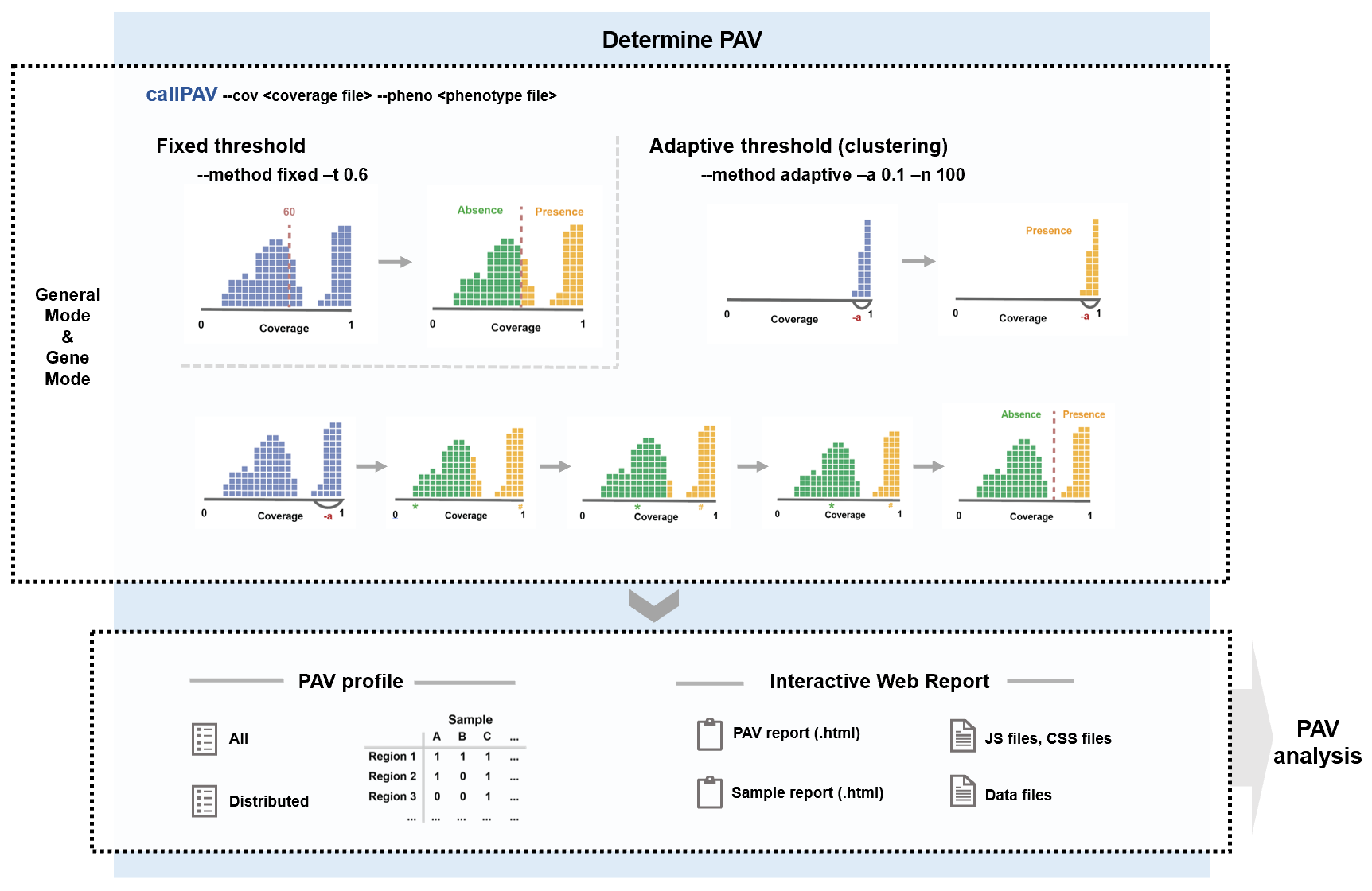


**S4 Fig. Description of the parameters of the “*callPAV*” command.** The “--cov” parameter is used to input the coverage file. The “--method” parameter allows users to select the method for determining PAV. In the fixed threshold method, the “-t” parameter sets the coverage threshold: values above this threshold are classified as present, while those below are classified as absent. In the flexible threshold method, if none of the samples exceed the value specified by the “-a” parameter, all samples are classified as present. Otherwise, clustering is performed to differentiate between the presence and absence groups. The phenotype data specified by the “--pheno” parameter is used for the sample report. Data from the “--fa”, “--gff” and “--bamdir” parameters are displayed in the Genome Browser in the PAV report.
