# Supplementary material for: APAV: An advanced pangenome analysis and visualization toolkit
Source: PLoS Comput Biol. 2025 Jul 7;21(7):e1013288. doi: 10.1371/journal.pcbi.1013288 (PMC12251200; doi:10.1371/journal.pcbi.1013288)
Supplement: S5 Fig — (DOCX) [file pcbi.1013288.s008.docx]

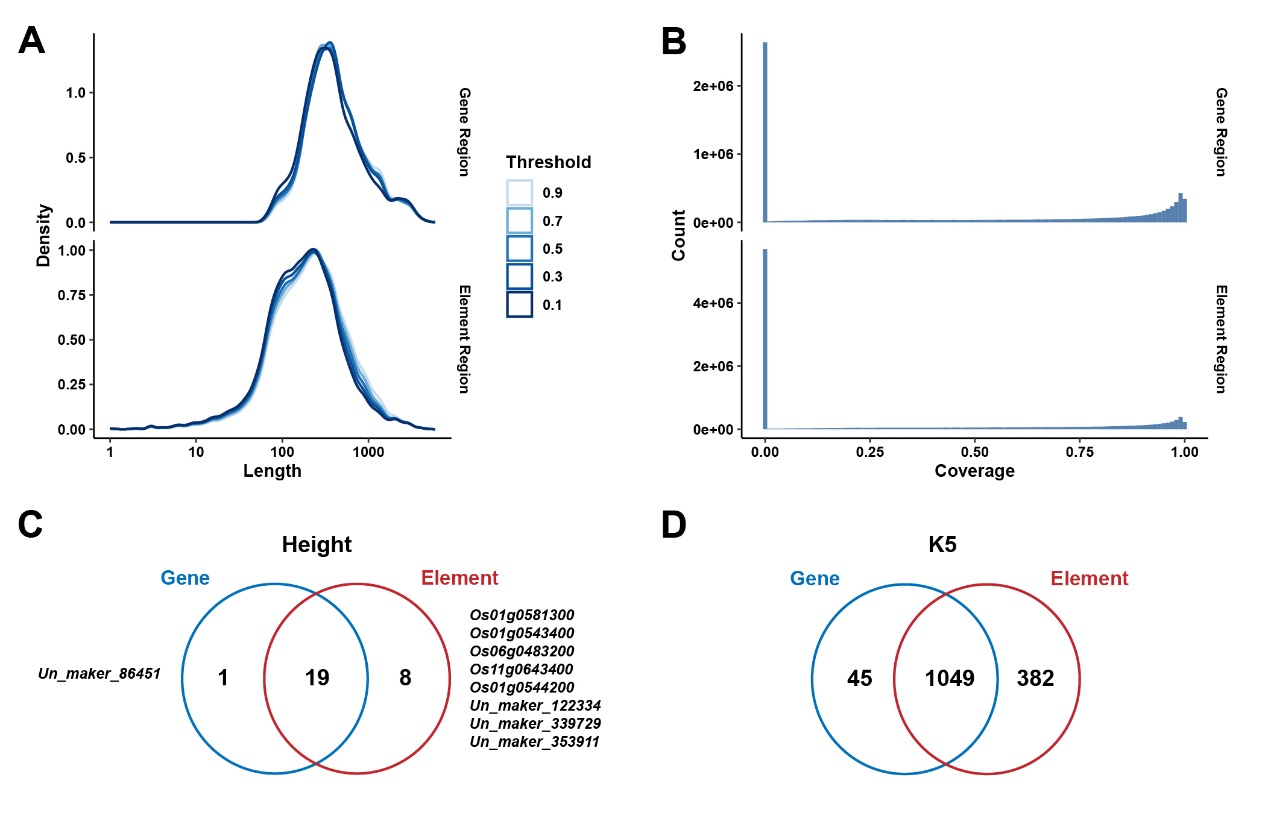


**S5 Fig. Comparison of results at the gene level and element level in the rice genomes. (A) Length distribution of dispensable regions.** The colors indicate the thresholds used to determine PAV and dispensable regions. **(B) Histogram of coverage for regions with less than 100% coverage.** **(C, D) Comparison of phenotypic association results at the gene level and element level.** A threshold of 0.5 was applied to determine PAV, and results with adjusted p-values less than 1e-5 were filtered out.
