# Supplementary material for: APAV: An advanced pangenome analysis and visualization toolkit
Source: PLoS Comput Biol. 2025 Jul 7;21(7):e1013288. doi: 10.1371/journal.pcbi.1013288 (PMC12251200; doi:10.1371/journal.pcbi.1013288)
Supplement: S6 Fig — (DOCX) [file pcbi.1013288.s009.docx]

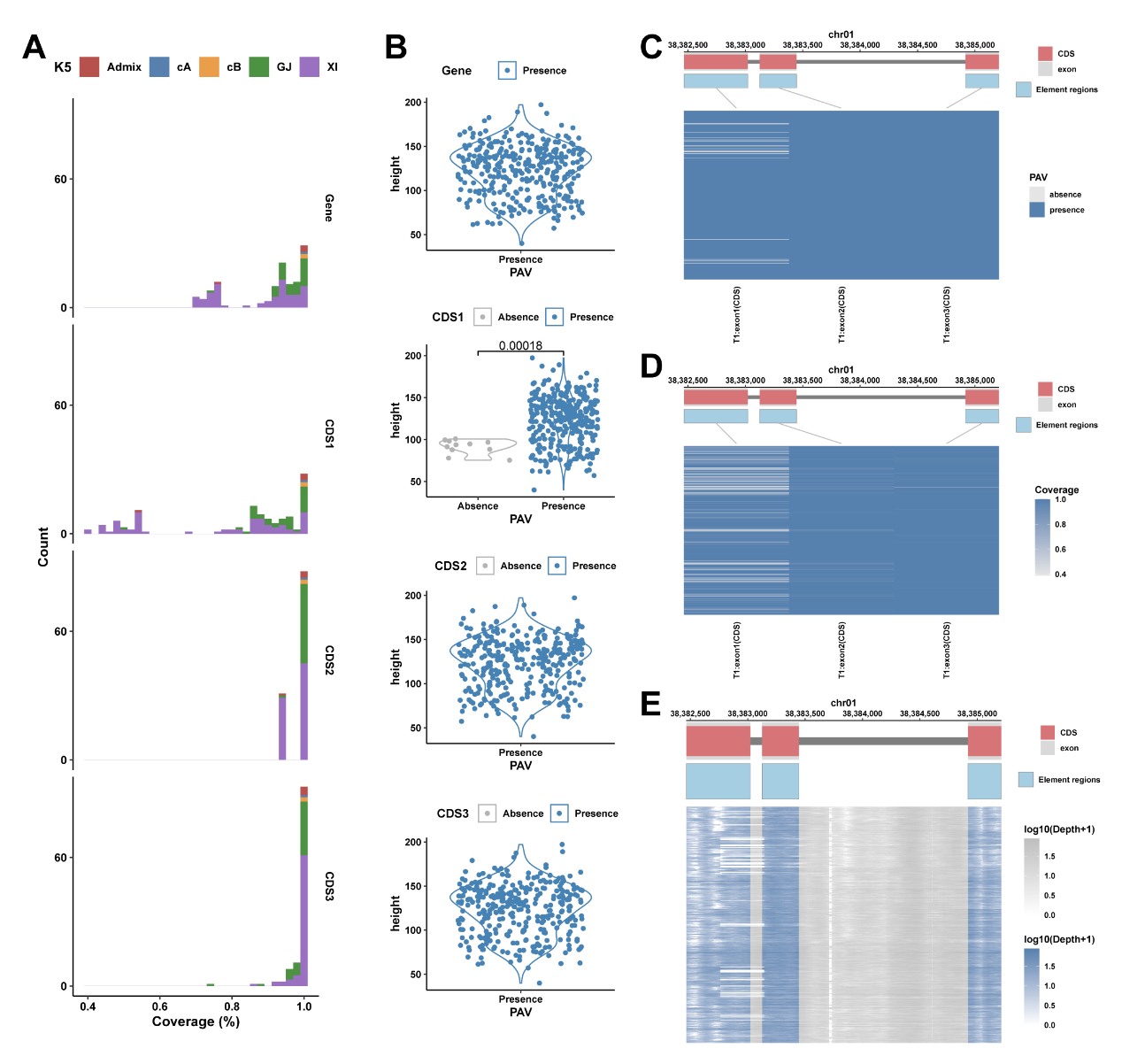


**S6 Fig. Element-level analyses for *sd1*(*Os01g0883800*) gene. (A)** Coverage distribution of the gene and all CDSs. **(B)** **The height of samples in both the presence and absence groups for the gene and its elements.** A threshold of 0.5 was used to determine PAV. The correlation was observed between the first CDS and plant height (*p*=0.00018, Wilcox test). **(C-E) Element level PAV in terms of presence/absence, coverage, and sequencing read depth of elements in the *sd1* gene.**
