# Supplementary material for: APAV: An advanced pangenome analysis and visualization toolkit
Source: PLoS Comput Biol. 2025 Jul 7;21(7):e1013288. doi: 10.1371/journal.pcbi.1013288 (PMC12251200; doi:10.1371/journal.pcbi.1013288)
Supplement: S7 Fig — (DOCX) [file pcbi.1013288.s010.docx]

**
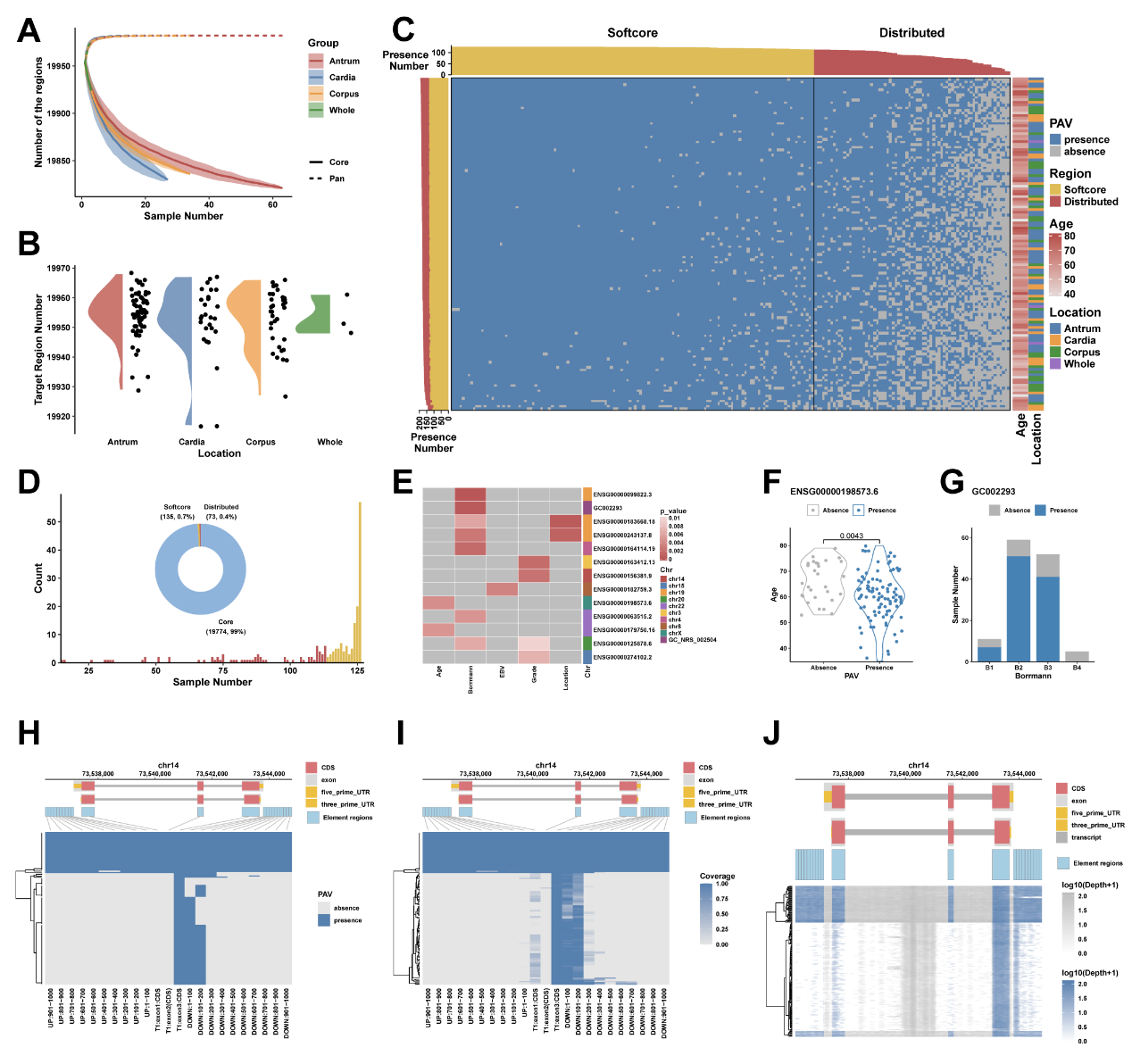
**

**S7 Fig. Visualization of pangenome analysis results in tumor genomes. (A) Pangenome size estimation.** The pangenome and core-genome sizes are drawn for different groups of individuals. **(B)** **Distribution of the numbers of targeted genomic regions**. **(C)** **Heatmap of gene PAV profile**. Each row of the heatmap is for a sample and each column is for a gene. **(D)** **Proportion and distribution of core, softcore, and distributed genes**. The proportion is shown in the pie chart and the distribution of gene numbers is shown in the bar plot. **(E) Results of phenotype and gene PAV association analysis.** **(F, G) An example of the relationship between a phenotype and a gene PAV. (H, I and J)** **Element level PAV in terms of Presence/absence, coverage, and sequencing read depth.** Here, every 100bp upstream and downstream region is shown as an element. Compared to the two PAV analysis methods (H and I), the sequencing read mapping depth(J) is the most sensitive way for element PAV analysis.
