# Supplementary material for: APAV: An advanced pangenome analysis and visualization toolkit
Source: PLoS Comput Biol. 2025 Jul 7;21(7):e1013288. doi: 10.1371/journal.pcbi.1013288 (PMC12251200; doi:10.1371/journal.pcbi.1013288)
Supplement: S8 Fig — (DOCX) [file pcbi.1013288.s011.docx]

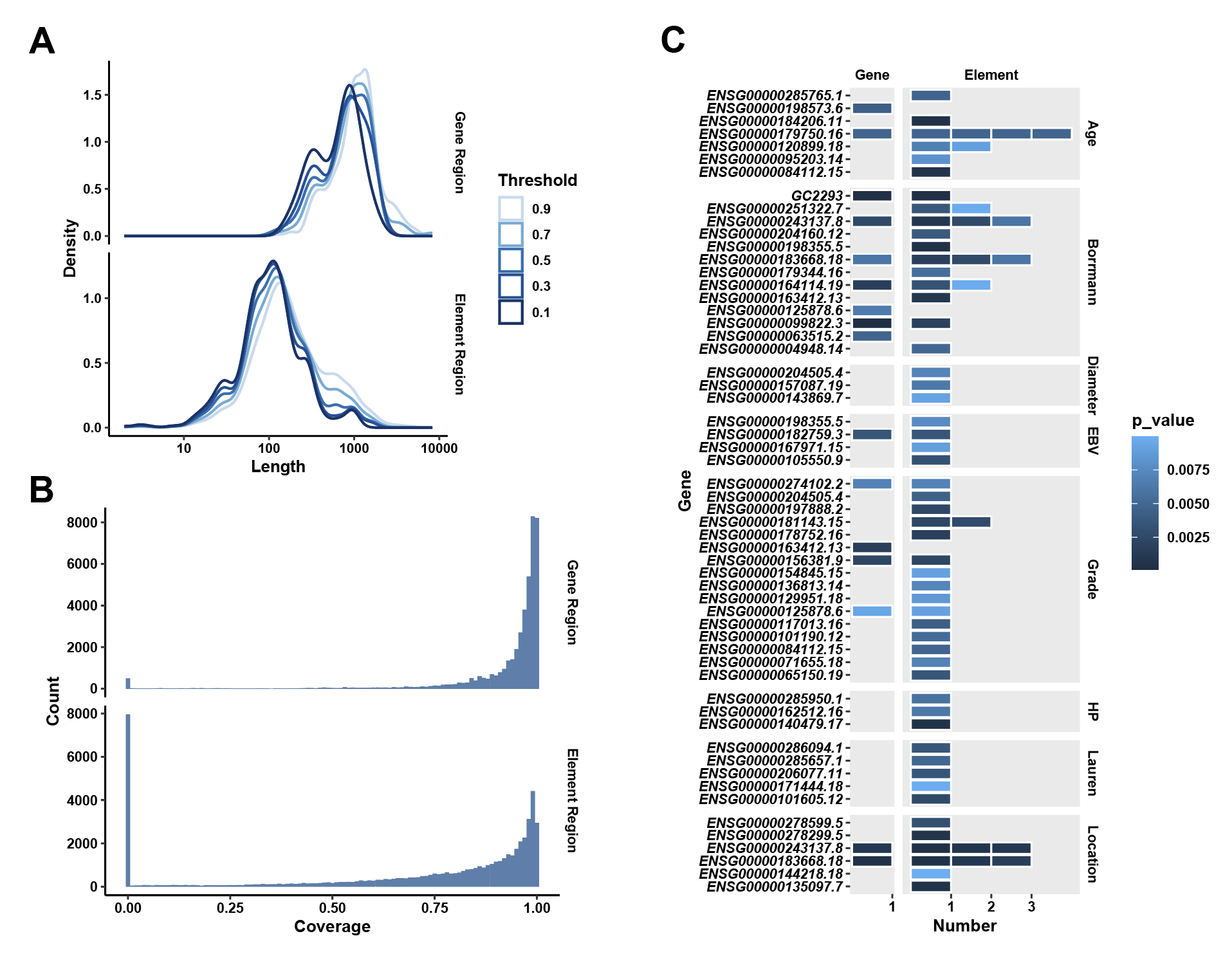


**S8 Fig. Comparison of results at the gene level and element level in the tumor genomes. (A) Length distribution of dispensable regions.** The colors indicate the thresholds used to determine PAV and dispensable region. **(B) Histogram of coverage for regions with less than 100% coverage.** **(C, D) Comparison of phenotypic association results at the gene level and element level.** A threshold of 0.8 was applied to determine PAV, and results with p-values less than 0.01 were filtered out.
