# Supplementary material for: APAV: An advanced pangenome analysis and visualization toolkit
Source: PLoS Comput Biol. 2025 Jul 7;21(7):e1013288. doi: 10.1371/journal.pcbi.1013288 (PMC12251200; doi:10.1371/journal.pcbi.1013288)
Supplement: S9 Fig — (DOCX) [file pcbi.1013288.s012.docx]

**
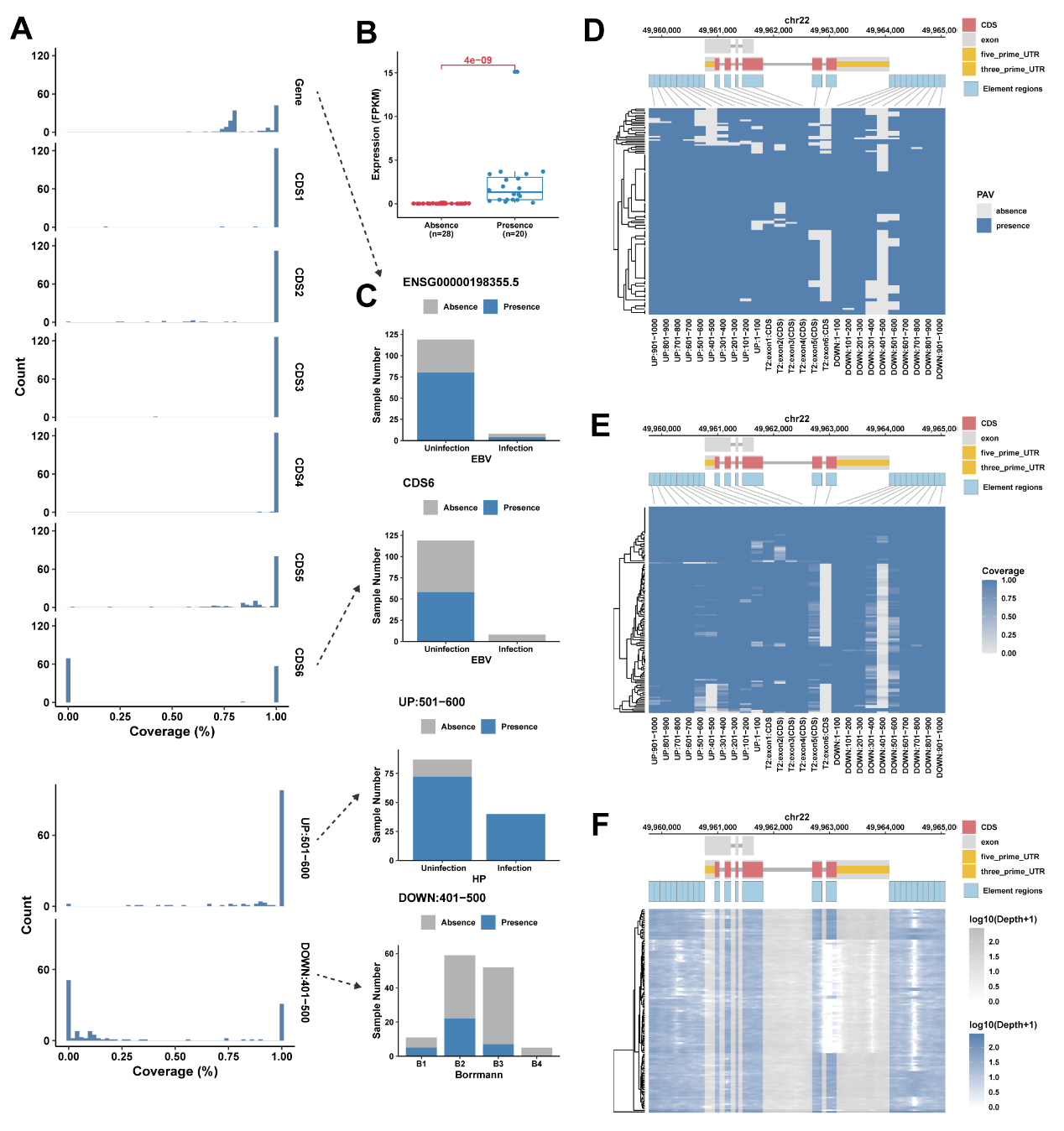
**

**S9 Fig. Element-level analyses for the *PIM3* gene. (A) The coverage distribution of the *PIM3* gene and its genetic elements.** Elements include the first to sixth CDS, a region 501-600 bp upstream, and a region 401-500 bp downstream. **(B) Gene expression of samples with and without the sixth CDS region of the gene.** The expression in the presence group was significantly higher than that in the absence group (*p* = 4e-9, Wilcoxon test). **(C) Associations between element regions and phenotypes.** The sixth CDS was significantly associated with EBV (*p* = 0.0076, Fisher's test). The 501-600 bp upstream was significantly associated with HP (*p* = 0.0028, Fisher's test). The 401-500 bp upstream was significantly associated with the Borrmann classification (*p* = 0.0062, Fisher's test). **(D-F) Element level PAV in terms of presence/absence, coverage, and sequencing read depth of elements in the *PIM3* gene.** Each 100bp upstream and downstream region is shown as an element.
